# Two haplotype-resolved genomes reveal important flower traits in bigleaf hydrangea (*Hydrangea macrophylla*) and insights into Asterid evolution

**DOI:** 10.1093/hr/uhad217

**Published:** 2023-11-09

**Authors:** Xingbo Wu, Sheron A Simpson, Ramey C Youngblood, Xiaofen F Liu, Brian E Scheffler, Timothy A Rinehart, Lisa W Alexander, Amanda M Hulse-Kemp

**Affiliations:** Department of Environmental Horticulture, Tropical Research and Education Center, University of Florida, Homestead, FL 33031, United States; Genomics and Bioinformatics Research Unit, USDA-ARS, Raleigh, NC 27695, United States; Genomics and Bioinformatics Research Unit, USDA-ARS, Stoneville, MS 38776, United States; Institute for Genomics, Biocomputing and Biotechnology, Mississippi State University, Starkville, MS 39762, United States; Genomics and Bioinformatics Research Unit, USDA-ARS, Stoneville, MS 38776, United States; Genomics and Bioinformatics Research Unit, USDA-ARS, Stoneville, MS 38776, United States; Crop Production and Protection, USDA-ARS, Beltsville, MD 20705, United States; Floral and Nursery Plants Research Unit, U.S. National Arboretum, USDA-ARS, McMinnville, TN 37110, United States; Genomics and Bioinformatics Research Unit, USDA-ARS, Raleigh, NC 27695, United States; Department of Crop and Soil Sciences, North Carolina State University, Raleigh, NC 27695, United States

## Abstract

The *Hydrangea* genus belongs to the Hydrangeaceae family, in the Cornales order of flowering plants, which early diverged among the Asterids, and includes several species that are commonly used ornamental plants. Of them, *Hydrangea macrophylla* is one of the most valuable species in the nursery trade, yet few genomic resources are available for this crop or closely related Asterid species. Two high-quality haplotype-resolved reference genomes of hydrangea cultivars ‘Veitchii’ and ‘Endless Summer’ [highest quality at 2.22 gigabase pairs (Gb), 396 contigs, N50 22.8 megabase pairs (Mb)] were assembled and scaffolded into the expected 18 pseudochromosomes. Utilizing the newly developed high-quality reference genomes along with high-quality genomes of other related flowering plants, nuclear data were found to support a single divergence point in the Asterids clade where both the Cornales and Ericales diverged from the euasterids. Genetic mapping with an F_1_ hybrid population demonstrated the power of linkage mapping combined with the new genomic resources to identify the gene for inflorescence shape, *CYP78A5* located on chromosome 4, and a novel gene, *BAM3* located on chromosome 17, for causing double flower. Resources developed in this study will not only help to accelerate hydrangea genetic improvement but also contribute to understanding the largest group of flowering plants, the Asterids.

## Introduction

Bigleaf hydrangea (*Hydrangea macrophylla*) is a perennial shrub belonging to the Hydrangeaceae family. It is an angiosperm, a member of the Asterids clade of flowering plants, and is classified in the order Cornales. The Asterids represent the largest group of flowering plants, with over 80 000 species, which comprises almost one-third of all flowering plants [1]. Taxonomists have utilized molecular data as they have become available to refine and correct phylogenies and perform phylogenetic dating to understand divergence times. Plastid, or chloroplast, DNA markers have been extensively used for this purpose with different numbers of markers, often integrating new phyla into the previously studied phylogenies [1–3]. As additional and more complete data become available, studies tend to utilize larger amounts of markers to help contend with problems such as incomplete lineage sorting and deal with conflicting results in past studies [4]. Most recently (2021), whole chloroplast genomes, or plastomes, have been used for developing a whole flowering plant phylogeny, where Cornales was the basal most order of the Asterids with an early unique speciation event diverging from the rest of the Asterids [4].

Among flowering plants, hydrangea is a valuable ornamental crop, with its center of diversity in southern Asia [5]. Of the four *Hydrangea* species widely available in the ornamental trade (*H. arborescens*, *H. macrophylla*, *H. paniculata*, and *H. quercifolia*), bigleaf hydrangea is the most popular among consumers for its multiple uses in gardens, landscapes, containers, and as a cut-flower plant. This ornamental crop was introduced into Europe around 1788 and soon gained popularity across the world as breeders and growers introduced hundreds of diverse cultivars [6]. The large, showy inflorescences of bigleaf hydrangea are its primary draw as both a landscape and floriculture plant. Important flower traits in hydrangea include inflorescence or flower shape, flower type, and flowering habit (reblooming or once-blooming) [7]. While hydrangea breeding in Asia and Europe focuses on flower color, shape, and types, the US breeding program aims to improve traits important for primary use in landscape environments, including the flowering habit, cold hardiness, and disease resistance [8]. Due to self-incompatibility, selection in hydrangea usually happens in the F_1_ generation, following a minimum 11-month growth period. The resultant progenies are highly heterozygous and have strong genetic and phenotypic variation, requiring the evaluation of large numbers of plants. Molecular selection using genetic markers could significantly reduce the space, labor, and cost of the breeding process in hydrangea.

**Figure 1:**
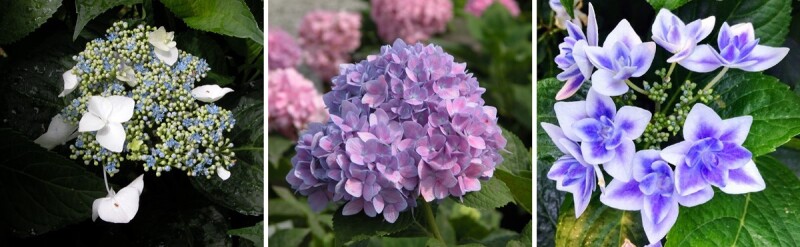
Flower morphology of *H. macrophylla*. Left: single-flower lacecap *H. macrophylla* cv. ‘Veitchii’; Middle: single-flower mophead *H. macrophylla* cv. ‘Endless Summer’; Right: double-flower *H. macrophylla* cv. ‘Double Delights’.

Flower architecture is one of the most attractive traits in hydrangea. Bigleaf hydrangea has two flower architectures: lacecap and mophead (Fig. 1) [7]. Lacecap hydrangeas usually show a flat inflorescence with a plane of fertile flowers surrounded by a ring of showy sepals (Fig. 1, left), while mophead hydrangeas have fertile flowers fully surrounded by showy sepals, exhibiting a rounded inflorescence [9] (Fig. 1, middle). Two cultivars representing the two distinct flower shape types are ‘Veitchii’ and ‘Endless Summer’ (Table 1). Veitchii is a prominent lacecap cultivar that occurred naturally and Endless Summer is a popular reblooming cultivar selected from planned crosses in a breeding program by Bailey Nurseries [6]. Studies of lacecap hydrangea and its mophead mutant indicated that mophead is caused by a single mutation occurring in a lacecap flower type, which leads to the replacement of partial inflorescences with decorative flowers on the upper nodes of the inflorescence axes [10, 11]. Linkage mapping showed that the inflorescence shape in hydrangea was controlled by a single recessive gene [12]. Uemachi *et al*. [11] proposed that an insertion of a long terminal repeat (LTR) retrotransposon into the locus controlling inflorescence type is responsible for this trait, but the candidate gene was not found due to a lack of genomic information.

**Table 1:** Contrasting traits exhibited by three different hydrangea cultivars (‘Veitchii’, ‘Endless Summer’, and ‘Double Delights’).

Cultivar	Inflorescence flower shape	Double flower	Reblooming	Sepal bluing under acidic soil
Veitchii	Lacecap	No	No	No
Endless Summer	Mophead	No	Yes	Yes
Double Delights	Lacecap	Yes	No	No

Hydrangea can be categorized into single- and double-flower phenotypes based on its flower structure (Fig. 1, right). The single-flower phenotype has only four petaloid sepals in the decorative flower, while in the double-flower phenotype, the petals and stamen are both mutated to petaloid sepals, leading to more compact flowers [12]. Double-flower phenotypes are often sterile due to the loss of reproductive organs [13], which increases the challenge for breeding double-flower hydrangea cultivars. Double flower is an elite trait that has increased the value of many ornamental plants for floriculture, including carnations (*Dianthus caryophyllus*), camellias (*Camellia japonica*), roses (*Rosa hybrida*), and petunias (*Petunia hybrida*) [14–17]. In hydrangea, there are two types of double-flower phenotypes that are nearly identical visually but controlled by two recessive genes independently [18]. It has been found that one phenotype is caused by a mutation in the *LFY* gene that causes leaf-like or sepal-like organs with no petal identity. In the other phenotype, the floral organs keep their petal identity with papilla cells but the causative gene is still unknown.

**Table 2:** Statistics of the two newly assembled hydrangea genomes and the previously published genome.

		*H. macrophylla* cv. Aogashima-1	*H. macrophylla* cv. Veitchii	*H. macrophylla* cv. Endless Summer
Reference		[18]	This study	This study
Platform		PacBio SeqII CLR	PacBio SeqII CLR	PacBio SeqII CCS/HiFi
Assembly size, Gb		2.23	2.21	2.22
Number of contigs		3779	6790	396
Contigs N50 (Kbp)		39.4	706.955	22 839
Number of scaffolds		3780	1172	169
Scaffolds N50 (Mb)		1.5	122.4	119.5
Number of pseudochromosomes		18	18	18
% of Assembly length in pseudochromosomes		49.33	96.38	98.65
Gap, %		0.10	0.16	0.01
LAI		17.50	21.12	21.85
Number of annotated genes		34 149	42 194	50 326
BUSCO (%)	Complete	63.1	89.8	93.0
	Single-copy	60.0	83.3	87.9
	Duplicated	3.1	6.5	5.1
	Fragmented	2.4	2.2	2.2
	Missing	34.5	8.0	4.8

In order to dissect traits such as these important flowering types, reference genomes have become essential tools for the plant breeding community. Traditional ornamental breeding techniques, such as cross and mutation breeding, have developed many new cultivars but are often laborious, costly, long, and target-unspecific processes. The availability of a genome sequence can not only provide information for developing tools for marker-assisted selection (MAS), which offers promise to accelerate the breeding process, but also enable candidate gene discovery and further support for precision breeding through gene editing, which are not currently available in hydrangea. The bigleaf hydrangea genome is composed of 18 chromosomes with a haploid genome size of ~2.2 gigabase pairs (Gb), which is the largest of the four major ornamental hydrangea species in the family [*H. arborescens* ~1.1 Gb, *H. quercifolia* ~970 megabase pairs (Mb), *H. paniculata* ~1.9 Gb] [19]. A draft genome sequenced on an Illumina short-read platform was first reported, where the total size of the assembly was 1.6 Gb, with 1 519 429 contigs [20]. However, this genome assembly is highly fragmented (large contig number) and not yet available to the public. Recently, a whole-genome sequencing project using PacBio continuous long reads (CLR) was conducted for the bigleaf hydrangea cultivar ‘Aogashima-1’, but the chromosome-level haplotype-resolved genome assembly was only 1.1 Gb, or about half the estimated haploid genome size [18].

Despite its important role in the worldwide ornamental industry, there are few genomic resources developed for hydrangea, which largely hampers the understanding of genome structure and genetic mechanisms for important traits that are critical to implement modern breeding techniques such as MAS. As the second most valuable deciduous shrub class in the USA, the development of a high-quality reference genome containing the majority of the haploid genome size in chromosome-scale scaffolds, or pseudochromosomes, is vital for hydrangea. This is essential for hydrangea to enter the era of modern plant breeding to benefit the green industry with elite flower traits such as mophead inflorescence and double flower. The objective of this study was to (i) build a high-quality pseudochromosome scale reference genome for two important hydrangea (*H. macrophylla*) cultivars with contrasting flower characteristics, (ii) provide a high-quality gene annotation, (iii) confirm the previously reported double-flower candidate gene and identify the second unknown gene associated with the double-flower trait, (iv) identify the candidate gene for the inflorescence flower shape trait, and (v) utilize high-quality genomes to evaluate the evolutionary history of *Hydrangea* among flowering plants.

## Results

### Whole-genome sequencing and haplotype genome assembly

Flow cytometry estimations showed that the genome size of ‘Veitchii’ and ‘Endless Summer’ was 2.1 and 2.2 Gb, respectively (Fig. S1). Based on the genome size estimation, a total of 250-Gb reads were generated for ‘Veitchii’ using PacBio CLR technology and 100-Gb reads were generated for ‘Endless Summer’ using PacBio circular consensus sequencing (CCS), or high-fidelity (HiFi), technology.

The ‘Veitchii’ PacBio long reads were assembled into 10 792 contigs with a total size of 3.35 Gb. Five chloroplast genomes from *Arabidopsis thaliana* (NC_000932.1, NC_037304.1), *Hydrangea serrata* (KU140669.1), *Hydrangea aspera* (MG524992.1), *Hydrangea febrifuga* (MN380702.1), and *Hydrangea luteovenosa* (MF370556.1) were BLASTed against the genome, and 16 highly similar sequence reads were removed from the nuclear genome assembly. Due to the large genome size with high levels of heterozygosity, a customized approach (see Materials and methods, Fig. S2) involving a cyclical manual curation process with HiC data was used to separate the primary and alternative contigs, as no haplotype-purging pipelines were found to be effective. Initially, a total of 1153 contigs (<50 Kb) were separated from the large contigs. The remaining 9623 large contigs were used for the haplotype-purging process. The HiC generated 486 289 871 paired sequence reads (174.8 Gb), which were used for haplotype purging, and 5846 contigs were identified for the primary assembly. The final primary haplotype assembly contained 6790 contigs (including the initially separated small contigs), containing a total length of 2.30 Gb with contig N50 value of 709 Kb. The secondary haplotype assembly contained 3776 haplotigs, with total length of 1.05 Gb with contig N50 of 404.29 Kb. To obtain the chromosome-level genome, 83.9% of the HiC reads were mapped to the final primary assembly and 2672 scaffolds were generated through contact mapping with the Juicer toolbox [21]. Eighteen superscaffolds (accounting for 96.38% of the assembled genome) were identified as pseudochromosomes with N50 of 123.11 Mb for the primary haplotype (Table 2).

The *hifiasm* pipeline was used to assemble the 100-Gb PacBio CCS reads for ‘Endless Summer’ into a primary haplotype containing 397 primary contigs with total length of 2.27 Gb and contig N50 of 22.8 Mb. The secondary haplotype contained 4970 alternative contigs with total length of 1.61 Gb and contig N50 of 1.6 Mb. The HiC reads from ‘Veitchii’ were mapped to the primary assembly (80% map rate) and resulted in 169 scaffolds, with 18 of them representing the 18 pseudochromosomes with N50 of 119.5 Mb (Table 2). During this comparative scaffolding process between cultivar assemblies, an additional set of alternative contigs were identified: 209 alternative contigs were identified from the ‘Veitchii’ primary assembly and one contig was identified from the ‘Endless Summer’ primary assembly. After moving these identified contigs to the secondary haplotype, the primary haplotypes contained a total of 5847 contigs for ‘Veitchii’ and 396 contigs for the final ‘Endless Summer’ reference genomes. The pseudochromosome-level primary and secondary haplotypes were compared for the final reference genomes (Fig. S3). Benchmarking Universal Single-Copy Orthologs (BUSCO)-identified complete genes (using the Embryophyta core gene set) found the ‘Veitchii’ and ‘Endless Summer’ genomes to have 89.80% and 93.0%, respectively, indicating a high level of completeness. In addition, the adjusted LTR assembly index (LAI) score was 21.12 for the ‘Veitchii’ assembly and 21.85 for ‘Endless Summer’ assembly, which indicates both have reached the ‘gold quality’ level according to the algorithm standard [22].

### Genome annotation

Both *ab initio* and homology-based approaches were used to annotate the ‘Veitchii’ and ‘Endless Summer’ reference genomes. A total 79.09% of the assembled ‘Veitchii’ genome was identified as repetitive, including 40.67% LTR elements and 1.37% DNA transposons (Table S1). In addition, the *Gypsy/DIRS1* and *Ty1/Copia* elements accounted for 32.01% and 8.16% of the genome. Illumina RNA sequencing-generated reads from seven ‘Veitchii’ plant tissues as well as the high-quality isoforms from Iso-Seq CCS reads [18] were used to annotate the ‘Veitchii’ genome using Braker2 [23]. A total of 59 474 protein-coding genes were identified in the primary assembly with average gene and coding sequence length of 4837 and 960 bp, respectively. Gene functions, gene ontology (GO) terms, and Kyoto Encyclopedia of Genes and Genomes (KEGG) objects were assigned by EnTAP using protein databases including UniProt, EggNOG, and RefSeq for each of the predicted genes. In the primary assembly annotation, 42 194 out of 59 474 (70.9%) genes were functionally annotated. Of which, 41 288 were annotated with GO terms (27 366 genes with biological processes, 21 999 genes with cellular components, and 27 167 genes with molecular functions). Of the functionally annotated set, the average gene length was 5942 bp with a mean coding sequence length of 1122 bp (Table S2).

**Figure 2:**
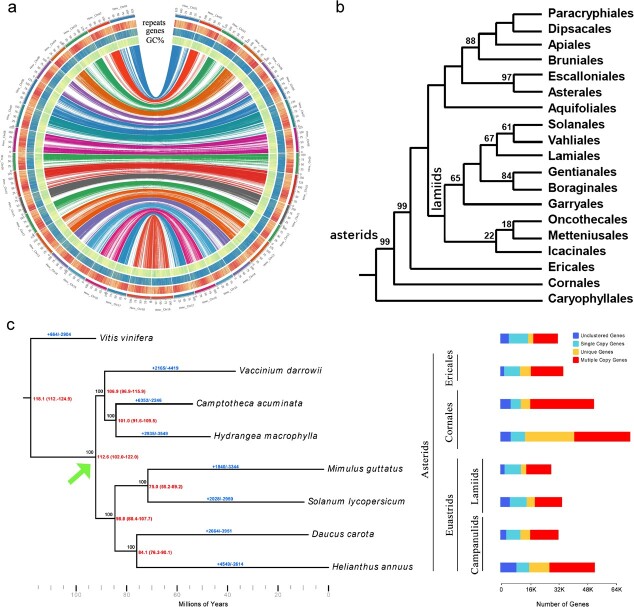
Comparative genomics and phylogenetic relationship analyses. a) Genome synteny of primary haplotypes of the ‘Endless Summer’ and ‘Veitchii’ genome; b) Previously published phylogenetic relationship of the Asterids clade, derived from plastome data modified from Figure 1 of Li et al. (2021), and c) Phylogenetic relationship of *H. macrophylla* (Endless Summer genome) with seven other plant species. Numerical value beside each node to the right shows the estimated divergence time (MYA, million years ago), and to the left shows bootstrap values. Positive and negative blue numerical values on each species branch represent the number of expanded and contracted genes, which are also plotted to the right. The arrow in 2c (green) indicates the divergence point of the euasterids lineage (bottom) from Cornales and Ericales.

The ‘Endless Summer’ genome had fewer repetitive regions (76.44%) but a higher portion of LTR elements (51.15%), DNA transposons (4.74%), *Gypsy*/*DIRS1* (40.02%), and *Ty1/Copia* (10.41%) compared with the ‘Veitchii’ genome (Table S1). The same process as indicated above for ‘Veitchii’, but using ‘Endless Summer’-derived Illumina RNA-sequencing reads and 116 634 Iso-Seq isoforms [18] was used for the annotation of the ‘Endless Summer’ genome. A total of 71 773 protein-coding genes were identified in the primary assembly and 50 326 out of 74 554 (68.8%) genes were functionally annotated. Of the functionally annotated set, the average gene length was 5187 bp with mean coding sequence length of 1143 bp (Table S2).

### Comparative genomics and phylogenomic analyses

‘Veitchii’ and ‘Endless Summer’ have many contrasting traits, such as sepal color, inflorescence shape, and disease resistance that are of interest for breeding programs. Comparing the primary haplotype assemblies showed a high level of synteny as expected (Fig. 2a). All genes from both haplotypes of each cultivar assembly (total of 215 058 genes) were included for comparative orthologous analyses; out of them, 193 297 genes were assigned to 47 649 orthogroups (Table S3). There were 109 317 genes from ‘Endless Summer’ and 83 980 genes from ‘Veitchii’ assigned to the orthogroups. A total of 10 716 single-copy orthogroups were identified among the two genomes (Table S3). In ‘Endless Summer’, there was a total of 23 283 unique genes (not found to be shared with ‘Veitchii’) assigned to 4599 orthogroups, while 7950 genes were unique in ‘Veitchii’ and assigned to 2277 orthogroups. Among the genes that are unique to the two cultivars are genes with functions of disease resistance from the NBS-LRR gene family (RPP8, RPP13, RPM1, and R1A), MLO gene family (MLO13), and RGA gene family (RGA1, RGA2, RGA3, and RGA4), as well as flower regulation genes such as MADS-Box genes SVP and JOINTLESS, the CLAVATA3 gene family, and *ULTRAPETALA*, a key negative regulator of cell accumulation in shoot and floral meristems (Table S4).

Ortholog analyses of seven plant species (*Camptotheca acuminata*, *Daucus carota*, *Helianthus annuus*, *Mimulus guttatus*, *Solanum lycopersicum*, *Vaccinium darrowii*, *Vitis vinifera*) [24–30] and the ‘Endless Summer’ genome, used to represent *Hydrangea* as the highest quality genome available, assigned 298 400 genes (88.6% of total) to 29 395 orthogroups (Table S5). Fifty percent of all genes were contained in the largest 6884 orthogroups, which all contained 13 or more genes. There were 8914 orthogroups with all species present and 607 of these consisted entirely of single-copy genes, which were used for phylogenetic analysis. Of the identified gene families, 2967 gene families containing 27 162 genes were unique to the ‘Endless Summer’ genome. A total of 3452 expansions and 3178 contractions in the gene families were specific to hydrangea. The evolutionary relationships among hydrangea and the seven other plant species largely supported the expected phylogeny of Asterids clade, with *V. vinifera* as the expected outgroup (Fig. 2b and c). There was found to be a monophyletic breakpoint for the Cornales and Ericales. The estimated divergence times indicated that hydrangea and blueberry diverged ~101.8 million years ago (Mya) (Fig. 2c). As expected, hydrangea has large numbers of duplicated genes, compared with the other species, indicative of whole-genome duplication (WGD) (Fig. 2c, right side).

The types of duplication observed in the protein-coding genes of hydrangea, as indicated by the OrthoFinder [31] classifications, included 10 251 (~10.2%) gene pairs that had undergone WGD, 1513 (~1.5%) gene pairs in tandem duplication, 13 654 (~13.6%) gene pairs in transposon duplication, 72 840 (~72.3%) gene pairs in dispersed duplication, and 2380 (~2.4%) gene pairs in proximal duplication.

### Genetic mapping of the inflorescence locus

Of the 341 F_1_ progeny, 205 were lacecap inflorescence type while 126 had mophead inflorescences. Ten progeny failed to flower and thus were coded as missing. Libraries of 341 F_1_ progeny yielded 1 billion reads (an average of 2.7 million reads per progeny), which resulted in 376 153 raw single-nucleotide polymorphisms (SNPs) by using the Tassel UNEAK SNP calling pipeline. By choosing SNPs that were polymorphic between the parents and segregating in the F_1_ population, a total of 9018 SNPs were selected for genetic analyses. After filtering for missing data and allele frequency, the final dataset that was used as input for mapping included 2668 SNPs for 334 progeny. Seven progeny were excluded from mapping for having >20% missing data.

**Figure 3:**
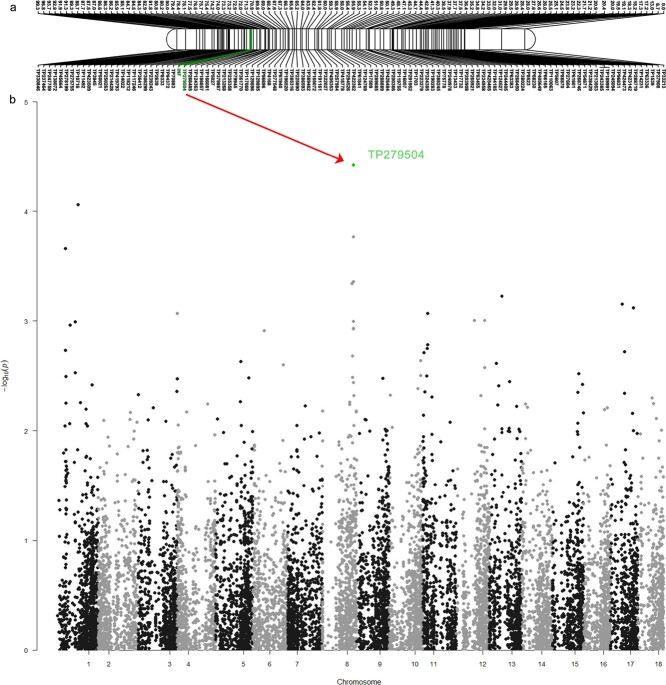
Mapping of candidate genes for the inflorescence trait (*INF)*. a) Linkage mapping showing Linkage Group 04 including the inflorescence trait phenotypic marker *INF*; b) Genome-wide association mapping study.

The final linkage map consisted of 2489 markers, including 2488 SNP markers and one phenotypic trait marker for inflorescence type, *INF*. These 2468 SNP markers were distributed in 18 linkage groups (LGs) with the total map length of 1848.92 cM at logarithm of odds (LOD) score of 34 (Table 3, Fig. S4). The linkage groups length ranged from 90.25 cM of LG 18 to 122.69 cM of LG 16, with the number of markers per linkage group ranging from 178 on LG 9 to 97 on LG 13. The average number of markers per LG was 138, with an average marker density of one marker per 0.74 cM. The maximum gap varied from 3.20 cM (LG 6) to 13.10 cM (LG 16). The inflorescence locus (*INF*) was mapped to 20.87 cM on LG 4 (Fig. 3). Two SNP markers flanking the *INF* locus were TP70883 (19.88 cM) and TP279504 (21.26 cM). The upstream marker TP70883, 0.99 cM away from *INF*, was segregating in both parents (marker type hk × hk), while the downstream marker TP279504, 0.39 cM from *INF*, was segregating only in the female parent (marker type lm × ll). Given the genetic nature of the inflorescence trait, TP279504 became the marker of interest associated with the inflorescence locus *INF*.

**Table 3:** Distribution of SNPs derived from the genetic map of ‘Veitchii’ × ‘Endless Summer’ mapping population.

Linkage group (LG)	No. of SNPs	Genetic length (cM)	Marker interval (cM)	Max gap (cM)
1	166	111.38	0.67	12.80
2	152	102.36	0.67	4.10
3	141	94.45	0.67	4.20
4	111	99.31	0.89	6.70
5	144	113.29	0.79	4.50
6	142	104.46	0.74	3.20
7	137	113.08	0.83	12.90
8	152	91.78	0.60	4.30
9	178	101.33	0.57	11.20
10	138	110.81	0.80	10.80
11	110	99.25	0.90	5.20
12	170	97.12	0.57	5.20
13	97	97.31	1.00	4.30
14	146	100.84	0.69	3.40
15	153	102.00	0.67	8.40
16	119	122.69	1.03	13.10
17	125	97.20	0.78	5.00
18	107	90.25	0.84	3.60
Total	2488	1848.92	0.74	12.80

The association study identified 180 575 SNPs using a reference-guided approach. The reported SNP marker, *Hy_CAPS_Inflo*, was found to be highly associated with the inflorescence trait as described previously [32]. The *Hy_CAPS_Inflo* marker was located on chromosome 4 of the ‘Endless summer’ genome, the exact same location of *INF* that was identified by linkage mapping (Fig. 3).

### *CYP78A5* is the candidate gene for inflorescence shape

**Figure 4:**
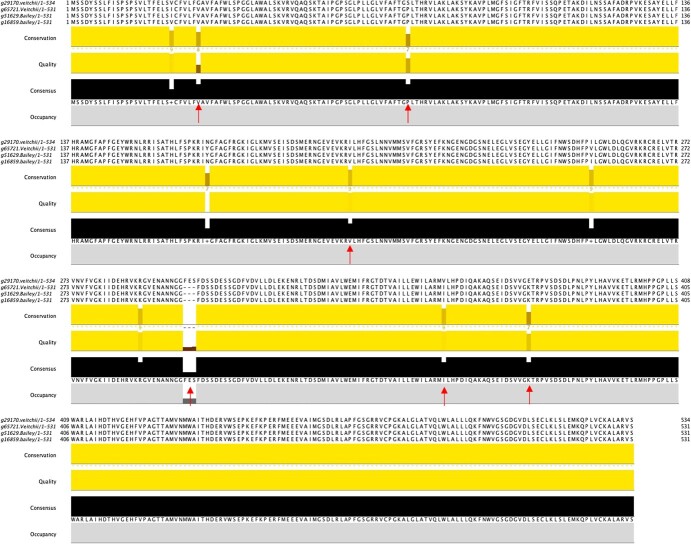
Investigating the candidate gene for the inflorescence trait. Protein sequence of *CYP78A5* gene in the four haplotypes of ‘Veitchii’ and ‘Endless Summer’ genomes. Red arrows indicate positions of potential causative variation.

Mapping of four molecular markers [simple sequence repeats (SSRs), SNPs, and cleaved amplified polymorphic sequence (CAPS)] and the *INF* trait identified four genomic regions on chromosome 4 on each haplotype of the two assembled genomes (Table S6). The genomic regions encompassed by highly selective SNP markers were 424 Kb in the ‘Endless Summer’ haplotypes and 36 Kb in ‘Veitchii’ haplotypes, while the SSR marker was mapped to 9.2 Mb downstream of its closest SNP marker A109A110. The genomic regions had 23 and 27 genes in the two haplotypes of ‘Endless Summer’ genome, and 19 and 18 genes in the two haplotypes of the ‘Veitchii’ genome. Among these identified genes, a single-copy CYP78A5-like gene associated with floral organ regulation was identified in all four haplotypes. Protein sequences of the four gene copies identified 5 sequence variants and one InDel that were homozygous in ‘Endless Summer’ and heterozygous in ‘Veitchii’ (Fig. 4), following the expected trait architecture and phenotypes of the cultivars.

### Novel candidate gene *BAM3* is associated with double flower

**Figure 5:**
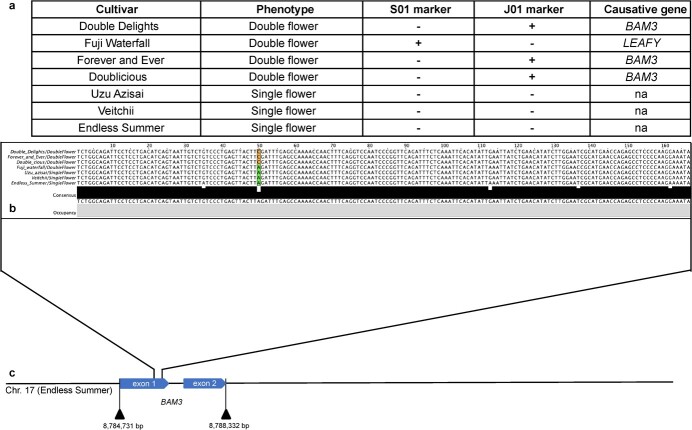
Identifying the second candidate gene for double flower. Nucleotide sequence variation on the first exon of the *BAM3* gene in six hydrangea cultivars. a) Phenotyping and genotyping of six hydrangea cultivars. b) Sequence alignment of J01 marker associated with double-flower trait [18] in six hydrangea cultivars. ‘Fuji Waterfall’ is a double-flower cultivar with mutated *LEAFY* gene. c) J01 marker was mapped to the *BAM3* gene on Chr. 17 of the ‘Endless Summer’ primary haplotype.

Two primers, InDel S01 and CAPS J01 [18], were used to detect the causative genes responsible for the double-flower trait in seven hydrangea cultivars (Fig. 5a). In seven cultivars that were tested for double-flower alleles, ‘Fuji Waterfall’ contained the allele associated with an InDel marker S01. ‘Double Delights’, ‘Forever and Ever’, and ‘Doublicious’ contained the alleles associated with the CAPS maker J01. None of the single-flower cultivars, ‘Uzu Azisai’, ‘Veitchii’, and ‘Endless Summer’, had recessive alleles.

The two markers were mapped to the two assembled genomes to identify potential candidate genes for the double-flower trait (Fig. 5b). The InDel marker S01 was located in the genomic region of gene g28477 that ranges from 47 444 151 bp to 47 446 052 bp on chromosome 4 of the ‘Veitchii’ genome and g50696 that ranges from 95 262 761 bp to 95 268 295 bp on chromosome 4 of the ‘Endless Summer’ genome. Both genes are homologous genes of *LFY*, an important transcription factor in flower development. The CAPS marker J01 was mapped to both assembled genomes*.* The SNP of this marker was located on 8 786 170 bp of chromosome 17 in ‘Endless Summer’ and 4 988 780 bp of chromosome 17 in ‘Veitchii’. The identified location was in the first exon of gene g20959 in ‘Endless Summer’ and gene g1913 in ‘Veitchii’. Both genes are homologous genes of *BAM3*, an important gene that regulates both shoot and flower meristem function in Arabidopsis (Fig. 5c).

## Discussion

In the present study, two high-quality pseudochromosome scale assemblies of the bigleaf hydrangea genome were produced using a combination of long-read, Chromatin-capture (HiC), and short-read sequencing. These are the most contiguous, complete, and high-quality bigleaf hydrangea genomes produced to date based on evaluation statistics performed for the present and previously published genomes [18]. These high-quality genomes will enable the study of genomic features associated with important phenotypic and agronomic traits of this important ornamental species.

Deep coverage of long sequencing reads, HiC mapping rate, and suitable technical pipelines play an important role to ensure a high-quality genome for plant species with large genome sizes such as bigleaf hydrangea. In this study, 150× coverage PacBio continuous long reads were produced for ‘Veitchii’ genome assembly as compared with the ~40× coverage PacBio HiFi reads for the ‘Endless Summer’ genome. However, the high coverage reads were still not adequate to purge haplotigs from the ‘Veitchii’ assembly due to the high heterozygosity of the genome. A special purging pipeline developed in this study that utilizes HiC reads could be used as a reference for future haplotype purging of large, highly heterozygous genomes, if traditional software does not appear to purge correctly. In this study, 80% of the HiC reads were mapped to the purged primary assembly, while only 697 Mb out of 105.3 Gb HiC data (0.67%) was mapped to the ‘Aogashima-1’ genome. The poor HiC mapping rate between cultivars could be caused by the presence of unpurged haplotigs and overlapping heterozygous regions, which may have caused difficulty in scaffolding of the ‘Aogashima-1’ genome.

This study provides the first genome-scale comparison of multiple bigleaf hydrangea cultivars. Nearly 3-fold more cultivar specific homolog genes were identified in ‘Endless Summer’ than ‘Veitchii’, probably due to the higher quality of the ‘Endless Summer’ genome and resultant higher annotation percentage than the ‘Veitchii’ genome. Many important gene types like disease-resistance and flower developmental gene orthologs were found in those unique gene groups. Apart from putative disease-resistance genes from NBS-LRR family, ‘Endless Summer’-specific gene orthologs were more related to phytophthora (RPP13, PGA gene family) and powdery mildew (MLO13) disease, while there were more genes associated with downy mildew disease (RPP8) and flower development (MADS-box) in ‘Veitchii’. Selections of parents with contrasting valuable traits are vital for breeding programs, and the availability of high-quality genome sequences for these parents has shown value here and in other studies to integrate pangenomic approaches [33]. ‘Endless Summer’ is a popular cultivar with mophead inflorescences, attractive sepal color variations, and continuous blooming. However, ‘Endless Summer’ is susceptible to powdery mildew while ‘Veitchii’ shows some powdery mildew tolerance. These cultivar-specific genes will be valuable resources for future genetic studies of disease resistance and flower traits and potential gene editing targets for crop improvement.

Inflorescence shape is an important trait in hydrangea but little information about the physiological and molecular mechanism of this phenotype is known. Though several highly efficient molecular markers have been developed through linkage mapping and genome-wide association mapping study (GWAS) [7], the candidate gene responsible for this valuable trait remained unknown due to lack of complete genomic resources. Through genome sequencing and associated mapping studies, the *CYP78A5* gene was identified in this study as the candidate gene for the inflorescence trait. Numerous studies indicate the *CYP78A5* gene plays a role in regulating directional growth at the meristem/organ boundary and is required for the promotion of leaf and floral organ growth and for the prolongation of the plastochron in Arabidopsis [34–36]. Lack of *CYP78A5* in Arabidopsis results defected flower organogenesis and reduced fertility [34]. In bigleaf hydrangea, mophead phenotype cultivars are much less fertile than lacecap cultivars due to a reduction in number of fertile flowers, placement of fertile flowers beneath the sepals, and the compression of fertile flowers in the axils of the inflorescence itself.

In this study, we identified a *BAM3* (barely any meristem 3) gene that encodes a receptor kinase-like protein associated with the marker that segregates with one of the two double-flower phenotypes. The most well-known double-flower-related gene, a putative *LFY* mutation, was found under the well-established ABCE gene model in one of the double-flower phenotypes of bigleaf hydrangea [18]. This is the first report of the novel function of *BAM3* leading to a double-flower phenotype*.* The *BAM3* gene is a regulatory gene for meristem function [37]. In Arabidopsis, mutations of *BAM* genes lead to phenotypes consistent with the loss of stem cells at the shoot and flower meristem. In addition, the BAM gene family (*BAM1*, *BAM2*, *BAM3*) was also found to play an essential role in male gametophyte development, as well as ovule specification and function [37, 38]. This finding will provide unique insight in studying the functionality of the BAM gene family in floral organ development, an important physiological process with broad impacts in horticultural and agricultural production.

Multiple lines of experimental evidence were essential to determining the candidate genes determined and presented in this study, as unlike other plant systems, a transformation and regeneration system functional across different genotypes is currently unavailable in hydrangea [39, 40]. Additional work is needed to establish a system beyond the very basic principles to enable routine testing of important trait candidate genes in model varieties. However, as presented here, multiple experimental lines of evidence can still provide confidence for developing markers for use in downstream MAS for important flower characteristics. In the future when a better transformation system is available, it will be beneficial to return to these previously identified candidate genes to confirm their function.

Hydrangeas contain high genetic diversity and display wide phenotypic variation within and among species. WGD is an important evolutionary event leading to plant speciation through functional diversification of two copies of genes. There are 42 reported species in the *Hydrangea* genus, of them *H. macrophylla*, *H. arborescens*, *H. quercifolia*, and *H. paniculata* are important species in the nursery trade with varying genome sizes. *Hydrangea macrophylla* has the biggest genome size (2.2 Gb) as compared with *H. quercifolia* (~970 Mb), *H. arborescens* (~1.1 Gb), and *H. paniculata* (~1.9 Gb). Hydrangeas contain variation for many traits such as the extent of sepal color change (response to soil pH or metal compounds), tolerance to hyperaccumulation of heavy metals, flower differentiation and development (as seen in the perpetual flowering of *H. arborescens* and *H. paniculata*), the shape of the inflorescence (such as the ‘pyramid’ shape of *H. quercifolia*), flower greening (natural greening in decorative flowers after flowering or infection with phytoplasma), climbing traits (as seen in *H. petiolaris* and *H. hydrangeoides*), fragrance (as seen in *H. quercifolia*), and disease resistance. However, traditional plant breeding methods have not been successful in identifying and utilizing this genetic variation due to the variations in genome size among species and difficulty in obtaining F_2_ or backcross populations within species [41, 42].

Due to the increasing worldwide demand for improved hydrangea cultivars, alternative methods are needed to identify and utilize the genetic diversity already present in the genus. Hydrangeas enjoy worldwide popularity across several green industries. For example, hydrangea is a top 10 plant in the worldwide floriculture industry, which had an estimated value of 42.43 billion USD in 2018 [39]. In the USA alone, demand for floriculture plants increased by 64% between 2007 and 2014 (USDA NASS) [43]. Novel plants and floral forms drive consumer demand for ornamental plants, such that flower shape, color, and length of bloom are of primary value to industry stakeholders to maintain growth and profitability [39]. These traits also have broad implications for ecology, where knowledge of genes underlying floral traits can be used to test hypotheses around speciation, evolution, and reproduction. The two high-quality *H. macrophylla* genomes developed in this study will be a foundational resource for the genetic analysis of these important traits and enable precision breeding within the genus *Hydrangea*.

In addition, the hydrangea (Hydrangeaceae family), along with dogwoods (Cornaceae family) are the two most horticulturally important species in the Cornales order that are basal Asterids. Study of the early evolution of plant species in this order has been difficult due to rapid radiation during the Cretaceous period [44]. Reference genomes are valuable resources for phylogeny and genomic studies to reveal the rise and extinction of new species. The reference genomes developed here serve as foundational resources to study the evolution of Asterid plants. Together with recently published high-quality genomes of two other basal asterids, *C. acuminata*, another Cornales, and blueberry (*Vaccinium darowii*), an Ericales, we have evidence that supports a single event where both the Cornales and the Ericales diverged from the euasterids (Fig. 2c, green arrow) contrary to previous studies using organellar DNA [2–4]. This result was confirmed independently in two separate phylogenetic software (IQ-TREE v2.2.0.3 and RAxML-NG v1.1) with 1000 iterations in both software producing bootstrap values of 100 for the node on the tree. As long-read technologies improve, availability of high-quality genomes for plants has rapidly increased, enabling new investigations. These will enable further investigation into the true phylogeny of flowering plants, as well as the whole tree of life. The availability of additional high-quality Asterid genomes will not only enable further study of the phylogenetic relationships, but to help understand important biological mechanisms across flowering plants and provide valuable tools for improvement in commercially valuable species.

## Materials and methods

### Plant materials and genome size estimation

Bigleaf hydrangea (*H. macrophylla*) cultivars ‘Veitchii’ and ‘Endless Summer’ were selected for whole-genome sequencing. Both cultivars are commercially available and maintained at the Otis L. Floyd Nursery Research Center in McMinnville, TN. ‘Veitchii’ is an old cultivar that was introduced to the USA around the beginning of the 20th century [45]. ‘Veitchii’ has a small, bushy shrub growth pattern with glossy dark-green leaves and small flowers at both primary and lateral shoots. ‘Veitchii’ is a single-flower, lacecap cultivar with white sepals and white, pink, or blue fertile flowers depending on the pH−/aluminum level in the soil. It is cold hardy and powdery mildew tolerant and thus is often used in breeding programs [46, 47]. ‘Endless Summer’ is a widely popular hydrangea cultivar that was named and marketed in 2000 [45]; previously this cultivar was called ‘Bailmer’. ‘Endless Summer’ has a mophead inflorescence ranging from pink to blue depending on the aluminum concentration in the soil; it is a reblooming cultivar (flowering on the current season’s growth) and susceptible to powdery mildew. The traits of different hydrangea cultivars were summarized in Table 1.

The 2C DNA content for each cultivar was determined using flow cytometry according to Dolezel *et al*. [48]. The *Pisum sativum* L. ‘Ctirad’ (2C = 9.09 pg) was used as the internal standard. Young hydrangea leaf samples (~0.3 cm^2^) and the standard (~0.9 cm^2^) were processed with extraction buffer, filtered with CellTrics filter (Partec) and loaded into Partec CyFlow Space flow cytometer (Partec GmbH, Münster, Germany) with 488 nm excitation from a blue solid-state laser. At least 5000 nuclei were analyzed to determine the single peak at CV < 4%. The software FloMax version 2.70 (Quantum Analysis GmbH) was used for the data analysis. The 2C DNA content was calculated as follows: [mean fluorescence value of sample × 2C content of standard/mean fluorescence value of standard].

### Genome sequencing, assembly, and scaffolding

High-molecular weight (HMW) DNA was extracted from leaf tissues for genome sequencing. Nuclei were isolated using the Bionano Prep Plant Tissue DNA Isolation kit (Bionano Genomics, San Diego, CA, USA). Subsequently, HMW DNA was extracted from the nuclei using the Circulomics Nanobind Plant Nuclei Big DNA kit (Pacific Biosciences, Menlo Park, CA, USA). The genomic DNA was validated by gel electrophoresis and quantified by spectrophotometry (Nanodrop, Thermo Fischer Scientific) and fluorimetry (Qubit V3.0, Thermo Fischer Scientific). Genomic libraries were generated for PacBio long-read sequencing and Illumina short-read sequencing for both whole genome and HiC according to manufacturer’s protocols. The ‘Veitchii’ genome was sequenced using the PacBio Sequel (Pacific Biosciences, Menlo Park, CA, USA) system in CLR mode. The MECAT2 pipeline [49] was used for genome assembly with default parameters. The draft assembly was self-corrected using the PacBio long reads following the Arrow pipeline [50]. Chloroplast and mitochondrial read contaminants were removed from the assembly. HMW genomic DNA from ‘Endless Summer’ was sequenced on the PacBio Sequel system in CCS or HiFi mode. The raw reads were cleaned and assembled using *hifiasm* (v15.4) [51] with default parameters.

Due to the high heterozygosity in combination with increased error rates in the CLR long-read technology causing fragmentation of the assembly, the raw assembly of ‘Veitchii’ was manually partitioned into primary and alternative haplotypes with HiC sequence reads using Juicer [21], ntJoin [52], and Gepard [53]. Specifically, short contigs that were <50 Kb were separated from the total assembly. The remaining long contigs were assembled manually by visualization into scaffolds with Juicer. Each scaffold was self-aligned and alternative haplotypes were identified from the interactive plot map generated by Gepard. The process was repeated several rounds. The purging approach is demonstrated in Fig. S2. For each round, the BUSCO scores [54], the mapping rate of genotyping-by-sequencing (GBS) subset reads from Wu *et al*. [55], and the genome size were used to assess the consequences of purging the identified set of contigs for that round for gene set completeness and duplication. For the ‘Endless Summer’ genome, the raw assembly was scaffolded using the ‘Veitchii’ HiC reads in Juicer without correction (*−r* 0).

The primary genome assemblies of ‘Veitchii’ and ‘Endless Summer’ were aligned with minimap2 v2.17 in asm5 mode [56]. The alignments were visualized as a dotplot to identify any remaining alternative haplotypes with D-GENIES [14]. Alternative contigs identified in the primary assemblies were reclassified into alternative contig sets. For both genomes, the alternative contigs were scaffolded based on the primary assembly using RagTag [57]. The sequence gaps in both assemblies were filled by Dentist [58] using the raw PacBio long reads for each sample, respectively. Finally, both primary and secondary ‘Veitchii’ assemblies (not ‘Endless Summer’) were polished with Illumina short reads using Pilon (v1.22) [59]. The chromosome numbers of both assemblies were identified based on relationship through whole-genome alignments to the chromosomes of the *H. macrophylla* ‘Aogashima-1’ genome [18] (Fig. S5).

### Genome annotation

For each of the cultivars used for genome assembly, total RNAs were extracted from seven tissue types, including sepals, buds, flowers, nodes, internodes, leaves, and roots, and subjected to library preparation according to the manufacturer’s instructions. The Illumina NovaSeq platform was used to generate 150 bp paired-end reads. The quality control of RNA-seq reads was implemented by fastp (https://github.com/OpenGene/fastp) and FastQC (http://www.bioinformatics.babraham.ac.uk/projects/fastqc) for quality control. Additionally, the publicly available Iso-seq reads on NCBI (PRJDB9979) were used for genome annotation.

BRAKER2 [23] was used to annotate the assembled genomes. For both assemblies, the repetitive elements were identified and masked by RepeatModeler [60] and RepeatMasker [61]. The high-quality RNA reads were mapped to masked assemblies using STAR v2.7.3a [62]. In addition, Iso-seq reads were aligned to the assemblies to identify potential gene models using minimap2 [56]. The aligned RNA reads then served as transcript evidence to annotate each assembly using BRAKER2 [23]. EnTAP [63] and gFACs [64] were used to filter the functional and structural annotations of the predicted gene models. The GO terms, KEGG entries, and gene functions were assigned to the gene models using RefSeq [65], Uniprot [66], and eggnog [67]. Genome assemblies and filter gene models were assessed by the analysis of BUSCO v3.0 using the embryophyta lineage. Genome continuity was evaluated using LAI in the LTR_retriever package [22].

### Comparative genomics analyses

The two haplotypes of ‘Veitchii’ and ‘Endless Summer’ assemblies were aligned using CoGe SynMap (parameters *-D* 12Kb *-A* 50 *-Dm* 48Kb) [68]. Additionally, the primary assemblies of ‘Veitchii’ and ‘Endless Summer’ were also aligned to each other with the same parameters stated above. Alignment results were extracted and visualized via Circos software [69].

OrthoFinder [31] was used to identify the homologous genes by analyzing 123 165 proteins from the ‘Endless Summer’ genome and 91 893 proteins from the ‘Veitchii’ genome. All-to-all BLASTP analysis was performed between the primary assemblies of ‘Endless Summer’ and ‘Veitchii’ genomes with an e-value of 1e-10. The first match hits from the reciprocal BLASTP results were retained for ontology analysis. The same approach was then used to identify orthologous genes from the ‘Endless Summer’ primary assembly and six other species from the Asterids clade, including happy tree (*C. acuminata*) [24], carrot (*D. carota*) [25], sunflower (*H. annuus*) [26], monkey flower (*M. guttatus*) [27], tomato (*S. lycopersicum*) [28], and blueberry (*V. darrowii*) [29], with grape (*V. vinifera*) [30] as outgroup species for phylogenetic analyses. The protein sequences of orthogroups containing single genes from each species were aligned using MUSCLE v5.1 [70]. The best model was then predicted by ModelTest-ng v.0.1.7 [71]. Both IQ-TREE v2.2.0.3 [72] and RAxML-NG v1.1 [73] were used to estimate the species tree with 1000 bootstrap repetitions. MCMCtree [74] was used to infer the divergence time of the species tree. The gene counts derived from OrthoFinder [31] were used to calculate the gene expansion and contraction analysis by CAFE v5.0 [75].

### Linkage mapping of the inflorescence trait

An F_1_ population (‘Endless Summer’ × ‘Veitchii’) consisting of 341 individuals was developed and phenotyped for two consecutive years. The two inflorescence types in bigleaf hydrangea, lacecap and mophead, were recorded for all individuals as 0 and 1, respectively.

The leaf tissues of each F_1_ individual and the parents were collected for genomic DNA extraction and submitted to the Biotechnology Center of the University of Wisconsin-Madison for genotyping by sequencing. Library and barcoding methods were done according to Elshire *et al*. [76]. To allow for unbiased validation of the reference genome, the Tassel UNEAK pipeline (without reference) was used for SNP calling, as described in the previous study [32].

A linkage map was created using the cross-pollinated (CP) model in JoinMap v5.0 [77]. Three segregation types (lm × ll, nn × np, and hk × hk) were used to code SNPs based on the software instruction. The flower shape locus was coded as ‘lm x ll’ as it is heterozygous (lm) in the male parent ‘Veitchii’ and homozygous (ll) in the female parent ‘Endless Summer’. Markers experiencing segregation distortion were checked by the chi-square test. Identical F_1_ hybrids and severely distorted loci (*P* ≤ 0.001) were discarded for map construction. The LOD score was set at 5.0 to establish the linkage groups using regression mapping methods with default parameters. Map distances were calculated using the Kosambi mapping function in centiMorgan (cM). The consensus map was calculated using the same setting as described for each individual population and visualized in MapChart v2.2 [78, 79].

### Association mapping study of the inflorescence trait

A GWAS was performed using a collection of 82 hydrangea cultivars reported by Wu and Alexander [7]. The ‘Endless Summer’ primary haplotype assembly was used as the reference for SNP discovery using Tassel v5.0 [76]. GWAS was performed using two models as described previously [7]. Briefly, genomic DNA was isolated and digested by the ApeKI restriction enzyme for library preparation. The SNPs were called using the default parameters in Tassel GBS 5.0 [76]. SNPs with <10% missing data were retained for further data analyses. Both general linear and mixed linear models were used for the association study, with a determinate significance threshold of 0.001 for marker–trait associations.

### Identification of the candidate gene associated with the inflorescence trait

Four molecular markers (SSRs, 2 SNPs, and CAPS) associated with the inflorescence trait have been developed through previously published genetic and association mapping studies [7, 12, 20]. To identify the candidate gene associated with the inflorescence shape trait, the flanking marker TP279504 identified in the GWAS performed in this study above, along with the four molecular markers discovered previously, was mapped to both the ‘Veitchii’ and ‘Endless Summer’ genomes. Candidate genes in the two genomic regions flanked by those markers were identified with their annotated gene information. Homologous genes that are homozygous in ‘Endless Summer’ and heterozygous in ‘Veitchii’ were subjected to functional analyses using the NCBI database.

### Identification and validation of the novel candidate gene associated with the double-flower trait

Primers associated with the double-flower traits were designed based on published data [18]. Four double-flower cultivars (‘Fuji waterfall’, ‘Double Delights’, ‘Forever and Ever’, and ‘Doublicious’), along with three single-flower cultivars (‘Uzu Azisai’, ‘Veitchii’, and ‘Endless Summer’), were used to amplify the regions associated with tested primers. The PCR was performed with the regions associated with specific primers. The PCR fragments were isolated, purified, and processed using BigDye® Terminator v3.1 Cycle Sequencing Kit (Thermo Fisher Scientific, USA). The raw sequencing data were obtained from the capillary sequencer 3730XL DNA Analyzer (Applied Biosystems, MA, USA) and analyzed with the DNASTAR SeqMan module (DNASTAR, Inc.) (aligned sequence shown in Table S7). The sequences were aligned to both genomes using BLAST to identify the candidate genes associated with the double-flower trait.

## Acknowledgments

The research presented in this article was supported in part by funds from the Floral and Nursery Research Initiative administered through the United States Department of Agriculture, Agricultural Research Service (USDA-ARS). The work was supported by USDA-ARS CRIS project numbers 6066-21310-005-00D and 8020-21000-072-000-D. The authors would like to thank Carrie Witcher for assistance with pollen collection and microscopy and Dr Jerry Jenkins from the HudsonAlpha Institute for Biotechnology for his technical advice on scaffolding. Benjamin Moore and Joseph Davis maintained the plants used in this experiment. This research used resources provided by the SCINet project of the USDA Agricultural Research Service, ARS project numbers 0500-00093-001-00-D and 5030-21000-069-00D. The mention of trade names of commercial products in the publication is solely for the purpose of providing specific information and does not imply recommendation or endorsement by the US Department of Agriculture.

## Author Contributions

X.W., L.W.A., and A.M.H.-K. developed the project. X.W. and A.M.H-K. performed all analyses. S.A.S., R.C.Y., X. F.L, and B.E.S. performed the sequencing. L.W.A. and T.A.R. generated plant populations and provided support. X.W., L.W.A., and A.M.H.-K. wrote the manuscript. All authors read and approved the final manuscript.

## Data Availability

All data supporting the findings in this study are available within this article and its additional files. The reference genome raw sequencing data, whole-genome sequencing data, RNA-seq data, and genome assembly data have been deposited at the NCBI Sequence Read Archive under BioProject number PRJNA675507 (https://www.ncbi.nlm.nih.gov/bioproject/PRJNA675507). Additional details can be found at https://github.com/USDA-ARS-GBRU/Hydrangea_Genomes.

## Conflict of Interests

The authors declare that they have no competing interests.

## Supplementary data

Supplementary data is available at Horticulture Research online.

## Supplementary Material

Web_Material_uhad217
