# Supplementary material for: Two haplotype-resolved genomes reveal important flower traits in bigleaf hydrangea (Hydrangea macrophylla) and insights into Asterid evolution
Source: Hortic Res. 2023 Nov 9;10(12):uhad217. doi: 10.1093/hr/uhad217 (PMC10734616; doi:10.1093/hr/uhad217)
Supplement: Web_Material_uhad217 [file web_material_uhad217.zip › HydrangeaGenome_SuppFigures_v5.pdf]

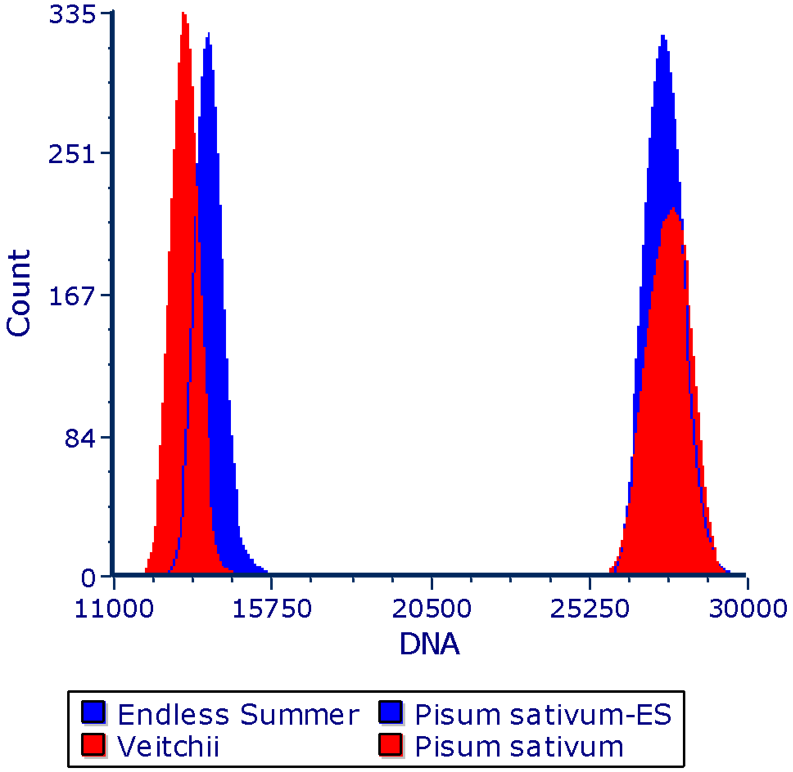

Figure S1. Flow cytometry of 'Veitchii' and 'Endless Summer' cultivars (*Pisum sativum* as internal standard).

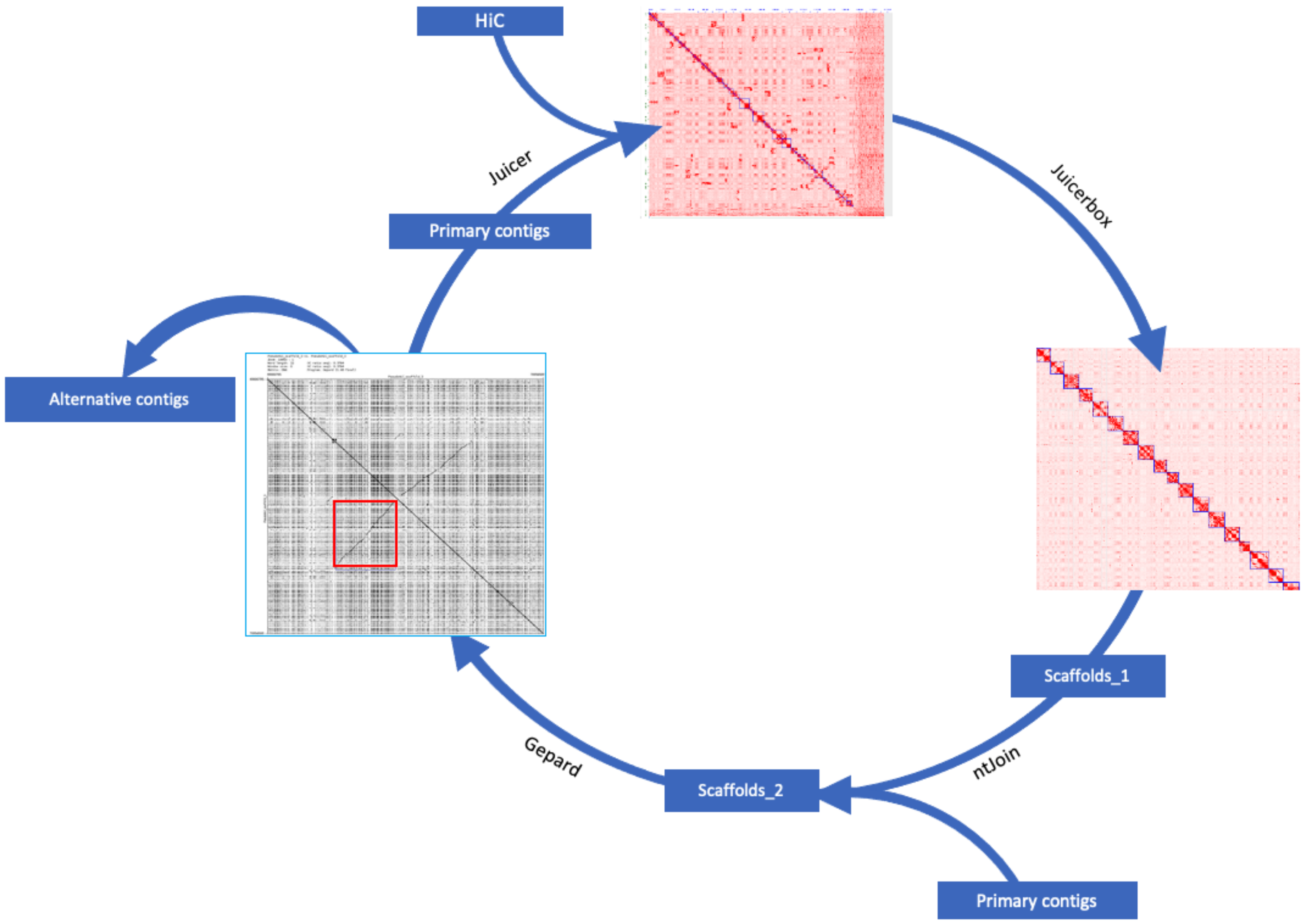

Figure S2. Purging approach used for 'Veitchii' genome assembly. The red square on the left dotplot indicate the existence of potential haplotigs to be purged.

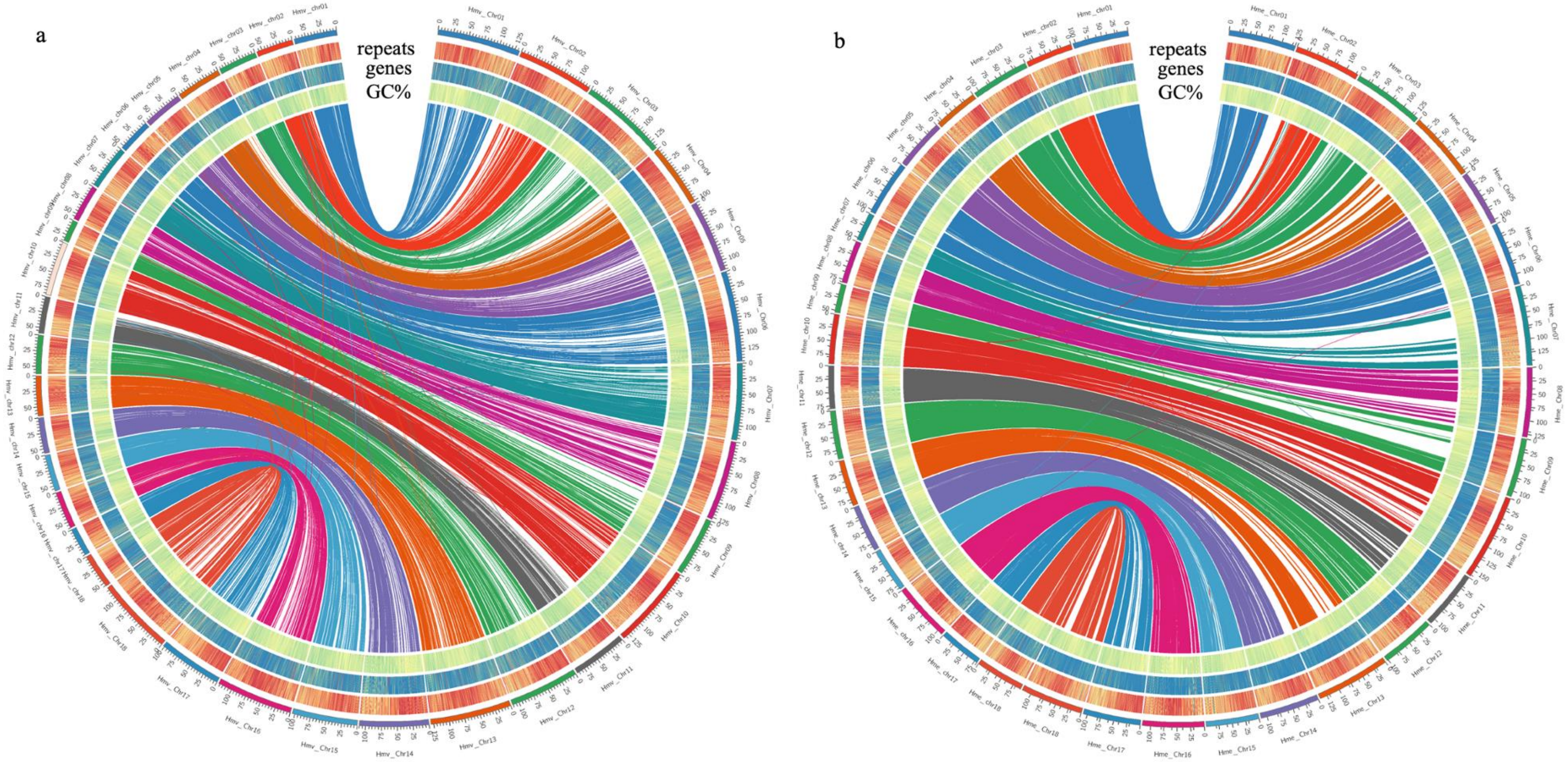

Figure S3. Genome landscapes of *Hydrangea macrophylla*. a) synteny of the two 'Veitchii' genome haplotypes; b) synteny of two 'Endless Summer' genome haplotypes.

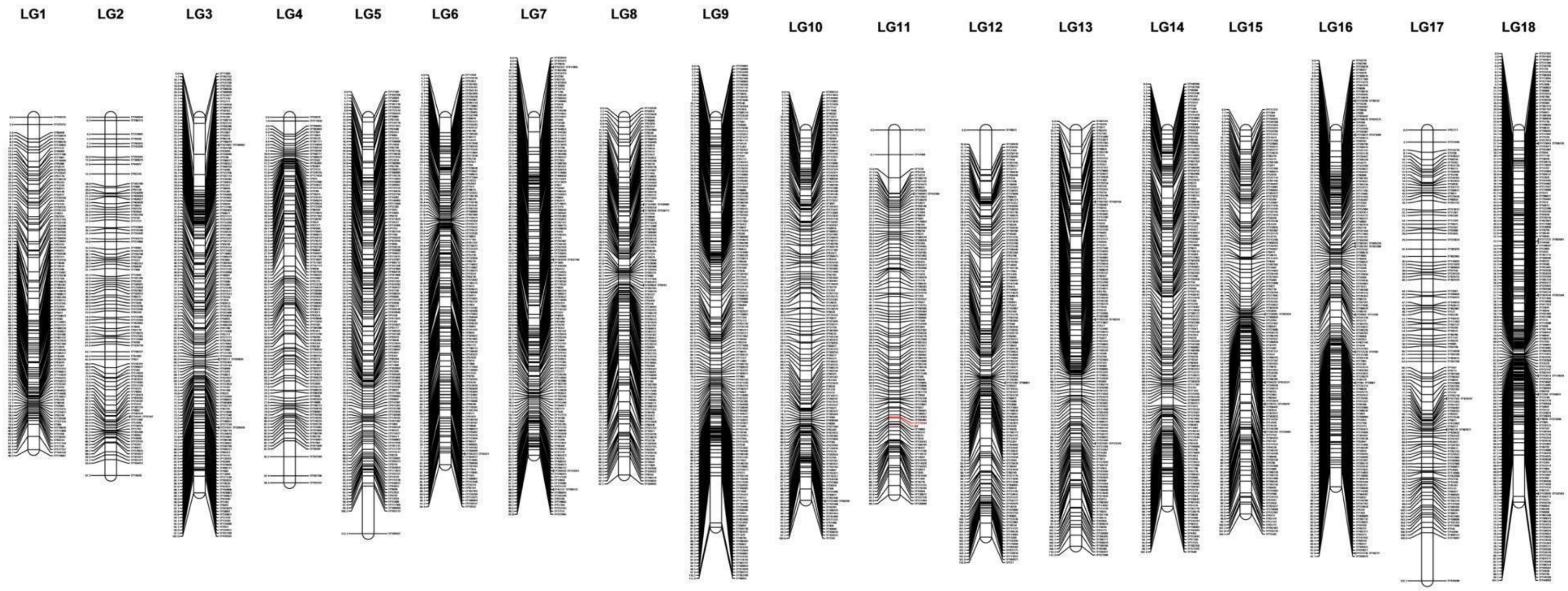

Figure S3. Linkage map derived from F_1 population ('Veitchii' x 'Endless Summer').

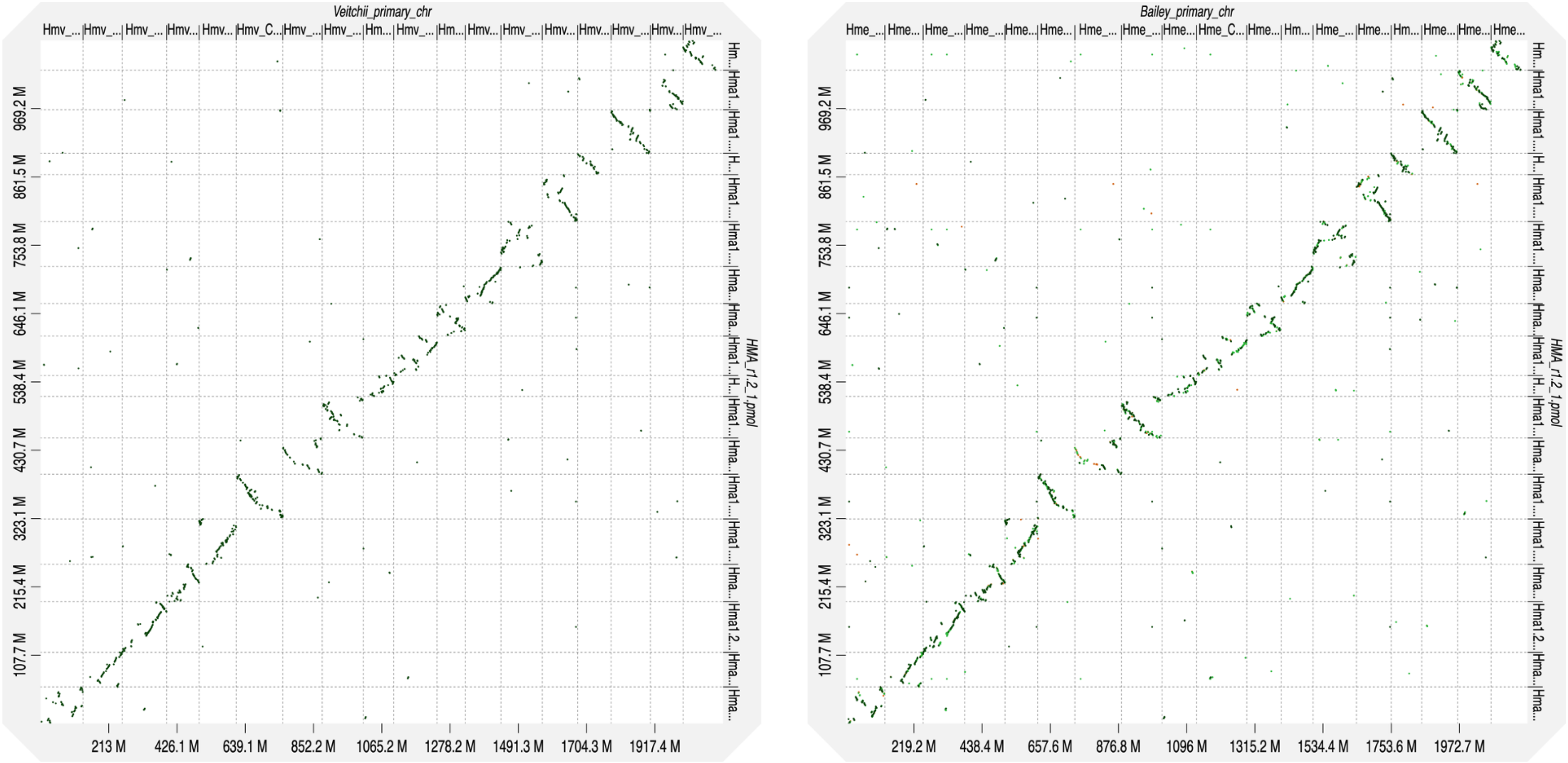

Figure S4. Alignments of assembled genomes ('Veitchii', left and 'Endless Summer', right) with available hydrangea genome (Nashima et al., 2014).
