# Supplementary material for: Two haplotype-resolved genomes reveal important flower traits in bigleaf hydrangea (Hydrangea macrophylla) and insights into Asterid evolution
Source: Hortic Res. 2023 Nov 9;10(12):uhad217. doi: 10.1093/hr/uhad217 (PMC10734616; doi:10.1093/hr/uhad217)
Supplement: Web_Material_uhad217 [file web_material_uhad217.zip › Hydrangea_SuppTables_v4.pdf]

Table S1. Genetic landscape of the two hydrangea genomes.

	Veitchii primary		Endless Summer primary	
	# of elements	length occupied (bp)	# of elements	length occupied (bp)
Retroelements	573,011	926,315,223	836,981	1,167,112,032
SINEs	0	0	14,951	2,340,112
Penelope	1,960	605,922	0	0
LINEs	33,199	25,320,660	62,736	28,191,406
L2/CR1/Rex	0	0	10,013	2,178,230
RTE/Bov-B	3,005	2,257,675	5,625	4,411,026
L1/CIN4	28,234	22,457,063	17,677	16,644,830
LTR elements	539,812	900,994,563	759,294	1,136,580,514
BEL/Pao	242	60,847	0	0
Ty1/Copia	136,186	180,792,912	188,524	231,282,411
Gypsy/DIRS1	388,367	709,179,494	539,158	889,243,828
Retroviral	5,282	2,100,841	4,079	3,606,141
DNA transposons	58,604	30,320,776	378,908	105,261,693
hobo-Activator	19,596	8,430,459	108,178	28,997,694
Tc1-IS630-Pogo	0	0	22,190	6,229,758
Tourist/Harbinger	4,606	2,060,775	47,273	8,683,794
Rolling-circles	10,005	5,173,995	12,726	13,660,900
Unclassified	2,237,862	763,839,461	986,238	324,957,889
Total interspersed repeats		1,720,475,460		1,597,331,614
Small RNA	751	563,084	23,594	13,267,330
Satellites	613	207,496	5,993	4,237,808
Simple repeats	567,519	22,806,015	657,507	67,568,687
Low complexity	62,341	3,086,012	69,678	3,570,814

Table S2. Number of predicted and annotated genes in two hydrangea genomes

	Total Predicted genes				Fuctionally Annotated genes			
Genome	Veitchii		Endless Summer		Veitchii		Endless Summer	
Haplotype	primary	secondary	primary	secondary	primary	secondary	primary	secondary
Number of genes	59,474	32,419	71,773	51,392	42,194	23,846	50,326	36,495
Average overall gene size (bp)	4837.07	4831.77	4200.94	4405.05	5941.57	5879.45	5187.25	5385.39
Median overall gene size (bp)	2,151	2,170	1,824	1,993	3,024	3,029	2,566	2,725
Average overall CDS size (bp)	959.53	971.01	967.14	992.71	1121.75	1129.86	1143.41	1163.70
Median overall CDS size (bp)	708	714	693	729	897	912	909	927
Average overall exon size (bp)	244.82	240.49	264.44	262.46	242.00	239.55	265.43	262.85
Median overall exon size (bp)	139	135	148	147	135	132	143	143

Table S3. Comparative genomics statistics between 'Endless Summer' and 'Veitchii'

	Endless Summer	Veitchii	Overall
Number of genes	123,165	91,893	215,058
Number of genes in orthogroups	109,317	83,980	193,297
Number of unassigned genes	13,848	7,913	21,761
% of genes in orthogroups	88.80	91.40	89.90
% of unassigned genes	11.20	8.60	10.10
Number of orthogroups containing cultivar	45,372	43,050	47,649
% of orthogroups containing cultivar	95.20	90.30	85.57
Number of cultivar-specific orthogroups	4,599	2,277	6,876
Number of genes in cultivar-specific orthogroups	23,283	7,950	31,233
% of genes in cultivar-specific orthogroups	18.90	8.70	14.50
Number of single-copy orthogroups	20,173	19,452	10,716

Table S4. Unique homologous genes within Vetichii and Endless_Summer genomes

Query Sequence	Query Sequence	Veitchii genome	Haplotype	Subject Sequence	Percent Identity	Description	Species	EggNOG Seed Ortholog	EggNOG Description	EggNOG GO Biological	EggNOG GO Cellular	EggNOG GO Molecular
Endless_Summer_g1	Endless_Summer_g1	Endless Summer genom	Primary assembly	tr|A0A2N9FZE3|A0A2N9FZE3_FAGSY	39.3	tr|A0A2N9FZE3|A0A2N9FZE3_FAGSY SWIM-type domain-containing protein OS=Fagus sylvatica OX=28930 GN=FSB_LO	fagus sylvatica	39947.LOC_Os04g33010.1	[illegible]	[illegible]	[illegible]	[illegible]
Endless_Summer_g10042	Endless_Summer_g10042	Endless Summer genom	Primary assembly	tr|A0A498JXY9|A0A498JXY9_MALDO	43.7	tr|A0A498JXY9|A0A498JXY9_MALDO Uncharacterized protein OS=Malus domestica OX=3750 GN=DVH24_030566 PE=4 S	malus domestica	3694.POPTR_0014s17180.1	[illegible]	[illegible]	[illegible]	[illegible]
Endless_Summer_g10075	Endless_Summer_g10075	Endless Summer genom	Primary assembly	XP_034212983.1	62.6	XP_034212983.1 myb-like protein F [Prunus dulcis]	prunus dulcis	39947.LOC_Os12g05520.1		[illegible]	[illegible]	[illegible]
Endless_Summer_g10094	Endless_Summer_g10094	Endless Summer genom	Primary assembly	tr|A0A5B6YHB8|A0A5B6YHB8_DAVIN	44.6	tr|A0A5B6YHB8|A0A5B6YHB8_DAVIN Putative disease resistance protein RGA3 OS=Davidia involucrata OX=16924 GN=Di	davidia involucrata	29760.VIT_00s0144g00120.t01	[illegible]	[illegible]	[illegible]	[illegible]
Endless_Summer_g10145	Endless_Summer_g10145	Endless Summer genom	Primary assembly	XP_021680462.1	48.5	XP_021680462.1 putative disease resistance protein RGA3 [Hevea brasiliensis]	hevea brasiliensis	29760.VIT_00s0144g00270.t01	[illegible]	[illegible]	[illegible]	[illegible]
Endless_Summer_g10182	Endless_Summer_g10182	Endless Summer genom	Primary assembly	tr|A0A7J7MW77|A0A7J7MW77_9MAGN	40.9	tr|A0A7J7MW77|A0A7J7MW77_9MAGN DDE Tnp4 domain-containing protein OS=Kingdonia uniflora OX=39325 GN=GIB	kingdonia uniflora	4577.GRMZM2G053744_P01	[illegible]	[illegible]	[illegible]	[illegible]
Endless_Summer_g10271	Endless_Summer_g10271	Endless Summer genom	Primary assembly	tr|A0A6S7PEV9|A0A6S7PEV9_LACSI	73.1	tr|A0A6S7PEV9|A0A6S7PEV9_LACSI Hydrolase_4 domain-containing protein OS=Lactuca saligna OX=75948 GN=LSAL_LC	lactuca saligna	3847.GLYMA20G39110.1	[illegible]	[illegible]	[illegible]	[illegible]
Endless_Summer_g10324	Endless_Summer_g10324	Endless Summer genom	Primary assembly	tr|A0A2R6QV75|A0A2R6QV75_ACTCC	37.9	tr|A0A2R6QV75|A0A2R6QV75_ACTCC E3 ubiquitin-protein ligase MID2 OS=Actinidia chinensis var. chinensis OX=159084	actinidia chinensis var. chi	3694.POPTR_0016s05820.1	[illegible]	[illegible]	[illegible]	[illegible]
Endless_Summer_g10450	Endless_Summer_g10450	Endless Summer genom	Primary assembly	tr|A0A6P6T122|A0A6P6T122_COFAR	26	tr|A0A6P6T122|A0A6P6T122_COFAR ATP-dependent DNA helicase OS=Coffea arabica OX=13443 GN=LOC113696833 PE=	coffea arabica	4558.Sb05g006165.1	[illegible]	[illegible]	[illegible]	[illegible]
Endless_Summer_g10460	Endless_Summer_g10460	Endless Summer genom	Primary assembly	XP_023916366.1	35.8	XP_023916366.1 uncharacterized protein LOC112027956 [Quercus suber]	quercus suber	39947.LOC_Os05g33490.1		[illegible]	[illegible]	[illegible]
Endless_Summer_g10582	Endless_Summer_g10582	Endless Summer genom	Primary assembly	tr|A0A7J7FX66|A0A7J7FX66_CAMSI	70.8	tr|A0A7J7FX66|A0A7J7FX66_CAMSI VWFA domain-containing protein OS=Camellia sinensis OX=4442 GN=HYC85_0291	camellia sinensis	15368.BRADI3G35660.1	[illegible]	[illegible]	[illegible]	[illegible]
Endless_Summer_g10595	Endless_Summer_g10595	Endless Summer genom	Primary assembly	tr|A0A5B6YHC2|A0A5B6YHC2_DAVIN	51.5	tr|A0A5B6YHC2|A0A5B6YHC2_DAVIN Putative disease resistance protein RGA3 OS=Davidia involucrata OX=16924 GN=Di	davidia involucrata	29760.VIT_08s0007g08420.t01	[illegible]	[illegible]	[illegible]	[illegible]
Endless_Summer_g10606	Endless_Summer_g10606	Endless Summer genom	Primary assembly	XP_034689141.1	43.9	XP_034689141.1 disease resistance protein RGA2-like [Vitis riparia]	vitis riparia	29760.VIT_08s0007g08420.t01	[illegible]	[illegible]	[illegible]	[illegible]
Endless_Summer_g10619	Endless_Summer_g10619	Endless Summer genom	Primary assembly	XP_034672573.1	51.4	XP_034672573.1 putative disease resistance protein RGA1 [Vitis riparia]	vitis riparia	29760.VIT_00s0144g00270.t01	[illegible]	[illegible]	[illegible]	[illegible]
Endless_Summer_g10644	Endless_Summer_g10644	Endless Summer genom	Primary assembly	XP_021649945.1	54.3	XP_021649945.1 disease resistance protein RGA2-like [Hevea brasiliensis]	hevea brasiliensis	29760.VIT_00s0144g00270.t01	[illegible]	[illegible]	[illegible]	[illegible]
Endless_Summer_g10651	Endless_Summer_g10651	Endless Summer genom	Primary assembly	tr|A0A5A7TIZ8|A0A5A7TIZ8_CUCME	56.4	tr|A0A5A7TIZ8|A0A5A7TIZ8_CUCME Retrovirus-related Pol polyprotein from transposon TNT 1-94 OS=Cucumis melo var.	cucumis melo var. makuwa	4081.Solyc04g011970.1.1	[illegible]	[illegible]	[illegible]	[illegible]
Endless_Summer_g10688	Endless_Summer_g10688	Endless Summer genom	Primary assembly	XP_023913134.1	39.8	XP_023913134.1 uncharacterized protein LOC112024732 [Quercus suber]	quercus suber	39947.LOC_Os10g37940.1	[illegible]	[illegible]	[illegible]	[illegible]
Endless_Summer_g10703	Endless_Summer_g10703	Endless Summer genom	Primary assembly	XP_039689926.1	64.8	XP_039689926.1 uncharacterized protein LOC120580313 isoform X2 [Medicago truncatula]	medicago truncatula	3694.POPTR_0011s05940.1	[illegible]	[illegible]	[illegible]	[illegible]
Endless_Summer_g10709	Endless_Summer_g10709	Endless Summer genom	Primary assembly	XP_028124712.1	36.9	XP_028124712.1 zinc finger BED domain-containing protein RICESLEEPER 2-like [Camellia sinensis]	camellia sinensis	3694.POPTR_0001s31070.1	[illegible]	[illegible]	[illegible]	[illegible]
Endless_Summer_g11190	Endless_Summer_g11190	Endless Summer genom	Primary assembly	XP_038983707.1	60.4	XP_038983707.1 uncharacterized protein LOC120111218 [Phoenix dactylifera]	phoenix dactylifera	39947.LOC_Os02g12320.1		[illegible]	[illegible]	[illegible]
Endless_Summer_g11567	Endless_Summer_g11567	Endless Summer genom	Primary assembly	XP_011021479.1	60	XP_011021479.1 PREDICTED: uncharacterized protein LOC105123549 [Populus euphratica]	populus euphratica	3694.POPTR_0019s05060.1	[illegible]	[illegible]	[illegible]	[illegible]
Endless_Summer_g11641	Endless_Summer_g11641	Endless Summer genom	Primary assembly	tr|A0A6P5R744|A0A6P5R744_PRUAV	28.6	tr|A0A6P5R744|A0A6P5R744_PRUAV uncharacterized protein LOC110744993 OS=Prunus avium OX=42229 GN=LOC11074	prunus avium	3694.POPTR_0004s23230.1	NA	[illegible]	[illegible]	[illegible]
Endless_Summer_g11742	Endless_Summer_g11742	Endless Summer genom	Primary assembly	XP_011075145.1	86.2	XP_011075145.1 delta(12)-fatty-acid desaturase FAD2 [Sesamum indicum]	sesamum indicum	4113.PGSC0003DMT400014051	[illegible]	[illegible]	[illegible]	[illegible]
Endless_Summer_g11865	Endless_Summer_g11865	Endless Summer genom	Primary assembly	tr|A0A5B6ZU10|A0A5B6ZU10_DAVIN	24.3	tr|A0A5B6ZU10|A0A5B6ZU10_DAVIN Putative LRR receptor-like serine/threonine-protein kinase ERECTA OS=Davidia invo	davidia involucrata	4081.Solyc01g008750.1.1	[illegible]	[illegible]	[illegible]	[illegible]
Endless_Summer_g1193	Endless_Summer_g1193	Endless Summer genom	Primary assembly	tr|A0A7J0D768|A0A7J0D768_9ERIC	26.4	tr|A0A7J0D768|A0A7J0D768_9ERIC Uncharacterized protein OS=Actinidia rufa OX=165716 GN=Acr_00g0003440 PE=4 SV=	actinidia rufa					
Endless_Summer_g11978	Endless_Summer_g11978	Endless Summer genom	Primary assembly	tr|A0A2N9HZW5|A0A2N9HZW5_FAGSY	60.3	tr|A0A2N9HZW5|A0A2N9HZW5_FAGSY Integrase catalytic domain-containing protein OS=Fagus sylvatica OX=28930 GN=F	fagus sylvatica	39947.LOC_Os02g43800.1	[illegible]	[illegible]	[illegible]	[illegible]
Endless_Summer_g12146	Endless_Summer_g12146	Endless Summer genom	Primary assembly	XP_009615891.1	71.1	XP_009615891.1 EEF1A lysine methyltransferase 1 [Nicotiana tomentosiformis]	nicotiana tomentosiformis	4113.PGSC0003DMT400072285	[illegible]	[illegible]	[illegible]	[illegible]
Endless_Summer_g12344	Endless_Summer_g12344	Endless Summer genom	Primary assembly	XP_028112025.1	64.3	XP_028112025.1 receptor-like protein EIX2 [Camellia sinensis]	camellia sinensis	29760.VIT_14s0171g00050.t01	[illegible]	[illegible]	[illegible]	[illegible]
Endless_Summer_g12392	Endless_Summer_g12392	Endless Summer genom	Primary assembly	tr|A0A834G4P3|A0A834G4P3_RHOSS	65	tr|A0A834G4P3|A0A834G4P3_RHOSS Uncharacterized protein OS=Rhododendron simsii OX=118357 GN=RHSIM_Rhsim12	rhododendron simsii	29760.VIT_01s0010g03700.t01	[illegible]	[illegible]	[illegible]	[illegible]
Endless_Summer_g12408	Endless_Summer_g12408	Endless Summer genom	Primary assembly	XP_028061728.1	38.2	XP_028061728.1 uncharacterized protein LOC114265167 [Camellia sinensis]	camellia sinensis	29760.VIT_01s0010g03790.t01	[illegible]	[illegible]	[illegible]	[illegible]
Endless_Summer_g1243	Endless_Summer_g1243	Endless Summer genom	Primary assembly	XP_030924541.1	33.7	XP_030924541.1 uncharacterized protein LOC115951488 [Quercus lobata]	quercus lobata	4641.GSMUA_Achr6P01610_001	[illegible]	[illegible]	[illegible]	[illegible]
Endless_Summer_g1248	Endless_Summer_g1248	Endless Summer genom	Primary assembly	tr|A2EE01|A2EE01_TRIVA	33.5	tr|A2EE01|A2EE01_TRIVA HORMA domain containing protein OS=Trichomonas vaginalis OX=5722 GN=TVAG_230730 PE	trichomonas vaginalis	15368.BRADI4G34650.1	[illegible]	[illegible]	[illegible]	[illegible]
Endless_Summer_g12508	Endless_Summer_g12508	Endless Summer genom	Primary assembly	tr|A0A5B7BT39|A0A5B7BT39_DAVIN	55.2	tr|A0A5B7BT39|A0A5B7BT39_DAVIN Uncharacterized protein OS=Davidia involucrata OX=16924 GN=Din_041089 PE=4 S	davidia involucrata	3694.POPTR_0010s06790.1	NA	[illegible]	[illegible]	[illegible]
Endless_Summer_g12559	Endless_Summer_g12559	Endless Summer genom	Primary assembly	XP_028127065.1	60.1	XP_028127065.1 receptor-like protein EIX2 [Camellia sinensis]	camellia sinensis	29760.VIT_14s0171g00050.t01	[illegible]	[illegible]	[illegible]	[illegible]
Endless_Summer_g12605	Endless_Summer_g12605	Endless Summer genom	Primary assembly	XP_010044171.2	62.4	XP_010044171.2 protein ALP1-like [Eucalyptus grandis]	eucalyptus grandis	4081.Solyc09g064290.1.1	[illegible]	[illegible]	[illegible]	[illegible]
Endless_Summer_g1284	Endless_Summer_g1284	Endless Summer genom	Primary assembly	XP_034676300.1	47.7	XP_034676300.1 tRNA nucleotidyltransferase cca2 isoform X2 [Vitis riparia]	vitis riparia	29760.VIT_18s0075g00880.t01	[illegible]	[illegible]	[illegible]	[illegible]
Endless_Summer_g12918	Endless_Summer_g12918	Endless Summer genom	Primary assembly	XP_034679115.1	75.6	XP_034679115.1 potassium transporter 5 [Vitis riparia]	vitis riparia	29760.VIT_01s0011g03020.t01	[illegible]	[illegible]	[illegible]	[illegible]
Endless_Summer_g13038	Endless_Summer_g13038	Endless Summer genom	Primary assembly	tr|A0A7J0DLA4|A0A7J0DLA4_9ERIC	60	tr|A0A7J0DLA4|A0A7J0DLA4_9ERIC Uncharacterized protein OS=Actinidia rufa OX=165716 GN=Acr_00g0047520 PE=4 SV	actinidia rufa					
Endless_Summer_g13066	Endless_Summer_g13066	Endless Summer genom	Primary assembly	tr|G3MJR8|G3MJR8_AMBMU	40	tr|G3MJR8|G3MJR8_AMBMU Protein kinase domain-containing protein (Fragment) OS=Amblyomma maculatum OX=34609	amblyomma maculatum	29760.VIT_05s0020g03300.t01	[illegible]	[illegible]	[illegible]	[illegible]
Endless_Summer_g13126	Endless_Summer_g13126	Endless Summer genom	Primary assembly	XP_030974848.1	80.2	XP_030974848.1 DExH-box ATP-dependent RNA helicase DExH16, mitochondrial isoform X2 [Quercus lobata]	quercus lobata	4081.Solyc09g082380.2.1	[illegible]	[illegible]	[illegible]	[illegible]
Endless_Summer_g13290	Endless_Summer_g13290	Endless Summer genom	Primary assembly	XP_028121132.1	27.9	XP_028121132.1 uncharacterized protein LOC114318438 [Camellia sinensis]	camellia sinensis					
Endless_Summer_g1346	Endless_Summer_g1346	Endless Summer genom	Primary assembly	XP_022889492.1	34.8	XP_022889492.1 uncharacterized protein LOC111405032 [Olea europaea var. sylvestris]	olea europaea var. sylvestri	4577.GRMZM5G827702_P01	[illegible]	[illegible]	[illegible]	[illegible]
Endless_Summer_g13668	Endless_Summer_g13668	Endless Summer genom	Primary assembly	tr|A0A5B7BZ64|A0A5B7BZ64_DAVIN	35.2	tr|A0A5B7BZ64|A0A5B7BZ64_DAVIN Retrotran_gag_3 domain-containing protein OS=Davidia involucrata OX=16924 GN=D	davidia involucrata	39947.LOC_Os10g04640.1	[illegible]	[illegible]	[illegible]	[illegible]
Endless_Summer_g14411	Endless_Summer_g14411	Endless Summer genom	Primary assembly	XP_034686273.1	55	XP_034686273.1 protein TIME FOR COFFEE isoform X1 [Vitis riparia]	vitis riparia	29760.VIT_05s0020g03150.t01	Protein TIME FOR	[illegible]	[illegible]	[illegible]
Endless_Summer_g14534	Endless_Summer_g14534	Endless Summer genom	Primary assembly	tr|A0A5J5BK47|A0A5J5BK47_9ASTE	76.6	tr|A0A5J5BK47|A0A5J5BK47_9ASTE Uncharacterized protein OS=Nyssa sinensis OX=561372 GN=F0562_022693 PE=4 SV=	nyssa sinensis	3694.POPTR_0010s25780.1	[illegible]	[illegible]	[illegible]	[illegible]
Endless_Summer_g14650	Endless_Summer_g14650	Endless Summer genom	Primary assembly	XP_009587100.1	84.5	XP_009587100.1 mannosyl-oligosaccharide 1,2-alpha-mannosidase MNS1-like isoform X1 [Nicotiana tomentosiformis]	nicotiana tomentosiformis	4081.Solyc03g123900.2.1	[illegible]	[illegible]	[illegible]	[illegible]
Endless_Summer_g14675	Endless_Summer_g14675	Endless Summer genom	Primary assembly	tr|A0A5B7BTY4|A0A5B7BTY4_DAVIN	70.9	tr|A0A5B7BTY4|A0A5B7BTY4_DAVIN Glutamate receptor OS=Davidia involucrata OX=16924 GN=Din_039994 PE=3 SV=	davidia involucrata	4081.Solyc06g063190.2.1	[illegible]	[illegible]	[illegible]	[illegible]
Endless_Summer_g14893	Endless_Summer_g14893	Endless Summer genom	Primary assembly	XP_019263696.1	37.2	XP_019263696.1 PREDICTED: uncharacterized protein LOC109241405 [Nicotiana attenuata]	nicotiana attenuata	4113.PGSC0003DMT400091433	[illegible]	[illegible]	[illegible]	[illegible]
Endless_Summer_g14955	Endless_Summer_g14955	Endless Summer genom	Primary assembly	tr|A0A5B7CAT6|A0A5B7CAT6_DAVIN	85	tr|A0A5B7CAT6|A0A5B7CAT6_DAVIN Lactamase_B domain-containing protein OS=Davidia involucrata OX=16924 GN=Di	davidia involucrata	29760.VIT_09s0002g02310.t01	[illegible]	[illegible]	[illegible]	[illegible]
Endless_Summer_g14968	Endless_Summer_g14968	Endless Summer genom	Primary assembly	tr|A0A6A4KPF9|A0A6A4KPF9_9ERIC	30.1	tr|A0A6A4KPF9|A0A6A4KPF9_9ERIC Uncharacterized protein (Fragment) OS=Rhododendron williamsianum OX=262921 GN	rhododendron williamsianum	29760.VIT_07s0031g02450.t01	NA	[illegible]	[illegible]	[illegible]
Endless_Summer_g15106	Endless_Summer_g15106	Endless Summer genom	Primary assembly	XP_021294642.1	46.6	XP_021294642.1 probable LRR receptor-like serine/threonine-protein kinase At1g51860 [Herrania umbratica]	herrania umbratica	4081.Solyc03g121230.2.1	[illegible]	[illegible]	[illegible]	[illegible]
Endless_Summer_g15194	Endless_Summer_g15194	Endless Summer genom	Primary assembly	tr|A0A2R6PTU1|A0A2R6PTU1_ACTCC	59.2	tr|A0A2R6PTU1|A0A2R6PTU1_ACTCC Leucine-rich repeat receptor-like protein kinase OS=Actinidia chinensis var. chinensis	actinidia chinensis var. chi	29760.VIT_09s0002g03030.t01	[illegible]	[illegible]	[illegible]	[illegible]
Endless_Summer_g15247	Endless_Summer_g15247	Endless Summer genom	Primary assembly	XP_038896231.1	44.2	XP_038896231.1 uncharacterized protein LOC120084509 [Benincasa hispida]	benincasa hispida	3711.Bra031278.1-P	NA	[illegible]	[illegible]	[illegible]
Endless_Summer_g1525	Endless_Summer_g1525	Endless Summer genom	Primary assembly	XP_034689383.1	79.7	XP_034689383.1 2-alkenal reductase (NADP(+)-dependent) [Vitis riparia]	vitis riparia	29760.VIT_00s0220g00130.t01	[illegible]	[illegible]	[illegible]	[illegible]
Endless_Summer_g15305	Endless_Summer_g15305	Endless Summer genom	Primary assembly	XP_024030944.1	41.4	XP_024030944.1 zinc finger BED domain-containing protein RICESLEEPER 2-like [Morus notabilis]	morus notabilis	3847.GLYMA14G13178.1	[illegible]	[illegible]	[illegible]	[illegible]
Endless_Summer_g15361	Endless_Summer_g15361	Endless Summer genom	Primary assembly	XP_034695285.1	74.2	XP_034695285.1 nuclear poly(A) polymerase 4-like isoform X1 [Vitis riparia]	vitis riparia	29760.VIT_09s0002g03380.t01	[illegible]	[illegible]	[illegible]	[illegible]
Endless_Summer_g15370	Endless_Summer_g15370	Endless Summer genom	Primary assembly	XP_027168708.1	79.9	XP_027168708.1 clavaminate synthase-like protein At3g21360 [Coffea eugenioides]	coffea eugenioides	3694.POPTR_0001s19100.1	[illegible]	[illegible]	[illegible]	[illegible]
Endless_Summer_g15724	Endless_Summer_g15724	Endless Summer genom	Primary assembly	tr|A0A834FY36|A0A834FY36_RHOSS	52.2	tr|A0A834FY36|A0A834FY36_RHOSS SWIM-type domain-containing protein OS=Rhododendron simsii OX=118357 GN=RH	rhododendron simsii	39947.LOC_Os07g40760.1	[illegible]	[illegible]	[illegible]	[illegible]
Endless_Summer_g15776	Endless_Summer_g15776	Endless Summer genom	Primary assembly	XP_028101347.1	54.1	XP_028101347.1 F-box/kelch-repeat protein At3g23880-like [Camellia sinensis]	camellia sinensis	3694.POPTR_0008s20430.1	[illegible]	[illegible]	[illegible]	[illegible]
Endless_Summer_g15915	Endless_Summer_g15915	Endless Summer genom	Primary assembly	XP_021646191.1	48.5	XP_021646191.1 NAC domain-containing protein 96-like [Hevea brasiliensis]	hevea brasiliensis	4081.Solyc11g065540.1.1	[illegible]	[illegible]	[illegible]	[illegible]
Endless_Summer_g16106	Endless_Summer_g16106	Endless Summer genom	Primary assembly	XP_027117180.1	46	XP_027117180.1 probable disease resistance protein At1g58602 [Coffea arabica]	coffea arabica	3694.POPTR_0018s13530.1	[illegible]	[illegible]	[illegible]	[illegible]
Endless_Summer_g16186	Endless_Summer_g16186	Endless Summer genom	Primary assembly	XP_038701961.1	41.1	XP_038701961.1 uncharacterized protein LOC119998671 [Tripterygium wilfordii]	tripterygium wilfordii					
Endless_Summer_g16245	Endless_Summer_g16245	Endless Summer genom	Primary assembly	tr|A0A5A7V6N0|A0A5A7V6N0_CUCME	58.4	tr|A0A5A7V6N0|A0A5A7V6N0_CUCME Gag/pol protein OS=Cucumis melo var. makuwa OX=1194695 GN=E6C27_scaffold	cucumis melo var. makuwa	39947.LOC_Os03g35560.1		[illegible]	[illegible]	[illegible]
Endless_Summer_g16336	Endless_Summer_g16336	Endless Summer genom	Primary assembly	XP_008234762.1	52.3	XP_008234762.1 PREDICTED: uncharacterized protein At4g00950 [Prunus mume]	prunus mume	3694.POPTR_0015s01420.1	NA	[illegible]	[illegible]	[illegible]
Endless_Summer_g1638	Endless_Summer_g1638	Endless Summer genom	Primary assembly	tr|A0A4S4D5A1|A0A4S4D5A1_CAMSI	38.1	tr|A0A4S4D5A1|A0A4S4D5A1_CAMSI Uncharacterized protein OS=Camellia sinensis var. sinensis OX=542762 GN=TEA_02	camellia sinensis var. sinen	29760.VIT_00s0208g00040.t01	[illegible]	[illegible]	[illegible]	[illegible]
Endless_Summer_g1642	Endless_Summer_g1642	Endless Summer genom	Primary assembly	XP_028099939.1	31.5	XP_028099939.1 putative F-box protein At3g52320 isoform X1 [Camellia sinensis]	camellia sinensis	29760.VIT_18s0041g01840.t01	[illegible]	[illegible]	[illegible]	[illegible]
Endless_Summer_g16438	Endless_Summer_g16438	Endless Summer genom	Primary assembly	tr|A0A5B7BUZ6|A0A5B7BUZ6_DAVIN	87.4	tr|A0A5B7BUZ6|A0A5B7BUZ6_DAVIN DYW_deaminase domain-containing protein (Fragment) OS=Davidia involucrata OX=	davidia involucrata	29760.VIT_18s0001g13940.t01	[illegible]	[illegible]	[illegible]	[illegible]
Endless_Summer_g16455	Endless_Summer_g16455	Endless Summer genom	Primary assembly	XP_031281093.1	77.6	XP_031281093.1 protein DETOXIFICATION 35-like [Pistacia vera]	pistacia vera	4081.Solyc06g036130.2.1	[illegible]	[illegible]	[illegible]	[illegible]
Endless_Summer_g16459	Endless_Summer_g16459	Endless Summer genom	Primary assembly	tr|A0A5B7CD89|A0A5B7CD89_DAVIN	34.4	tr|A0A5B7CD89|A0A5B7CD89_DAVIN HMG box domain-containing protein OS=Davidia involucrata OX=16924 GN=Din_04	davidia involucrata	4113.PGSC0003DMT400043037	[illegible]	[illegible]	[illegible]	[illegible]
Endless_Summer_g16482	Endless_Summer_g16482	Endless Summer genom	Primary assembly	tr|A0A833X666|A0A833X666_JUGRE	44.2	tr|A0A833X666|A0A833X666_JUGRE CCHC-type domain-containing protein (Fragment) OS=Juglans regia OX=51240 GN=F	juglans regia	4113.PGSC0003DMT400025159		[illegible]	[illegible]	[illegible]
Endless_Summer_g16509	Endless_Summer_g16509	Endless Summer genom	Primary assembly	tr|A0A5J5BCI2|A0A5J5BCI2_9ASTE	52.8	tr|A0A5J5BCI2|A0A5J5BCI2_9ASTE Uncharacterized protein OS=Nyssa sinensis OX=561372 GN=F0562_024967 PE=4 SV=	nyssa sinensis	3694.POPTR_0554s00200.1	[illegible]	[illegible]	[illegible]	[illegible]
Endless_Summer_g16629	Endless_Summer_g16629	Endless Summer genom	Primary assembly	tr|A0A7J7KFM4|A0A7J7KFM4_BUGNE	37	tr|A0A7J7KFM4|A0A7J7KFM4_BUGNE ASCC3 OS=Bugula neritina OX=10212 GN=EB796_005026 PE=4 SV=1	bugula neritina	29760.VIT_17s0000g07230.t01	[illegible]	[illegible]	[illegible]	[illegible]
Endless_Summer_g16741	Endless_Summer_g16741	Endless Summer genom	Primary assembly	XP_023924679.1	28.2	XP_023924679.1 uncharacterized protein LOC112036085 [Quercus suber]	quercus suber	29760.VIT_09s0002g08460.t01	[illegible]	[illegible]	[illegible]	[illegible]
Endless_Summer_g16845	Endless_Summer_g16845	Endless Summer genom	Primary assembly	tr|A0A482RYN4|A0A482RYN4_9ARCH	30.7	tr|A0A482RYN4|A0A482RYN4_9ARCH Uncharacterized protein (Fragment) OS=archaeon OX=1906665 GN=EON64_09260 P	archaeon	29760.VIT_08s0058g01140.t01	NA	[illegible]	[illegible]	[illegible]
Endless_Summer_g16899	Endless_Summer_g16899	Endless Summer genom	Primary assembly	tr|A0A834GXX4|A0A834GXX4_RHOSS	36.2	tr|A0A834GXX4|A0A834GXX4_RHOSS Uncharacterized protein OS=Rhododendron simsii OX=118357 GN=RHSIM_Rhsim0	rhododendron simsii	39947.LOC_Os12g35940.1	[illegible]	[illegible]	[illegible]	[illegible]
Endless_Summer_g16923	Endless_Summer_g16923	Endless Summer genom	Primary assembly	tr|A0A4U1CUX5|A0A4U1CUX5_9BACI	51.2	tr|A0A4U1CUX5|A0A4U1CUX5_9BACI Uncharacterized protein OS=Robertmurraya kyonggiensis OX=1037680 GN=FA727_2	robertmurraya kyonggiensis	3702.AT4G38180.1	[illegible]	[illegible]	[illegible]	[illegible]
Endless_Summer_g17005	Endless_Summer_g17005	Endless Summer genom	Primary assembly	XP_028056138.1	69.4	XP_028056138.1 probable LRR receptor-like serine/threonine-protein kinase At3g47570 [Camellia sinensis]	camellia sinensis	3694.POPTR_0008s03400.1	[illegible]	[illegible]	[illegible]	[illegible]
Endless_Summer_g1709	Endless_Summer_g1709	Endless Summer genom	Primary assembly	XP_027066518.1	69.6	XP_027066518.1 dynamin-related protein 4C-like [Coffea arabica]	coffea arabica	4081.Solyc04g072030.1.1	[illegible]	[illegible]	[illegible]	[illegible]
Endless_Summer_g17263	Endless_Summer_g17263	Endless Summer genom	Primary assembly	tr|A0A5J5B172|A0A5J5B172_9ASTE	63.7	tr|A0A5J5B172|A0A5J5B172_9ASTE Protein kinase domain-containing protein OS=Nyssa sinensis OX=561372 GN=F0562_0	nyssa sinensis	4081.Solyc01g106160.1.1	[illegible]	[illegible]	[illegible]	[illegible]
Endless_Summer_g17289	Endless_Summer_g17289	Endless Summer genom	Primary assembly	tr|A0A7J0DWA5|A0A7J0DWA5_9ERIC	38.2	tr|A0A7J0DWA5|A0A7J0DWA5_9ERIC Uncharacterized protein OS=Actinidia rufa OX=165716 GN=Acr_00g0082040 PE=3 S	actinidia rufa	29760.VIT_07s0031g02450.t01	NA	[illegible]	[illegible]	[illegible]
Endless_Summer_g17313	Endless_Summer_g17313	Endless Summer genom	Primary assembly	XP_015159990.1	32.6	XP_015159990.1 PREDICTED: uncharacterized protein LOC107058609 [Solanum tuberosum]	solanum tuberosum	29760.VIT_00s0823g00010.t01	[illegible]	[illegible]	[illegible]	[illegible]
Endless_Summer_g17320	Endless_Summer_g17320	Endless Summer genom	Primary assembly	tr|A0A8B6Y297|A0A8B6Y297_HYDVU	44.8	tr|A0A8B6Y297|A0A8B6Y297_HYDVU uncharacterized protein LOC100200506 OS=Hydra vulgaris OX=6087 GN=LOC1002	hydra vulgaris	3847.GLYMA02G29116.1	[illegible]	[illegible]	[illegible]	[illegible]
Endless_Summer_g17364	Endless_Summer_g17364	Endless Summer genom	Primary assembly	XP_030938745.1	64	XP_030938745.1 receptor kinase-like protein Xa21 [Quercus lobata]	quercus lobata	3694.POPTR_0018s02450.1	[illegible]	[illegible]	[illegible]	[illegible]
Endless_Summer_g17415	Endless_Summer_g17415	Endless Summer genom	Primary assembly	XP_011084965.1	81.2	XP_011084965.1 probable sugar phosphate/phosphate translocator At3g11320 isoform X1 [Sesamum indicum]	sesamum indicum	29760.VIT_13s0019g03270.t01	[illegible]	[illegible]	[illegible]	[illegible]
Endless_Summer_g17453	Endless_Summer_g17453	Endless Summer genom	Primary assembly	tr|A0A2N9ET26|A0A2N9ET26_FAGSY	67.9	tr|A0A2N9ET26|A0A2N9ET26_FAGSY Reverse transcriptase Ty1/copia-type domain-containing protein OS=Fagus sylvatica O	fagus sylvatica	39947.LOC_Os03g35560.1		[illegible]	[illegible]	[illegible]
Endless_Summer_g17512	Endless_Summer_g17512	Endless Summer genom	Primary assembly	tr|A0A2N9HBE1|A0A2N9HBE1_FAGSY	44.1	tr|A0A2N9HBE1|A0A2N9HBE1_FAGSY Uncharacterized protein OS=Fagus sylvatica OX=28930 GN=FSB_LOCUS35823 PE=	fagus sylvatica	39947.LOC_Os07g05280.1		[illegible]	[illegible]	[illegible]
Endless_Summer_g18024	Endless_Summer_g18024	Endless Summer genom	Primary assembly	tr|A0A3S3NQK9|A0A3S3NQK9_9MAGN	52.7	tr|A0A3S3NQK9|A0A3S3NQK9_9MAGN DNA-directed DNA polymerase OS=Cinnamomum micranthum f. kanehirae OX=337	cinnamomum micranthum f.	39947.LOC_Os04g02180.1		[illegible]	[illegible]	[illegible]
Endless_Summer_g18108	Endless_Summer_g18108	Endless Summer genom	Primary assembly	tr|A0A5J5AZN9|A0A5J5AZN9_9ASTE	59.5	tr|A0A5J5AZN9|A0A5J5AZN9_9ASTE Uncharacterized protein OS=Nyssa sinensis OX=561372 GN=F0562_031968 PE=4 SV	nyssa sinensis	29760.VIT_13s0019g04950.t01	Early transcription elongation factor of RNA pol II, NGN section	[illegible]	[illegible]	[illegible]
Endless_Summer_g18133	Endless_Summer_g18133	Endless Summer genom	Primary assembly	tr|A0A5B7ASX1|A0A5B7ASX1_DAVIN	83.2	tr|A0A5B7ASX1|A0A5B7ASX1_DAVIN NAC domain-containing protein OS=Davidia involucrata OX=16924 GN=Din_02911	davidia involucrata	4113.PGSC0003DMT400079791	No apical meristem (NAM) protein	[illegible]	[illegible]	[illegible]
Endless_Summer_g18345	Endless_Summer_g18345	Endless Summer genom	Primary assembly	tr|A0A4S4E4A4|A0A4S4E4A4_CAMSI	48.1	tr|A0A4S4E4A4|A0A4S4E4A4_CAMSI Uncharacterized protein OS=Camellia sinensis var. sinensis OX=542762 GN=TEA_02	camellia sinensis var. sinen	3694.POPTR_0002s24900.1	NA	[illegible]	[illegible]	[illegible]
Endless_Summer_g18450	Endless_Summer_g18450	Endless Summer genom	Primary assembly	XP_028085390.1	65	XP_028085390.1 uncharacterized protein LOC114286423 [Camellia sinensis]	camellia sinensis	4113.PGSC0003DMT400073840	NA	[illegible]	[illegible]	[illegible]
Endless_Summer_g18561	Endless_Summer_g18561	Endless Summer genom	Primary assembly	tr|A0A834HG83|A0A834HG83_RHOSS	33.3	tr|A0A834HG83|A0A834HG83_RHOSS PMD domain-containing protein OS=Rhododendron simsii OX=118357 GN=RHSIM	rhododendron simsii	29760.VIT_08s0007g08180.t01	NA	[illegible]	[illegible]	[illegible]
Endless_Summer_g18754	Endless_Summer_g18754	Endless Summer genom	Primary assembly	tr|A0A6P6SPU4|A0A6P6SPU4_COFAR	30	tr|A0A6P6SPU4|A0A6P6SPU4_COFAR uncharacterized protein LOC113693571 OS=Coffea arabica OX=13443 GN=LOC1136	coffea arabica					
Endless_Summer_g18779	Endless_Summer_g18779	Endless Summer genom	Primary assembly	tr|A0A5J4ZKS5|A0A5J4ZKS5_9ASTE	48.5	tr|A0A5J4ZKS5|A0A5J4ZKS5_9ASTE Phorbol-ester/DAG-type domain-containing protein OS=Nyssa sinensis OX=561372 GN	nyssa sinensis	3694.POPTR_0016s05820.1	[illegible]	[illegible]	[illegible]	[illegible]
Endless_Summer_g18810	Endless_Summer_g18810	Endless Summer genom	Primary assembly	XP_028056401.1	61.7	XP_028056401.1 breast cancer type 1 susceptibility protein homolog [Camellia sinensis]	camellia sinensis	4113.PGSC0003DMT400029692	[illegible]	[illegible]	[illegible]	[illegible]
Endless_Summer_g18919	Endless_Summer_g18919	Endless Summer genom	Primary assembly	tr|I4EMN3|I4EMN3_9CHLR	45.2	tr|I4EMN3|I4EMN3_9CHLR Putative RNA-directed DNA polymerase OS=Nitrolancea hollandica Lb OX=1129897 GN=NITHO	nitrolancea hollandica lb	3694.POPTR_0011s01230.1	[illegible]	[illegible]	[illegible]	[illegible]
Endless_Summer_g1892	Endless_Summer_g1892	Endless Summer genom	Primary assembly	tr|A0A803MT76|A0A803MT76_CHEQI	40.7	tr|A0A803MT76|A0A803MT76_CHEQI RNase H type-1 domain-containing protein OS=Chenopodium quinoa OX=63459 PE=	chenopodium quinoa					
Endless_Summer_g18962	Endless_Summer_g18962	Endless Summer genom	Primary assembly	tr|A0A5J4ZEN5|A0A5J4ZEN5_9ASTE	45.1	tr|A0A5J4ZEN5|A0A5J4ZEN5_9ASTE Phorbol-ester/DAG-type domain-containing protein OS=Nyssa sinensis OX=561372 GN	nyssa sinensis	3694.POPTR_0016s05820.1	[illegible]	[illegible]	[illegible]	[illegible]
Endless_Summer_g19260	Endless_Summer_g19260	Endless Summer genom	Primary assembly	tr|D7SPV8|D7SPV8_VITVI	58.1	tr|D7SPV8|D7SPV8_VITVI ASCH domain-containing protein OS=Vitis vinifera OX=29760 GN=VIT_00s0226g00060 PE=4 S	vitis vinifera	29760.VIT_00s0226g00060.t01	[illegible]	[illegible]	[illegible]	[illegible]
Endless_Summer_g19336	Endless_Summer_g19336	Endless Summer genom	Primary assembly	tr|A0A4U1CTW9|A0A4U1CTW9_9BACI	25.3	tr|A0A4U1CTW9|A0A4U1CTW9_9BACI Retrotrans_gag domain-containing protein (Fragment) OS=Robertmurraya kyonggien	robertmurraya kyonggiensis	39947.LOC_Os08g16000.1		[illegible]	[illegible]	[illegible]
Endless_Summer_g19372	Endless_Summer_g19372	Endless Summer genom	Primary assembly	tr|A0A7J7FX90|A0A7J7FX90_CAMSI	42.5	tr|A0A7J7FX90|A0A7J7FX90_CAMSI SWIM-type domain-containing protein OS=Camellia sinensis OX=4442 GN=HYC85_0	camellia sinensis	29760.VIT_03s0088g01070.t01	Pfam:MuDR	[illegible]	[illegible]	[illegible]
Endless_Summer_g19429	Endless_Summer_g19429	Endless Summer genom	Primary assembly	tr|A0A2I4EGG2|A0A2I4EGG2_JUGRE	52.7	tr|A0A2I4EGG2|A0A2I4EGG2_JUGRE zinc finger BED domain-containing protein RICESLEEPER 2-like OS=Juglans regia OX	juglans regia	3694.POPTR_0004s11480.1	Pfam:hATC	[illegible]	[illegible]	[illegible]
Endless_Summer_g1949	Endless_Summer_g1949	Endless Summer genom	Primary assembly	XP_028094338.1	84.3	XP_028094338.1 tubulin-folding cofactor B [Camellia sinensis]	camellia sinensis	29760.VIT_08s0007g05420.t01	[illegible]	[illegible]	[illegible]	[illegible]
Endless_Summer_g19750	Endless_Summer_g19750	Endless Summer genom	Primary assembly	XP_038704726.1	29	XP_038704726.1 uncharacterized protein LOC120000672 [Tripterygium wilfordii]	tripterygium wilfordii	4558.Sb01g011130.1	[illegible]	[illegible]	[illegible]	[illegible]
Endless_Summer_g19751	Endless_Summer_g19751	Endless Summer genom	Primary assembly	tr|A0A5J9TTS4|A0A5J9TTS4_9POAL	30.8	tr|A0A5J9TTS4|A0A5J9TTS4_9POAL ATP-dependent DNA helicase (Fragment) OS=Eragrostis curvula OX=38414 GN=EJB	eragrostis curvula	15368.BRADI1G56107.1	Inherit from euNOG: helicase-like protein	[illegible]	[illegible]	[illegible]
Endless_Summer_g19800	Endless_Summer_g19800	Endless Summer genom	Primary assembly	tr|M1A9U7|M1A9U7_SOLTU	40.5	tr|M1A9U7|M1A9U7_SOLTU RNase H family protein OS=Solanum tuberosum OX=4113 PE=4 SV=1	solanum tuberosum					
Endless_Summer_g19940	Endless_Summer_g19940	Endless Summer genom	Primary assembly	tr|A0A2N9IXV4|A0A2N9IXV4_FAGSY	49.8	tr|A0A2N9IXV4|A0A2N9IXV4_FAGSY RNA-directed DNA polymerase OS=Fagus sylvatica OX=28930 GN=FSB_LOCUS7	fagus sylvatica	39947.LOC_Os12g28970.1		[illegible]	[illegible]	[illegible]
Endless_Summer_g1996	Endless_Summer_g1996	Endless Summer genom	Primary assembly	XP_021649615.1	47.2	XP_021649615.1 uncharacterized protein LOC110642002 [Hevea brasiliensis]	hevea brasiliensis	4577.GRMZM2G002944_P01		[illegible]	[illegible]	[illegible]
Endless_Summer_g20026	Endless_Summer_g20026	Endless Summer genom	Primary assembly	XP_011008584.1	36.4	XP_011008584.1 PREDICTED: zinc finger BED domain-containing protein RICESLEEPER 1-like [Populus euphratica]	populus euphratica	3694.POPTR_0004s11480.1	Pfam:hATC	[illegible]	[illegible]	[illegible]
Endless_Summer_g20162	Endless_Summer_g20162	Endless Summer genom	Primary assembly	tr|A0A2K3N4W3|A0A2K3N4W3_TRIPR	45.6	tr|A0A2K3N4W3|A0A2K3N4W3_TRIPR Uncharacterized protein OS=Trifolium pratense OX=57577 GN=L195_g021334 PE=	trifolium pratense	3711.Bra039006.1-P	[illegible]	[illegible]	[illegible]	[illegible]
Endless_Summer_g20189	Endless_Summer_g20189	Endless Summer genom	Primary assembly	XP_045795534.1	70.6	XP_045795534.1 uncharacterized protein LOC123890043 [Trifolium pratense]	trifolium pratense	29760.VIT_07s0005g06030.t01	[illegible]	[illegible]	[illegible]	[illegible]
Endless_Summer_g20216	Endless_Summer_g20216	Endless Summer genom	Primary assembly	XP_022869535.1	62.3	XP_022869535.1 WD repeat-containing protein 44-like [Olea europaea var. sylvestris]	olea europaea var. sylvestri	4081.Solyc09g010720.2.1	[illegible]	[illegible]	[illegible]	[illegible]
Endless_Summer_g20644	Endless_Summer_g20644	Endless Summer genom	Primary assembly	XP_028069049.1	75.1	XP_028069049.1 glucose-1-phosphate adenylyltransferase large subunit, chloroplastic/amyloplastic-like [Camellia sinensis]	camellia sinensis	3694.POPTR_0005s25130.1	This protein plays a role in synthesis of starch. It catalyzes the synthesis of	[illegible]	[illegible]	[illegible]
Endless_Summer_g20772	Endless_Summer_g20772	Endless Summer genom	Primary assembly	tr|A0A6A1UXL9|A0A6A1UXL9_9ROSI	43.1	tr|A0A6A1UXL9|A0A6A1UXL9_9ROSI Uncharacterized protein OS=Morella rubra OX=262757 GN=CJ030_MR7G016717 PE	morella rubra	4558.Sb06g017960.1	NA	[illegible]	[illegible]	[illegible]
Endless_Summer_g20803	Endless_Summer_g20803	Endless Summer genom	Primary assembly	tr|A0A371IH63|A0A371IH63_MUCPR	33.2	tr|A0A371IH63|A0A371IH63_MUCPR G-patch domain-containing protein (Fragment) OS=Mucuna pruriens OX=157652 GN=	mucuna pruriens	29760.VIT_00s0823g00010.t01	[illegible]	[illegible]	[illegible]	[illegible]
Endless_Summer_g20821	Endless_Summer_g20821	Endless Summer genom	Primary assembly	tr|A0A438I7X6|A0A438I7X6_VITVI	50.8	tr|A0A438I7X6|A0A438I7X6_VITVI Retrovirus-related Pol polyprotein from transposon 17.6 OS=Vitis vinifera OX=29760 GN	vitis vinifera	3694.POPTR_0011s01230.1	[illegible]	[illegible]	[illegible]	[illegible]
Endless_Summer_g20822	Endless_Summer_g20822	Endless Summer genom	Primary assembly	tr|A0A438KGK6|A0A438KGK6_VITVI	65.4	tr|A0A438KGK6|A0A438KGK6_VITVI Gypsy retrotransposon integrase-like protein 1 OS=Vitis vinifera OX=29760 GN=GIN1	vitis vinifera	39947.LOC_Os01g06700.1	[illegible]	[illegible]	[illegible]	[illegible]
Endless_Summer_g20839	Endless_Summer_g20839	Endless Summer genom	Primary assembly	XP_042991545.1	73.8	XP_042991545.1 heat shock 70 kDa protein 17 [Carya illinoinensis]	carya illinoinensis	29760.VIT_04s0008g04410.t01	[illegible]	[illegible]	[illegible]	[illegible]
Endless_Summer_g20961	Endless_Summer_g20961	Endless Summer genom	Primary assembly	XP_027183975.1	46.9	XP_027183975.1 uncharacterized protein LOC113782279 [Coffea eugenioides]	coffea eugenioides	3694.POPTR_0005s27380.1	NA	[illegible]	[illegible]	[illegible]
Endless_Summer_g20980	Endless_Summer_g20980	Endless Summer genom	Primary assembly	XP_002519682.1	79.5	XP_002519682.1 calcyclin-binding protein [Ricinus communis]	ricinus communis	4113.PGSC0003DMT400031531	[illegible]	[illegible]	[illegible]	[illegible]
Endless_Summer_g21005	Endless_Summer_g21005	Endless Summer genom	Primary assembly	tr|A0A6P6T1C2|A0A6P6T1C2_COFAR	55.6	tr|A0A6P6T1C2|A0A6P6T1C2_COFAR uncharacterized protein LOC113696623 OS=Coffea arabica OX=13443 GN=LOC1136	coffea arabica	39947.LOC_Os11g45000.1		[illegible]	[illegible]	[illegible]
Endless_Summer_g21077	Endless_Summer_g21077	Endless Summer genom	Primary assembly	XP_021982040.1	80	XP_021982040.1 40S ribosomal protein S14-3 [Helianthus annuus]	helianthus annuus	4113.PGSC0003DMT400054512	40S ribosomal protein	[illegible]	[illegible]	[illegible]
Endless_Summer_g21171	Endless_Summer_g21171	Endless Summer genom	Primary assembly	XP_042980335.1	55.4	XP_042980335.1 zinc finger BED domain-containing protein RICESLEEPER 2-like isoform X1 [Carya illinoinensis]	carya illinoinensis	3694.POPTR_0001s40220.1	Pfam:hATC	[illegible]	[illegible]	[illegible]
Endless_Summer_g2125	Endless_Summer_g2125	Endless Summer genom	Primary assembly	XP_028087632.1	43.4	XP_028087632.1 protein FAR1-RELATED SEQUENCE 4-like [Camellia sinensis]	camellia sinensis	29760.VIT_14s0060g00370.t01	Pfam:MuDR	[illegible]	[illegible]	[illegible]
Endless_Summer_g2141	Endless_Summer_g2141	Endless Summer genom	Primary assembly	XP_028102249.1	77.2	XP_028102249.1 cathepsin B-like protease 3 [Camellia sinensis]	camellia sinensis	3694.POPTR_0002s18510.1	[illegible]	[illegible]	[illegible]	[illegible]
Endless_Summer_g21476	Endless_Summer_g21476	Endless Summer genom	Primary assembly	XP_028092033.1	65.3	XP_028092033.1 transcription factor bHLH30-like [Camellia sinensis]	camellia sinensis	3847.GLYMA07G32690.1	Transcription factor	[illegible]	[illegible]	[illegible]
Endless_Summer_g21489	Endless_Summer_g21489	Endless Summer genom	Primary assembly	tr|A0A6L9M4E1|A0A6L9M4E1_9DELT	76.2	tr|A0A6L9M4E1|A0A6L9M4E1_9DELT Reverse transcriptase Ty1/copia-type domain-containing protein (Fragment) OS=Pseud	pseudodesulfovibrio sp. jc9	39947.LOC_Os06g28750.1		[illegible]	[illegible]	[illegible]
Endless_Summer_g21525	Endless_Summer_g21525	Endless Summer genom	Primary assembly	XP_034697164.1	67.3	XP_034697164.1 spermidine sinapoyl-CoA acyltransferase [Vitis riparia]	vitis riparia	29760.VIT_10s0003g00900.t01	[illegible]	[illegible]	[illegible]	[illegible]
Endless_Summer_g21577	Endless_Summer_g21577	Endless Summer genom	Primary assembly	tr|A0A4Q3E9K1|A0A4Q3E9K1_9SPHI	43	tr|A0A4Q3E9K1|A0A4Q3E9K1_9SPHI Uncharacterized protein OS=Sphingobacteriaceae bacterium OX=2021370 GN=EOP45_1	sphingobacteriaceae bacteri	39947.LOC_Os09g08590.1	[illegible]	[illegible]	[illegible]	[illegible]
Endless_Summer_g21748	Endless_Summer_g21748	Endless Summer genom	Primary assembly	tr|A0A5E4GH82|A0A5E4GH82_PRUDU	45.1	tr|A0A5E4GH82|A0A5E4GH82_PRUDU DBD_Tnp_Mut domain-containing protein (Fragment) OS=Prunus dulcis OX=3755 C	prunus dulcis	4558.Sb01g027410.1	Pfam:MuDR	[illegible]	[illegible]	[illegible]
Endless_Summer_g2183	Endless_Summer_g2183	Endless Summer genom	Primary assembly	XP_012088472.1	75.1	XP_012088472.1 methyltransferase-like protein 23 isoform X1 [Jatropha curcas]	jatropha curcas	29760.VIT_00s0510g00020.t01	Putative methyltransferase	[illegible]	[illegible]	[illegible]
Endless_Summer_g21890	Endless_Summer_g21890	Endless Summer genom	Primary assembly	XP_030941866.1	40.8	XP_030941866.1 uncharacterized protein LOC115966862 [Quercus lobata]	quercus lobata	39947.LOC_Os11g38300.1	[illegible]	[illegible]	[illegible]	[illegible]
Endless_Summer_g21979	Endless_Summer_g21979	Endless Summer genom	Primary assembly	XP_028065195.1	68	XP_028065195.1 UPF0481 protein At3g47200-like [Camellia sinensis]	camellia sinensis	4113.PGSC0003DMT400016684	[illegible]	[illegible]	[illegible]	[illegible]
Endless_Summer_g22158	Endless_Summer_g22158	Endless Summer genom	Primary assembly	tr|A0A5J5AZ85|A0A5J5AZ85_9ASTE	45.9	tr|A0A5J5AZ85|A0A5J5AZ85_9ASTE Uncharacterized protein OS=Nyssa sinensis OX=561372 GN=F0562_030754 PE=4 SV=	nyssa sinensis	4577.GRMZM2G345597_P01	[illegible]	[illegible]	[illegible]	[illegible]
Endless_Summer_g22171	Endless_Summer_g22171	Endless Summer genom	Primary assembly	XP_023901326.1	61.6	XP_023901326.1 zinc finger BED domain-containing protein RICESLEEPER 1-like [Quercus suber]	quercus suber	3694.POPTR_0012s05700.1	Pfam:hATC	[illegible]	[illegible]	[illegible]
Endless_Summer_g22265	Endless_Summer_g22265	Endless Summer genom	Primary assembly	tr|A0A7J7GU63|A0A7J7GU63_CAMSI	40.7	tr|A0A7J7GU63|A0A7J7GU63_CAMSI Uncharacterized protein OS=Camellia sinensis OX=4442 GN=HYC85_018386 PE=4 S	camellia sinensis	4641.GSMUA_Achr3P16240_001	Heat Shock Protein	[illegible]	[illegible]	[illegible]
Endless_Summer_g2228	Endless_Summer_g2228	Endless Summer genom	Primary assembly	XP_023903868.1	61.4	XP_023903868.1 probable LRR receptor-like serine/threonine-protein kinase At3g47570 [Quercus suber]	quercus suber	3694.POPTR_0018s02450.1	[illegible]	[illegible]	[illegible]	[illegible]
Endless_Summer_g22309	Endless_Summer_g22309	Endless Summer genom	Primary assembly	XP_028098420.1	36.7	XP_028098420.1 uncharacterized protein LOC114298114 [Camellia sinensis]	camellia sinensis	3694.POPTR_0013s10690.1		[illegible]	[illegible]	[illegible]
Endless_Summer_g22398	Endless_Summer_g22398	Endless Summer genom	Primary assembly	tr|A0A2N9FAG3|A0A2N9FAG3_FAGSY	50.4	tr|A0A2N9FAG3|A0A2N9FAG3_FAGSY Uncharacterized protein OS=Fagus sylvatica OX=28930 GN=FSB_LOCUS11977 PE	fagus sylvatica	29760.VIT_02s0087g01020.t01	Cysteine-rich receptor-like protein kinase	[illegible]	[illegible]	[illegible]
Endless_Summer_g22483	Endless_Summer_g22483	Endless Summer genom	Primary assembly	XP_028096242.1	65.1	XP_028096242.1 uncharacterized protein LOC114296158 [Camellia sinensis]	camellia sinensis	4533.OB10G12270.1	[illegible]	[illegible]	[illegible]	[illegible]
Endless_Summer_g2254	Endless_Summer_g2254	Endless Summer genom	Primary assembly	XP_030970905.1	46.7	XP_030970905.1 receptor kinase-like protein Xa21 [Quercus lobata]	quercus lobata	29760.VIT_09s0018g01080.t01		[illegible]	[illegible]	[illegible]
Endless_Summer_g23096	Endless_Summer_g23096	Endless Summer genom	Primary assembly	tr|A0A7J7G2X8|A0A7J7G2X8_CAMSI	45.8	tr|A0A7J7G2X8|A0A7J7G2X8_CAMSI Uncharacterized protein OS=Camellia sinensis OX=4442 GN=HYC85_025986 PE=4 S	camellia sinensis	39947.LOC_Os08g25960.1		[illegible]	[illegible]	[illegible]
Endless_Summer_g23222	Endless_Summer_g23222	Endless Summer genom	Primary assembly	XP_034696611.1	65.2	XP_034696611.1 probable leucine-rich repeat receptor-like protein kinase At1g68400 [Vitis riparia]	vitis riparia	29760.VIT_16s0039g00230.t01	receptor kinase	[illegible]	[illegible]	[illegible]
Endless_Summer_g23226	Endless_Summer_g23226	Endless Summer genom	Primary assembly	XP_018835535.1	82	XP_018835535.1 methionine aminopeptidase 2B-like [Juglans regia]	juglans regia	3702.AT3G59990.1	Removes the N-terminal methionine from nascent proteins (By similarity)	[illegible]	[illegible]	[illegible]
Endless_Summer_g23414	Endless_Summer_g23414	Endless Summer genom	Primary assembly	XP_028071092.1	29.9	XP_028071092.1 uncharacterized protein LOC114273489 isoform X2 [Camellia sinensis]	camellia sinensis	4577.GRMZM2G107943_P01	[illegible]	[illegible]	[illegible]	[illegible]
Endless_Summer_g23419	Endless_Summer_g23419	Endless Summer genom	Primary assembly	tr|A0A7N2L2M4|A0A7N2L2M4_QUELO	46.9	tr|A0A7N2L2M4|A0A7N2L2M4_QUELO BED-type domain-containing protein OS=Quercus lobata OX=97700 PE=4 SV=1	quercus lobata	3847.GLYMA04G17450.1	[illegible]	[illegible]	[illegible]	[illegible]
Endless_Summer_g23673	Endless_Summer_g23673	Endless Summer genom	Primary assembly	tr|A0A5C2SMV7|A0A5C2SMV7_9APHY	47	tr|A0A5C2SMV7|A0A5C2SMV7_9APHY Helitron_like_N domain-containing protein (Fragment) OS=Lentinus tigrinus ALCF	lentinus tigrinus alcf2ss1-6	240176.XP_001835316.2	Inherit from KOG: DNA helicase	[illegible]	[illegible]	[illegible]
Endless_Summer_g237	Endless_Summer_g237	Endless Summer genom	Primary assembly	tr|A0A4S8I6Z0|A0A4S8I6Z0_MUSBA	54	tr|A0A4S8I6Z0|A0A4S8I6Z0_MUSBA 30S ribosomal protein S8, chloroplastic OS=Musa balbisiana OX=52838 GN=C4D60_	musa balbisiana	4081.Solyc01g007590.2.1	[illegible]	[illegible]	[illegible]	[illegible]
Endless_Summer_g23774	Endless_Summer_g23774	Endless Summer genom	Primary assembly	tr|A0A7N2M1X7|A0A7N2M1X7_QUELO	63	tr|A0A7N2M1X7|A0A7N2M1X7_QUELO BED-type domain-containing protein OS=Quercus lobata OX=97700 PE=4 SV=1	quercus lobata	3694.POPTR_0004s11480.1	Pfam:hATC	[illegible]	[illegible]	[illegible]
Endless_Summer_g23884	Endless_Summer_g23884	Endless Summer genom	Primary assembly	XP_030941647.1	42.6	XP_030941647.1 uncharacterized protein LOC115966579 [Quercus lobata]	quercus lobata	39947.LOC_Os05g06390.1	[illegible]	[illegible]	[illegible]	[illegible]
Endless_Summer_g23932	Endless_Summer_g23932	Endless Summer genom	Primary assembly	tr|A0A4Q3EEW7|A0A4Q3EEW7_9SPHI	48.3	tr|A0A4Q3EEW7|A0A4Q3EEW7_9SPHI DUF4413 domain-containing protein (Fragment) OS=Sphingobacteriaceae bacterium C	sphingobacteriaceae bacteri	3694.POPTR_0004s11480.1	Pfam:hATC	[illegible]	[illegible]	[illegible]
Endless_Summer_g24092	Endless_Summer_g24092	Endless Summer genom	Primary assembly	XP_028105773.1	67.1	XP_028105773.1 1-aminocyclopropane-1-carboxylate oxidase homolog 1-like [Camellia sinensis]	camellia sinensis	29760.VIT_05s0049g00310.t01	[illegible]	[illegible]	[illegible]	[illegible]
Endless_Summer_g24178	Endless_Summer_g24178	Endless Summer genom	Primary assembly	XP_038981135.1	34.8	XP_038981135.1 uncharacterized protein K02A2.6-like [Phoenix dactylifera]	phoenix dactylifera	3711.Bra017917.1-P	[illegible]	[illegible]	[illegible]	[illegible]
Endless_Summer_g24285	Endless_Summer_g24285	Endless Summer genom	Primary assembly	tr|A0A438KRC4|A0A438KRC4_VITVI	54.8	tr|A0A438KRC4|A0A438KRC4_VITVI Retrovirus-related Pol polyprotein from transposon RE1 OS=Vitis vinifera OX=29760	vitis vinifera	3711.Bra015746.1-P	[illegible]	[illegible]	[illegible]	[illegible]
Endless_Summer_g24370	Endless_Summer_g24370	Endless Summer genom	Primary assembly	XP_023914847.1	73.5	XP_023914847.1 basic 7S globulin-like [Quercus suber]	quercus suber	4081.Solyc01g080010.2.1	Basic 7S	[illegible]	[illegible]	[illegible]
Endless_Summer_g24584	Endless_Summer_g24584	Endless Summer genom	Primary assembly	tr|A0A2P5C6Z2|A0A2P5C6Z2_PARAD	26.9	tr|A0A2P5C6Z2|A0A2P5C6Z2_PARAD Uncharacterized protein (Fragment) OS=Parasponia andersonii OX=3476 GN=PanWU	parasponia andersonii					
Endless_Summer_g2459	Endless_Summer_g2459	Endless Summer genom	Primary assembly	tr|A0A438KBJ0|A0A438KBJ0_VITVI	49.3	tr|A0A438KBJ0|A0A438KBJ0_VITVI Retrovirus-related Pol polyprotein from transposon TNT 1-94 OS=Vitis vinifera OX=297	vitis vinifera	39947.LOC_Os08g17750.1	[illegible]	[illegible]	[illegible]	[illegible]
Endless_Summer_g24757	Endless_Summer_g24757	Endless Summer genom	Primary assembly	XP_030945859.1	33.8	XP_030945859.1 pectinesterase inhibitor-like [Quercus lobata]	quercus lobata	29760.VIT_07s0005g01580.t01	[illegible]	[illegible]	[illegible]	[illegible]
Endless_Summer_g24861	Endless_Summer_g24861	Endless Summer genom	Primary assembly	tr|A0A5J4ZF50|A0A5J4ZF50_9ASTE	65.6	tr|A0A5J4ZF50|A0A5J4ZF50_9ASTE DUF4216 domain-containing protein OS=Nyssa sinensis OX=561372 GN=F0562_0174	nyssa sinensis	39947.LOC_Os03g50670.1		[illegible]	[illegible]	[illegible]
Endless_Summer_g24898	Endless_Summer_g24898	Endless Summer genom	Primary assembly	XP_010106380.2	70.2	XP_010106380.2 mitochondrial zinc maintenance protein 1, mitochondrial [Morus notabilis]	morus notabilis	3694.POPTR_0014s12430.1	[illegible]	[illegible]	[illegible]	[illegible]
Endless_Summer_g24915	Endless_Summer_g24915	Endless Summer genom	Primary assembly	tr|A0A5J5C5I2|A0A5J5C5I2_9ASTE	77.8	tr|A0A5J5C5I2|A0A5J5C5I2_9ASTE AAA domain-containing protein OS=Nyssa sinensis OX=561372 GN=F0562_000856 PE	nyssa sinensis	4081.Solyc06g084530.2.1	[illegible]	[illegible]	[illegible]	[illegible]
Endless_Summer_g24955	Endless_Summer_g24955	Endless Summer genom	Primary assembly	XP_038709419.1	63.1	XP_038709419.1 uncharacterized protein LOC120004222 [Tripterygium wilfordii]	tripterygium wilfordii	4577.GRMZM2G002718_P01	[illegible]	[illegible]	[illegible]	[illegible]
Endless_Summer_g25107	Endless_Summer_g25107	Endless Summer genom	Primary assembly	tr|A0A7J7FYF1|A0A7J7FYF1_CAMSI	32.3	tr|A0A7J7FYF1|A0A7J7FYF1_CAMSI Uncharacterized protein OS=Camellia sinensis OX=4442 GN=HYC85_029530 PE=4 S	camellia sinensis					
Endless_Summer_g25117	Endless_Summer_g25117	Endless Summer genom	Primary assembly	tr|A0A5N5F9K3|A0A5N5F9K3_9ROSA	39.2	tr|A0A5N5F9K3|A0A5N5F9K3_9ROSA Uncharacterized protein OS=Pyrus ussuriensis x Pyrus communis OX=2448454 GN=	pyrus ussuriensis x pyrus co	39947.LOC_Os10g23870.1		[illegible]	[illegible]	[illegible]
Endless_Summer_g25118	Endless_Summer_g25118	Endless Summer genom	Primary assembly	tr|A0A2N9G5Z3|A0A2N9G5Z3_FAGSY	40.1	tr|A0A2N9G5Z3|A0A2N9G5Z3_FAGSY Uncharacterized protein OS=Fagus sylvatica OX=28930 GN=FSB_LOCUS22493 PE=	fagus sylvatica	4577.GRMZM2G132874_P01	[illegible]	[illegible]	[illegible]	[illegible]
Endless_Summer_g25350	Endless_Summer_g25350	Endless Summer genom	Primary assembly	tr|A0A3S1MGG9|A0A3S1MGG9_9HYPH	58.9	tr|A0A3S1MGG9|A0A3S1MGG9_9HYPH ACP_syn_III_C domain-containing protein (Fragment) OS=Mesorhizobium sp. M7A	mesorhizobium sp. m7a.f.ca	29760.VIT_07s0141g00090.t01	[illegible]	[illegible]	[illegible]	[illegible]
Endless_Summer_g25457	Endless_Summer_g25457	Endless Summer genom	Primary assembly	tr|A0A5J4ZPD0|A0A5J4ZPD0_9ASTE	36.7	tr|A0A5J4ZPD0|A0A5J4ZPD0_9ASTE CCHC-type domain-containing protein OS=Nyssa sinensis OX=561372 GN=F0562_01	nyssa sinensis	39947.LOC_Os03g61660.1		[illegible]	[illegible]	[illegible]
Endless_Summer_g25546	Endless_Summer_g25546	Endless Summer genom	Primary assembly	tr|A0A5A7VHG7|A0A5A7VHG7_CUCME	58.2	tr|A0A5A7VHG7|A0A5A7VHG7_CUCME Pol protein OS=Cucumis melo var. makuwa OX=1194695 GN=E6C27_scaffold657	cucumis melo var. makuwa	39947.LOC_Os11g39720.1	Chromo (CHRromatin Organisation MOdifier) domain	[illegible]	[illegible]	[illegible]
Endless_Summer_g25668	Endless_Summer_g25668	Endless Summer genom	Primary assembly	tr|A5AGZ0|A5AGZ0_VITVI	51.2	tr|A5AGZ0|A5AGZ0_VITVI Uncharacterized protein OS=Vitis vinifera OX=29760 GN=VITISV_007002 PE=3 SV=1	vitis vinifera	39947.LOC_Os08g01570.1	[illegible]	[illegible]	[illegible]	[illegible]
Endless_Summer_g25714	Endless_Summer_g25714	Endless Summer genom	Primary assembly	XP_023891781.1	53	XP_023891781.1 zinc finger BED domain-containing protein RICESLEEPER 1-like [Quercus suber]	quercus suber	3694.POPTR_0004s11480.1	Pfam:hATC	[illegible]	[illegible]	[illegible]
Endless_Summer_g25791	Endless_Summer_g25791	Endless Summer genom	Primary assembly	XP_030946465.1	53.6	XP_030946465.1 zinc finger BED domain-containing protein RICESLEEPER 2-like [Quercus lobata]	quercus lobata	3847.GLYMA14G13178.1	Pfam:hATC	[illegible]	[illegible]	[illegible]
Endless_Summer_g25893	Endless_Summer_g25893	Endless Summer genom	Primary assembly	XP_028080124.1	50.3	XP_028080124.1 uncharacterized protein LOC114281463 isoform X3 [Camellia sinensis]	camellia sinensis	29760.VIT_06s0004g02520.t01	Myb-like DNA-binding domain	[illegible]	[illegible]	[illegible]
Endless_Summer_g25929	Endless_Summer_g25929	Endless Summer genom	Primary assembly	XP_027166685.1	38.9	XP_027166685.1 uncharacterized protein LOC113766722 [Coffea eugenioides]	coffea eugenioides	39947.LOC_Os12g35940.1	[illegible]	[illegible]	[illegible]	[illegible]
Endless_Summer_g26336	Endless_Summer_g26336	Endless Summer genom	Primary assembly	XP_022877831.1	62.9	XP_022877831.1 uncharacterized protein LOC111395863 [Olea europaea var. sylvestris]	olea europaea var. sylvestri	3694.POPTR_0004s03580.1	[illegible]	[illegible]	[illegible]	[illegible]
Endless_Summer_g26338	Endless_Summer_g26338	Endless Summer genom	Primary assembly	tr|A0A8B8FYA5|A0A8B8FYA5_9HEMI	33.9	tr|A0A8B8FYA5|A0A8B8FYA5_9HEMI 52 kDa repressor of the inhibitor of the protein kinase-like OS=Sipha flava OX=14395	sipha flava	3694.POPTR_0004s03580.1	[illegible]	[illegible]	[illegible]	[illegible]
Endless_Summer_g26340	Endless_Summer_g26340	Endless Summer genom	Primary assembly	tr|A0A2U1PQS8|A0A2U1PQS8_ARTAN	37.8	tr|A0A2U1PQS8|A0A2U1PQS8_ARTAN Dimer_Tnp_hAT domain-containing protein OS=Artemisia annua OX=35608 GN=C	artemisia annua	3694.POPTR_0004s03580.1	[illegible]	[illegible]	[illegible]	[illegible]
Endless_Summer_g26367	Endless_Summer_g26367	Endless Summer genom	Primary assembly	XP_027171712.1	27.6	XP_027171712.1 uncharacterized protein LOC113771315 [Coffea eugenioides]	coffea eugenioides					
Endless_Summer_g26427	Endless_Summer_g26427	Endless Summer genom	Primary assembly	XP_028092293.1	41.8	XP_028092293.1 uncharacterized protein LOC114292508 [Camellia sinensis]	camellia sinensis					
Endless_Summer_g2652	Endless_Summer_g2652	Endless Summer genom	Primary assembly	XP_021816914.1	38.3	XP_021816914.1 uncharacterized protein LOC110759188, partial [Prunus avium]	prunus avium	39947.LOC_Os02g12320.1		[illegible]	[illegible]	[illegible]
Endless_Summer_g26533	Endless_Summer_g26533	Endless Summer genom	Primary assembly	XP_030923123.1	41.1	XP_030923123.1 zinc finger BED domain-containing protein RICESLEEPER 1-like [Quercus lobata]	quercus lobata	3694.POPTR_0012s05700.1	Pfam:hATC	[illegible]	[illegible]	[illegible]
Endless_Summer_g26759	Endless_Summer_g26759	Endless Summer genom	Primary assembly	XP_028111700.1	88.4	XP_028111700.1 protein SHORT-ROOT-like [Camellia sinensis]	camellia sinensis	3694.POPTR_0017s05450.1	Transcription factor	[illegible]	[illegible]	[illegible]
Endless_Summer_g26813	Endless_Summer_g26813	Endless Summer genom	Primary assembly	XP_023876824.1	42.2	XP_023876824.1 putative disease resistance protein RGA3 [Quercus suber]	quercus suber	3694.POPTR_0014s00720.1	[illegible]	[illegible]	[illegible]	[illegible]
Endless_Summer_g26882	Endless_Summer_g26882	Endless Summer genom	Primary assembly	tr|A0A7J0F2B7|A0A7J0F2B7_9ERIC	50.6	tr|A0A7J0F2B7|A0A7J0F2B7_9ERIC Uncharacterized protein OS=Actinidia rufa OX=165716 GN=Acr_08g0012200 PE=4 SV=	actinidia rufa	3694.POPTR_0014s00550.1	[illegible]	[illegible]	[illegible]	[illegible]
Endless_Summer_g26908	Endless_Summer_g26908	Endless Summer genom	Primary assembly	XP_034672574.1	61.5	XP_034672574.1 disease resistance protein RGA2-like [Vitis riparia]	vitis riparia	29760.VIT_08s0007g08420.t01	[illegible]	[illegible]	[illegible]	[illegible]
Endless_Summer_g27035	Endless_Summer_g27035	Endless Summer genom	Primary assembly	XP_042501024.1	70.9	XP_042501024.1 probable serine/threonine-protein kinase abkC isoform X1 [Macadamia integrifolia]	macadamia integrifolia	4641.GSMUA_Achr11P08690_001	[illegible]	[illegible]	[illegible]	[illegible]
Endless_Summer_g27175	Endless_Summer_g27175	Endless Summer genom	Primary assembly	tr|A0A2N9HT52|A0A2N9HT52_FAGSY	41.7	tr|A0A2N9HT52|A0A2N9HT52_FAGSY SWIM-type domain-containing protein OS=Fagus sylvatica OX=28930 GN=FSB_LC	fagus sylvatica	39947.LOC_Os04g27730.1	[illegible]	[illegible]	[illegible]	[illegible]
Endless_Summer_g272	Endless_Summer_g272	Endless Summer genom	Primary assembly	YP_010363890.1	94.2	YP_010363890.1 photosystem II CP47 chlorophyll apoprotein (chloroplast) [Jaltomata sinuosa]	jaltomata sinuosa	4081.Solyc01g007500.2.1	One of the components of the core antenna complex of photosystem II. It b	[illegible]	[illegible]	[illegible]
Endless_Summer_g27211	Endless_Summer_g27211	Endless Summer genom	Primary assembly	tr|A0A7J7FYR7|A0A7J7FYR7_CAMSI	72.5	tr|A0A7J7FYR7|A0A7J7FYR7_CAMSI DUF659 domain-containing protein OS=Camellia sinensis OX=4442 GN=HYC85_02	camellia sinensis	3694.POPTR_0018s06430.1	Protein of unknown function (DUF 659)	[illegible]	[illegible]	[illegible]
Endless_Summer_g2729	Endless_Summer_g2729	Endless Summer genom	Primary assembly	tr|A0A834WMU9|A0A834WMU9_9FABA	43.3	tr|A0A834WMU9|A0A834WMU9_9FABA Zinc finger BED domain-containing protein RICESLEEPER 2-like OS=Senna tora	senna tora	3694.POPTR_0007s00640.1	[illegible]	[illegible]	[illegible]	[illegible]
Endless_Summer_g27332	Endless_Summer_g27332	Endless Summer genom	Primary assembly	tr|A0A5B7BQS6|A0A5B7BQS6_DAVIN	57.3	tr|A0A5B7BQS6|A0A5B7BQS6_DAVIN Reverse transcriptase domain-containing protein (Fragment) OS=Davidia involucrata C	davidia involucrata	39947.LOC_Os01g33750.1		[illegible]	[illegible]	[illegible]
Endless_Summer_g27419	Endless_Summer_g27419	Endless Summer genom	Primary assembly	XP_028054850.1	70.2	XP_028054850.1 probable LRR receptor-like serine/threonine-protein kinase At3g47570 [Camellia sinensis]	camellia sinensis	29760.VIT_08s0007g02110.t01	[illegible]	[illegible]	[illegible]	[illegible]
Endless_Summer_g27430	Endless_Summer_g27430	Endless Summer genom	Primary assembly	tr|A0A2N9IIB3|A0A2N9IIB3_FAGSY	50.2	tr|A0A2N9IIB3|A0A2N9IIB3_FAGSY Uncharacterized protein OS=Fagus sylvatica OX=28930 GN=FSB_LOCUS51701 PE=4	fagus sylvatica	39947.LOC_Os05g06390.1	[illegible]	[illegible]	[illegible]	[illegible]
Endless_Summer_g27546	Endless_Summer_g27546	Endless Summer genom	Primary assembly	tr|A0A5R9QKN8|A0A5R9QKN8_9BACT	37	tr|A0A5R9QKN8|A0A5R9QKN8_9BACT RNA-directed DNA polymerase (Fragment) OS=Labilibacter sediminis OX=2570926	labilibacter sediminis	39947.LOC_Os10g21350.1	[illegible]	[illegible]	[illegible]	[illegible]
Endless_Summer_g2758	Endless_Summer_g2758	Endless Summer genom	Primary assembly	XP_026441803.1	35.2	XP_026441803.1 uncharacterized protein LOC113340978 [Papaver somniferum]	papaver somniferum	39947.LOC_Os08g25640.1	[illegible]	[illegible]	[illegible]	[illegible]
Endless_Summer_g27614	Endless_Summer_g27614	Endless Summer genom	Primary assembly	XP_030970280.1	53.4	XP_030970280.1 secreted RxLR effector protein 161-like [Quercus lobata]	quercus lobata	39947.LOC_Os12g05520.1		[illegible]	[illegible]	[illegible]
Endless_Summer_g27620	Endless_Summer_g27620	Endless Summer genom	Primary assembly	tr|A0A2N9H3S0|A0A2N9H3S0_FAGSY	33	tr|A0A2N9H3S0|A0A2N9H3S0_FAGSY Uncharacterized protein OS=Fagus sylvatica OX=28930 GN=FSB_LOCUS34066 PE=	fagus sylvatica					
Endless_Summer_g27639	Endless_Summer_g27639	Endless Summer genom	Primary assembly	XP_028056126.1	72.3	XP_028056126.1 probable rhamnogalacturonate lyase B isoform X1 [Camellia sinensis]	camellia sinensis	4081.Solyc11g011310.1.1	Rhamnogalacturonate lyase family	[illegible]	[illegible]	[illegible]
Endless_Summer_g27676	Endless_Summer_g27676	Endless Summer genom	Primary assembly	XP_028116257.1	33.5	XP_028116257.1 uncharacterized protein LOC114314023 [Camellia sinensis]	camellia sinensis	3694.POPTR_0004s23230.1	NA	[illegible]	[illegible]	[illegible]
Endless_Summer_g27748	Endless_Summer_g27748	Endless Summer genom	Primary assembly	tr|A0A438D896|A0A438D896_VITVI	33.9	tr|A0A438D896|A0A438D896_VITVI Retrovirus-related Pol polyprotein from transposon RE2 OS=Vitis vinifera OX=29760 GN	vitis vinifera	3711.Bra008452.1-P	[illegible]	[illegible]	[illegible]	[illegible]
Endless_Summer_g27846	Endless_Summer_g27846	Endless Summer genom	Primary assembly	tr|A0A5C7GZP0|A0A5C7GZP0_9ROSI	38.7	tr|A0A5C7GZP0|A0A5C7GZP0_9ROSI Myb_DNA-bind_3 domain-containing protein OS=Acer yangbiense OX=1000413 GN=	acer yangbiense	3847.GLYMA03G06712.1	NA	[illegible]	[illegible]	[illegible]
Endless_Summer_g2791	Endless_Summer_g2791	Endless Summer genom	Primary assembly	XP_028052373.1	86.2	XP_028052373.1 ubiquitin-conjugating enzyme E2 7 isoform X1 [Camellia sinensis]	camellia sinensis	29760.VIT_13s0019g00910.t01	[illegible]	[illegible]	[illegible]	[illegible]
Endless_Summer_g2798	Endless_Summer_g2798	Endless Summer genom	Primary assembly	XP_020402868.1	70.4	XP_020402868.1 protein ALP1-like isoform X1 [Zea mays]	zea mays	4577.GRMZM2G004107_P01	[illegible]	[illegible]	[illegible]	[illegible]
Endless_Summer_g28024	Endless_Summer_g28024	Endless Summer genom	Primary assembly	XP_028078121.1	76.1	XP_028078121.1 GDSL esterase/lipase At3g27950-like [Camellia sinensis]	camellia sinensis	29760.VIT_14s0066g01270.t01	[illegible]	[illegible]	[illegible]	[illegible]
Endless_Summer_g28181	Endless_Summer_g28181	Endless Summer genom	Primary assembly	tr|A0A5B7AUD0|A0A5B7AUD0_DAVIN	41.3	tr|A0A5B7AUD0|A0A5B7AUD0_DAVIN Putative 4-coumarate--CoA ligase-like 9 OS=Davidia involucrata OX=16924 GN=Din	davidia involucrata	29760.VIT_14s0171g00300.t01	[illegible]	[illegible]	[illegible]	[illegible]
Endless_Summer_g28213	Endless_Summer_g28213	Endless Summer genom	Primary assembly	XP_035539989.1	44	XP_035539989.1 uncharacterized protein LOC118344161 [Juglans regia]	juglans regia	39947.LOC_Os05g06390.1	[illegible]	[illegible]	[illegible]	[illegible]
Endless_Summer_g28311	Endless_Summer_g28311	Endless Summer genom	Primary assembly	XP_028118961.1	42.4	XP_028118961.1 uncharacterized protein LOC114316509 [Camellia sinensis]	camellia sinensis	4081.Solyc03g083760.1.1		[illegible]	[illegible]	[illegible]
Endless_Summer_g28358	Endless_Summer_g28358	Endless Summer genom	Primary assembly	tr|A0A6A1WK79|A0A6A1WK79_9ROSI	51.5	tr|A0A6A1WK79|A0A6A1WK79_9ROSI Uncharacterized protein OS=Morella rubra OX=262757 GN=CJ030_MR1G016593 PE	morella rubra	39946.BGIOSGA007282-PA	[illegible]	[illegible]	[illegible]	[illegible]
Endless_Summer_g28446	Endless_Summer_g28446	Endless Summer genom	Primary assembly	tr|A0A438EMN0|A0A438EMN0_VITVI	63.6	tr|A0A438EMN0|A0A438EMN0_VITVI RNA-directed DNA polymerase OS=Vitis vinifera OX=29760 GN=TY3B-I_151 PE=4	vitis vinifera	39947.LOC_Os02g43800.1		[illegible]	[illegible]	[illegible]
Endless_Summer_g2849	Endless_Summer_g2849	Endless Summer genom	Primary assembly	tr|A0A7J7HS79|A0A7J7HS79_CAMSI	58.5	tr|A0A7J7HS79|A0A7J7HS79_CAMSI Uncharacterized protein OS=Camellia sinensis OX=4442 GN=HYC85_008073 PE=4 SV	camellia sinensis	29760.VIT_13s0139g00270.t01		[illegible]	[illegible]	[illegible]
Endless_Summer_g28551	Endless_Summer_g28551	Endless Summer genom	Primary assembly	tr|A0A2N9HQP9|A0A2N9HQP9_FAGSY	36.1	tr|A0A2N9HQP9|A0A2N9HQP9_FAGSY Uncharacterized protein OS=Fagus sylvatica OX=28930 GN=FSB_LOCUS41962 PE	fagus sylvatica					
Endless_Summer_g2862	Endless_Summer_g2862	Endless Summer genom	Primary assembly	tr|A0A444WSB0|A0A444WSB0_ARAHY	31.6	tr|A0A444WSB0|A0A444WSB0_ARAHY Reverse transcriptase domain-containing protein OS=Arachis hypogaea OX=3818 GN	arachis hypogaea	39947.LOC_Os09g37320.1	[illegible]	[illegible]	[illegible]	[illegible]
Endless_Summer_g28625	Endless_Summer_g28625	Endless Summer genom	Primary assembly	XP_028071846.1	50.9	XP_028071846.1 uncharacterized protein LOC114274173 [Camellia sinensis]	camellia sinensis	3847.GLYMA03G12105.1	[illegible]	[illegible]	[illegible]	[illegible]
Endless_Summer_g2882	Endless_Summer_g2882	Endless Summer genom	Primary assembly	tr|A0A7J0GJI9|A0A7J0GJI9_9ERIC	52.4	tr|A0A7J0GJI9|A0A7J0GJI9_9ERIC SWIM-type domain-containing protein OS=Actinidia rufa OX=165716 GN=Acr_22g00030	actinidia rufa	4533.OB04G32400.1	Helix-loop-helix DNA-binding domain	[illegible]	[illegible]	[illegible]
Endless_Summer_g2883	Endless_Summer_g2883	Endless Summer genom	Primary assembly	XP_030955587.1	36.9	XP_030955587.1 uncharacterized protein LOC115977699 isoform X2 [Quercus lobata]	quercus lobata	4577.GRMZM2G132874_P01	SWIM zinc finger	[illegible]	[illegible]	[illegible]
Endless_Summer_g2885	Endless_Summer_g2885	Endless Summer genom	Primary assembly	tr|A0A484MPI8|A0A484MPI8_9ASTE	55.2	tr|A0A484MPI8|A0A484MPI8_9ASTE CCHC-type domain-containing protein OS=Cuscuta campestris OX=132261 GN=CCA	cuscuta campestris	4081.Solyc06g060030.1.1	[illegible]	[illegible]	[illegible]	[illegible]
Endless_Summer_g28977	Endless_Summer_g28977	Endless Summer genom	Primary assembly	tr|A0A5E4FPN5|A0A5E4FPN5_PRUDU	35.4	tr|A0A5E4FPN5|A0A5E4FPN5_PRUDU PREDICTED: reverse mRNAase (Fragment) OS=Prunus dulcis OX=3755 GN=ALMO	prunus dulcis	39947.LOC_Os12g26500.1		[illegible]	[illegible]	[illegible]
Endless_Summer_g29013	Endless_Summer_g29013	Endless Summer genom	Primary assembly	tr|A0A5B7A8Z2|A0A5B7A8Z2_DAVIN	34.8	tr|A0A5B7A8Z2|A0A5B7A8Z2_DAVIN Endo/exonuclease/phosphatase domain-containing protein OS=Davidia involucrata OX=	davidia involucrata	39947.LOC_Os11g08550.1		[illegible]	[illegible]	[illegible]
Endless_Summer_g29058	Endless_Summer_g29058	Endless Summer genom	Primary assembly	XP_028079092.1	43.8	XP_028079092.1 uncharacterized protein LOC114280879 [Camellia sinensis]	camellia sinensis					
Endless_Summer_g2907	Endless_Summer_g2907	Endless Summer genom	Primary assembly	XP_024200409.1	39.1	XP_024200409.1 uncharacterized protein LOC112203718 [Rosa chinensis]	rosa chinensis	39946.BGIOSGA038560-PA	Protein of unknown function (DUF 659)	[illegible]	[illegible]	[illegible]
Endless_Summer_g29174	Endless_Summer_g29174	Endless Summer genom	Primary assembly	tr|A0A6P6WT34|A0A6P6WT34_COFAR	54.8	tr|A0A6P6WT34|A0A6P6WT34_COFAR ATP-dependent DNA helicase OS=Coffea arabica OX=13443 GN=LOC113735936 PI	coffea arabica	39947.LOC_Os05g44786.1	[illegible]	[illegible]	[illegible]	[illegible]
Endless_Summer_g29196	Endless_Summer_g29196	Endless Summer genom	Primary assembly	tr|A0A4R5JDU6|A0A4R5JDU6_9FIRM	58.5	tr|A0A4R5JDU6|A0A4R5JDU6_9FIRM Transposase (Fragment) OS=Zhaonella formicivorans OX=2528593 GN=EYS13_1717	zhaonella formicivorans	4558.Sb10g026363.1	[illegible]	[illegible]	[illegible]	[illegible]
Endless_Summer_g29197	Endless_Summer_g29197	Endless Summer genom	Primary assembly	tr|A0A5J5A4A9|A0A5J5A4A9_9ASTE	34.5	tr|A0A5J5A4A9|A0A5J5A4A9_9ASTE CCHC-type domain-containing protein OS=Nyssa sinensis OX=561372 GN=F0562_01	nyssa sinensis	39947.LOC_Os03g20930.1	Inherit from euNOG: Retrotransposon protein	[illegible]	[illegible]	[illegible]
Endless_Summer_g29312	Endless_Summer_g29312	Endless Summer genom	Primary assembly	tr|A0A2N9GX21|A0A2N9GX21_FAGSY	43.6	tr|A0A2N9GX21|A0A2N9GX21_FAGSY SWIM-type domain-containing protein OS=Fagus sylvatica OX=28930 GN=FSB_LC	fagus sylvatica	39947.LOC_Os07g40760.1	SWIM zinc finger	[illegible]	[illegible]	[illegible]
Endless_Summer_g29400	Endless_Summer_g29400	Endless Summer genom	Primary assembly	tr|A5BND9|A5BND9_VITVI	51.2	tr|A5BND9|A5BND9_VITVI DDE_Tnp4 domain-containing protein OS=Vitis vinifera OX=29760 GN=VITISV_027369 PE=3	vitis vinifera	4081.Solyc09g064290.1.1	[illegible]	[illegible]	[illegible]	[illegible]
Endless_Summer_g29600	Endless_Summer_g29600	Endless Summer genom	Primary assembly	tr|A0A452XT79|A0A452XT79_AEGTS	53.6	tr|A0A452XT79|A0A452XT79_AEGTS ATP-dependent DNA helicase OS=Aegilops tauschii subsp. strangulata OX=200361 P	aegilops tauschii subsp. str	4555.Si019133m	[illegible]	[illegible]	[illegible]	[illegible]
Endless_Summer_g29724	Endless_Summer_g29724	Endless Summer genom	Primary assembly	XP_034690458.1	60.2	XP_034690458.1 pentatricopeptide repeat-containing protein At3g07290, mitochondrial [Vitis riparia]	vitis riparia	29760.VIT_07s0151g00320.t01	[illegible]	[illegible]	[illegible]	[illegible]
Endless_Summer_g29773	Endless_Summer_g29773	Endless Summer genom	Primary assembly	tr|A0A834G328|A0A834G328_RHOSS	38.8	tr|A0A834G328|A0A834G328_RHOSS Uncharacterized protein OS=Rhododendron simsii OX=118357 GN=RHSIM_Rhsim13	rhododendron simsii	3694.POPTR_0006s22910.1	F-box/Kelch-repeat protein	[illegible]	[illegible]	[illegible]
Endless_Summer_g29885	Endless_Summer_g29885	Endless Summer genom	Primary assembly	tr|A0A7J7FTN6|A0A7J7FTN6_CAMSI	38.2	tr|A0A7J7FTN6|A0A7J7FTN6_CAMSI DUF4216 domain-containing protein OS=Camellia sinensis OX=4442 GN=HYC85_02	camellia sinensis	39947.LOC_Os10g11870.1	[illegible]	[illegible]	[illegible]	[illegible]
Endless_Summer_g29965	Endless_Summer_g29965	Endless Summer genom	Primary assembly	tr|A0A5B6Z8X6|A0A5B6Z8X6_DAVIN	73	tr|A0A5B6Z8X6|A0A5B6Z8X6_DAVIN RanBD1 domain-containing protein OS=Davidia involucrata OX=16924 GN=Din_009	davidia involucrata	29760.VIT_04s0008g01410.t01	[illegible]	[illegible]	[illegible]	[illegible]
Endless_Summer_g30068	Endless_Summer_g30068	Endless Summer genom	Primary assembly	XP_012856951.1	67	XP_012856951.1 PREDICTED: uncharacterized protein LOC105976216 [Erythranthe guttata]	erythranthe guttata	3847.GLYMA03G15841.1	Inherit from KOG: Retrotransposon protein	[illegible]	[illegible]	[illegible]
Endless_Summer_g30094	Endless_Summer_g30094	Endless Summer genom	Primary assembly	tr|A0A6V7NQR9|A0A6V7NQR9_ANACO	59.9	tr|A0A6V7NQR9|A0A6V7NQR9_ANACO Integrase catalytic domain-containing protein OS=Ananas comosus var. bracteatus O	ananas comosus var. bracte	39947.LOC_Os03g61660.1		[illegible]	[illegible]	[illegible]
Endless_Summer_g30615	Endless_Summer_g30615	Endless Summer genom	Primary assembly	tr|A0A834L2R4|A0A834L2R4_RHOSS	38.6	tr|A0A834L2R4|A0A834L2R4_RHOSS NAM-associated domain-containing protein OS=Rhododendron simsii OX=118357 GN	rhododendron simsii					
Endless_Summer_g30616	Endless_Summer_g30616	Endless Summer genom	Primary assembly	XP_012851405.1	61	XP_012851405.1 PREDICTED: uncharacterized protein LOC105971104 [Erythranthe guttata]	erythranthe guttata	4577.GRMZM2G568027_P01	[illegible]	[illegible]	[illegible]	[illegible]
Endless_Summer_g30690	Endless_Summer_g30690	Endless Summer genom	Primary assembly	tr|A0A2N9GNN7|A0A2N9GNN7_FAGSY	59.7	tr|A0A2N9GNN7|A0A2N9GNN7_FAGSY Integrase catalytic domain-containing protein OS=Fagus sylvatica OX=28930 GN=F	fagus sylvatica	39947.LOC_Os12g20300.1		[illegible]	[illegible]	[illegible]
Endless_Summer_g30716	Endless_Summer_g30716	Endless Summer genom	Primary assembly	tr|M1AKM4|M1AKM4_SOLTU	45.7	tr|M1AKM4|M1AKM4_SOLTU PMD domain-containing protein OS=Solanum tuberosum OX=4113 PE=4 SV=1	solanum tuberosum	4113.PGSC0003DMT400035683	Conserved gene of	[illegible]	[illegible]	[illegible]

Endless_Summer_g32748	Endless_Summer_g32748	Endless Summer genom	Primary assembly	tr|A0A7N2RDL0|A0A7N2RDL0_QUELO	34.3	tr|A0A7N2RDL0|A0A7N2RDL0_QUELO ATP-dependent DNA helicase OS=Quercus lobata OX=97700 PE=3 SV=1	quercus lobata	39946.BGIOSGA027560-PA	[illegible]	[illegible]
Endless_Summer_g32799	Endless_Summer_g32799	Endless Summer genom	Primary assembly	tr|A0A5R9QK12|A0A5R9QK12_9BACT	64.1	tr|A0A5R9QK12|A0A5R9QK12_9BACT RT_RNaseH_2 domain-containing protein (Fragment) OS=Labilibacter sediminis OX	labilibacter sediminis	39947.LOC_Os01g23330.1		[illegible]
Endless_Summer_g32851	Endless_Summer_g32851	Endless Summer genom	Primary assembly	XP_028065233.1	77.8	XP_028065233.1 probable LRR receptor-like serine/threonine-protein kinase At1g63430 isoform X1 [Camellia sinensis]	camellia sinensis	29760.VIT_02s0025g03700.t01	[illegible]	[illegible]
Endless_Summer_g32907	Endless_Summer_g32907	Endless Summer genom	Primary assembly	tr|A0A2N9FZG7|A0A2N9FZG7_FAGSY	54	tr|A0A2N9FZG7|A0A2N9FZG7_FAGSY Uncharacterized protein OS=Fagus sylvatica OX=28930 GN=FSB_LOCUS20183 PE=	fagus sylvatica	4577.GRMZM2G033054_P01	[illegible]	[illegible]
Endless_Summer_g32929	Endless_Summer_g32929	Endless Summer genom	Primary assembly	XP_030959232.1	32	XP_030959232.1 uncharacterized protein LOC115981206 [Quercus lobata]	quercus lobata			[illegible]
Endless_Summer_g33064	Endless_Summer_g33064	Endless Summer genom	Primary assembly	XP_028099132.1	68.5	XP_028099132.1 BTB/POZ domain-containing protein At3g05675-like isoform X1 [Camellia sinensis]	camellia sinensis	29760.VIT_02s0025g02270.t01	[illegible]	[illegible]
Endless_Summer_g3313	Endless_Summer_g3313	Endless Summer genom	Primary assembly	tr|A0A7J7MRR8|A0A7J7MRR8_9MAGN	46	tr|A0A7J7MRR8|A0A7J7MRR8_9MAGN Uncharacterized protein OS=Kingdonia uniflora OX=39325 GN=GIB67_037212 PE=	kingdonia uniflora	240176.XP_001835316.2	[illegible]	[illegible]
Endless_Summer_g33187	Endless_Summer_g33187	Endless Summer genom	Primary assembly	tr|A0A8C6LHS0|A0A8C6LHS0_NOTFU	34.6	tr|A0A8C6LHS0|A0A8C6LHS0_NOTFU Lanosterol synthase (2,3-oxidosqualene-lanosterol cyclase) OS=Nothobranchius furzeri	nothobranchius furzeri	3847.GLYMA03G27661.1	[illegible]	[illegible]
Endless_Summer_g33266	Endless_Summer_g33266	Endless Summer genom	Primary assembly	tr|A0A5B7B1Y6|A0A5B7B1Y6_DAVIN	81	tr|A0A5B7B1Y6|A0A5B7B1Y6_DAVIN Reverse transcriptase Ty1/copia-type domain-containing protein OS=Davidia involucra	davidia involucrata	4113.PGSC0003DMT400074691	NA	[illegible]
Endless_Summer_g33288	Endless_Summer_g33288	Endless Summer genom	Primary assembly	tr|A0A5B7BH38|A0A5B7BH38_DAVIN	89.1	tr|A0A5B7BH38|A0A5B7BH38_DAVIN Aspartyl-tRNA synthetase OS=Davidia involucrata OX=16924 GN=Din_037600 PE=3	davidia involucrata	29760.VIT_04s0008g03010.t01	Aspartyl-tRNA synthetase	[illegible]
Endless_Summer_g33512	Endless_Summer_g33512	Endless Summer genom	Primary assembly	XP_028069785.1	31.1	XP_028069785.1 uncharacterized protein LOC114272312 [Camellia sinensis]	camellia sinensis	29760.VIT_14s0060g00370.t01	[illegible]	[illegible]
Endless_Summer_g33525	Endless_Summer_g33525	Endless Summer genom	Primary assembly	tr|A0A558GV05|A0A558GV05_9GAMM	44.6	tr|A0A558GV05|A0A558GV05_9GAMM Uncharacterized protein OS=Cobetia crustatorum OX=553385 GN=FQP86_17895 PE	cobetia crustatorum	39947.LOC_Os07g05280.1		[illegible]
Endless_Summer_g33561	Endless_Summer_g33561	Endless Summer genom	Primary assembly	tr|A0A7N2MU28|A0A7N2MU28_QUELO	34.9	tr|A0A7N2MU28|A0A7N2MU28_QUELO Retrotrans_gag domain-containing protein OS=Quercus lobata OX=97700 PE=4 SV=	quercus lobata	39947.LOC_Os09g16370.1		[illegible]
Endless_Summer_g33603	Endless_Summer_g33603	Endless Summer genom	Primary assembly	tr|A0A6S7NB07|A0A6S7NB07_LACSI	47.4	tr|A0A6S7NB07|A0A6S7NB07_LACSI Uncharacterized protein OS=Lactuca saligna OX=75948 GN=LSAL_LOCUS26037 PE=	lactuca saligna	4081.Solyc03g013270.1.1	NA	[illegible]
Endless_Summer_g33730	Endless_Summer_g33730	Endless Summer genom	Primary assembly	XP_021975001.1	44.9	XP_021975001.1 zinc finger BED domain-containing protein RICESLEEPER 2-like [Helianthus annuus]	helianthus annuus	3847.GLYMA14G13178.1	[illegible]	[illegible]
Endless_Summer_g33760	Endless_Summer_g33760	Endless Summer genom	Primary assembly	XP_041025233.1	83.3	XP_041025233.1 TLC domain-containing protein 4-B-like [Juglans microcarpa x Juglans regia]	juglans microcarpa x juglans	4113.PGSC0003DMT400054655	[illegible]	[illegible]
Endless_Summer_g33826	Endless_Summer_g33826	Endless Summer genom	Primary assembly	tr|A0A4Q3EVW2|A0A4Q3EVW2_9SPHI	41.5	tr|A0A4Q3EVW2|A0A4Q3EVW2_9SPHI Transpos_assoc domain-containing protein OS=Sphingobacteriaceae bacterium OX=2	sphingobacteriaceae bacteri	4558.Sb03g026736.1	[illegible]	[illegible]
Endless_Summer_g33838	Endless_Summer_g33838	Endless Summer genom	Primary assembly	tr|A0A2N9H6L3|A0A2N9H6L3_FAGSY	36.6	tr|A0A2N9H6L3|A0A2N9H6L3_FAGSY Retrotrans_gag domain-containing protein OS=Fagus sylvatica OX=28930 GN=FSB	fagus sylvatica	4558.Sb03g023051.1	[illegible]	[illegible]
Endless_Summer_g33852	Endless_Summer_g33852	Endless Summer genom	Primary assembly	XP_028090420.1	31.6	XP_028090420.1 replication protein A 70 kDa DNA-binding subunit B-like [Camellia sinensis]	camellia sinensis	3847.GLYMA17G08660.1	[illegible]	[illegible]
Endless_Summer_g34097	Endless_Summer_g34097	Endless Summer genom	Primary assembly	XP_028061657.1	31.3	XP_028061657.1 putative lysozyme-like protein [Camellia sinensis]	camellia sinensis			[illegible]
Endless_Summer_g34190	Endless_Summer_g34190	Endless Summer genom	Primary assembly	tr|A0A6I1DV38|A0A6I1DV38_9FLAO	47.1	tr|A0A6I1DV38|A0A6I1DV38_9FLAO DDE-type integrase/transposase/recombinase (Fragment) OS=Muricauda olearia OX=552	muricauda olearia	4113.PGSC0003DMT400056816	[illegible]	[illegible]
Endless_Summer_g3436	Endless_Summer_g3436	Endless Summer genom	Primary assembly	tr|A0A5J5AUX5|A0A5J5AUX5_9ASTE	73	tr|A0A5J5AUX5|A0A5J5AUX5_9ASTE Protein kinase domain-containing protein OS=Nyssa sinensis OX=561372 GN=F0562	nyssa sinensis	29760.VIT_08s0007g02110.t01	[illegible]	[illegible]
Endless_Summer_g3440	Endless_Summer_g3440	Endless Summer genom	Primary assembly	XP_031253748.1	77	XP_031253748.1 LOW QUALITY PROTEIN: probable LRR receptor-like serine/threonine-protein kinase At3g47570 [Pistacia v	pistacia vera	29760.VIT_09s0018g01080.t01		[illegible]
Endless_Summer_g34467	Endless_Summer_g34467	Endless Summer genom	Primary assembly	XP_038699222.1	43.9	XP_038699222.1 zinc finger MYM-type protein 1-like isoform X1 [Tripterygium wilfordii]	tripterygium wilfordii	3694.POPTR_0019s05600.1	[illegible]	[illegible]
Endless_Summer_g3448	Endless_Summer_g3448	Endless Summer genom	Primary assembly	tr|B6D3Q7|B6D3Q7_PLAAC	63.9	tr|B6D3Q7|B6D3Q7_PLAAC Kinase-like protein pac.Erf10 (Fragment) OS=Platanus acerifolia OX=140101 PE=4 SV=1	platanus acerifolia	3694.POPTR_0011s10410.1	[illegible]	[illegible]
[illegible]	[illegible]	[illegible]	[illegible]	[illegible]	[illegible]	[illegible]	[illegible]	[illegible]	[illegible]	[illegible]

Endless_Summer_g5461	Endless_Summer_g5461	Endless Summer genom	Primary assembly	tr|A0A5B7CA58|A0A5B7CA58_DAVIN	71.2	tr|A0A5B7CA58|A0A5B7CA58 DAVIN Uncharacterized protein OS=Davidia involucrata OX=16924 GN=Din 047241 PE=4 S…	davidia involucrata
Endless_Summer_g5479	Endless_Summer_g5479	Endless Summer genom	Primary assembly	XP_018839063.2	35.2	XP_018839063.2 uncharacterized protein LOC109004818 [Juglans regia]	juglans regia
Endless_Summer_g54848	Endless_Summer_g54848	Endless Summer genom	Primary assembly	tr|A0A2N9GKQ3|A0A2N9GKQ3_FAGSY	58.8	tr|A0A2N9GKQ3|A0A2N9GKQ3 FAGSY Integrase catalytic domain-containing protein OS=Fagus sylvatica OX=28930 GN=F…	fagus sylvatica
Endless_Summer_g55097	Endless_Summer_g55097	Endless Summer genom	Primary assembly	tr|A0A4S4D7Z4|A0A4S4D7Z4_CAMSI	63.7	tr|A0A4S4D7Z4|A0A4S4D7Z4 CAMSI Receptor-like serine/threonine-protein kinase OS=Camellia sinensis var. sinensis OX=5…	camellia sinensis var. sinen…
Endless_Summer_g55320	Endless_Summer_g55320	Endless Summer genom	Primary assembly	XP_028100526.1	81.8	XP_028100526.1 LOW QUALITY PROTEIN: protein JINGUBANG-like [Camellia sinensis]	camellia sinensis
Endless_Summer_g55321	Endless_Summer_g55321	Endless Summer genom	Primary assembly	tr|M1D7E8|M1D7E8_SOLTU	41	tr|M1D7E8|M1D7E8_SOLTU Uncharacterized protein OS=Solanum tuberosum OX=4113 PE=4 SV=1	solanum tuberosum
Endless_Summer_g55458	Endless_Summer_g55458	Endless Summer genom	Primary assembly	XP_027067908.1	30.3	XP_027067908.1 uncharacterized protein LOC113693571 [Coffea arabica]	coffea arabica
Endless_Summer_g55553	Endless_Summer_g55553	Endless Summer genom	Primary assembly	XP_015879387.1	37.8	XP_015879387.1 uncharacterized protein LOC107415557 [Ziziphus jujuba]	ziziphus jujuba
Endless_Summer_g55705	Endless_Summer_g55705	Endless Summer genom	Primary assembly	tr|A0A5B6YUU1|A0A5B6YUU1_DAVIN	45	tr|A0A5B6YUU1|A0A5B6YUU1 DAVIN Uncharacterized protein OS=Davidia involucrata OX=16924 GN=Din 005031 PE=4 S…	davidia involucrata
Endless_Summer_g5574	Endless_Summer_g5574	Endless Summer genom	Primary assembly	XP_027115702.1	30.5	XP_027115702.1 uncharacterized protein LOC113733712 [Coffea arabica]	coffea arabica
Endless_Summer_g56051	Endless_Summer_g56051	Endless Summer genom	Primary assembly	tr|A0A7N0TY65|A0A7N0TY65_KALFE	90.2	tr|A0A7N0TY65|A0A7N0TY65_KALFE Uncharacterized protein OS=Kalanchoe fedtschenkoi OX=63787 PE=4 SV=1	kalanchoe fedtschenkoi
Endless_Summer_g56246	Endless_Summer_g56246	Endless Summer genom	Primary assembly	XP_042950096.1	44.4	XP_042950096.1 uncharacterized protein LOC122282211 [Carya illinoinensis]	carya illinoinensis
Endless_Summer_g5625	Endless_Summer_g5625	Endless Summer genom	Primary assembly	tr|A0A5B6ZH35|A0A5B6ZH35_DAVIN	82.8	tr|A0A5B6ZH35|A0A5B6ZH35 DAVIN Putative programmed cell death protein 2 OS=Davidia involucrata OX=16924 GN=Din…	davidia involucrata
Endless_Summer_g56302	Endless_Summer_g56302	Endless Summer genom	Primary assembly	XP_038713392.1	75.4	XP_038713392.1 zinc finger MYM-type protein 1-like [Tripterygium wilfordii]	tripterygium wilfordii
Endless_Summer_g56472	Endless_Summer_g56472	Endless Summer genom	Primary assembly	tr|A0A5B6YZU5|A0A5B6YZU5_DAVIN	57.6	tr|A0A5B6YZU5|A0A5B6YZU5 DAVIN Uncharacterized protein OS=Davidia involucrata OX=16924 GN=Din 006559 PE=4 S…	davidia involucrata
Endless_Summer_g56557	Endless_Summer_g56557	Endless Summer genom	Primary assembly	XP_028122816.1	33.4	XP_028122816.1 uncharacterized protein LOC114319950 [Camellia sinensis]	camellia sinensis
Endless_Summer_g56605	Endless_Summer_g56605	Endless Summer genom	Primary assembly	tr|A0A5B7BGJ0|A0A5B7BGJ0_DAVIN	65	tr|A0A5B7BGJ0|A0A5B7BGJ0 DAVIN DNL-type domain-containing protein (Fragment) OS=Davidia involucrata OX=16924 …	davidia involucrata
Endless_Summer_g56609	Endless_Summer_g56609	Endless Summer genom	Primary assembly	XP_020246343.1	30.6	XP_020246343.1 protein FAR1-RELATED SEQUENCE 5-like [Asparagus officinalis]	asparagus officinalis
Endless_Summer_g56648	Endless_Summer_g56648	Endless Summer genom	Primary assembly	XP_021832886.1	55.2	XP_021832886.1 PKS-NRPS hybrid synthetase CHGG_01239-like [Prunus avium]	prunus avium
Endless_Summer_g56722	Endless_Summer_g56722	Endless Summer genom	Primary assembly	tr|A0A7J7FV04|A0A7J7FV04_CAMSI	30.8	tr|A0A7J7FV04|A0A7J7FV04 CAMSI SWIM-type domain-containing protein OS=Camellia sinensis OX=4442 GN=HYC85 0…	camellia sinensis
Endless_Summer_g56735	Endless_Summer_g56735	Endless Summer genom	Primary assembly	tr|A0A4S4E6A6|A0A4S4E6A6_CAMSI	39.7	tr|A0A4S4E6A6|A0A4S4E6A6 CAMSI NB-ARC domain-containing protein OS=Camellia sinensis var. sinensis OX=542762 …	camellia sinensis var. sinen…
Endless_Summer_g56742	Endless_Summer_g56742	Endless Summer genom	Primary assembly	XP_028063960.1	34.3	XP_028063960.1 putative late blight resistance protein homolog R1A-10 [Camellia sinensis]	camellia sinensis
Endless_Summer_g56755	Endless_Summer_g56755	Endless Summer genom	Primary assembly	tr|A0A5B7AGD2|A0A5B7AGD2_DAVIN	44.1	tr|A0A5B7AGD2|A0A5B7AGD2 DAVIN Uncharacterized protein (Fragment) OS=Davidia involucrata OX=16924 GN=Din 025…	davidia involucrata
Endless_Summer_g56878	Endless_Summer_g56878	Endless Summer genom	Primary assembly	XP_028098604.1	61.3	XP_028098604.1 putative late blight resistance protein homolog R1B-14 isoform X1 [Camellia sinensis]	camellia sinensis
Endless_Summer_g56967	Endless_Summer_g56967	Endless Summer genom	Primary assembly	XP_028051547.1	70.7	XP_028051547.1 receptor protein kinase TMK1-like isoform X2 [Camellia sinensis]	camellia sinensis
Endless_Summer_g57453	Endless_Summer_g57453	Endless Summer genom	Primary assembly	XP_034207926.1	61.1	XP_034207926.1 GDSL esterase/lipase 2-like isoform X2 [Prunus dulcis]	prunus dulcis
Endless_Summer_g57499	Endless_Summer_g57499	Endless Summer genom	Primary assembly	tr|A0A834GDS2|A0A834GDS2_RHOSS	34.8	tr|A0A834GDS2|A0A834GDS2 RHOSS SWIM-type domain-containing protein OS=Rhododendron simsii OX=118357 GN=R…	rhododendron simsii
Endless_Summer_g57523	Endless_Summer_g57523	Endless Summer genom	Primary assembly	tr|A0A4V1FWB2|A0A4V1FWB2_9HYME	41.5	tr|A0A4V1FWB2|A0A4V1FWB2 9HYME Ionotropic receptor (Fragment) OS=Meteorus pulchricornis OX=51522 GN=IR25 P…	meteorus pulchricornis
Endless_Summer_g57540	Endless_Summer_g57540	Endless Summer genom	Primary assembly	tr|A0A834X6T9|A0A834X6T9_9FABA	44.6	tr|A0A834X6T9|A0A834X6T9 9FABA ATP-dependent DNA helicase OS=Senna tora OX=362788 GN=G2W53 007517 PE=3…	senna tora
Endless_Summer_g57748	Endless_Summer_g57748	Endless Summer genom	Primary assembly	tr|A0A835D3X1|A0A835D3X1_9MAGN	34.9	tr|A0A835D3X1|A0A835D3X1 9MAGN Integrase catalytic domain-containing protein OS=Tetracentron sinense OX=13715 GN…	tetracentron sinense
Endless_Summer_g57782	Endless_Summer_g57782	Endless Summer genom	Primary assembly	XP_028097070.1	82.2	XP_028097070.1 mitochondrial import inner membrane translocase subunit TIM23-2-like [Camellia sinensis]	camellia sinensis
Endless_Summer_g57813	Endless_Summer_g57813	Endless Summer genom	Primary assembly	tr|A0A5E4EHM2|A0A5E4EHM2_PRUDU	42.5	tr|A0A5E4EHM2|A0A5E4EHM2 PRUDU RNase H type-1 domain-containing protein (Fragment) OS=Prunus dulcis OX=3755	prunus dulcis
Endless_Summer_g57860	Endless_Summer_g57860	Endless Summer genom	Primary assembly	tr|A0A5B6YP91|A0A5B6YP91_DAVIN	64.6	tr|A0A5B6YP91|A0A5B6YP91 DAVIN PWWP domain-containing protein OS=Davidia involucrata OX=16924 GN=Din 0026…	davidia involucrata
Endless_Summer_g57943	Endless_Summer_g57943	Endless Summer genom	Primary assembly	tr|A0A4U2KHV6|A0A4U2KHV6_9BACT	59.8	tr|A0A4U2KHV6|A0A4U2KHV6 9BACT RNA-directed DNA polymerase (Fragment) OS=Puteibacter caeruleilacunae OX=252…	puteibacter caeruleilacunae
Endless_Summer_g5795	Endless_Summer_g5795	Endless Summer genom	Primary assembly	XP_031123350.1	75.8	XP_031123350.1 L-cysteine desulfhydrase-like [Ipomoea triloba]	ipomoea triloba
Endless_Summer_g58058	Endless_Summer_g58058	Endless Summer genom	Primary assembly	tr|A0A5J5BF50|A0A5J5BF50_9ASTE	77.6	tr|A0A5J5BF50|A0A5J5BF50 9ASTE Hydrolase 4 domain-containing protein OS=Nyssa sinensis OX=561372 GN=F0562 02…	nyssa sinensis
Endless_Summer_g58062	Endless_Summer_g58062	Endless Summer genom	Primary assembly	tr|A0A6P5SK67|A0A6P5SK67_PRUAV	49.9	tr|A0A6P5SK67|A0A6P5SK67 PRUAV Protein FAR1-RELATED SEQUENCE OS=Prunus avium OX=42229 GN=LOC1107…	prunus avium
Endless_Summer_g58252	Endless_Summer_g58252	Endless Summer genom	Primary assembly	tr|A0A2N9FJU1|A0A2N9FJU1_FAGSY	35	tr|A0A2N9FJU1|A0A2N9FJU1 FAGSY ATP-dependent DNA helicase OS=Fagus sylvatica OX=28930 GN=FSB LOCUS1533…	fagus sylvatica
Endless_Summer_g58274	Endless_Summer_g58274	Endless Summer genom	Primary assembly	tr|A0A803Q3H7|A0A803Q3H7_CANSA	66.7	tr|A0A803Q3H7|A0A803Q3H7_CANSA Uncharacterized protein OS=Cannabis sativa OX=3483 PE=4 SV=1	cannabis sativa
Endless_Summer_g58307	Endless_Summer_g58307	Endless Summer genom	Primary assembly	tr|A0A834GZ27|A0A834GZ27_RHOSS	41.6	tr|A0A834GZ27|A0A834GZ27 RHOSS SWIM-type domain-containing protein OS=Rhododendron simsii OX=118357 GN=R…	rhododendron simsii
Endless_Summer_g58320	Endless_Summer_g58320	Endless Summer genom	Primary assembly	tr|A0A438HRK2|A0A438HRK2_VITVI	22.5	tr|A0A438HRK2|A0A438HRK2 VITVI HMG box domain-containing protein OS=Vitis vinifera OX=29760 GN=CK203 04832…	vitis vinifera
Endless_Summer_g58353	Endless_Summer_g58353	Endless Summer genom	Primary assembly	XP_028089536.1	36.3	XP_028089536.1 uncharacterized protein LOC114289901 [Camellia sinensis]	camellia sinensis
Endless_Summer_g58368	Endless_Summer_g58368	Endless Summer genom	Primary assembly	XP_028051092.1	64.3	XP_028051092.1 PX domain-containing protein EREX isoform X1 [Camellia sinensis]	camellia sinensis
Endless_Summer_g5838	Endless_Summer_g5838	Endless Summer genom	Primary assembly	tr|A0A7G3D5Z7|A0A7G3D5Z7_9RHOB	43.5	tr|A0A7G3D5Z7|A0A7G3D5Z7 9RHOB Reverse transcriptase domain-containing protein (Fragment) OS=Carideicomes alvinoc…	carideicomes alvinocaridis
Endless_Summer_g58659	Endless_Summer_g58659	Endless Summer genom	Primary assembly	XP_016736870.2	70.6	XP_016736870.2 mitochondrial import inner membrane translocase subunit PAM16 like 2-like isoform X1 [Gossypium hirsutum…	gossypium hirsutum
Endless_Summer_g58754	Endless_Summer_g58754	Endless Summer genom	Primary assembly	XP_028121464.1	44.4	XP_028121464.1 uncharacterized protein LOC114318721 [Camellia sinensis]	camellia sinensis
Endless_Summer_g59194	Endless_Summer_g59194	Endless Summer genom	Primary assembly	XP_028066453.1	49.4	XP_028066453.1 uncharacterized protein LOC114269342 [Camellia sinensis]	camellia sinensis
Endless_Summer_g59461	Endless_Summer_g59461	Endless Summer genom	Primary assembly	tr|A0A5B7C7Z6|A0A5B7C7Z6_DAVIN	62.9	tr|A0A5B7C7Z6|A0A5B7C7Z6 DAVIN Uncharacterized protein (Fragment) OS=Davidia involucrata OX=16924 GN=Din 0459…	davidia involucrata
Endless_Summer_g59479	Endless_Summer_g59479	Endless Summer genom	Primary assembly	tr|A0A4P1R1V7|A0A4P1R1V7_LUPAN	74.4	tr|A0A4P1R1V7|A0A4P1R1V7 LUPAN Uncharacterized protein OS=Lupinus angustifolius OX=3871 GN=TanjilG 26105 PE=…	lupinus angustifolius
Endless_Summer_g59503	Endless_Summer_g59503	Endless Summer genom	Primary assembly	tr|A0A5B7C259|A0A5B7C259_DAVIN	66.1	tr|A0A5B7C259|A0A5B7C259 DAVIN Bifunctional lysine-specific demethylase and histidyl-hydroxylase OS=Davidia involucr…	davidia involucrata
Endless_Summer_g59537	Endless_Summer_g59537	Endless Summer genom	Primary assembly	XP_028119845.1	46.4	XP_028119845.1 uncharacterized protein LOC114317339 [Camellia sinensis]	camellia sinensis
Endless_Summer_g59574	Endless_Summer_g59574	Endless Summer genom	Primary assembly	tr|A0A5A7U8L3|A0A5A7U8L3_CUCME	44.5	tr|A0A5A7U8L3|A0A5A7U8L3 CUCME PMD domain-containing protein OS=Cucumis melo var. makuwa OX=1194695 GN=…	cucumis melo var. makuwa
Endless_Summer_g59620	Endless_Summer_g59620	Endless Summer genom	Primary assembly	tr|A0A5J5B843|A0A5J5B843_9ASTE	79.2	tr|A0A5J5B843|A0A5J5B843 9ASTE Protein kinase domain-containing protein OS=Nyssa sinensis OX=561372 GN=F0562 0…	nyssa sinensis
Endless_Summer_g59677	Endless_Summer_g59677	Endless Summer genom	Primary assembly	XP_021802190.1	62.1	XP_021802190.1 uncharacterized protein LOC110746292 [Prunus avium]	prunus avium
Endless_Summer_g59710	Endless_Summer_g59710	Endless Summer genom	Primary assembly	tr|A0A6P6U2Y6|A0A6P6U2Y6_COFAR	28.4	tr|A0A6P6U2Y6|A0A6P6U2Y6 COFAR uncharacterized protein LOC113706678 OS=Coffea arabica OX=13443 GN=LOC1137…	coffea arabica
Endless_Summer_g5982	Endless_Summer_g5982	Endless Summer genom	Primary assembly	XP_030964224.1	30.3	XP_030964224.1 uncharacterized protein LOC115985424 [Quercus lobata]	quercus lobata
Endless_Summer_g59923	Endless_Summer_g59923	Endless Summer genom	Primary assembly	XP_022865485.1	34.8	XP_022865485.1 uncharacterized protein LOC111385333, partial [Olea europaea var. sylvestris]	olea europaea var. sylvestri…
Endless_Summer_g59977	Endless_Summer_g59977	Endless Summer genom	Primary assembly	XP_024980415.1	61.3	XP_024980415.1 O-acyltransferase WSD1-like isoform X2 [Cynara cardunculus var. scolymus]	cynara cardunculus var. sc…
Endless_Summer_g60011	Endless_Summer_g60011	Endless Summer genom	Primary assembly	tr|A5B7K8|A5B7K8_VITVI	51.9	tr|A5B7K8|A5B7K8 VITVI DDE Tnp4 domain-containing protein OS=Vitis vinifera OX=29760 GN=VITISV 033852 PE=3 S…	vitis vinifera
Endless_Summer_g60083	Endless_Summer_g60083	Endless Summer genom	Primary assembly	XP_028068740.1	55.4	XP_028068740.1 uncharacterized protein LOC114271323 isoform X1 [Camellia sinensis]	camellia sinensis
Endless_Summer_g60179	Endless_Summer_g60179	Endless Summer genom	Primary assembly	XP_028078847.1	79.8	XP_028078847.1 pentatricopeptide repeat-containing protein At1g31430-like [Camellia sinensis]	camellia sinensis
Endless_Summer_g60265	Endless_Summer_g60265	Endless Summer genom	Primary assembly	tr|A0A6A4MDR5|A0A6A4MDR5_9ERIC	30.8	tr|A0A6A4MDR5|A0A6A4MDR5 9ERIC SWIM-type domain-containing protein (Fragment) OS=Rhododendron williamsianum…	rhododendron williamsianum
Endless_Summer_g60302	Endless_Summer_g60302	Endless Summer genom	Primary assembly	XP_028077328.1	73.2	XP_028077328.1 protein NRT1/ PTR FAMILY 1.1-like [Camellia sinensis]	camellia sinensis
Endless_Summer_g60325	Endless_Summer_g60325	Endless Summer genom	Primary assembly	XP_041007281.1	73.7	XP_041007281.1 pentatricopeptide repeat-containing protein At4g02750-like [Juglans microcarpa x Juglans regia]	juglans microcarpa x juglans…
Endless_Summer_g6039	Endless_Summer_g6039	Endless Summer genom	Primary assembly	tr|A0A6A1UZK3|A0A6A1UZK3_9ROSI	49.4	tr|A0A6A1UZK3|A0A6A1UZK3 9ROSI Uncharacterized protein OS=Morella rubra OX=262757 GN=CJ030 MR7G016721 PE…	morella rubra
Endless_Summer_g60495	Endless_Summer_g60495	Endless Summer genom	Primary assembly	XP_030530104.1	81.8	XP_030530104.1 acyl-CoA-binding protein-like [Rhodamnia argentea]	rhodamnia argentea
Endless_Summer_g60525	Endless_Summer_g60525	Endless Summer genom	Primary assembly	tr|A0A2N9EPZ7|A0A2N9EPZ7_FAGSY	54.5	tr|A0A2N9EPZ7|A0A2N9EPZ7 FAGSY RT RNaseH domain-containing protein OS=Fagus sylvatica OX=28930 GN=FSB L…	fagus sylvatica
Endless_Summer_g60716	Endless_Summer_g60716	Endless Summer genom	Primary assembly	tr|A0A5J5BGX3|A0A5J5BGX3_9ASTE	68.2	tr|A0A5J5BGX3|A0A5J5BGX3 9ASTE START domain-containing protein OS=Nyssa sinensis OX=561372 GN=F0562 0226…	nyssa sinensis
Endless_Summer_g60795	Endless_Summer_g60795	Endless Summer genom	Primary assembly	XP_021300630.1	77.6	XP_021300630.1 protein MEI2-like 2, partial [Herrania umbratica]	herrania umbratica
Endless_Summer_g60830	Endless_Summer_g60830	Endless Summer genom	Primary assembly	XP_015084181.2	31.7	XP_015084181.2 uncharacterized protein LOC107027561 [Solanum pennellii]	solanum pennellii
Endless_Summer_g60967	Endless_Summer_g60967	Endless Summer genom	Primary assembly	XP_018818488.2	50.9	XP_018818488.2 zinc finger BED domain-containing protein RICESLEEPER 2-like [Juglans regia]	juglans regia
Endless_Summer_g6097	Endless_Summer_g6097	Endless Summer genom	Primary assembly	tr|A0A4R5JFQ4|A0A4R5JFQ4_9FIRM	50	tr|A0A4R5JFQ4|A0A4R5JFQ4 9FIRM Uncharacterized protein (Fragment) OS=Zhaonella formicivorans OX=2528593 GN=EY…	zhaonella formicivorans
Endless_Summer_g60996	Endless_Summer_g60996	Endless Summer genom	Primary assembly	tr|A0A6M2E666|A0A6M2E666_9ROSI	35.5	tr|A0A6M2E666|A0A6M2E666 9ROSI RNase H type-1 domain-containing protein OS=Populus davidiana OX=266767 PE=4 S…	populus davidiana
Endless_Summer_g61078	Endless_Summer_g61078	Endless Summer genom	Primary assembly	XP_028094369.1	62.2	XP_028094369.1 uncharacterized protein LOC114294433 [Camellia sinensis]	camellia sinensis
Endless_Summer_g61085	Endless_Summer_g61085	Endless Summer genom	Primary assembly	tr|A0A2N9I5J1|A0A2N9I5J1_FAGSY	63.2	tr|A0A2N9I5J1|A0A2N9I5J1 FAGSY Uncharacterized protein OS=Fagus sylvatica OX=28930 GN=FSB LOCUS47151 PE=4 S…	fagus sylvatica
Endless_Summer_g6111	Endless_Summer_g6111	Endless Summer genom	Primary assembly	XP_030936133.1	29.8	XP_030936133.1 uncharacterized protein LOC115961268 [Quercus lobata]	quercus lobata
Endless_Summer_g61211	Endless_Summer_g61211	Endless Summer genom	Primary assembly	tr|A0A5B6Z857|A0A5B6Z857_DAVIN	33.1	tr|A0A5B6Z857|A0A5B6Z857 DAVIN Retrotrans gag domain-containing protein OS=Davidia involucrata OX=16924 GN=Din…	davidia involucrata
Endless_Summer_g61242	Endless_Summer_g61242	Endless Summer genom	Primary assembly	tr|A0A5B7CE55|A0A5B7CE55_DAVIN	40.4	tr|A0A5B7CE55|A0A5B7CE55 DAVIN Uncharacterized protein (Fragment) OS=Davidia involucrata OX=16924 GN=Din 0481…	davidia involucrata
Endless_Summer_g61248	Endless_Summer_g61248	Endless Summer genom	Primary assembly	XP_028088561.1	72.5	XP_028088561.1 probable carboxylesterase 17 [Camellia sinensis]	camellia sinensis
Endless_Summer_g61341	Endless_Summer_g61341	Endless Summer genom	Primary assembly	XP_034928104.1	64.7	XP_034928104.1 uncharacterized protein LOC118059369 [Populus alba]	populus alba
Endless_Summer_g61460	Endless_Summer_g61460	Endless Summer genom	Primary assembly	tr|A0A438DXJ0|A0A438DXJ0_VITVI	59.6	tr|A0A438DXJ0|A0A438DXJ0 VITVI Transposon Tf2-12 polyprotein OS=Vitis vinifera OX=29760 GN=Tf2-12 20 PE=4 SV=…	vitis vinifera
Endless_Summer_g61535	Endless_Summer_g61535	Endless Summer genom	Primary assembly	tr|A0A7N2R3T7|A0A7N2R3T7_QUELO	38.3	tr|A0A7N2R3T7|A0A7N2R3T7_QUELO Uncharacterized protein OS=Quercus lobata OX=97700 PE=4 SV=1	quercus lobata
Endless_Summer_g61663	Endless_Summer_g61663	Endless Summer genom	Primary assembly	tr|A0A7J6WLI0|A0A7J6WLI0_THATH	27.2	tr|A0A7J6WLI0|A0A7J6WLI0 THATH Aminotransferase-like (Fragment) OS=Thalictrum thalictroides OX=46969 GN=FRX31…	thalictrum thalictroides
Endless_Summer_g61697	Endless_Summer_g61697	Endless Summer genom	Primary assembly	tr|A0A2R6QCJ3|A0A2R6QCJ3_ACTCC	85.5	tr|A0A2R6QCJ3|A0A2R6QCJ3 ACTCC Multiple organellar RNA editing factor 8 like OS=Actinidia chinensis var. chinensis …	actinidia chinensis var. chi…
Endless_Summer_g61703	Endless_Summer_g61703	Endless Summer genom	Primary assembly	tr|A0A6I1DVT3|A0A6I1DVT3_9FLAO	52.3	tr|A0A6I1DVT3|A0A6I1DVT3 9FLAO Uncharacterized protein (Fragment) OS=Muricauda olearia OX=552546 GN=F8C76 17…	muricauda olearia
Endless_Summer_g6175	Endless_Summer_g6175	Endless Summer genom	Primary assembly	tr|A5BX03|A5BX03_VITVI	40.4	tr|A5BX03|A5BX03_VITVI Uncharacterized protein OS=Vitis vinifera OX=29760 GN=VITISV_032357 PE=4 SV=1	vitis vinifera
Endless_Summer_g61814	Endless_Summer_g61814	Endless Summer genom	Primary assembly	tr|M5XMT9|M5XMT9_PRUPE	53.8	tr|M5XMT9|M5XMT9 PRUPE DUF4219 domain-containing protein OS=Prunus persica OX=3760 GN=PRUPE 1G219500 P…	prunus persica
Endless_Summer_g61819	Endless_Summer_g61819	Endless Summer genom	Primary assembly	XP_047265337.1	36.8	XP_047265337.1 uncharacterized protein LOC124897072 [Capsicum annuum]	capsicum annuum
Endless_Summer_g61833	Endless_Summer_g61833	Endless Summer genom	Primary assembly	tr|A0A2N9GB97|A0A2N9GB97_FAGSY	39.3	tr|A0A2N9GB97|A0A2N9GB97 FAGSY CCHC-type domain-containing protein OS=Fagus sylvatica OX=28930 GN=FSB L…	fagus sylvatica
Endless_Summer_g61927	Endless_Summer_g61927	Endless Summer genom	Primary assembly	XP_028072675.1	76.4	XP_028072675.1 uncharacterized protein LOC114274901 [Camellia sinensis]	camellia sinensis
Endless_Summer_g62056	Endless_Summer_g62056	Endless Summer genom	Primary assembly	tr|A0A2N9FUK6|A0A2N9FUK6_FAGSY	58	tr|A0A2N9FUK6|A0A2N9FUK6 FAGSY Integrase catalytic domain-containing protein OS=Fagus sylvatica OX=28930 GN=FS…	fagus sylvatica
Endless_Summer_g62125	Endless_Summer_g62125	Endless Summer genom	Primary assembly	XP_043630442.1	42.7	XP_043630442.1 protein FAR1-RELATED SEQUENCE 8-like [Erigeron canadensis]	erigeron canadensis
Endless_Summer_g62326	Endless_Summer_g62326	Endless Summer genom	Primary assembly	XP_020250852.1	45.5	XP_020250852.1 protein FAR-RED IMPAIRED RESPONSE 1-like [Asparagus officinalis]	asparagus officinalis
Endless_Summer_g62382	Endless_Summer_g62382	Endless Summer genom	Primary assembly	XP_027115703.1	30.2	XP_027115703.1 uncharacterized protein LOC113733712 [Coffea arabica]	coffea arabica
Endless_Summer_g62388	Endless_Summer_g62388	Endless Summer genom	Primary assembly	XP_028056713.1	36.1	XP_028056713.1 putative lysozyme-like protein [Camellia sinensis]	camellia sinensis
Endless_Summer_g62443	Endless_Summer_g62443	Endless Summer genom	Primary assembly	tr|A0A5B7BQH1|A0A5B7BQH1_DAVIN	84.9	tr|A0A5B7BQH1|A0A5B7BQH1 DAVIN Germin-like protein OS=Davidia involucrata OX=16924 GN=Din 040583 PE=3 SV=…	davidia involucrata
Endless_Summer_g62445	Endless_Summer_g62445	Endless Summer genom	Primary assembly	XP_007199217.1	67.3	XP_007199217.1 germin-like protein subfamily 1 member 13 [Prunus persica]	prunus persica
Endless_Summer_g62462	Endless_Summer_g62462	Endless Summer genom	Primary assembly	XP_020595657.1	69	XP_020595657.1 uncharacterized protein LOC110035689 [Phalaenopsis equestris]	phalaenopsis equestris
Endless_Summer_g62604	Endless_Summer_g62604	Endless Summer genom	Primary assembly	tr|A0A6A1VLD9|A0A6A1VLD9_9ROSI	44.1	tr|A0A6A1VLD9|A0A6A1VLD9 9ROSI Reverse transcriptase domain-containing protein OS=Morella rubra OX=262757 GN=C…	morella rubra
Endless_Summer_g62723	Endless_Summer_g62723	Endless Summer genom	Primary assembly	tr|A0A6A1W3K5|A0A6A1W3K5_9ROSI	46.2	tr|A0A6A1W3K5|A0A6A1W3K5 9ROSI Uncharacterized protein OS=Morella rubra OX=262757 GN=CJ030 MR3G009481 PE…	morella rubra
Endless_Summer_g62843	Endless_Summer_g62843	Endless Summer genom	Primary assembly	XP_024190280.1	46.1	XP_024190280.1 uncharacterized protein LOC112194263 [Rosa chinensis]	rosa chinensis
Endless_Summer_g62967	Endless_Summer_g62967	Endless Summer genom	Primary assembly	tr|A0A438HTM2|A0A438HTM2_VITVI	81.9	tr|A0A438HTM2|A0A438HTM2 VITVI Retrovirus-related Pol polyprotein from transposon RE2 OS=Vitis vinifera OX=29760…	vitis vinifera
Endless_Summer_g63002	Endless_Summer_g63002	Endless Summer genom	Primary assembly	tr|M5X4F9|M5X4F9_PRUPE	37.3	tr|M5X4F9|M5X4F9 PRUPE Protein FAR1-RELATED SEQUENCE OS=Prunus persica OX=3760 GN=PRUPE 2G013600 P…	prunus persica
Endless_Summer_g63056	Endless_Summer_g63056	Endless Summer genom	Primary assembly	tr|A0A5B7C5K7|A0A5B7C5K7_DAVIN	37.1	tr|A0A5B7C5K7|A0A5B7C5K7 DAVIN GRF-type domain-containing protein (Fragment) OS=Davidia involucrata OX=16924 …	davidia involucrata
Endless_Summer_g63244	Endless_Summer_g63244	Endless Summer genom	Primary assembly	tr|A0A2N9H6B6|A0A2N9H6B6_FAGSY	49	tr|A0A2N9H6B6|A0A2N9H6B6 FAGSY RNA-directed DNA polymerase OS=Fagus sylvatica OX=28930 GN=FSB LOCUS3…	fagus sylvatica
Endless_Summer_g63359	Endless_Summer_g63359	Endless Summer genom	Primary assembly	tr|A0A834GJW4|A0A834GJW4_RHOSS	39.7	tr|A0A834GJW4|A0A834GJW4 RHOSS Helitron like N domain-containing protein OS=Rhododendron simsii OX=118357 G…	rhododendron simsii
Endless_Summer_g63523	Endless_Summer_g63523	Endless Summer genom	Primary assembly	XP_028071712.1	51.8	XP_028071712.1 ankyrin repeat-containing protein ITN1-like isoform X2 [Camellia sinensis]	camellia sinensis
Endless_Summer_g6354	Endless_Summer_g6354	Endless Summer genom	Primary assembly	tr|A0A6P8CSE9|A0A6P8CSE9_PUNGR	36.9	tr|A0A6P8CSE9|A0A6P8CSE9 PUNGR protein MAINTENANCE OF MERISTEMS-like isoform X1 OS=Punica granatum O…	punica granatum
Endless_Summer_g63563	Endless_Summer_g63563	Endless Summer genom	Primary assembly	XP_028102454.1	37.9	XP_028102454.1 uncharacterized protein LOC114301689 [Camellia sinensis]	camellia sinensis
Endless_Summer_g63568	Endless_Summer_g63568	Endless Summer genom	Primary assembly	tr|A0A2N9GE10|A0A2N9GE10_FAGSY	63.4	tr|A0A2N9GE10|A0A2N9GE10 FAGSY RNA-directed DNA polymerase OS=Fagus sylvatica OX=28930 GN=FSB LOCUS25…	fagus sylvatica
Endless_Summer_g63569	Endless_Summer_g63569	Endless Summer genom	Primary assembly	XP_034701568.1	64.2	XP_034701568.1 leucine-rich repeat extensin-like protein 5 [Vitis riparia]	vitis riparia
Endless_Summer_g63659	Endless_Summer_g63659	Endless Summer genom	Primary assembly	tr|A0A6P9EH03|A0A6P9EH03_JUGRE	21.2	tr|A0A6P9EH03|A0A6P9EH03 JUGRE ATP-dependent DNA helicase OS=Juglans regia OX=51240 GN=LOC118344820 PE=…	juglans regia
Endless_Summer_g63740	Endless_Summer_g63740	Endless Summer genom	Primary assembly	XP_021274018.1	77.6	XP_021274018.1 senescence-specific cysteine protease SAG39-like [Herrania umbratica]	herrania umbratica
Endless_Summer_g63743	Endless_Summer_g63743	Endless Summer genom	Primary assembly	XP_010099380.2	69.5	XP_010099380.2 inactive TPR repeat-containing thioredoxin TTL3 [Morus notabilis]	morus notabilis
Endless_Summer_g63880	Endless_Summer_g63880	Endless Summer genom	Primary assembly	XP_028100178.1	27.6	XP_028100178.1 uncharacterized protein LOC114299601 [Camellia sinensis]	camellia sinensis
Endless_Summer_g63925	Endless_Summer_g63925	Endless Summer genom	Primary assembly	tr|A0A7J0DM16|A0A7J0DM16_9ERIC	41.9	tr|A0A7J0DM16|A0A7J0DM16 9ERIC CCHC-type domain-containing protein OS=Actinidia rufa OX=165716 GN=Acr 00a00…	actinidia rufa
Endless_Summer_g63989	Endless_Summer_g63989	Endless Summer genom	Primary assembly	tr|A0A371G642|A0A371G642_MUCPR	34.1	tr|A0A371G642|A0A371G642 MUCPR G-patch domain-containing protein (Fragment) OS=Mucuna pruriens OX=157652 GN=…	mucuna pruriens
Endless_Summer_g63991	Endless_Summer_g63991	Endless Summer genom	Primary assembly	XP_027069099.1	29.1	XP_027069099.1 uncharacterized protein LOC113694481 [Coffea arabica]	coffea arabica
Endless_Summer_g64065	Endless_Summer_g64065	Endless Summer genom	Primary assembly	tr|A0A2N9FMS3|A0A2N9FMS3_FAGSY	25	tr|A0A2N9FMS3|A0A2N9FMS3 FAGSY Uncharacterized protein OS=Fagus sylvatica OX=28930 GN=FSB LOCUS16111 PE…	fagus sylvatica
Endless_Summer_g64143	Endless_Summer_g64143	Endless Summer genom	Primary assembly	XP_034679629.1	91	XP_034679629.1 uridine kinase-like protein 3 [Vitis riparia]	vitis riparia
Endless_Summer_g64186	Endless_Summer_g64186	Endless Summer genom	Primary assembly	tr|A0A5C7ITY1|A0A5C7ITY1_9ROSI	50.9	tr|A0A5C7ITY1|A0A5C7ITY1 9ROSI Myb DNA-bind 3 domain-containing protein OS=Acer yangbiense OX=1000413 GN=E…	acer yangbiense
Endless_Summer_g64229	Endless_Summer_g64229	Endless Summer genom	Primary assembly	tr|A0A5B7CC92|A0A5B7CC92_DAVIN	81.6	tr|A0A5B7CC92|A0A5B7CC92 DAVIN MTS domain-containing protein OS=Davidia involucrata OX=16924 GN=Din 04741…	davidia involucrata
Endless_Summer_g65203	Endless_Summer_g65203	Endless Summer genom	Primary assembly	tr|A0A4Y8W7I5|A0A4Y8W7I5_9VIBR	38.1	tr|A0A4Y8W7I5|A0A4Y8W7I5 9VIBR Retrotrans gag domain-containing protein (Fragment) OS=Vibrio ouci OX=2499078 G…	vibrio ouci
Endless_Summer_g65409	Endless_Summer_g65409	Endless Summer genom	Primary assembly	tr|A0A392QJU7|A0A392QJU7_9FABA	44.1	tr|A0A392QJU7|A0A392QJU7 9FABA Retrotrans gag domain-containing protein OS=Trifolium medium OX=97028 PE=4 SV…	trifolium medium
Endless_Summer_g65416	Endless_Summer_g65416	Endless Summer genom	Primary assembly	XP_038978932.1	50	XP_038978932.1 protein ALP1-like [Phoenix dactylifera]	phoenix dactylifera
Endless_Summer_g65521	Endless_Summer_g65521	Endless Summer genom	Primary assembly	tr|A0A4S4FGY4|A0A4S4FGY4_9MICO	21.7	tr|A0A4S4FGY4|A0A4S4FGY4 9MICO Reverse transcriptase-like protein (Fragment) OS=Glaciibacter flavus OX=2565934 GN…	glaciibacter flavus
Endless_Summer_g65547	Endless_Summer_g65547	Endless Summer genom	Primary assembly	XP_020272573.1	44.3	XP_020272573.1 protein FAR1-RELATED SEQUENCE 5-like [Asparagus officinalis]	asparagus officinalis
Endless_Summer_g65567	Endless_Summer_g65567	Endless Summer genom	Primary assembly	tr|A0A5J5B7C1|A0A5J5B7C1_9ASTE	55	tr|A0A5J5B7C1|A0A5J5B7C1 9ASTE Uncharacterized protein OS=Nyssa sinensis OX=561372 GN=F0562 025817 PE=4 SV=…	nyssa sinensis
Endless_Summer_g65630	Endless_Summer_g65630	Endless Summer genom	Primary assembly	XP_034678880.1	34.6	XP_034678880.1 uncharacterized protein LOC117909087 [Vitis riparia]	vitis riparia
Endless_Summer_g65701	Endless_Summer_g65701	Endless Summer genom	Primary assembly	tr|A0A834GIU2|A0A834GIU2_RHOSS	82.1	tr|A0A834GIU2|A0A834GIU2 RHOSS Uncharacterized protein OS=Rhododendron simsii OX=118357 GN=RHSIM Rhsim09…	rhododendron simsii
Endless_Summer_g65844	Endless_Summer_g65844	Endless Summer genom	Primary assembly	tr|A0A2N9GY58|A0A2N9GY58_FAGSY	51.5	tr|A0A2N9GY58|A0A2N9GY58 FAGSY Uncharacterized protein OS=Fagus sylvatica OX=28930 GN=FSB LOCUS32181 PE=…	fagus sylvatica
Endless_Summer_g65917	Endless_Summer_g65917	Endless Summer genom	Primary assembly	tr|A0A7J6WHW8|A0A7J6WHW8_THATH	58.9	tr|A0A7J6WHW8|A0A7J6WHW8 THATH Retrovirus-related pol polyprotein from transposon OS=Thalictrum thalictroides O…	thalictrum thalictroides
Endless_Summer_g66025	Endless_Summer_g66025	Endless Summer genom	Primary assembly	XP_010647437.1	57	XP_010647437.1 PREDICTED: uncharacterized protein LOC100259457 isoform X1 [Vitis vinifera]	vitis vinifera
Endless_Summer_g66054	Endless_Summer_g66054	Endless Summer genom	Primary assembly	tr|A0A2N9GS10|A0A2N9GS10_FAGSY	36	tr|A0A2N9GS10|A0A2N9GS10 FAGSY Integrase catalytic domain-containing protein OS=Fagus sylvatica OX=28930 GN=FS…	fagus sylvatica
Endless_Summer_g66092	Endless_Summer_g66092	Endless Summer genom	Primary assembly	XP_019074400.1	38.5	XP_019074400.1 PREDICTED: uncharacterized protein LOC109122316 [Vitis vinifera]	vitis vinifera
Endless_Summer_g66156	Endless_Summer_g66156	Endless Summer genom	Primary assembly	XP_024993455.1	72.5	XP_024993455.1 disease resistance protein RPP13-like [Cynara cardunculus var. scolymus]	cynara cardunculus var. sc…
Endless_Summer_g66157	Endless_Summer_g66157	Endless Summer genom	Primary assembly	XP_024993454.1	63.7	XP_024993454.1 disease resistance protein RPP13-like [Cynara cardunculus var. scolymus]	cynara cardunculus var. sc…
Endless_Summer_g66187	Endless_Summer_g66187	Endless Summer genom	Primary assembly	tr|A0A834LB87|A0A834LB87_RHOSS	28.5	tr|A0A834LB87|A0A834LB87 RHOSS SWIM-type domain-containing protein OS=Rhododendron simsii OX=118357 GN=R…	rhododendron simsii
Endless_Summer_g66189	Endless_Summer_g66189	Endless Summer genom	Primary assembly	XP_028094042.1	34.8	XP_028094042.1 uncharacterized protein LOC114294121 [Camellia sinensis]	camellia sinensis
Endless_Summer_g66200	Endless_Summer_g66200	Endless Summer genom	Primary assembly	tr|A0A438GFQ3|A0A438GFQ3_VITVI	55.2	tr|A0A438GFQ3|A0A438GFQ3 VITVI Transposon Ty3-I Gag-Pol polyprotein OS=Vitis vinifera OX=29760 GN=TY3B-I 182…	vitis vinifera
Endless_Summer_g66218	Endless_Summer_g66218	Endless Summer genom	Primary assembly	tr|A0A2Z7CW89|A0A2Z7CW89_9LAMI	42.1	tr|A0A2Z7CW89|A0A2Z7CW89 9LAMI Retrotran gag 3 domain-containing protein OS=Dorcoceras hygrometricum OX=4723…	dorcoceras hygrometricum
Endless_Summer_g66238	Endless_Summer_g66238	Endless Summer genom	Primary assembly	XP_020245144.1	43.7	XP_020245144.1 uncharacterized protein LOC109823270 [Asparagus officinalis]	asparagus officinalis
Endless_Summer_g66429	Endless_Summer_g66429	Endless Summer genom	Primary assembly	XP_012834531.1	63.9	XP_012834531.1 PREDICTED: uncharacterized protein LOC105955361 [Erythranthe guttata]	erythranthe guttata
Endless_Summer_g66614	Endless_Summer_g66614	Endless Summer genom	Primary assembly	tr|A0A5J4ZYY2|A0A5J4ZYY2_9ASTE	55.6	tr|A0A5J4ZYY2|A0A5J4ZYY2 9ASTE Uncharacterized protein OS=Nyssa sinensis OX=561372 GN=F0562 010514 PE=4 SV…	nyssa sinensis
Endless_Summer_g66654	Endless_Summer_g66654	Endless Summer genom	Primary assembly	tr|A0A5B0NZB7|A0A5B0NZB7_PUCGR	37.1	tr|A0A5B0NZB7|A0A5B0NZB7 PUCGR Uncharacterized protein OS=Puccinia graminis f. sp. tritici OX=56615 GN=PGT21 …	puccinia graminis f. sp. tri…
Endless_Summer_g66845	Endless_Summer_g66845	Endless Summer genom	Primary assembly	tr|A0A5B7CBN0|A0A5B7CBN0_DAVIN	64.6	tr|A0A5B7CBN0|A0A5B7CBN0 DAVIN Uncharacterized protein (Fragment) OS=Davidia involucrata OX=16924 GN=Din 048…	davidia involucrata
Endless_Summer_g6692	Endless_Summer_g6692	Endless Summer genom	Primary assembly	XP_028123698.1	26.5	XP_028123698.1 uncharacterized protein LOC114320834 [Camellia sinensis]	camellia sinensis
Endless_Summer_g66973	Endless_Summer_g66973	Endless Summer genom	Primary assembly	tr|A0A4R5JGA9|A0A4R5JGA9_9FIRM	53.8	tr|A0A4R5JGA9|A0A4R5JGA9 9FIRM RNA-directed DNA polymerase OS=Zhaonella formicivorans OX=2528593 GN=EYS1…	zhaonella formicivorans
Endless_Summer_g67005	Endless_Summer_g67005	Endless Summer genom	Primary assembly	XP_027101280.1	29.5	XP_027101280.1 uncharacterized protein LOC113720813 [Coffea arabica]	coffea arabica
Endless_Summer_g67031	Endless_Summer_g67031	Endless Summer genom	Primary assembly	XP_012851642.1	57.5	XP_012851642.1 PREDICTED: uncharacterized protein LOC105971340 [Erythranthe guttata]	erythranthe guttata
Endless_Summer_g67094	Endless_Summer_g67094	Endless Summer genom	Primary assembly	tr|A0A835LPT4|A0A835LPT4_9MAGN	44.8	tr|A0A835LPT4|A0A835LPT4 9MAGN Retrotrans gag domain-containing protein OS=Coptis chinensis OX=261450 GN=IFN…	coptis chinensis
Endless_Summer_g67131	Endless_Summer_g67131	Endless Summer genom	Primary assembly	tr|A0A3S3PJT7|A0A3S3PJT7_9MAGN	30.4	tr|A0A3S3PJT7|A0A3S3PJT7 9MAGN Bromodomain-containing protein OS=Cinnamomum micranthum f. kanehirae OX=337…	cinnamomum micranthum f.
Endless_Summer_g67145	Endless_Summer_g67145	Endless Summer genom	Primary assembly	XP_022011580.1	60.3	XP_022011580.1 cytosolic sulfotransferase 12 [Helianthus annuus]	helianthus annuus
Endless_Summer_g67173	Endless_Summer_g67173	Endless Summer genom	Primary assembly	XP_028104015.1	55.5	XP_028104015.1 receptor-like protein kinase FERONIA [Camellia sinensis]	camellia sinensis
Endless_Summer_g6729	Endless_Summer_g6729	Endless Summer genom	Primary assembly	tr|A0A7J6UG71|A0A7J6UG71_PEROL	48.9	tr|A0A7J6UG71|A0A7J6UG71 PEROL RT RNaseH 2 domain-containing protein (Fragment) OS=Perkinsus olseni OX=32597…	perkinsus olseni
Endless_Summer_g67303	Endless_Summer_g67303	Endless Summer genom	Primary assembly	tr|A0A5B7BF47|A0A5B7BF47_DAVIN	83.2	tr|A0A5B7BF47|A0A5B7BF47 DAVIN gag pre-integrs domain-containing protein (Fragment) OS=Davidia involucrata OX=16…	davidia involucrata
Endless_Summer_g67356	Endless_Summer_g67356	Endless Summer genom	Primary assembly	tr|A0A2N9IH25|A0A2N9IH25_FAGSY	38.6	tr|A0A2N9IH25|A0A2N9IH25 FAGSY RNA-directed DNA polymerase OS=Fagus sylvatica OX=28930 GN=FSB LOCUS529…	fagus sylvatica
Endless_Summer_g67604	Endless_Summer_g67604	Endless Summer genom	Primary assembly	tr|A0A835DLY7|A0A835DLY7_9MAGN	54.7	tr|A0A835DLY7|A0A835DLY7 9MAGN DUF4220 domain-containing protein OS=Tetracentron sinense OX=13715 GN=HHK…	tetracentron sinense
Endless_Summer_g67637	Endless_Summer_g67637	Endless Summer genom	Primary assembly	tr|A0A7N2KWL9|A0A7N2KWL9_QUELO	53.2	tr|A0A7N2KWL9|A0A7N2KWL9_QUELO RNA-directed DNA polymerase OS=Quercus lobata OX=97700 PE=4 SV=1	quercus lobata
Endless_Summer_g67681	Endless_Summer_g67681	Endless Summer genom	Primary assembly	tr|A0A7J0HCS5|A0A7J0HCS5_9ERIC	25.2	tr|A0A7J0HCS5|A0A7J0HCS5 9ERIC DUF4283 domain-containing protein OS=Actinidia rufa OX=165716 GN=Acr 28g0014…	actinidia rufa
Endless_Summer_g67693	Endless_Summer_g67693	Endless Summer genom	Primary assembly	XP_028103562.1	60.7	XP_028103562.1 uncharacterized protein LOC114302697 [Camellia sinensis]	camellia sinensis
Endless_Summer_g67769	Endless_Summer_g67769	Endless Summer genom	Primary assembly	XP_022028404.1	53.9	XP_022028404.1 zinc finger BED domain-containing protein RICESLEEPER 2 [Helianthus annuus]	helianthus annuus
Endless_Summer_g6782	Endless_Summer_g6782	Endless Summer genom	Primary assembly	tr|A0A6D2K662|A0A6D2K662_9BRAS	84	tr|A0A6D2K662|A0A6D2K662 9BRAS RT RNaseH domain-containing protein OS=Microthlaspi erraticum OX=1685480 GN=…	microthlaspi erraticum
Endless_Summer_g67845	Endless_Summer_g67845	Endless Summer genom	Primary assembly	XP_028099684.1	79.9	XP_028099684.1 probable nucleoredoxin 1 [Camellia sinensis]	camellia sinensis
Endless_Summer_g682	Endless_Summer_g682	Endless Summer genom	Primary assembly	tr|A0A2N9EV59|A0A2N9EV59_FAGSY	33.9	tr|A0A2N9EV59|A0A2N9EV59 FAGSY BED-type domain-containing protein OS=Fagus sylvatica OX=28930 GN=FSB LOC…	fagus sylvatica
Endless_Summer_g68488	Endless_Summer_g68488	Endless Summer genom	Primary assembly	tr|A0A2P6RRJ7|A0A2P6RRJ7_ROSCH	73.4	tr|A0A2P6RRJ7|A0A2P6RRJ7 ROSCH Putative nucleotidyltransferase, Ribonuclease H OS=Rosa chinensis OX=74649 GN=R…	rosa chinensis
Endless_Summer_g68667	Endless_Summer_g68667	Endless Summer genom	Primary assembly	XP_028112672.1	84.1	XP_028112672.1 probable DNA helicase MCM8 [Camellia sinensis]	camellia sinensis
Endless_Summer_g68904	Endless_Summer_g68904	Endless Summer genom	Primary assembly	XP_011028660.1	70.4	XP_011028660.1 PREDICTED: uncharacterized protein LOC105128613 [Populus euphratica]	populus euphratica
Endless_Summer_g68930	Endless_Summer_g68930	Endless Summer genom	Primary assembly	tr|A0A4R4EEA7|A0A4R4EEA7_9GAMM	64.8	tr|A0A4R4EEA7|A0A4R4EEA7 9GAMM Transposase (Fragment) OS=Lysobacter sp. N42 OX=2545719 GN=EYQ95 26200 F…	lysobacter sp. n42
Endless_Summer_g68933	Endless_Summer_g68933	Endless Summer genom	Primary assembly	tr|A0A4S4ECE2|A0A4S4ECE2_CAMSI	45.2	tr|A0A4S4ECE2|A0A4S4ECE2 CAMSI Receptor-like serine/threonine-protein kinase OS=Camellia sinensis var. sinensis OX=5…	camellia sinensis var. sinen…
Endless_Summer_g69017	Endless_Summer_g69017	Endless Summer genom	Primary assembly	tr|A0A4R5JHI4|A0A4R5JHI4_9FIRM	26.9	tr|A0A4R5JHI4|A0A4R5JHI4 9FIRM Retrotrans gag domain-containing protein (Fragment) OS=Zhaonella formicivorans OX=2…	zhaonella formicivorans
Endless_Summer_g69022	Endless_Summer_g69022	Endless Summer genom	Primary assembly	XP_028058335.1	36.9	XP_028058335.1 uncharacterized protein LOC114262178 [Camellia sinensis]	camellia sinensis
Endless_Summer_g69023	Endless_Summer_g69023	Endless Summer genom	Primary assembly	tr|A0A5B7BMT7|A0A5B7BMT7_DAVIN	59.7	tr|A0A5B7BMT7|A0A5B7BMT7 DAVIN Putative mitogen-activated protein kinase-binding protein 1 isoform X2 (Fragment) …	davidia involucrata
Endless_Summer_g69140	Endless_Summer_g69140	Endless Summer genom	Primary assembly	tr|A0A5B7B3V2|A0A5B7B3V2_DAVIN	77.3	tr|A0A5B7B3V2|A0A5B7B3V2 DAVIN Uncharacterized protein (Fragment) OS=Davidia involucrata OX=16924 GN=Din 0324…	davidia involucrata
Endless_Summer_g69180	Endless_Summer_g69180	Endless Summer genom	Primary assembly	tr|A0A5J5BD06|A0A5J5BD06_9ASTE	66.7	tr|A0A5J5BD06|A0A5J5BD06 9ASTE BED-type domain-containing protein OS=Nyssa sinensis OX=561372 GN=F0562 0257…	nyssa sinensis
Endless_Summer_g69271	Endless_Summer_g69271	Endless Summer genom	Primary assembly	XP_028085070.1	57.7	XP_028085070.1 uncharacterized protein LOC114286174 [Camellia sinensis]	camellia sinensis
Endless_Summer_g69425	Endless_Summer_g69425	Endless Summer genom	Primary assembly	tr|A0A2K3PB23|A0A2K3PB23_TRIPR	42.7	tr|A0A2K3PB23|A0A2K3PB23 TRIPR Retroelement pol polyprotein-like OS=Trifolium pratense OX=57577 GN=L195 g0091…	trifolium pratense
Endless_Summer_g69748	Endless_Summer_g69748	Endless Summer genom	Primary assembly	XP_020423126.1	41.9	XP_020423126.1 uncharacterized protein LOC109950034 [Prunus persica]	prunus persica
Endless_Summer_g69768	Endless_Summer_g69768	Endless Summer genom	Primary assembly	XP_034708677.1	64.8	XP_034708677.1 putative UDP-rhamnose:rhamnosyltransferase 1 [Vitis riparia]	vitis riparia
Endless_Summer_g69846	Endless_Summer_g69846	Endless Summer genom	Primary assembly	XP_030478412.1	42.6	XP_030478412.1 LOW QUALITY PROTEIN: zinc finger MYM-type protein 1-like [Cannabis sativa]	cannabis sativa
Endless_Summer_g70034	Endless_Summer_g70034	Endless Summer genom	Primary assembly	tr|A0A834GWK5|A0A834GWK5_RHOSS	64	tr|A0A834GWK5|A0A834GWK5 RHOSS Uncharacterized protein OS=Rhododendron simsii OX=118357 GN=RHSIM Rhsim…	rhododendron simsii
Endless_Summer_g70062	Endless_Summer_g70062	Endless Summer genom	Primary assembly	XP_020080379.1	41.9	XP_020080379.1 uncharacterized protein LOC109704055 [Ananas comosus]	ananas comosus
Endless_Summer_g70099	Endless_Summer_g70099	Endless Summer genom	Primary assembly	tr|A0A493TWA4|A0A493TWA4_ANAPP	60.9	tr|A0A493TWA4|A0A493TWA4 ANAPP Integrase catalytic domain-containing protein OS=Anas platyrhynchos platyrhynchos…	anas platyrhynchos platyrh…
Endless_Summer_g70107	Endless_Summer_g70107	Endless Summer genom	Primary assembly	XP_028095187.1	44.6	XP_028095187.1 uncharacterized protein LOC114295190 [Camellia sinensis]	camellia sinensis
Endless_Summer_g70143	Endless_Summer_g70143	Endless Summer genom	Primary assembly	tr|A0A4D6FU75|A0A4D6FU75_ACTCH	90.5	tr|A0A4D6FU75|A0A4D6FU75 ACTCH DNA polymerase OS=Actinidia chinensis OX=3625 GN=Ac DNA pol B PE=3 SV=…	actinidia chinensis
Endless_Summer_g70210	Endless_Summer_g70210	Endless Summer genom	Primary assembly	tr|B9T8M9|B9T8M9_RICCO	78.1	tr|B9T8M9|B9T8M9_RICCO F-ATPase protein 6 OS=Ricinus communis OX=3988 GN=RCOM_1988470 PE=3 SV=1	ricinus communis
Endless_Summer_g70234	Endless_Summer_g70234	Endless Summer genom	Primary assembly	YP_009414428.1	98.8	YP_009414428.1 ribosomal protein L10 (mitochondrion) [Lagerstroemia indica]	lagerstroemia indica
Endless_Summer_g70235	Endless_Summer_g70235	Endless Summer genom	Primary assembly	YP_010352659.1	68.2	YP_010352659.1 hypothetical protein LK193_mgp25 (mitochondrion) [Bidens tripartita]	bidens tripartita
Endless_Summer_g70279	Endless_Summer_g70279	Endless Summer genom	Primary assembly	YP_009706783.1	88.9	YP_009706783.1 ATPase subunit 4 (mitochondrion) [Ilex pubescens]	ilex pubescens
Endless_Summer_g70443	Endless_Summer_g70443	Endless Summer genom	Primary assembly	tr|A0A7J0ELV6|A0A7J0ELV6_9ERIC	96.1	tr|A0A7J0ELV6|A0A7J0ELV6 9ERIC Uncharacterized protein OS=Actinidia rufa OX=165716 GN=Acr 05g0010190 PE=4 SV=…	actinidia rufa
Endless_Summer_g70445	Endless_Summer_g70445	Endless Summer genom	Primary assembly	YP_009706805.1	96.9	YP_009706805.1 maturase (mitochondrion) [Ilex pubescens]	ilex pubescens
Endless_Summer_g70485	Endless_Summer_g70485	Endless Summer genom	Primary assembly	YP_009526555.1	88.2	YP_009526555.1 ribosomal protein S3 (mitochondrion) [Ammopiptanthus mongolicus]	ammopiptanthus mongolicu…
Endless_Summer_g70528	Endless_Summer_g70528	Endless Summer genom	Primary assembly	tr|A0A7J0D711|A0A7J0D711_9ERIC	69.7	tr|A0A7J0D711|A0A7J0D711 9ERIC Uncharacterized protein OS=Actinidia rufa OX=165716 GN=Acr 00g0003000 PE=4 SV=…	actinidia rufa
Endless_Summer_g70531	Endless_Summer_g70531	Endless Summer genom	Primary assembly	tr|A0A6J5U6Y6|A0A6J5U6Y6_PRUAR	97.8	tr|A0A6J5U6Y6|A0A6J5U6Y6 PRUAR Uncharacterized protein OS=Prunus armeniaca OX=36596 GN=CURHAP LOCUS186…	prunus armeniaca
Endless_Summer_g70535	Endless_Summer_g70535	Endless Summer genom	Primary assembly	tr|A0A834TIA0|A0A834TIA0_9FABA	54.3	tr|A0A834TIA0|A0A834TIA0 9FABA Retrovirus-related Pol polyprotein from transposon TNT 1-94 OS=Senna tora OX=3627…	senna tora
Endless_Summer_g70554	Endless_Summer_g70554	Endless Summer genom	Primary assembly	XP_037474138.1	78.3	XP_037474138.1 NADH-ubiquinone oxidoreductase chain 4-like [Triticum dicoccoides]	triticum dicoccoides
Endless_Summer_g70662	Endless_Summer_g70662	Endless Summer genom	Primary assembly	XP_038685500.1	70.5	XP_038685500.1 LOW QUALITY PROTEIN: receptor-like protein EIX1 [Tripterygium wilfordii]	tripterygium wilfordii
Endless_Summer_g70681	Endless_Summer_g70681	Endless Summer genom	Primary assembly	XP_028106143.1	45.7	XP_028106143.1 putative nuclease HARBI1 [Camellia sinensis]	camellia sinensis
Endless_Summer_g7074	Endless_Summer_g7074	Endless Summer genom	Primary assembly	XP_028126745.1	47.4	XP_028126745.1 uncharacterized protein LOC114323364 [Camellia sinensis]	camellia sinensis
Endless_Summer_g70763	Endless_Summer_g70763	Endless Summer genom	Primary assembly	XP_028097994.1	34.8	XP_028097994.1 uncharacterized protein LOC114297725 isoform X4 [Camellia sinensis]	camellia sinensis
Endless_Summer_g70844	Endless_Summer_g70844	Endless Summer genom	Primary assembly	XP_030933876.1	49.9	XP_030933876.1 zinc finger BED domain-containing protein RICESLEEPER 2-like [Quercus lobata]	quercus lobata
Endless_Summer_g70861	Endless_Summer_g70861	Endless Summer genom	Primary assembly	XP_012859012.1	69.6	XP_012859012.1 PREDICTED: uncharacterized protein LOC105978141 [Erythranthe guttata]	erythranthe guttata
Endless_Summer_g70949	Endless_Summer_g70949	Endless Summer genom	Primary assembly	tr|M5WCA3|M5WCA3_PRUPE	29.7	tr|M5WCA3|M5WCA3 PRUPE Uncharacterized protein OS=Prunus persica OX=3760 GN=PRUPE ppa017281mg PE=3 SV=…	prunus persica
Endless_Summer_g70968	Endless_Summer_g70968	Endless Summer genom	Primary assembly	tr|A0A7J7NSP2|A0A7J7NSP2_9MAGN	30.6	tr|A0A7J7NSP2|A0A7J7NSP2 9MAGN PMD domain-containing protein OS=Kingdonia uniflora OX=39325 GN=GIB67 0387…	kingdonia uniflora
Endless_Summer_g71039	Endless_Summer_g71039	Endless Summer genom	Primary assembly	XP_006448611.2	61.6	XP_006448611.2 UDP-glycosyltransferase 43 [Citrus clementina]	citrus clementina
Endless_Summer_g71348	Endless_Summer_g71348	Endless Summer genom	Primary assembly	tr|A0A834H3K2|A0A834H3K2_RHOSS	41.5	tr|A0A834H3K2|A0A834H3K2 RHOSS SWIM-type domain-containing protein OS=Rhododendron simsii OX=118357 GN=R…	rhododendron simsii
Endless_Summer_g71359	Endless_Summer_g71359	Endless Summer genom	Primary assembly	tr|A0A6J5WP26|A0A6J5WP26_PRUAR	43.3	tr|A0A6J5WP26|A0A6J5WP26 PRUAR Retrotran gag 3 domain-containing protein OS=Prunus armeniaca OX=36596 GN=O…	prunus armeniaca
Endless_Summer_g71525	Endless_Summer_g71525	Endless Summer genom	Primary assembly	XP_019250891.1	57	XP_019250891.1 PREDICTED: uncharacterized protein LOC109229793 [Nicotiana attenuata]	nicotiana attenuata
Endless_Summer_g7170	Endless_Summer_g7170	Endless Summer genom	Primary assembly	tr|A0A5C6WZP8|A0A5C6WZP8_9DELT	36.1	tr|A0A5C6WZP8|A0A5C6WZP8 9DELT Uncharacterized protein OS=Bradymonadales bacterium TMO4 OX=2600176 GN=F…	bradymonadales bacterium
Endless_Summer_g71789	Endless_Summer_g71789	Endless Summer genom	Primary assembly	XP_028106461.1	91.5	XP_028106461.1 methionine aminopeptidase 1A [Camellia sinensis]	camellia sinensis
Endless_Summer_g71798	Endless_Summer_g71798	Endless Summer genom	Primary assembly	XP_021748948.1	50	XP_021748948.1 protein ALP1-like isoform X2 [Chenopodium quinoa]	chenopodium quinoa
Endless_Summer_g71804	Endless_Summer_g71804	Endless Summer genom	Primary assembly	tr|A0A834YK84|A0A834YK84_9MAGN	78.9	tr|A0A834YK84|A0A834YK84 9MAGN ECH 2 domain-containing protein OS=Tetracentron sinense OX=13715 GN=HHK36	tetracentron sinense
Endless_Summer_g71870	Endless_Summer_g71870	Endless Summer genom	Primary assembly	XP_042980333.1	52.9	XP_042980333.1 zinc finger BED domain-containing protein RICESLEEPER 2-like isoform X1 [Carya illinoinensis]	carya illinoinensis
Endless_Summer_g71933	Endless_Summer_g71933	Endless Summer genom	Primary assembly	XP_028083082.1	66.9	XP_028083082.1 MADS-box protein SOC1-like isoform X1 [Camellia sinensis]	camellia sinensis
Endless_Summer_g71995	Endless_Summer_g71995	Endless Summer genom	Primary assembly	tr|A0A4Y7JG34|A0A4Y7JG34_PAPSO	55.4	tr|A0A4Y7JG34|A0A4Y7JG34 PAPSO GH16 domain-containing protein OS=Papaver somniferum OX=3469 GN=C5167 0062…	papaver somniferum
Endless_Summer_g72202	Endless_Summer_g72202	Endless Summer genom	Primary assembly	XP_034709216.1	53.7	XP_034709216.1 AT-rich interactive domain-containing protein 1 isoform X2 [Vitis riparia]	vitis riparia
Endless_Summer_g72422	Endless_Summer_g72422	Endless Summer genom	Primary assembly	XP_020215280.1	58.9	XP_020215280.1 cyanogenic beta-glucosidase [Cajanus cajan]	cajanus cajan
Endless_Summer_g72505	Endless_Summer_g72505	Endless Summer genom	Primary assembly	XP_015571965.1	37.2	XP_015571965.1 uncharacterized protein LOC107260911 [Ricinus communis]	ricinus communis
Endless_Summer_g72543	Endless_Summer_g72543	Endless Summer genom	Primary assembly	XP_040368457.1	39.7	XP_040368457.1 uncharacterized protein LOC112175850 isoform X1 [Rosa chinensis]	rosa chinensis
Endless_Summer_g72847	Endless_Summer_g72847	Endless Summer genom	Primary assembly	XP_034687365.1	75.4	XP_034687365.1 fatty acyl-CoA reductase 3-like [Vitis riparia]	vitis riparia
Endless_Summer_g72902	Endless_Summer_g72902	Endless Summer genom	Primary assembly	XP_024950087.1	52.6	XP_024950087.1 uncharacterized protein LOC102629376 [Citrus sinensis]	citrus sinensis
Endless_Summer_g73032	Endless_Summer_g73032	Endless Summer genom	Primary assembly	XP_034903206.1	80.4	XP_034903206.1 uncharacterized protein LOC118040386 [Populus alba]	populus alba
Endless_Summer_g73033	Endless_Summer_g73033	Endless Summer genom	Primary assembly	XP_035551461.1	61.4	XP_035551461.1 uncharacterized protein LOC118349841 [Juglans regia]	juglans regia
Endless_Summer_g73158	Endless_Summer_g73158	Endless Summer genom	Primary assembly	XP_028104295.1	84.6	XP_028104295.1 elongation factor-like GTPase 1 isoform X1 [Camellia sinensis]	camellia sinensis
Endless_Summer_g73162	Endless_Summer_g73162	Endless Summer genom	Primary assembly	tr|A0A418XFQ5|A0A418XFQ5_9GAMM	59.8	tr|A0A418XFQ5|A0A418XFQ5 9GAMM Ty1/Copia family ribonuclease HI (Fragment) OS=Alcanivorax profundi OX=233836…	alcanivorax profundi
Endless_Summer_g73188	Endless_Summer_g73188	Endless Summer genom	Primary assembly	tr|A0A8I2AW09|A0A8I2AW09_VIBPH	52	tr|A0A8I2AW09|A0A8I2AW09 VIBPH Uncharacterized protein (Fragment) OS=Vibrio parahaemolyticus OX=670 GN=J0682 …	vibrio parahaemolyticus
Endless_Summer_g73229	Endless_Summer_g73229	Endless Summer genom	Primary assembly	XP_023747237.1	77.1	XP_023747237.1 geranylgeranyl diphosphate reductase, chloroplastic [Lactuca sativa]	lactuca sativa
Endless_Summer_g73231	Endless_Summer_g73231	Endless Summer genom	Primary assembly	XP_028078426.1	73.6	XP_028078426.1 F-box/WD-40 repeat-containing protein At5g21040 [Camellia sinensis]	camellia sinensis
Endless_Summer_g73234	Endless_Summer_g73234	Endless Summer genom	Primary assembly	tr|A0A5J5C7Z2|A0A5J5C7Z2_9ASTE	45.8	tr|A0A5J5C7Z2|A0A5J5C7Z2 9ASTE AAI domain-containing protein OS=Nyssa sinensis OX=561372 GN=F0562 001784 PE…	nyssa sinensis
Endless_Summer_g73250	Endless_Summer_g73250	Endless Summer genom	Primary assembly	tr|A0A6A2XVH2|A0A6A2XVH2_HIBSY	79.1	tr|A0A6A2XVH2|A0A6A2XVH2 HIBSY Valyl-tRNA synthetase OS=Hibiscus syriacus OX=106335 GN=F3Y22 tig00116954…	hibiscus syriacus
Endless_Summer_g73528	Endless_Summer_g73528	Endless Summer genom	Primary assembly	XP_023896277.1	52.9	XP_023896277.1 zinc finger BED domain-containing protein RICESLEEPER 2-like [Quercus suber]	quercus suber
Endless_Summer_g73804	Endless_Summer_g73804	Endless Summer genom	Primary assembly	tr|A0A835DAZ8|A0A835DAZ8_9MAGN	81.1	tr|A0A835DAZ8|A0A835DAZ8 9MAGN Uncharacterized protein OS=Tetracentron sinense OX=13715 GN=HHK36 020409 PE…	tetracentron sinense
Endless_Summer_g73844	Endless_Summer_g73844	Endless Summer genom	Primary assembly	tr|A0A4U1CX85|A0A4U1CX85_9BACI	32.8	tr|A0A4U1CX85|A0A4U1CX85 9BACI Integrase H2C2 domain-containing protein OS=Robertmurraya kyonggiensis OX=1037…	robertmurraya kyonggiensis
Endless_Summer_g73962	Endless_Summer_g73962	Endless Summer genom	Primary assembly	tr|A0A5B7BAY1|A0A5B7BAY1_DAVIN	57	tr|A0A5B7BAY1|A0A5B7BAY1 DAVIN Protein kinase domain-containing protein OS=Davidia involucrata OX=16924 GN=D…	davidia involucrata
Endless_Summer_g74084	Endless_Summer_g74084	Endless Summer genom	Primary assembly	tr|A0A2N9GS39|A0A2N9GS39_FAGSY	32.6	tr|A0A2N9GS39|A0A2N9GS39 FAGSY Uncharacterized protein OS=Fagus sylvatica OX=28930 GN=FSB LOCUS30072 PE=…	fagus sylvatica
Endless_Summer_g7418	Endless_Summer_g7418	Endless Summer genom	Primary assembly	tr|A0A7J0FUS2|A0A7J0FUS2_9ERIC	34.4	tr|A0A7J0FUS2|A0A7J0FUS2 9ERIC Uncharacterized protein OS=Actinidia rufa OX=165716 GN=Acr 15g0010310 PE=4 SV=…	actinidia rufa
Endless_Summer_g74227	Endless_Summer_g74227	Endless Summer genom	Primary assembly	XP_020257992.1	54.5	XP_020257992.1 putative nuclease HARBI1 [Asparagus officinalis]	asparagus officinalis
Endless_Summer_g74404	Endless_Summer_g74404	Endless Summer genom	Primary assembly	tr|A0A5J5BIH0|A0A5J5BIH0_9ASTE	55.7	tr|A0A5J5BIH0|A0A5J5BIH0 9ASTE SWIM-type domain-containing protein OS=Nyssa sinensis OX=561372 GN=F0562 023…	nyssa sinensis
Endless_Summer_g74924	Endless_Summer_g74924	Endless Summer genom	Primary assembly	tr|A0A833TFI0|A0A833TFI0_JUGRE	76.7	tr|A0A833TFI0|A0A833TFI0 JUGRE Reverse transcriptase domain-containing protein OS=Juglans regia OX=51240 GN=F2P5…	juglans regia
Endless_Summer_g7500	Endless_Summer_g7500	Endless Summer genom	Primary assembly	XP_027062797.1	24.4	XP_027062797.1 uncharacterized protein LOC113689176 [Coffea arabica]	coffea arabica
Endless_Summer_g75045	Endless_Summer_g75045	Endless Summer genom	Primary assembly	XP_028089233.1	44.7	XP_028089233.1 uncharacterized protein LOC114289664 [Camellia sinensis]	camellia sinensis
Endless_Summer_g75046	Endless_Summer_g75046	Endless Summer genom	Primary assembly	tr|A0A6D2IEE2|A0A6D2IEE2_9BRAS	80.4	tr|A0A6D2IEE2|A0A6D2IEE2 9BRAS RT RNaseH domain-containing protein OS=Microthlaspi erraticum OX=1685480 GN=…	microthlaspi erraticum
Endless_Summer_g75084	Endless_Summer_g75084	Endless Summer genom	Primary assembly	tr|A0A6A4L4L9|A0A6A4L4L9_9ERIC	31.1	tr|A0A6A4L4L9|A0A6A4L4L9 9ERIC PMD domain-containing protein (Fragment) OS=Rhododendron williamsianum OX=26…	rhododendron williamsianum
Endless_Summer_g75312	Endless_Summer_g75312	Endless Summer genom	Primary assembly	tr|A0A4Q3DDD1|A0A4Q3DDD1_9RICK	67.7	tr|A0A4Q3DDD1|A0A4Q3DDD1 9RICK RNA-directed DNA polymerase (Fragment) OS=Rickettsiaceae bacterium OX=202336…	rickettsiaceae bacterium
Endless_Summer_g75335	Endless_Summer_g75335	Endless Summer genom	Primary assembly	tr|A0A5B6YZH4|A0A5B6YZH4_DAVIN	66.4	tr|A0A5B6YZH4|A0A5B6YZH4 DAVIN DYW deaminase domain-containing protein OS=Davidia involucrata OX=16924 GN=…	davidia involucrata
Endless_Summer_g75613	Endless_Summer_g75613	Endless Summer genom	Primary assembly	tr|A0A3P6C3H7|A0A3P6C3H7_BRAOL	38.2	tr|A0A3P6C3H7|A0A3P6C3H7 BRAOL Uncharacterized protein (Fragment) OS=Brassica oleracea OX=3712 GN=BOLC4T251…	brassica oleracea
Endless_Summer_g75693	Endless_Summer_g75693	Endless Summer genom	Primary assembly	tr|A0A5B7BUA5|A0A5B7BUA5_DAVIN	55.7	tr|A0A5B7BUA5|A0A5B7BUA5 DAVIN NAM-associated domain-containing protein (Fragment) OS=Davidia involucrata OX=…	davidia involucrata
Endless_Summer_g7571	Endless_Summer_g7571	Endless Summer genom	Primary assembly	XP_031253897.1	63.8	XP_031253897.1 benzoate carboxyl methyltransferase-like [Pistacia vera]	pistacia vera
Endless_Summer_g75711	Endless_Summer_g75711	Endless Summer genom	Primary assembly	XP_028095483.1	38.5	XP_028095483.1 uncharacterized protein LOC114295453 [Camellia sinensis]	camellia sinensis
Endless_Summer_g75775	Endless_Summer_g75775	Endless Summer genom	Primary assembly	tr|A0A2N9GRM6|A0A2N9GRM6_FAGSY	76.6	tr|A0A2N9GRM6|A0A2N9GRM6 FAGSY Uncharacterized protein OS=Fagus sylvatica OX=28930 GN=FSB LOCUS30164 P…	fagus sylvatica
Endless_Summer_g76002	Endless_Summer_g76002	Endless Summer genom	Primary assembly	XP_020109460.1	41.8	XP_020109460.1 uncharacterized protein LOC109724894 [Ananas comosus]	ananas comosus
Endless_Summer_g76021	Endless_Summer_g76021	Endless Summer genom	Primary assembly	XP_030924826.1	56.3	XP_030924826.1 uncharacterized protein LOC115951825 [Quercus lobata]	quercus lobata
Endless_Summer_g76035	Endless_Summer_g76035	Endless Summer genom	Primary assembly	tr|A0A834H924|A0A834H924_RHOSS	63.7	tr|A0A834H924|A0A834H924 RHOSS Uncharacterized protein OS=Rhododendron simsii OX=118357 GN=RHSIM Rhsim02…	rhododendron simsii
Endless_Summer_g76180	Endless_Summer_g76180	Endless Summer genom	Primary assembly	tr|A0A6P6UDY8|A0A6P6UDY8_COFAR	50.5	tr|A0A6P6UDY8|A0A6P6UDY8 COFAR zinc finger MYM-type protein 1-like OS=Coffea arabica OX=13443 GN=LOC113710…	coffea arabica
Endless_Summer_g76255	Endless_Summer_g76255	Endless Summer genom	Primary assembly	XP_034197970.1	59.5	XP_034197970.1 uncharacterized protein LOC117613470 [Prunus dulcis]	prunus dulcis
Endless_Summer_g76265	Endless_Summer_g76265	Endless Summer genom	Primary assembly	XP_016461548.1	31.5	XP_016461548.1 PREDICTED: uncharacterized protein LOC107784869 [Nicotiana tabacum]	nicotiana tabacum
Endless_Summer_g7663	Endless_Summer_g7663	Endless Summer genom	Primary assembly	tr|A0A5J4ZNR0|A0A5J4ZNR0_9ASTE	29.5	tr|A0A5J4ZNR0|A0A5J4ZNR0 9ASTE Rx N domain-containing protein OS=Nyssa sinensis OX=561372 GN=F0562 014541…	nyssa sinensis
Endless_Summer_g76679	Endless_Summer_g76679	Endless Summer genom	Primary assembly	tr|A0A2R6R8X5|A0A2R6R8X5_ACTCC	47.8	tr|A0A2R6R8X5|A0A2R6R8X5 ACTCC NADH-ubiquinone oxidoreductase OS=Actinidia chinensis var. chinensis OX=15908…	actinidia chinensis var. chi…
Endless_Summer_g76687	Endless_Summer_g76687	Endless Summer genom	Primary assembly	tr|A0A699HZ75|A0A699HZ75_TANCI	41.1	tr|A0A699HZ75|A0A699HZ75 TANCI Transposon Ty3-G Gag-Pol polyprotein OS=Tanacetum cinerariifolium OX=118510 G…	tanacetum cinerariifolium
Endless_Summer_g76943	Endless_Summer_g76943	Endless Summer genom	Primary assembly	XP_028107584.1	73.4	XP_028107584.1 calcium-dependent protein kinase 26 [Camellia sinensis]	camellia sinensis
Endless_Summer_g76959	Endless_Summer_g76959	Endless Summer genom	Primary assembly	XP_008369472.2	79.1	XP_008369472.2 lysophospholipid acyltransferase 1-like [Malus domestica]	malus domestica
Endless_Summer_g77079	Endless_Summer_g77079	Endless Summer genom	Primary assembly	XP_015896153.1	75	XP_015896153.1 probable phospholipid hydroperoxide glutathione peroxidase isoform X1 [Ziziphus jujuba]	ziziphus jujuba

Endless_Summer_g77084	Endless_Summer_g77084	Endless Summer genom	Primary assembly	XP_042980334.1	55.2	XP_042980334.1 zinc finger BED domain-containing protein RICESLEEPER 2-like isoform X1 [Carya illinoinensis]	[illegible]
Endless_Summer_g77098	Endless_Summer_g77098	Endless Summer genom	Primary assembly	XP_028124889.1	80	XP_028124889.1 uncharacterized protein LOC114321858 [Camellia sinensis]	[illegible]
Endless_Summer_g77148	Endless_Summer_g77148	Endless Summer genom	Primary assembly	XP_034710662.1	65.7	XP_034710662.1 phosphoglycerate mutase-like protein AT74 [Vitis riparia]	[illegible]
Endless_Summer_g77184	Endless_Summer_g77184	Endless Summer genom	Primary assembly	tr|A0A5J4ZSP7|A0A5J4ZSP7_9ASTE	53.6	tr|A0A5J4ZSP7|A0A5J4ZSP7_9ASTE WRKY domain-containing protein OS=Nyssa sinensis OX=561372 GN=F0562_011547	[illegible]
Endless_Summer_g77320	Endless_Summer_g77320	Endless Summer genom	Primary assembly	XP_028115472.1	85.5	XP_028115472.1 DNA primase small subunit-like isoform X1 [Camellia sinensis]	[illegible]
Endless_Summer_g7736	Endless_Summer_g7736	Endless Summer genom	Primary assembly	XP_030440242.1	49.8	XP_030440242.1 agglutinin-like, partial [Syzygium oleosum]	[illegible]
Endless_Summer_g77384	Endless_Summer_g77384	Endless Summer genom	Primary assembly	tr|A0A2K1Y5K8|A0A2K1Y5K8_POPTR	81.6	tr|A0A2K1Y5K8|A0A2K1Y5K8_POPTR ATP-synt_C domain-containing protein OS=Populus trichocarpa OX=3694 GN=POP	[illegible]
Endless_Summer_g77509	Endless_Summer_g77509	Endless Summer genom	Primary assembly	tr|A0A2U1KWE7|A0A2U1KWE7_ARTAN	33.2	tr|A0A2U1KWE7|A0A2U1KWE7_ARTAN RNase H type-1 domain-containing protein OS=Artemisia annua OX=35608 GN=C	[illegible]
Endless_Summer_g77514	Endless_Summer_g77514	Endless Summer genom	Primary assembly	XP_044461508.1	49.2	XP_044461508.1 VQ motif-containing protein 31 [Mangifera indica]	[illegible]
Endless_Summer_g77549	Endless_Summer_g77549	Endless Summer genom	Primary assembly	tr|A0A5B6YQP7|A0A5B6YQP7_DAVIN	57.3	tr|A0A5B6YQP7|A0A5B6YQP7_DAVIN BED-type domain-containing protein OS=Davidia involucrata OX=16924 GN=Din_0(	[illegible]
Endless_Summer_g77568	Endless_Summer_g77568	Endless Summer genom	Primary assembly	tr|A0A4S4DFC9|A0A4S4DFC9_CAMSI	83	tr|A0A4S4DFC9|A0A4S4DFC9_CAMSI Germin-like protein OS=Camellia sinensis var. sinensis OX=542762 GN=TEA_0236	[illegible]
Endless_Summer_g77579	Endless_Summer_g77579	Endless Summer genom	Primary assembly	tr|A0A4S4EKT9|A0A4S4EKT9_CAMSI	86.2	tr|A0A4S4EKT9|A0A4S4EKT9_CAMSI Germin-like protein OS=Camellia sinensis var. sinensis OX=542762 GN=TEA_0114(	[illegible]
Endless_Summer_g77758	Endless_Summer_g77758	Endless Summer genom	Primary assembly	XP_011465864.1	33.6	XP_011465864.1 PREDICTED: uncharacterized protein LOC105351939 [Fragaria vesca subsp. vesca]	[illegible]
Endless_Summer_g78055	Endless_Summer_g78055	Endless Summer genom	Primary assembly	tr|A0A834ZLP3|A0A834ZLP3_9MAGN	31.9	tr|A0A834ZLP3|A0A834ZLP3_9MAGN Uncharacterized protein OS=Tetracentron sinense OX=13715 GN=HHK36_006860 PE=	[illegible]
Endless_Summer_g78082	Endless_Summer_g78082	Endless Summer genom	Primary assembly	XP_021978766.2	51.1	XP_021978766.2 zinc finger BED domain-containing protein RICESLEEPER 2-like [Helianthus annuus]	[illegible]
Endless_Summer_g78119	Endless_Summer_g78119	Endless Summer genom	Primary assembly	tr|A0A438D6C4|A0A438D6C4_VITVI	43.2	tr|A0A438D6C4|A0A438D6C4_VITVI Integrase catalytic domain-containing protein OS=Vitis vinifera OX=29760 GN=CK203	[illegible]
Endless_Summer_g78366	Endless_Summer_g78366	Endless Summer genom	Primary assembly	XP_034927076.1	57.5	XP_034927076.1 uncharacterized protein LOC118058481 isoform X2 [Populus alba]	[illegible]
Endless_Summer_g78404	Endless_Summer_g78404	Endless Summer genom	Primary assembly	XP_027186673.1	57	XP_027186673.1 uncharacterized protein LOC113784625 [Cicer arietinum]	[illegible]
Endless_Summer_g78503	Endless_Summer_g78503	Endless Summer genom	Primary assembly	XP_035834403.1	53.3	XP_035834403.1 zinc finger BED domain-containing protein RICESLEEPER 2-like isoform X1 [Helianthus annuus]	[illegible]
Endless_Summer_g78510	Endless_Summer_g78510	Endless Summer genom	Primary assembly	XP_022871214.1	79.5	XP_022871214.1 uncharacterized protein LOC111390412 [Olea europaea var. sylvestris]	[illegible]
Endless_Summer_g78539	Endless_Summer_g78539	Endless Summer genom	Primary assembly	XP_022017274.1	41.9	XP_022017274.1 zinc finger BED domain-containing protein RICESLEEPER 2 [Helianthus annuus]	[illegible]
Endless_Summer_g7862	Endless_Summer_g7862	Endless Summer genom	Primary assembly	tr|A0A834H1D6|A0A834H1D6_RHOSS	71.8	tr|A0A834H1D6|A0A834H1D6_RHOSS Terpene_synth_C domain-containing protein OS=Rhododendron simsii OX=118357 G	[illegible]
Endless_Summer_g7877	Endless_Summer_g7877	Endless Summer genom	Primary assembly	tr|A0A558HBQ1|A0A558HBQ1_9GAMM	39.4	tr|A0A558HBQ1|A0A558HBQ1_9GAMM Reverse transcriptase-like protein (Fragment) OS=Cobetia crustatorum OX=553385 G	[illegible]
Endless_Summer_g78901	Endless_Summer_g78901	Endless Summer genom	Primary assembly	XP_028100376.1	75.2	XP_028100376.1 ATP-dependent DNA helicase PIF1-like [Camellia sinensis]	[illegible]
Endless_Summer_g78943	Endless_Summer_g78943	Endless Summer genom	Primary assembly	tr|A0A438D3R4|A0A438D3R4_VITVI	40.6	tr|A0A438D3R4|A0A438D3R4_VITVI Retrovirus-related Pol polyprotein from transposon 17.6 OS=Vitis vinifera OX=29760 G	[illegible]
Endless_Summer_g79014	Endless_Summer_g79014	Endless Summer genom	Primary assembly	tr|A0A5J5AIE8|A0A5J5AIE8_9ASTE	78.8	tr|A0A5J5AIE8|A0A5J5AIE8_9ASTE Glycosyltransferase OS=Nyssa sinensis OX=561372 GN=F0562_005541 PE=3 SV=1	[illegible]
Endless_Summer_g79107	Endless_Summer_g79107	Endless Summer genom	Primary assembly	tr|A0A7J7FZC3|A0A7J7FZC3_CAMSI	28.8	tr|A0A7J7FZC3|A0A7J7FZC3_CAMSI Retrotrans_gag domain-containing protein OS=Camellia sinensis OX=4442 GN=HYC8	[illegible]
Endless_Summer_g79171	Endless_Summer_g79171	Endless Summer genom	Primary assembly	tr|A0A7M4BQA9|A0A7M4BQA9_9MICC	46.7	tr|A0A7M4BQA9|A0A7M4BQA9_9MICC Uncharacterized protein OS=Rothia koreensis OX=592378 GN=GMA10_12985 PE=	[illegible]
Endless_Summer_g79191	Endless_Summer_g79191	Endless Summer genom	Primary assembly	XP_028064637.1	93.7	XP_028064637.1 guanosine nucleotide diphosphate dissociation inhibitor At5g09550-like isoform X1 [Camellia sinensis]	[illegible]
Endless_Summer_g79251	Endless_Summer_g79251	Endless Summer genom	Primary assembly	XP_028107127.1	39.2	XP_028107127.1 uncharacterized protein LOC114306133 [Camellia sinensis]	[illegible]
Endless_Summer_g79290	Endless_Summer_g79290	Endless Summer genom	Primary assembly	tr|A0A835D789|A0A835D789_9MAGN	56.8	tr|A0A835D789|A0A835D789_9MAGN Uncharacterized protein OS=Tetracentron sinense OX=13715 GN=HHK36_021314 PE=	[illegible]
Endless_Summer_g79294	Endless_Summer_g79294	Endless Summer genom	Primary assembly	XP_009762365.1	34.4	XP_009762365.1 PREDICTED: uncharacterized protein LOC104214404 [Nicotiana sylvestris]	[illegible]
Endless_Summer_g79326	Endless_Summer_g79326	Endless Summer genom	Primary assembly	tr|A0A4R5JD76|A0A4R5JD76_9FIRM	37	tr|A0A4R5JD76|A0A4R5JD76_9FIRM Reverse transcriptase domain-containing protein (Fragment) OS=Zhaonella formicivorans	[illegible]
Endless_Summer_g79534	Endless_Summer_g79534	Endless Summer genom	Primary assembly	XP_042455122.1	51.1	XP_042455122.1 zinc finger BED domain-containing protein RICESLEEPER 1-like [Zingiber officinale]	[illegible]
Endless_Summer_g79540	Endless_Summer_g79540	Endless Summer genom	Primary assembly	XP_024970992.1	74.1	XP_024970992.1 uncharacterized protein LOC112509980 [Cynara cardunculus var. scolymus]	[illegible]
Endless_Summer_g79563	Endless_Summer_g79563	Endless Summer genom	Primary assembly	tr|A0A5B6Z401|A0A5B6Z401_DAVIN	79.3	tr|A0A5B6Z401|A0A5B6Z401_DAVIN Amino-acid N-acetyltransferase OS=Davidia involucrata OX=16924 GN=Din_008388 PI	[illegible]
Endless_Summer_g79586	Endless_Summer_g79586	Endless Summer genom	Primary assembly	XP_028119765.1	48.6	XP_028119765.1 uncharacterized protein LOC114317252 [Camellia sinensis]	[illegible]
Endless_Summer_g79588	Endless_Summer_g79588	Endless Summer genom	Primary assembly	tr|A0A2I4FW02|A0A2I4FW02_JUGRE	32.5	tr|A0A2I4FW02|A0A2I4FW02_JUGRE uncharacterized protein LOC109002494 OS=Juglans regia OX=51240 GN=LOC109002	[illegible]
Endless_Summer_g79668	Endless_Summer_g79668	Endless Summer genom	Primary assembly	tr|A0A834T0D5|A0A834T0D5_9FABA	40.8	tr|A0A834T0D5|A0A834T0D5_9FABA Retrotrans_gag domain-containing protein OS=Senna tora OX=362788 GN=G2W53_0	[illegible]
Endless_Summer_g79912	Endless_Summer_g79912	Endless Summer genom	Primary assembly	tr|A0A4V2ZP56|A0A4V2ZP56_9FIRM	40.4	tr|A0A4V2ZP56|A0A4V2ZP56_9FIRM Uncharacterized protein OS=Zhaonella formicivorans OX=2528593 GN=EYS13_15615	[illegible]
Endless_Summer_g80071	Endless_Summer_g80071	Endless Summer genom	Primary assembly	XP_028084687.1	43.5	XP_028084687.1 uncharacterized protein LOC114285806 [Camellia sinensis]	[illegible]
Endless_Summer_g80266	Endless_Summer_g80266	Endless Summer genom	Primary assembly	XP_012843131.1	66.3	XP_012843131.1 PREDICTED: uncharacterized protein LOC105963284 [Erythranthe guttata]	[illegible]
Endless_Summer_g80294	Endless_Summer_g80294	Endless Summer genom	Primary assembly	tr|A0A7J0E1J9|A0A7J0E1J9_9ERIC	84	tr|A0A7J0E1J9|A0A7J0E1J9_9ERIC ADP-ribosylation factor family protein OS=Actinidia rufa OX=165716 GN=Acr_01g00019	[illegible]
Endless_Summer_g80469	Endless_Summer_g80469	Endless Summer genom	Primary assembly	tr|A0A5B6ZIH5|A0A5B6ZIH5_DAVIN	81.2	tr|A0A5B6ZIH5|A0A5B6ZIH5_DAVIN Uncharacterized protein (Fragment) OS=Davidia involucrata OX=16924 GN=Din_01263	[illegible]
Endless_Summer_g8052	Endless_Summer_g8052	Endless Summer genom	Primary assembly	XP_006383725.2	44	XP_006383725.2 jacalin-related lectin 3 isoform X2 [Populus trichocarpa]	[illegible]
Endless_Summer_g80553	Endless_Summer_g80553	Endless Summer genom	Primary assembly	XP_011099892.1	74.8	XP_011099892.1 phospholipase D delta [Sesamum indicum]	[illegible]
Endless_Summer_g80632	Endless_Summer_g80632	Endless Summer genom	Primary assembly	tr|A0A5B7AQE4|A0A5B7AQE4_DAVIN	73.9	tr|A0A5B7AQE4|A0A5B7AQE4_DAVIN Uncharacterized protein OS=Davidia involucrata OX=16924 GN=Din_027082 PE=4 S	[illegible]
Endless_Summer_g80646	Endless_Summer_g80646	Endless Summer genom	Primary assembly	XP_028109089.1	72.5	XP_028109089.1 LOW QUALITY PROTEIN: glutamate receptor 3.6-like [Camellia sinensis]	[illegible]
Endless_Summer_g80875	Endless_Summer_g80875	Endless Summer genom	Primary assembly	XP_028105270.1	81.4	XP_028105270.1 LOW QUALITY PROTEIN: glucan endo-1,3-beta-glucosidase 2-like [Camellia sinensis]	[illegible]
Endless_Summer_g81036	Endless_Summer_g81036	Endless Summer genom	Primary assembly	XP_034707825.1	76.8	XP_034707825.1 protein ABA DEFICIENT 4, chloroplastic isoform X1 [Vitis riparia]	[illegible]
Endless_Summer_g81081	Endless_Summer_g81081	Endless Summer genom	Primary assembly	XP_027077316.1	74.8	XP_027077316.1 probable translation initiation factor eIF-2B subunit delta isoform X1 [Coffea arabica]	[illegible]
Endless_Summer_g81104	Endless_Summer_g81104	Endless Summer genom	Primary assembly	XP_022861641.1	92.3	XP_022861641.1 uncharacterized protein LOC111382013 [Olea europaea var. sylvestris]	[illegible]
Endless_Summer_g81199	Endless_Summer_g81199	Endless Summer genom	Primary assembly	XP_028093598.1	38.1	XP_028093598.1 uncharacterized protein LOC114293688 [Camellia sinensis]	[illegible]
Endless_Summer_g81284	Endless_Summer_g81284	Endless Summer genom	Primary assembly	tr|A0A7J7LPT0|A0A7J7LPT0_9MAGN	53.2	tr|A0A7J7LPT0|A0A7J7LPT0_9MAGN Uncharacterized protein OS=Kingdonia uniflora OX=39325 GN=GIB67_006071 PE=4	[illegible]
Endless_Summer_g81287	Endless_Summer_g81287	Endless Summer genom	Primary assembly	tr|A0A7D9I0E7|A0A7D9I0E7_PARCT	54.2	tr|A0A7D9I0E7|A0A7D9I0E7_PARCT Mitochondrial-processing peptidase subunit beta (Fragment) OS=Paramuricea clavata OX	[illegible]
Endless_Summer_g81321	Endless_Summer_g81321	Endless Summer genom	Primary assembly	tr|A0A5B6Z1I6|A0A5B6Z1I6_DAVIN	40.8	tr|A0A5B6Z1I6|A0A5B6Z1I6_DAVIN DDE_Tnp4 domain-containing protein OS=Davidia involucrata OX=16924 GN=Din_007	[illegible]
Endless_Summer_g81363	Endless_Summer_g81363	Endless Summer genom	Primary assembly	XP_028066215.1	79	XP_028066215.1 polypyrimidine tract-binding protein homolog 1-like isoform X1 [Camellia sinensis]	[illegible]
Endless_Summer_g8141	Endless_Summer_g8141	Endless Summer genom	Primary assembly	tr|A0A7J9JB64|A0A7J9JB64_9ROSI	38.4	tr|A0A7J9JB64|A0A7J9JB64_9ROSI Uncharacterized protein (Fragment) OS=Gossypium armourianum OX=34283 GN=Goarm	[illegible]
Endless_Summer_g81561	Endless_Summer_g81561	Endless Summer genom	Primary assembly	tr|A0A822Y304|A0A822Y304_NELNU	37.2	tr|A0A822Y304|A0A822Y304_NELNU Uncharacterized protein OS=Nelumbo nucifera OX=4432 GN=HUJ06_027013 PE=4 SV	[illegible]
Endless_Summer_g81688	Endless_Summer_g81688	Endless Summer genom	Primary assembly	tr|A0A5B7ACB1|A0A5B7ACB1_DAVIN	51.1	tr|A0A5B7ACB1|A0A5B7ACB1_DAVIN Uncharacterized protein OS=Davidia involucrata OX=16924 GN=Din_023811 PE=4 S	[illegible]
Endless_Summer_g81869	Endless_Summer_g81869	Endless Summer genom	Primary assembly	XP_022883950.1	55.6	XP_022883950.1 CASP-like protein 1F2 [Olea europaea var. sylvestris]	[illegible]
Endless_Summer_g81873	Endless_Summer_g81873	Endless Summer genom	Primary assembly	XP_027103137.1	29.2	XP_027103137.1 uncharacterized protein LOC113724434 [Coffea arabica]	[illegible]
Endless_Summer_g8317	Endless_Summer_g8317	Endless Summer genom	Primary assembly	tr|A0A5B6ZY34|A0A5B6ZY34_DAVIN	50.5	tr|A0A5B6ZY34|A0A5B6ZY34_DAVIN Putative disease resistance RPP13-like protein 1 OS=Davidia involucrata OX=16924 G	[illegible]
Endless_Summer_g8434	Endless_Summer_g8434	Endless Summer genom	Primary assembly	tr|A0A834T922|A0A834T922_9FABA	58.7	tr|A0A834T922|A0A834T922_9FABA Retrovirus-related Pol polyprotein from transposon TNT 1-94 OS=Senna tora OX=3627	[illegible]
Endless_Summer_g8438	Endless_Summer_g8438	Endless Summer genom	Primary assembly	XP_020412113.1	44.1	XP_020412113.1 uncharacterized protein LOC109947087 [Prunus persica]	[illegible]
Endless_Summer_g8501	Endless_Summer_g8501	Endless Summer genom	Primary assembly	tr|A0A5J4ZDU5|A0A5J4ZDU5_9ASTE	67.2	tr|A0A5J4ZDU5|A0A5J4ZDU5_9ASTE Helicase ATP-binding domain-containing protein OS=Nyssa sinensis OX=561372 GN=	[illegible]
Endless_Summer_g8502	Endless_Summer_g8502	Endless Summer genom	Primary assembly	XP_028119528.1	37.9	XP_028119528.1 uncharacterized protein LOC114317038 [Camellia sinensis]	[illegible]
Endless_Summer_g8555	Endless_Summer_g8555	Endless Summer genom	Primary assembly	XP_030932563.1	84.5	XP_030932563.1 protein-ribulosamine 3-kinase, chloroplastic [Quercus lobata]	[illegible]
Endless_Summer_g8615	Endless_Summer_g8615	Endless Summer genom	Primary assembly	XP_023871790.1	26.4	XP_023871790.1 replication protein A 70 kDa DNA-binding subunit D-like [Quercus suber]	[illegible]
Endless_Summer_g8630	Endless_Summer_g8630	Endless Summer genom	Primary assembly	tr|A0A2N9I4B1|A0A2N9I4B1_FAGSY	64.3	tr|A0A2N9I4B1|A0A2N9I4B1_FAGSY RNA-directed DNA polymerase OS=Fagus sylvatica OX=28930 GN=FSB_LOCUS467	[illegible]
Endless_Summer_g8702	Endless_Summer_g8702	Endless Summer genom	Primary assembly	XP_028067271.1	63.8	XP_028067271.1 autophagy-related protein 18h-like isoform X1 [Camellia sinensis]	[illegible]
Endless_Summer_g882	Endless_Summer_g882	Endless Summer genom	Primary assembly	tr|A0A7J7LK53|A0A7J7LK53_9MAGN	96.7	tr|A0A7J7LK53|A0A7J7LK53_9MAGN Uncharacterized protein OS=Kingdonia uniflora OX=39325 GN=GIB67_003895 PE=3	[illegible]
Endless_Summer_g892	Endless_Summer_g892	Endless Summer genom	Primary assembly	tr|A0A5D2UQB3|A0A5D2UQB3_GOSMU	93.6	tr|A0A5D2UQB3|A0A5D2UQB3_GOSMU Photosystem I assembly protein Ycf4 OS=Gossypium mustelinum OX=34275 GN=E	[illegible]
Endless_Summer_g9006	Endless_Summer_g9006	Endless Summer genom	Primary assembly	XP_047330617.1	53.9	XP_047330617.1 MLO-like protein 13 isoform X1 [Impatiens glandulifera]	[illegible]
Endless_Summer_g9015	Endless_Summer_g9015	Endless Summer genom	Primary assembly	XP_028073914.1	61	XP_028073914.1 probable leucine-rich repeat receptor-like protein kinase At5g49770 isoform X1 [Camellia sinensis]	[illegible]
Endless_Summer_g9231	Endless_Summer_g9231	Endless Summer genom	Primary assembly	XP_012845932.1	68.7	XP_012845932.1 PREDICTED: putative nuclease HARBI1 [Erythranthe guttata]	[illegible]
Endless_Summer_g9335	Endless_Summer_g9335	Endless Summer genom	Primary assembly	XP_022896914.1	61.6	XP_022896914.1 L-type lectin-domain containing receptor kinase IX.1-like [Olea europaea var. sylvestris]	[illegible]
Endless_Summer_g9401	Endless_Summer_g9401	Endless Summer genom	Primary assembly	tr|A0A6J5X9V2|A0A6J5X9V2_PRUAR	64.2	tr|A0A6J5X9V2|A0A6J5X9V2_PRUAR RNase H type-1 domain-containing protein OS=Prunus armeniaca OX=36596 GN=CUI	[illegible]
Endless_Summer_g9432	Endless_Summer_g9432	Endless Summer genom	Primary assembly	XP_011000836.1	56.2	XP_011000836.1 PREDICTED: uncharacterized protein LOC105108282 [Populus euphratica]	[illegible]
Endless_Summer_g95	Endless_Summer_g95	Endless Summer genom	Primary assembly	XP_027120740.1	61.3	XP_027120740.1 uncharacterized protein LOC113737757 [Coffea arabica]	[illegible]
Endless_Summer_g9509	Endless_Summer_g9509	Endless Summer genom	Primary assembly	tr|A0A5J4ZJG7|A0A5J4ZJG7_9ASTE	42.6	tr|A0A5J4ZJG7|A0A5J4ZJG7_9ASTE Rep_fac-A_C domain-containing protein OS=Nyssa sinensis OX=561372 GN=F0562_01	[illegible]
Endless_Summer_g9712	Endless_Summer_g9712	Endless Summer genom	Primary assembly	XP_023872064.1	46.4	XP_023872064.1 uncharacterized protein LOC111984679 [Quercus suber]	[illegible]
Endless_Summer_g9858	Endless_Summer_g9858	Endless Summer genom	Primary assembly	XP_028081950.1	76.1	XP_028081950.1 RNA N6-adenosine-methyltransferase mettl16-like [Camellia sinensis]	[illegible]
Endless_Summer_g9918	Endless_Summer_g9918	Endless Summer genom	Primary assembly	tr|A0A2N9FMX4|A0A2N9FMX4_FAGSY	50.9	tr|A0A2N9FMX4|A0A2N9FMX4_FAGSY Uncharacterized protein OS=Fagus sylvatica OX=28930 GN=FSB_LOCUS16347 PI	[illegible]
Endless_Summer_g996	Endless_Summer_g996	Endless Summer genom	Primary assembly	tr|A0A834YAQ3|A0A834YAQ3_9MAGN	94.5	tr|A0A834YAQ3|A0A834YAQ3_9MAGN ATP-synt_C domain-containing protein OS=Tetracentron sinense OX=13715 GN=HI	[illegible]
endless_summer2_g10442	endless_summer2_g10442	Endless Summer genom	Alternative assembl	tr|A0A5B7C912|A0A5B7C912_DAVIN	69.7	tr|A0A5B7C912|A0A5B7C912_DAVIN Putative cellulose synthase-like protein G1 (Fragment) OS=Davidia involucrata OX=16	[illegible]
endless_summer2_g10465	endless_summer2_g10465	Endless Summer genom	Alternative assembl	XP_041009463.1	52.6	XP_041009463.1 PLASMODESMATA CALLOSE-BINDING PROTEIN 1-like [Juglans microcarpa x Juglans regia]	[illegible]
endless_summer2_g1057	endless_summer2_g1057	Endless Summer genom	Alternative assembl	XP_023927100.1	39.4	XP_023927100.1 uncharacterized protein LOC112038511 isoform X1 [Quercus suber]	[illegible]
endless_summer2_g10822	endless_summer2_g10822	Endless Summer genom	Alternative assembl	tr|A0A2N9GQ91|A0A2N9GQ91_FAGSY	27	tr|A0A2N9GQ91|A0A2N9GQ91_FAGSY Uncharacterized protein OS=Fagus sylvatica OX=28930 GN=FSB_LOCUS29607 PE-	[illegible]
endless_summer2_g10848	endless_summer2_g10848	Endless Summer genom	Alternative assembl	XP_028113580.1	78.1	XP_028113580.1 protein JINGUBANG-like [Camellia sinensis]	[illegible]
endless_summer2_g10957	endless_summer2_g10957	Endless Summer genom	Alternative assembl	XP_028078861.1	52.2	XP_028078861.1 thionin-like protein 2 [Camellia sinensis]	[illegible]
endless_summer2_g1096	endless_summer2_g1096	Endless Summer genom	Alternative assembl	tr|A0A5B7C5E0|A0A5B7C5E0_DAVIN	47.5	tr|A0A5B7C5E0|A0A5B7C5E0_DAVIN Uncharacterized protein OS=Davidia involucrata OX=16924 GN=Din_044014 PE=4 S'	[illegible]
endless_summer2_g10999	endless_summer2_g10999	Endless Summer genom	Alternative assembl	XP_034696329.1	79.2	XP_034696329.1 probable 2-oxoglutarate-dependent dioxygenase AOP1 [Vitis riparia]	[illegible]
endless_summer2_g11009	endless_summer2_g11009	Endless Summer genom	Alternative assembl	tr|A0A2K3K848|A0A2K3K848_TRIPR	48.8	tr|A0A2K3K848|A0A2K3K848_TRIPR Cysteine-rich receptor-like protein kinase (Fragment) OS=Trifolium pratense OX=57577	[illegible]
endless_summer2_g11098	endless_summer2_g11098	Endless Summer genom	Alternative assembl	XP_028105264.1	43.8	XP_028105264.1 uncharacterized protein LOC114304299, partial [Camellia sinensis]	[illegible]
endless_summer2_g11208	endless_summer2_g11208	Endless Summer genom	Alternative assembl	XP_015169574.1	30.2	XP_015169574.1 PREDICTED: uncharacterized protein LOC107062893 [Solanum tuberosum]	[illegible]
endless_summer2_g11230	endless_summer2_g11230	Endless Summer genom	Alternative assembl	tr|A0A6P6V931|A0A6P6V931_COFAR	28.1	tr|A0A6P6V931|A0A6P6V931_COFAR uncharacterized protein LOC113718416 OS=Coffea arabica OX=13443 GN=LOC11371	[illegible]
endless_summer2_g1128	endless_summer2_g1128	Endless Summer genom	Alternative assembl	XP_020242770.1	72	XP_020242770.1 uncharacterized protein LOC109820985 [Asparagus officinalis]	[illegible]
endless_summer2_g11302	endless_summer2_g11302	Endless Summer genom	Alternative assembl	XP_026459437.1	28.6	XP_026459437.1 uncharacterized protein LOC113360105 [Papaver somniferum]	[illegible]
endless_summer2_g11359	endless_summer2_g11359	Endless Summer genom	Alternative assembl	XP_034675850.1	78.3	XP_034675850.1 RNA-binding protein 24-like isoform X1 [Vitis riparia]	[illegible]
endless_summer2_g11638	endless_summer2_g11638	Endless Summer genom	Alternative assembl	tr|A0A834GW55|A0A834GW55_RHOSS	39.1	tr|A0A834GW55|A0A834GW55_RHOSS CCHC-type domain-containing protein OS=Rhododendron simsii OX=118357 GN=I	[illegible]
endless_summer2_g12101	endless_summer2_g12101	Endless Summer genom	Alternative assembl	XP_038705337.1	64.9	XP_038705337.1 uncharacterized protein LOC120001138 [Tripterygium wilfordii]	[illegible]
endless_summer2_g12152	endless_summer2_g12152	Endless Summer genom	Alternative assembl	tr|A0A5B7BIU9|A0A5B7BIU9_DAVIN	66	tr|A0A5B7BIU9|A0A5B7BIU9_DAVIN Receptor-like serine/threonine-protein kinase OS=Davidia involucrata OX=16924 GN=I	[illegible]
endless_summer2_g12175	endless_summer2_g12175	Endless Summer genom	Alternative assembl	XP_028114298.1	66.7	XP_028114298.1 uncharacterized protein LOC114312279 [Camellia sinensis]	[illegible]
endless_summer2_g12240	endless_summer2_g12240	Endless Summer genom	Alternative assembl	tr|A0A4Y8W7X4|A0A4Y8W7X4_9VIBR	28.7	tr|A0A4Y8W7X4|A0A4Y8W7X4_9VIBR Reverse transcriptase domain-containing protein (Fragment) OS=Vibrio ouci OX=249	[illegible]
endless_summer2_g12259	endless_summer2_g12259	Endless Summer genom	Alternative assembl	tr|A0A2K3K7L4|A0A2K3K7L4_TRIPR	49.5	tr|A0A2K3K7L4|A0A2K3K7L4_TRIPR Uncharacterized protein (Fragment) OS=Trifolium pratense OX=57577 GN=L195_g061	[illegible]
endless_summer2_g12268	endless_summer2_g12268	Endless Summer genom	Alternative assembl	XP_038699598.1	40.9	XP_038699598.1 zinc finger BED domain-containing protein RICESLEEPER 2-like [Tripterygium wilfordii]	[illegible]
endless_summer2_g12307	endless_summer2_g12307	Endless Summer genom	Alternative assembl	XP_026441802.1	35.2	XP_026441802.1 uncharacterized protein LOC113340978 [Papaver somniferum]	[illegible]
endless_summer2_g12436	endless_summer2_g12436	Endless Summer genom	Alternative assembl	tr|A0A2P2KD31|A0A2P2KD31_RHIMU	74.7	tr|A0A2P2KD31|A0A2P2KD31_RHIMU WW domain-containing protein OS=Rhizophora mucronata OX=61149 PE=4 SV=1	[illegible]
endless_summer2_g12437	endless_summer2_g12437	Endless Summer genom	Alternative assembl	tr|A0A4C1UX64|A0A4C1UX64_EUMVA	26.8	tr|A0A4C1UX64|A0A4C1UX64_EUMVA Retrovirus-related Pol polyprotein from transposon TNT 1-94 OS=Eumeta variegata (	[illegible]
endless_summer2_g12438	endless_summer2_g12438	Endless Summer genom	Alternative assembl	XP_028120098.1	49.8	XP_028120098.1 uncharacterized protein LOC114317550 isoform X1 [Camellia sinensis]	[illegible]
endless_summer2_g12458	endless_summer2_g12458	Endless Summer genom	Alternative assembl	XP_027124349.1	55.1	XP_027124349.1 uncharacterized protein LOC113741057 [Coffea arabica]	[illegible]
endless_summer2_g12497	endless_summer2_g12497	Endless Summer genom	Alternative assembl	XP_015961036.1	35.1	XP_015961036.1 uncharacterized protein LOC107485010 [Arachis duranensis]	[illegible]
endless_summer2_g12561	endless_summer2_g12561	Endless Summer genom	Alternative assembl	XP_031252311.1	58.7	XP_031252311.1 wall-associated receptor kinase 2-like [Pistacia vera]	[illegible]
endless_summer2_g12579	endless_summer2_g12579	Endless Summer genom	Alternative assembl	XP_019253961.1	29.5	XP_019253961.1 PREDICTED: uncharacterized protein LOC109232660 [Nicotiana attenuata]	[illegible]
endless_summer2_g12601	endless_summer2_g12601	Endless Summer genom	Alternative assembl	tr|A0A5B7AP96|A0A5B7AP96_DAVIN	69.4	tr|A0A5B7AP96|A0A5B7AP96_DAVIN Uncharacterized protein (Fragment) OS=Davidia involucrata OX=16924 GN=Din_0278	[illegible]
endless_summer2_g12749	endless_summer2_g12749	Endless Summer genom	Alternative assembl	tr|A0A2P6PD65|A0A2P6PD65_ROSCH	37.3	tr|A0A2P6PD65|A0A2P6PD65_ROSCH RNA-directed DNA polymerase OS=Rosa chinensis OX=74649 GN=RchiOBHm_Chr	[illegible]
endless_summer2_g12756	endless_summer2_g12756	Endless Summer genom	Alternative assembl	tr|A0A2N9HG34|A0A2N9HG34_FAGSY	30.6	tr|A0A2N9HG34|A0A2N9HG34_FAGSY Myb_DNA-bind_3 domain-containing protein OS=Fagus sylvatica OX=28930 GN=F	[illegible]
endless_summer2_g12791	endless_summer2_g12791	Endless Summer genom	Alternative assembl	tr|F4NCK1|F4NCK1_BETVV	35.3	tr|F4NCK1|F4NCK1_BETVV Reverse transcriptase domain-containing protein OS=Beta vulgaris subsp. vulgaris OX=3555 PE-	[illegible]
endless_summer2_g12797	endless_summer2_g12797	Endless Summer genom	Alternative assembl	tr|A0A6P6UFU4|A0A6P6UFU4_COFAR	29.1	tr|A0A6P6UFU4|A0A6P6UFU4_COFAR uncharacterized protein LOC113710476 OS=Coffea arabica OX=13443 GN=LOC1137	[illegible]
endless_summer2_g12905	endless_summer2_g12905	Endless Summer genom	Alternative assembl	tr|A0A5J5B3Q4|A0A5J5B3Q4_9ASTE	66.5	tr|A0A5J5B3Q4|A0A5J5B3Q4_9ASTE Uncharacterized protein OS=Nyssa sinensis OX=561372 GN=F0562_027525 PE=4 SV-	[illegible]
endless_summer2_g12991	endless_summer2_g12991	Endless Summer genom	Alternative assembl	XP_020424590.1	31.4	XP_020424590.1 uncharacterized protein LOC109950407 [Prunus persica]	[illegible]
endless_summer2_g13039	endless_summer2_g13039	Endless Summer genom	Alternative assembl	tr|A0A834Z1I2|A0A834Z1I2_9MAGN	71.6	tr|A0A834Z1I2|A0A834Z1I2_9MAGN Uncharacterized protein OS=Tetracentron sinense OX=13715 GN=HHK36_014530 PE=4	[illegible]
endless_summer2_g13047	endless_summer2_g13047	Endless Summer genom	Alternative assembl	XP_028120954.1	72.3	XP_028120954.1 uncharacterized protein LOC114318302 [Camellia sinensis]	[illegible]
endless_summer2_g13234	endless_summer2_g13234	Endless Summer genom	Alternative assembl	tr|A0A540NPV2|A0A540NPV2_MALBA	46.4	tr|A0A540NPV2|A0A540NPV2_MALBA Integrase catalytic domain-containing protein OS=Malus baccata OX=106549 GN=C1	[illegible]
endless_summer2_g13242	endless_summer2_g13242	Endless Summer genom	Alternative assembl	XP_028085763.1	78.9	XP_028085763.1 protein EXORDIUM-like isoform X2 [Camellia sinensis]	[illegible]
endless_summer2_g13303	endless_summer2_g13303	Endless Summer genom	Alternative assembl	tr|A0A834G286|A0A834G286_RHOSS	33.8	tr|A0A834G286|A0A834G286_RHOSS PMD domain-containing protein OS=Rhododendron simsii OX=118357 GN=RHSIM_I	[illegible]
endless_summer2_g13408	endless_summer2_g13408	Endless Summer genom	Alternative assembl	tr|A0A5B6ZQY4|A0A5B6ZQY4_DAVIN	76.6	tr|A0A5B6ZQY4|A0A5B6ZQY4_DAVIN Auxin-responsive protein OS=Davidia involucrata OX=16924 GN=Din_015377 PE=3	[illegible]
endless_summer2_g1349	endless_summer2_g1349	Endless Summer genom	Alternative assembl	XP_003632035.1	40	XP_003632035.1 PREDICTED: uncharacterized protein LOC100854167 [Vitis vinifera]	[illegible]
endless_summer2_g13504	endless_summer2_g13504	Endless Summer genom	Alternative assembl	tr|A0A5J5ABS7|A0A5J5ABS7_9ASTE	73.3	tr|A0A5J5ABS7|A0A5J5ABS7_9ASTE Adenine phosphoribosyltransferase OS=Nyssa sinensis OX=561372 GN=F0562_0358	[illegible]
endless_summer2_g13535	endless_summer2_g13535	Endless Summer genom	Alternative assembl	XP_006362073.1	81.5	XP_006362073.1 PREDICTED: uncharacterized protein LOC102578171 [Solanum tuberosum]	[illegible]
endless_summer2_g13549	endless_summer2_g13549	Endless Summer genom	Alternative assembl	XP_028085043.1	84.5	XP_028085043.1 mannose-P-dolichol utilization defect 1 protein homolog 2 isoform X1 [Camellia sinensis]	[illegible]
endless_summer2_g13556	endless_summer2_g13556	Endless Summer genom	Alternative assembl	tr|A0A830C981|A0A830C981_9LAMI	43.4	tr|A0A830C981|A0A830C981_9LAMI Uncharacterized protein OS=Phtheirospermum japonicum OX=374723 GN=PHJA_0017	[illegible]
endless_summer2_g13563	endless_summer2_g13563	Endless Summer genom	Alternative assembl	tr|A0A7J6XD91|A0A7J6XD91_THATH	70.3	tr|A0A7J6XD91|A0A7J6XD91_THATH Transposon tf2-9 polyprotein (Fragment) OS=Thalictrum thalictroides OX=46969 GN=	[illegible]
endless_summer2_g13596	endless_summer2_g13596	Endless Summer genom	Alternative assembl	tr|M1DGW6|M1DGW6_SOLTU	31.5	tr|M1DGW6|M1DGW6_SOLTU Gag-pro OS=Solanum tuberosum OX=4113 PE=4 SV=1	[illegible]
endless_summer2_g13708	endless_summer2_g13708	Endless Summer genom	Alternative assembl	XP_010059967.2	76.9	XP_010059967.2 patatin-like protein 2 [Eucalyptus grandis]	[illegible]
endless_summer2_g13722	endless_summer2_g13722	Endless Summer genom	Alternative assembl	tr|A0A834G690|A0A834G690_RHOSS	32.3	tr|A0A834G690|A0A834G690_RHOSS SWIM-type domain-containing protein OS=Rhododendron simsii OX=118357 GN=RH	[illegible]
endless_summer2_g13808	endless_summer2_g13808	Endless Summer genom	Alternative assembl	tr|A0A2K3L0W0|A0A2K3L0W0_TRIPR	42	tr|A0A2K3L0W0|A0A2K3L0W0_TRIPR Protein FAR1-RELATED SEQUENCE OS=Trifolium pratense OX=57577 GN=L19	[illegible]
endless_summer2_g13889	endless_summer2_g13889	Endless Summer genom	Alternative assembl	XP_026380697.1	25.5	XP_026380697.1 uncharacterized protein LOC113275419 isoform X3 [Papaver somniferum]	[illegible]
endless_summer2_g13901	endless_summer2_g13901	Endless Summer genom	Alternative assembl	tr|A0A5B6Z1D5|A0A5B6Z1D5_DAVIN	74.2	tr|A0A5B6Z1D5|A0A5B6Z1D5_DAVIN Protein kinase domain-containing protein OS=Davidia involucrata OX=16924 GN=Din	[illegible]
endless_summer2_g13930	endless_summer2_g13930	Endless Summer genom	Alternative assembl	XP_043617908.1	90.7	XP_043617908.1 proteasome subunit alpha type-1-A-like [Erigeron canadensis]	[illegible]
endless_summer2_g13959	endless_summer2_g13959	Endless Summer genom	Alternative assembl	XP_041027886.1	34.6	XP_041027886.1 replication protein A 70 kDa DNA-binding subunit B-like [Juglans microcarpa x Juglans regia]	[illegible]
endless_summer2_g13965	endless_summer2_g13965	Endless Summer genom	Alternative assembl	tr|A0A7J7HUY2|A0A7J7HUY2_CAMSI	64.2	tr|A0A7J7HUY2|A0A7J7HUY2_CAMSI Inositol-pentakisphosphate 2-kinase OS=Camellia sinensis OX=4442 GN=HYC85_00	[illegible]
endless_summer2_g13991	endless_summer2_g13991	Endless Summer genom	Alternative assembl	XP_028081787.1	34.7	XP_028081787.1 uncharacterized protein LOC114283156 [Camellia sinensis]	[illegible]
endless_summer2_g13996	endless_summer2_g13996	Endless Summer genom	Alternative assembl	XP_028112242.1	63.5	XP_028112242.1 BEL1-like homeodomain protein 11 isoform X1 [Camellia sinensis]	[illegible]
endless_summer2_g14078	endless_summer2_g14078	Endless Summer genom	Alternative assembl	tr|A0A2N9I4E3|A0A2N9I4E3_FAGSY	24.1	tr|A0A2N9I4E3|A0A2N9I4E3_FAGSY RNase H type-1 domain-containing protein OS=Fagus sylvatica OX=28930 GN=FSB_L	[illegible]
endless_summer2_g14297	endless_summer2_g14297	Endless Summer genom	Alternative assembl	XP_028060687.1	31.3	XP_028060687.1 uncharacterized protein LOC114264281 [Camellia sinensis]	[illegible]
endless_summer2_g1448	endless_summer2_g1448	Endless Summer genom	Alternative assembl	tr|A0A5B7BA39|A0A5B7BA39_DAVIN	69.9	tr|A0A5B7BA39|A0A5B7BA39_DAVIN Protein cereblon OS=Davidia involucrata OX=16924 GN=Din_034786 PE=3 SV=1	[illegible]
endless_summer2_g1450	endless_summer2_g1450	Endless Summer genom	Alternative assembl	tr|A0A5B7BAT3|A0A5B7BAT3_DAVIN	40.6	tr|A0A5B7BAT3|A0A5B7BAT3_DAVIN Retrotran_gag_3 domain-containing protein (Fragment) OS=Davidia involucrata OX=	[illegible]
endless_summer2_g14526	endless_summer2_g14526	Endless Summer genom	Alternative assembl	XP_028094324.1	25.7	XP_028094324.1 uncharacterized protein LOC114294393 [Camellia sinensis]	[illegible]
endless_summer2_g14529	endless_summer2_g14529	Endless Summer genom	Alternative assembl	tr|A0A2N9IAI1|A0A2N9IAI1_FAGSY	42.6	tr|A0A2N9IAI1|A0A2N9IAI1_FAGSY Uncharacterized protein OS=Fagus sylvatica OX=28930 GN=FSB_LOCUS42997 PE=4	[illegible]
endless_summer2_g14643	endless_summer2_g14643	Endless Summer genom	Alternative assembl	XP_028096459.1	93.2	XP_028096459.1 vacuolar protein sorting-associated protein 29 [Camellia sinensis]	[illegible]
endless_summer2_g14790	endless_summer2_g14790	Endless Summer genom	Alternative assembl	XP_016486922.1	44.9	XP_016486922.1 PREDICTED: zinc finger BED domain-containing protein RICESLEEPER 4-like [Nicotiana tabacum]	[illegible]
endless_summer2_g14862	endless_summer2_g14862	Endless Summer genom	Alternative assembl	XP_040962330.1	85.6	XP_040962330.1 tryptophan synthase beta chain 2 isoform X2 [Gossypium hirsutum]	[illegible]
endless_summer2_g14940	endless_summer2_g14940	Endless Summer genom	Alternative assembl	tr|A0A5E4G8X6|A0A5E4G8X6_PRUDU	53.4	tr|A0A5E4G8X6|A0A5E4G8X6_PRUDU RNA-directed DNA polymerase (Fragment) OS=Prunus dulcis OX=3755 GN=ALMO	[illegible]
endless_summer2_g14958	endless_summer2_g14958	Endless Summer genom	Alternative assembl	tr|A0A7J7CUH1|A0A7J7CUH1_TRIWF	85	tr|A0A7J7CUH1|A0A7J7CUH1_TRIWF Cytokinin hydroxylase OS=Tripterygium wilfordii OX=458696 GN=HS088_TW13G	[illegible]
endless_summer2_g14980	endless_summer2_g14980	Endless Summer genom	Alternative assembl	tr|A0A6V7JBP7|A0A6V7JBP7_9HYME	46.6	tr|A0A6V7JBP7|A0A6V7JBP7_9HYME Mannose-6-phosphate isomerase (Fragment) OS=Bracon brevicornis OX=1563983 GN-	[illegible]
endless_summer2_g14989	endless_summer2_g14989	Endless Summer genom	Alternative assembl	XP_010108888.2	51.8	XP_010108888.2 protein PLASTID MOVEMENT IMPAIRED 2 [Morus notabilis]	[illegible]
endless_summer2_g15031	endless_summer2_g15031	Endless Summer genom	Alternative assembl	XP_028100317.1	62.9	XP_028100317.1 helicase sen1-like isoform X2 [Camellia sinensis]	[illegible]
endless_summer2_g15068	endless_summer2_g15068	Endless Summer genom	Alternative assembl	XP_041011272.1	50.5	XP_041011272.1 uncharacterized protein LOC121255044 [Juglans microcarpa x Juglans regia]	[illegible]
endless_summer2_g15103	endless_summer2_g15103	Endless Summer genom	Alternative assembl	tr|A0A438HTF7|A0A438HTF7_VITVI	62	tr|A0A438HTF7|A0A438HTF7_VITVI Retrovirus-related Pol polyprotein from transposon 17.6 OS=Vitis vinifera OX=29760 G	[illegible]
endless_summer2_g15140	endless_summer2_g15140	Endless Summer genom	Alternative assembl	XP_028122368.1	31.5	XP_028122368.1 uncharacterized protein LOC114319545 [Camellia sinensis]	[illegible]
endless_summer2_g15182	endless_summer2_g15182	Endless Summer genom	Alternative assembl	tr|A0A6P9ELN5|A0A6P9ELN5_JUGRE	34.5	tr|A0A6P9ELN5|A0A6P9ELN5_JUGRE uncharacterized protein LOC118347670 OS=Juglans regia OX=51240 GN=LOC11834	[illegible]
endless_summer2_g15213	endless_summer2_g15213	Endless Summer genom	Alternative assembl	tr|A0A2R6PQ89|A0A2R6PQ89_ACTCC	64	tr|A0A2R6PQ89|A0A2R6PQ89_ACTCC Protein FAR1-RELATED SEQUENCE OS=Actinidia chinensis var. chinensis OX=1	[illegible]
endless_summer2_g15253	endless_summer2_g15253	Endless Summer genom	Alternative assembl	tr|A0A438CAB5|A0A438CAB5_VITVI	58.1	tr|A0A438CAB5|A0A438CAB5_VITVI Transposon Ty3-I Gag-Pol polyprotein OS=Vitis vinifera OX=29760 GN=TY3B-I_307	[illegible]
endless_summer2_g15332	endless_summer2_g15332	Endless Summer genom	Alternative assembl	tr|A0A2N9HZX1|A0A2N9HZX1_FAGSY	36.6	tr|A0A2N9HZX1|A0A2N9HZX1_FAGSY CCHC-type domain-containing protein OS=Fagus sylvatica OX=28930 GN=FSB_LO	[illegible]
endless_summer2_g15393	endless_summer2_g15393	Endless Summer genom	Alternative assembl	XP_011036080.1	44.8	XP_011036080.1 PREDICTED: uncharacterized protein LOC105133696 [Populus euphratica]	[illegible]
endless_summer2_g15408	endless_summer2_g15408	Endless Summer genom	Alternative assembl	tr|A0A5B7AWN5|A0A5B7AWN5_DAVIN	42.3	tr|A0A5B7AWN5|A0A5B7AWN5_DAVIN Uncharacterized protein (Fragment) OS=Davidia involucrata OX=16924 GN=Din_03	[illegible]
endless_summer2_g15630	endless_summer2_g15630	Endless Summer genom	Alternative assembl	XP_044441014.1	25.7	XP_044441014.1 uncharacterized protein LOC123167247 [Triticum aestivum]	[illegible]
endless_summer2_g15649	endless_summer2_g15649	Endless Summer genom	Alternative assembl	XP_028111963.1	73.4	XP_028111963.1 uncharacterized protein LOC114310209 [Camellia sinensis]	[illegible]
endless_summer2_g15714	endless_summer2_g15714	Endless Summer genom	Alternative assembl	tr|A0A5J5BPQ9|A0A5J5BPQ9_9ASTE	48.3	tr|A0A5J5BPQ9|A0A5J5BPQ9_9ASTE Uncharacterized protein OS=Nyssa sinensis OX=561372 GN=F0562_022646 PE=4 SV	[illegible]
endless_summer2_g15914	endless_summer2_g15914	Endless Summer genom	Alternative assembl	XP_020416315.1	65	XP_020416315.1 translocon-associated protein subunit beta [Prunus persica]	[illegible]
endless_summer2_g15930	endless_summer2_g15930	Endless Summer genom	Alternative assembl	tr|A0A4Q3DDF9|A0A4Q3DDF9_9RICK	31.6	tr|A0A4Q3DDF9|A0A4Q3DDF9_9RICK Reverse transcriptase-like protein (Fragment) OS=Rickettsiaceae bacterium OX=20233	[illegible]
endless_summer2_g16123	endless_summer2_g16123	Endless Summer genom	Alternative assembl	tr|A0A5J5BHT1|A0A5J5BHT1_9ASTE	55.5	tr|A0A5J5BHT1|A0A5J5BHT1_9ASTE Uncharacterized protein OS=Nyssa sinensis OX=561372 GN=F0562_023320 PE=4 SV	[illegible]
endless_summer2_g16140	endless_summer2_g16140	Endless Summer genom	Alternative assembl	XP_038723417.1	87	XP_038723417.1 callose synthase 10-like [Tripterygium wilfordii]	[illegible]
endless_summer2_g16141	endless_summer2_g16141	Endless Summer genom	Alternative assembl	tr|A0A7J0ES58|A0A7J0ES58_9ERIC	48.6	tr|A0A7J0ES58|A0A7J0ES58_9ERIC BED-type domain-containing protein OS=Actinidia rufa OX=165716 GN=Acr_06g00076	[illegible]
endless_summer2_g16263	endless_summer2_g16263	Endless Summer genom	Alternative assembl	tr|A0A2N9GT10|A0A2N9GT10_FAGSY	50.8	tr|A0A2N9GT10|A0A2N9GT10_FAGSY Retrotrans_gag domain-containing protein OS=Fagus sylvatica OX=28930 GN=FSB	[illegible]
endless_summer2_g16264	endless_summer2_g16264	Endless Summer genom	Alternative assembl	tr|A0A498JM91|A0A498JM91_MALDO	48.7	tr|A0A498JM91|A0A498JM91_MALDO Histone deacetylase OS=Malus domestica OX=3750 GN=DVH24_008685 PE=3 SV=	[illegible]
endless_summer2_g16349	endless_summer2_g16349	Endless Summer genom	Alternative assembl	tr|A0A5J5C6G7|A0A5J5C6G7_9ASTE	52	tr|A0A5J5C6G7|A0A5J5C6G7_9ASTE CCHC-type domain-containing protein OS=Nyssa sinensis OX=561372 GN=F0562_00	[illegible]
endless_summer2_g16401	endless_summer2_g16401	Endless Summer genom	Alternative assembl	XP_028121201.1	69.8	XP_028121201.1 transcription factor bHLH162-like isoform X1 [Camellia sinensis]	[illegible]
endless_summer2_g16406	endless_summer2_g16406	Endless Summer genom	Alternative assembl	XP_024021000.1	53.1	XP_024021000.1 zinc finger BED domain-containing protein RICESLEEPER 2-like [Morus notabilis]	[illegible]
endless_summer2_g16424	endless_summer2_g16424	Endless Summer genom	Alternative assembl	XP_028112913.1	27.7	XP_028112913.1 uncharacterized protein LOC114311024 [Camellia sinensis]	[illegible]
endless_summer2_g16440	endless_summer2_g16440	Endless Summer genom	Alternative assembl	XP_038981243.1	40.6	XP_038981243.1 protein ALP1-like isoform X1 [Phoenix dactylifera]	[illegible]
endless_summer2_g16584	endless_summer2_g16584	Endless Summer genom	Alternative assembl	tr|A0A7J0DDM9|A0A7J0DDM9_9ERIC	36.8	tr|A0A7J0DDM9|A0A7J0DDM9_9ERIC CCHC-type domain-containing protein OS=Actinidia rufa OX=165716 GN=Acr_00g0	[illegible]
endless_summer2_g16628	endless_summer2_g16628	Endless Summer genom	Alternative assembl	tr|A0A6D2IZ61|A0A6D2IZ61_9BRAS	57.9	tr|A0A6D2IZ61|A0A6D2IZ61_9BRAS Uncharacterized protein OS=Microthlaspi erraticum OX=1685480 GN=MERR_LOCUS1	[illegible]
endless_summer2_g16648	endless_summer2_g16648	Endless Summer genom	Alternative assembl	tr|A0A2Z7CVX2|A0A2Z7CVX2_9LAMI	47.8	tr|A0A2Z7CVX2|A0A2Z7CVX2_9LAMI Retrotran_gag_3 domain-containing protein OS=Dorcoceras hygrometricum OX=4723	[illegible]
endless_summer2_g16676	endless_summer2_g16676	Endless Summer genom	Alternative assembl	XP_021820668.1	54.3	XP_021820668.1 protein EMBRYO DEFECTIVE 514 [Prunus avium]	[illegible]
endless_summer2_g16748	endless_summer2_g16748	Endless Summer genom	Alternative assembl	tr|A0A2N9G3M9|A0A2N9G3M9_FAGSY	31.2	tr|A0A2N9G3M9|A0A2N9G3M9_FAGSY CCHC-type domain-containing protein OS=Fagus sylvatica OX=28930 GN=FSB_L	[illegible]
endless_summer2_g16973	endless_summer2_g16973	Endless Summer genom	Alternative assembl	tr|A0A5B6YZH8|A0A5B6YZH8_DAVIN	64.2	tr|A0A5B6YZH8|A0A5B6YZH8_DAVIN BED-type domain-containing protein (Fragment) OS=Davidia involucrata OX=16924	[illegible]
endless_summer2_g16997	endless_summer2_g16997	Endless Summer genom	Alternative assembl	XP_028101048.1	70.8	XP_028101048.1 uncharacterized protein LOC114300362 [Camellia sinensis]	[illegible]
endless_summer2_g17002	endless_summer2_g17002	Endless Summer genom	Alternative assembl	tr|A0A803RA90|A0A803RA90_CANSA	38.8	tr|A0A803RA90|A0A803RA90_CANSA Myb_DNA-bind_3 domain-containing protein OS=Cannabis sativa OX=3483 PE=4 S'	[illegible]
endless_summer2_g17067	endless_summer2_g17067	Endless Summer genom	Alternative assembl	XP_022724844.1	63.7	XP_022724844.1 proteasome subunit alpha type-5 isoform X2 [Durio zibethinus]	[illegible]
endless_summer2_g17151	endless_summer2_g17151	Endless Summer genom	Alternative assembl	tr|A0A5B7C0X4|A0A5B7C0X4_DAVIN	75.3	tr|A0A5B7C0X4|A0A5B7C0X4_DAVIN Uncharacterized protein OS=Davidia involucrata OX=16924 GN=Din_044260 PE=3 S	[illegible]
endless_summer2_g17374	endless_summer2_g17374	Endless Summer genom	Alternative assembl	XP_030457839.1	45.7	XP_030457839.1 uncharacterized protein LOC115678587 [Syzygium oleosum]	[illegible]
endless_summer2_g17580	endless_summer2_g17580	Endless Summer genom	Alternative assembl	tr|A0A7J0F9I8|A0A7J0F9I8_9ERIC	35.8	tr|A0A7J0F9I8|A0A7J0F9I8_9ERIC CCHC-type domain-containing protein OS=Actinidia rufa OX=165716 GN=Acr_10g00072	[illegible]
endless_summer2_g17705	endless_summer2_g17705	Endless Summer genom	Alternative assembl	tr|A0A7J0E0Y9|A0A7J0E0Y9_9ERIC	51.8	tr|A0A7J0E0Y9|A0A7J0E0Y9_9ERIC Integrase catalytic domain-containing protein OS=Actinidia rufa OX=165716 GN=Acr_00	[illegible]
endless_summer2_g17709	endless_summer2_g17709	Endless Summer genom	Alternative assembl	XP_030518623.1	49.5	XP_030518623.1 jacalin-related lectin 3-like, partial [Rhodamnia argentea]	[illegible]
endless_summer2_g17732	endless_summer2_g17732	Endless Summer genom	Alternative assembl	tr|A0A5B7APT1|A0A5B7APT1_DAVIN	57.9	tr|A0A5B7APT1|A0A5B7APT1_DAVIN Uncharacterized protein (Fragment) OS=Davidia involucrata OX=16924 GN=Din_026	[illegible]
endless_summer2_g17766	endless_summer2_g17766	Endless Summer genom	Alternative assembl	XP_015578893.1	64.7	XP_015578893.1 uncharacterized protein LOC107261783 [Ricinus communis]	[illegible]
endless_summer2_g17796	endless_summer2_g17796	Endless Summer genom	Alternative assembl	tr|A0A7K1IL06|A0A7K1IL06_9ACTN	83.8	tr|A0A7K1IL06|A0A7K1IL06_9ACTN Hsp70 family protein (Fragment) OS=Nitriliruptoraceae bacterium ZYF776 OX=260468	[illegible]
endless_summer2_g17920	endless_summer2_g17920	Endless Summer genom	Alternative assembl	tr|A0A7J0GN79|A0A7J0GN79_9ERIC	37.5	tr|A0A7J0GN79|A0A7J0GN79_9ERIC CCHC-type domain-containing protein OS=Actinidia rufa OX=165716 GN=Acr_23g000	[illegible]
endless_summer2_g1798	endless_summer2_g1798	Endless Summer genom	Alternative assembl	tr|A0A2N9II65|A0A2N9II65_FAGSY	49.4	tr|A0A2N9II65|A0A2N9II65_FAGSY Integrase catalytic domain-containing protein OS=Fagus sylvatica OX=28930 GN=FSB_L	[illegible]
endless_summer2_g18065	endless_summer2_g18065	Endless Summer genom	Alternative assembl	tr|A0A2N9H6R8|A0A2N9H6R8_FAGSY	65.2	tr|A0A2N9H6R8|A0A2N9H6R8_FAGSY Uncharacterized protein OS=Fagus sylvatica OX=28930 GN=FSB_LOCUS38028 PE-	[illegible]
endless_summer2_g18113	endless_summer2_g18113	Endless Summer genom	Alternative assembl	tr|A0A4Q3DG62|A0A4Q3DG62_9RICK	57.3	tr|A0A4Q3DG62|A0A4Q3DG62_9RICK Reverse transcriptase Ty1/copia-type domain-containing protein OS=Rickettsiaceae bac	[illegible]
endless_summer2_g18459	endless_summer2_g18459	Endless Summer genom	Alternative assembl	tr|A0A2N9FXX7|A0A2N9FXX7_FAGSY	47.3	tr|A0A2N9FXX7|A0A2N9FXX7_FAGSY CCHC-type domain-containing protein OS=Fagus sylvatica OX=28930 GN=FSB_LO	[illegible]
endless_summer2_g18530	endless_summer2_g18530	Endless Summer genom	Alternative assembl	XP_028105120.1	35.1	XP_028105120.1 uncharacterized protein LOC114304166 [Camellia sinensis]	[illegible]
endless_summer2_g18567	endless_summer2_g18567	Endless Summer genom	Alternative assembl	tr|K1KXG9|K1KXG9_9BACT	53.4	tr|K1KXG9|K1KXG9_9BACT Integrase catalytic domain-containing protein OS=Cecembia lonarensis LW9 OX=1225176 GN=1	[illegible]
endless_summer2_g1867	endless_summer2_g1867	Endless Summer genom	Alternative assembl	XP_028106172.1	46.7	XP_028106172.1 uncharacterized protein LOC114305297 [Camellia sinensis]	[illegible]
endless_summer2_g18672	endless_summer2_g18672	Endless Summer genom	Alternative assembl	tr|A0A5B7C6Y1|A0A5B7C6Y1_DAVIN	65.3	tr|A0A5B7C6Y1|A0A5B7C6Y1_DAVIN Putative disease resistance RPP13-like protein 1 (Fragment) OS=Davidia involucrata C	[illegible]
endless_summer2_g18679	endless_summer2_g18679	Endless Summer genom	Alternative assembl	XP_012078838.1	48.2	XP_012078838.1 putative disease resistance RPP13-like protein 1 [Jatropha curcas]	[illegible]
endless_summer2_g18693	endless_summer2_g18693	Endless Summer genom	Alternative assembl	tr|A0A6P9F3D4|A0A6P9F3D4_JUGRE	45.3	tr|A0A6P9F3D4|A0A6P9F3D4_JUGRE ATP-dependent DNA helicase OS=Juglans regia OX=51240 GN=LOC118349287 PE=	[illegible]
endless_summer2_g18994	endless_summer2_g18994	Endless Summer genom	Alternative assembl	tr|A0A2N9FL78|A0A2N9FL78_FAGSY	60.1	tr|A0A2N9FL78|A0A2N9FL78_FAGSY Integrase catalytic domain-containing protein OS=Fagus sylvatica OX=28930 GN=FSE	[illegible]
endless_summer2_g19001	endless_summer2_g19001	Endless Summer genom	Alternative assembl	XP_015055807.1	67	XP_015055807.1 basic 30 kDa endochitinase [Solanum pennellii]	[illegible]
endless_summer2_g19003	endless_summer2_g19003	Endless Summer genom	Alternative assembl	tr|Q4JLT7|Q4JLT7_CAPAN	76	tr|Q4JLT7|Q4JLT7_CAPAN Endochitinase (Fragment) OS=Capsicum annuum OX=4072 PE=2 SV=1	[illegible]
endless_summer2_g1912	endless_summer2_g1912	Endless Summer genom	Alternative assembl	tr|A0A7J0H061|A0A7J0H061_9ERIC	32.2	tr|A0A7J0H061|A0A7J0H061_9ERIC Retrotrans_gag domain-containing protein OS=Actinidia rufa OX=165716 GN=Acr_25g0(	[illegible]
endless_summer2_g19197	endless_summer2_g19197	Endless Summer genom	Alternative assembl	XP_027106952.1	49.6	XP_027106952.1 putative disease resistance RPP13-like protein 1 [Coffea arabica]	[illegible]
endless_summer2_g19298	endless_summer2_g19298	Endless Summer genom	Alternative assembl	XP_028091438.1	74.8	XP_028091438.1 protein-lysine N-methyltransferase EFM1 isoform X2 [Camellia sinensis]	[illegible]
endless_summer2_g19309	endless_summer2_g19309	Endless Summer genom	Alternative assembl	XP_021289258.1	63.1	XP_021289258.1 cactin [Herrania umbratica]	[illegible]
endless_summer2_g19377	endless_summer2_g19377	Endless Summer genom	Alternative assembl	XP_028058223.1	42.4	XP_028058223.1 uncharacterized protein LOC114262079 isoform X1 [Camellia sinensis]	[illegible]
endless_summer2_g19407	endless_summer2_g19407	Endless Summer genom	Alternative assembl	XP_028071247.1	42.2	XP_028071247.1 putative nuclease HARBI1 [Camellia sinensis]	[illegible]
endless_summer2_g19486	endless_summer2_g19486	Endless Summer genom	Alternative assembl	XP_028089459.1	56.3	XP_028089459.1 uncharacterized protein LOC114289837 [Camellia sinensis]	[illegible]
endless_summer2_g19555	endless_summer2_g19555	Endless Summer genom	Alternative assembl	tr|A0A5B6ZUT8|A0A5B6ZUT8_DAVIN	55.7	tr|A0A5B6ZUT8|A0A5B6ZUT8_DAVIN Uncharacterized protein OS=Davidia involucrata OX=16924 GN=Din_017550 PE=4 S	[illegible]
endless_summer2_g19664	endless_summer2_g19664	Endless Summer genom	Alternative assembl	XP_018836180.1	56.7	XP_018836180.1 vacuolar protein-sorting-associated protein 37 homolog 1-like [Juglans regia]	[illegible]
endless_summer2_g19700	endless_summer2_g19700	Endless Summer genom	Alternative assembl	tr|A0A834GAV9|A0A834GAV9_RHOSS	62.7	tr|A0A834GAV9|A0A834GAV9_RHOSS Uncharacterized protein OS=Rhododendron simsii OX=118357 GN=RHSIM_Rhsim1	[illegible]
endless_summer2_g1977	endless_summer2_g1977	Endless Summer genom	Alternative assembl	tr|A0A7J9A3B9|A0A7J9A3B9_9ROSI	72.3	tr|A0A7J9A3B9|A0A7J9A3B9_9ROSI Fe2OG dioxygenase domain-containing protein OS=Gossypium laxum OX=34288 GN=	[illegible]
endless_summer2_g19879	endless_summer2_g19879	Endless Summer genom	Alternative assembl	XP_022159216.1	58.9	XP_022159216.1 uncharacterized protein LOC111025635 [Momordica charantia]	[illegible]
endless_summer2_g20005	endless_summer2_g20005	Endless Summer genom	Alternative assembl	XP_028055845.1	44.8	XP_028055845.1 protein LNK1-like isoform X2 [Camellia sinensis]	[illegible]
endless_summer2_g20020	endless_summer2_g20020	Endless Summer genom	Alternative assembl	tr|A0A7J7G403|A0A7J7G403_CAMSI	39.4	tr|A0A7J7G403|A0A7J7G403_CAMSI DBD_Tnp_Mut domain-containing protein OS=Camellia sinensis OX=4442 GN=HYC8	[illegible]
endless_summer2_g20035	endless_summer2_g20035	Endless Summer genom	Alternative assembl	tr|A0A7J7FX79|A0A7J7FX79_CAMSI	31.2	tr|A0A7J7FX79|A0A7J7FX79_CAMSI SWIM-type domain-containing protein OS=Camellia sinensis OX=4442 GN=HYC85_0	[illegible]
endless_summer2_g2013	endless_summer2_g2013	Endless Summer genom	Alternative assembl	tr|A0A834GAD9|A0A834GAD9_RHOSS	49.4	tr|A0A834GAD9|A0A834GAD9_RHOSS ULP_PROTEASE domain-containing protein OS=Rhododendron simsii OX=118357	[illegible]
endless_summer2_g20130	endless_summer2_g20130	Endless Summer genom	Alternative assembl	XP_028093741.1	65.2	XP_028093741.1 L-type lectin-domain containing receptor kinase IX.1-like [Camellia sinensis]	[illegible]
endless_summer2_g20271	endless_summer2_g20271	Endless Summer genom	Alternative assembl	tr|A0A5B7A9C3|A0A5B7A9C3_DAVIN	36.6	tr|A0A5B7A9C3|A0A5B7A9C3_DAVIN ATP-dependent DNA helicase OS=Davidia involucrata OX=16924 GN=Din_021512 P	[illegible]
endless_summer2_g20303	endless_summer2_g20303	Endless Summer genom	Alternative assembl	XP_039778092.1	53.7	XP_039778092.1 protein ALP1-like [Panicum virgatum]	[illegible]
endless_summer2_g20395	endless_summer2_g20395	Endless Summer genom	Alternative assembl	tr|A0A7J0DUL2|A0A7J0DUL2_9ERIC	82.6	tr|A0A7J0DUL2|A0A7J0DUL2_9ERIC Uncharacterized protein OS=Actinidia rufa OX=165716 GN=Acr_00g0082480 PE=4 SV	[illegible]
endless_summer2_g20676	endless_summer2_g20676	Endless Summer genom	Alternative assembl	tr|A0A2I4FBZ1|A0A2I4FBZ1_JUGRE	76.6	tr|A0A2I4FBZ1|A0A2I4FBZ1_JUGRE brefeldin A-inhibited guanine nucleotide-exchange protein 1-like isoform X1 OS=Juglans	[illegible]
endless_summer2_g20747	endless_summer2_g20747	Endless Summer genom	Alternative assembl	XP_038709524.1	47.6	XP_038709524.1 uncharacterized protein LOC120004292 [Tripterygium wilfordii]	[illegible]
endless_summer2_g20856	endless_summer2_g20856	Endless Summer genom	Alternative assembl	tr|A0A5B6YYS9|A0A5B6YYS9_DAVIN	70.8	tr|A0A5B6YYS9|A0A5B6YYS9_DAVIN Uncharacterized protein (Fragment) OS=Davidia involucrata OX=16924 GN=Din_006	[illegible]
endless_summer2_g20926	endless_summer2_g20926	Endless Summer genom	Alternative assembl	XP_028115326.1	65.3	XP_028115326.1 UDP-glycosyltransferase 74E2-like [Camellia sinensis]	[illegible]
endless_summer2_g21028	endless_summer2_g21028	Endless Summer genom	Alternative assembl	XP_012859009.1	58.5	XP_012859009.1 PREDICTED: putative nuclease HARBI1 [Erythranthe guttata]	[illegible]
endless_summer2_g21061	endless_summer2_g21061	Endless Summer genom	Alternative assembl	tr|A0A7J0HA58|A0A7J0HA58_9ERIC	68.4	tr|A0A7J0HA58|A0A7J0HA58_9ERIC EF-TU receptor OS=Actinidia rufa OX=165716 GN=Acr_28g0003190 PE=3 SV=1	[illegible]
endless_summer2_g21085	endless_summer2_g21085	Endless Summer genom	Alternative assembl	tr|D7RPB4|D7RPB4_FUSSL	34.9	tr|D7RPB4|D7RPB4_FUSSL Taxadiene synthase (Fragment) OS=Fusarium solani OX=169388 GN=TDS PE=2 SV=1	[illegible]
endless_summer2_g2109	endless_summer2_g2109	Endless Summer genom	Alternative assembl	XP_023914200.1	64.8	XP_023914200.1 ATP-dependent DNA helicase PIF1-like [Quercus suber]	[illegible]
endless_summer2_g21191	endless_summer2_g21191	Endless Summer genom	Alternative assembl	tr|A0A392M2B5|A0A392M2B5_9FABA	28.7	tr|A0A392M2B5|A0A392M2B5_9FABA RNA recognition motif OS=Trifolium medium OX=97028 GN=A2U01_0002227 PE-	[illegible]
endless_summer2_g21199	endless_summer2_g21199	Endless Summer genom	Alternative assembl	tr|A0A834LZ17|A0A834LZ17_RHOSS	62.2	tr|A0A834LZ17|A0A834LZ17_RHOSS Retrotran_gag_3 domain-containing protein OS=Rhododendron simsii OX=118357 GN	[illegible]
endless_summer2_g21273	endless_summer2_g21273	Endless Summer genom	Alternative assembl	XP_022875593.1	67.2	XP_022875593.1 cytochrome P450 71B10-like [Olea europaea var. sylvestris]	[illegible]
endless_summer2_g21279	endless_summer2_g21279	Endless Summer genom	Alternative assembl	tr|A0A7J8XN10|A0A7J8XN10_GOSAI	77.5	tr|A0A7J8XN10|A0A7J8XN10_GOSAI 4-hydroxy-tetrahydrodipicolinate synthase OS=Gossypium aridum OX=34290 GN=Goar	[illegible]
endless_summer2_g21349	endless_summer2_g21349	Endless Summer genom	Alternative assembl	tr|A0A4S4DBI9|A0A4S4DBI9_CAMSI	35.1	tr|A0A4S4DBI9|A0A4S4DBI9_CAMSI Bromo domain-containing protein OS=Camellia sinensis var. sinensis OX=542762 GN	[illegible]
endless_summer2_g21383	endless_summer2_g21383	Endless Summer genom	Alternative assembl	XP_042980491.1	55.5	XP_042980491.1 protein ALP1-like [Carya illinoinensis]	[illegible]
endless_summer2_g21579	endless_summer2_g21579	Endless Summer genom	Alternative assembl	XP_030937790.1	77.8	XP_030937790.1 2-alkenal reductase (NADP(+)-dependent)-like isoform X2 [Quercus lobata]	[illegible]
endless_summer2_g21632	endless_summer2_g21632	Endless Summer genom	Alternative assembl	XP_034683171.1	51	XP_034683171.1 protein PLASTID MOVEMENT IMPAIRED 1-RELATED 1-like isoform X1 [Vitis riparia]	[illegible]
endless_summer2_g21700	endless_summer2_g21700	Endless Summer genom	Alternative assembl	tr|A0A5B7BEF1|A0A5B7BEF1_DAVIN	45.7	tr|A0A5B7BEF1|A0A5B7BEF1_DAVIN Phorbol-ester/DAG-type domain-containing protein (Fragment) OS=Davidia involucrat	[illegible]
endless_summer2_g21860	endless_summer2_g21860	Endless Summer genom	Alternative assembl	tr|A0A445J4A7|A0A445J4A7_GLYSO	58.8	tr|A0A445J4A7|A0A445J4A7_GLYSO Uncharacterized protein OS=Glycine soja OX=3848 GN=D0Y65_024877 PE=4 SV=1	[illegible]
endless_summer2_g21910	endless_summer2_g21910	Endless Summer genom	Alternative assembl	XP_034679204.1	75	XP_034679204.1 calcium homeostasis endoplasmic reticulum protein-like isoform X2 [Vitis riparia]	[illegible]
endless_summer2_g22511	endless_summer2_g22511	Endless Summer genom	Alternative assembl	tr|A0A5B7AEQ3|A0A5B7AEQ3_DAVIN	75.8	tr|A0A5B7AEQ3|A0A5B7AEQ3_DAVIN Uncharacterized protein OS=Davidia involucrata OX=16924 GN=Din_024205 PE=4 S	[illegible]
endless_summer2_g22561	endless_summer2_g22561	Endless Summer genom	Alternative assembl	XP_028081457.1	66.5	XP_028081457.1 uncharacterized protein LOC114282883 [Camellia sinensis]	[illegible]
endless_summer2_g22620	endless_summer2_g22620	Endless Summer genom	Alternative assembl	tr|A0A5B6YMF9|A0A5B6YMF9_DAVIN	47.9	tr|A0A5B6YMF9|A0A5B6YMF9_DAVIN NB-ARC domain-containing protein (Fragment) OS=Davidia involucrata OX=16924	[illegible]
endless_summer2_g22627	endless_summer2_g22627	Endless Summer genom	Alternative assembl	tr|V5UN18|V5UN18_CICAR	51.1	tr|V5UN18|V5UN18_CICAR CC-NBS-LRR disease resistance protein OS=Cicer arietinum OX=3827 PE=4 SV=1	[illegible]
endless_summer2_g22628	endless_summer2_g22628	Endless Summer genom	Alternative assembl	XP_038704662.1	74.9	XP_038704662.1 putative nuclease HARBI1 [Tripterygium wilfordii]	[illegible]
endless_summer2_g22671	endless_summer2_g22671	Endless Summer genom	Alternative assembl	XP_024441023.1	40	XP_024441023.1 uncharacterized protein LOC18105336 [Populus trichocarpa]	[illegible]
endless_summer2_g2272	endless_summer2_g2272	Endless Summer genom	Alternative assembl	tr|W1PQ40|W1PQ40_AMBTC	29.1	tr|W1PQ40|W1PQ40_AMBTC Uncharacterized protein OS=Amborella trichopoda OX=13333 GN=AMTR_s00168p00019630 I	[illegible]
endless_summer2_g22987	endless_summer2_g22987	Endless Summer genom	Alternative assembl	tr|A0A5J5U8A9|A0A5J5U8A9_GOSBA	26.8	tr|A0A5J5U8A9|A0A5J5U8A9_GOSBA RRM domain-containing protein (Fragment) OS=Gossypium barbadense OX=3634 GN	[illegible]
endless_summer2_g2306	endless_summer2_g2306	Endless Summer genom	Alternative assembl	tr|A0A7J6WH99|A0A7J6WH99_THATH	37.3	tr|A0A7J6WH99|A0A7J6WH99_THATH Uncharacterized protein OS=Thalictrum thalictroides OX=46969 GN=FRX31_01412	[illegible]

endless_summer2_g23210	endless_summer2_g23210	Endless Summer genom	Alternative assembl	tr|A0A3S4P1Q0|A0A3S4P1Q0_9MAGN	25.2	tr|A0A3S4P1Q0|A0A3S4P1Q0_9MAGN Bromodomain-containing protein 4-like protein isoform X3 OS=Cinnamomum micran	cinnamomum micranthum f.	4577.GRMZM2G315394_P01	[illegible]	[illegible]
endless_summer2_g23212	endless_summer2_g23212	Endless Summer genom	Alternative assembl	tr|A0A835IPT6|A0A835IPT6_9MAGN	33.3	tr|A0A835IPT6|A0A835IPT6_9MAGN Transpos_assoc domain-containing protein OS=Coptis chinensis OX=261450 GN=IFM	coptis chinensis	39947.LOC_Os08g30250.1	[illegible]	[illegible]
endless_summer2_g23250	endless_summer2_g23250	Endless Summer genom	Alternative assembl	XP_040867755.1	83.2	XP_040867755.1 secreted RxLR effector protein 161-like [Glycine max]	glycine max	39947.LOC_Os07g22810.1	[illegible]	[illegible]
endless_summer2_g23276	endless_summer2_g23276	Endless Summer genom	Alternative assembl	tr|A0A5B7BGR8|A0A5B7BGR8_DAVIN	55.8	tr|A0A5B7BGR8|A0A5B7BGR8_DAVIN Lectin_legB domain-containing protein OS=Davidia involucrata OX=16924 GN=Din	davidia involucrata	3847.GLYMA07G30260.1	[illegible]	[illegible]
endless_summer2_g23290	endless_summer2_g23290	Endless Summer genom	Alternative assembl	tr|C3ZAR5|C3ZAR5_BRAFL	31.6	tr|C3ZAR5|C3ZAR5_BRAFL Uncharacterized protein (Fragment) OS=Branchiostoma floridae OX=7739 GN=BRAFLDRAFT	branchiostoma floridae	29760.VIT_08s0007g09040.t01	[illegible]	[illegible]
endless_summer2_g23301	endless_summer2_g23301	Endless Summer genom	Alternative assembl	XP_028091758.1	82.6	XP_028091758.1 methionine gamma-lyase-like [Camellia sinensis]	camellia sinensis	29760.VIT_02s0025g02560.t01	[illegible]	[illegible]
endless_summer2_g23306	endless_summer2_g23306	Endless Summer genom	Alternative assembl	XP_017617003.1	41	XP_017617003.1 PREDICTED: GDT1-like protein 4 isoform X2 [Gossypium arboreum]	gossypium arboreum	4113.PGSC0003DMT400002167	[illegible]	[illegible]
endless_summer2_g23384	endless_summer2_g23384	Endless Summer genom	Alternative assembl	tr|A0A6V7NYX3|A0A6V7NYX3_ANACO	50	tr|A0A6V7NYX3|A0A6V7NYX3_ANACO SWIM-type domain-containing protein OS=Ananas comosus var. bracteatus OX=29(	ananas comosus var. bracte	39947.LOC_Os03g41570.1	[illegible]	[illegible]
endless_summer2_g23430	endless_summer2_g23430	Endless Summer genom	Alternative assembl	XP_028096025.1	46.1	XP_028096025.1 PKS-NRPS hybrid synthetase CHGG_01239-like [Camellia sinensis]	camellia sinensis	29760.VIT_03s0038g03060.t01	[illegible]	[illegible]
endless_summer2_g23717	endless_summer2_g23717	Endless Summer genom	Alternative assembl	tr|A0A4Q3EAQ4|A0A4Q3EAQ4_9SPHI	39.7	tr|A0A4Q3EAQ4|A0A4Q3EAQ4_9SPHI Retrotrans_gag domain-containing protein (Fragment) OS=Sphingobacteriaceae bacteriu	sphingobacteriaceae bacteri	39947.LOC_Os12g24920.1	[illegible]	[illegible]
endless_summer2_g24062	endless_summer2_g24062	Endless Summer genom	Alternative assembl	XP_028088941.1	61.4	XP_028088941.1 E3 ubiquitin-protein ligase RMA1H1-like [Camellia sinensis]	camellia sinensis	4081.Solyc02g078320.1.1	[illegible]	[illegible]
endless_summer2_g24134	endless_summer2_g24134	Endless Summer genom	Alternative assembl	XP_038970678.1	36.9	XP_038970678.1 60 kDa jasmonate-induced protein-like [Phoenix dactylifera]	phoenix dactylifera	148304.MAPG_11083T0	[illegible]	[illegible]
endless_summer2_g24191	endless_summer2_g24191	Endless Summer genom	Alternative assembl	tr|A0A835CE06|A0A835CE06_9FABA	28.1	tr|A0A835CE06|A0A835CE06_9FABA Reverse transcriptase OS=Senna tora OX=362788 GN=G2W53_007773 PE=4 SV=1	senna tora	39947.LOC_Os01g06700.1	[illegible]	[illegible]
endless_summer2_g24278	endless_summer2_g24278	Endless Summer genom	Alternative assembl	XP_028113968.1	82.2	XP_028113968.1 protein TRANSPORT INHIBITOR RESPONSE 1-like [Camellia sinensis]	camellia sinensis	29760.VIT_00s0181g00040.t01	[illegible]	[illegible]
endless_summer2_g24322	endless_summer2_g24322	Endless Summer genom	Alternative assembl	XP_028091263.1	40.8	XP_028091263.1 uncharacterized protein LOC114291609 [Camellia sinensis]	camellia sinensis	39947.LOC_Os10g11250.1	[illegible]	[illegible]
endless_summer2_g24331	endless_summer2_g24331	Endless Summer genom	Alternative assembl	tr|A0A5J5A4E6|A0A5J5A4E6_9ASTE	35.1	tr|A0A5J5A4E6|A0A5J5A4E6_9ASTE Uncharacterized protein OS=Nyssa sinensis OX=561372 GN=F0562_011338 PE=4 SV=	nyssa sinensis	4577.GRMZM2G384011_P01	[illegible]	[illegible]
endless_summer2_g24366	endless_summer2_g24366	Endless Summer genom	Alternative assembl	XP_027099125.1	31.9	XP_027099125.1 uncharacterized protein LOC113718416 [Coffea arabica]	coffea arabica	29760.VIT_00s0476g00020.t01	[illegible]	[illegible]
endless_summer2_g24373	endless_summer2_g24373	Endless Summer genom	Alternative assembl	tr|A0A3S3NSJ7|A0A3S3NSJ7_9MAGN	37.2	tr|A0A3S3NSJ7|A0A3S3NSJ7_9MAGN Uncharacterized protein OS=Cinnamomum micranthum f. kanehirae OX=337451 GN=(	cinnamomum micranthum f.	3847.GLYMA05G29901.1	[illegible]	[illegible]
endless_summer2_g2450	endless_summer2_g2450	Endless Summer genom	Alternative assembl	XP_024452079.1	58.5	XP_024452079.1 uncharacterized protein At4g22758 [Populus trichocarpa]	populus trichocarpa	29760.VIT_02s0025g05170.t01	[illegible]	[illegible]
endless_summer2_g24514	endless_summer2_g24514	Endless Summer genom	Alternative assembl	tr|A0A6A4MBT9|A0A6A4MBT9_9ERIC	23.9	tr|A0A6A4MBT9|A0A6A4MBT9_9ERIC ULP_PROTEASE domain-containing protein (Fragment) OS=Rhododendron william	rhododendron williamsianum		[illegible]	[illegible]
endless_summer2_g24573	endless_summer2_g24573	Endless Summer genom	Alternative assembl	XP_010271443.1	56.5	XP_010271443.1 PREDICTED: uncharacterized protein At2g29880-like [Nelumbo nucifera]	nelumbo nucifera	29760.VIT_03s0038g02150.t01	[illegible]	[illegible]
endless_summer2_g24743	endless_summer2_g24743	Endless Summer genom	Alternative assembl	XP_028078467.1	32.7	XP_028078467.1 uncharacterized protein LOC114280312 isoform X1 [Camellia sinensis]	camellia sinensis	3847.GLYMA05G29901.1	[illegible]	[illegible]
endless_summer2_g24745	endless_summer2_g24745	Endless Summer genom	Alternative assembl	tr|A0A7J0DE87|A0A7J0DE87_9ERIC	39.9	tr|A0A7J0DE87|A0A7J0DE87_9ERIC DUF4218 domain-containing protein OS=Actinidia rufa OX=165716 GN=Acr_00g00284	actinidia rufa	15368.BRADI3G32920.1	[illegible]	[illegible]
endless_summer2_g24747	endless_summer2_g24747	Endless Summer genom	Alternative assembl	tr|A0A834HE02|A0A834HE02_RHOSS	26.7	tr|A0A834HE02|A0A834HE02_RHOSS Uncharacterized protein OS=Rhododendron simsii OX=118357 GN=RHSIM_Rhsim02	rhododendron simsii	39947.LOC_Os04g33010.1	[illegible]	[illegible]
endless_summer2_g24817	endless_summer2_g24817	Endless Summer genom	Alternative assembl	tr|A0A5B7AIL2|A0A5B7AIL2_DAVIN	90.5	tr|A0A5B7AIL2|A0A5B7AIL2_DAVIN RING-type domain-containing protein OS=Davidia involucrata OX=16924 GN=Din_02	davidia involucrata	29760.VIT_17s0000g04160.t01	[illegible]	[illegible]
endless_summer2_g24852	endless_summer2_g24852	Endless Summer genom	Alternative assembl	tr|A0A5B7C0W0|A0A5B7C0W0_DAVIN	61.9	tr|A0A5B7C0W0|A0A5B7C0W0_DAVIN DBD_Tnp_Mut domain-containing protein (Fragment) OS=Davidia involucrata OX=	davidia involucrata	39947.LOC_Os08g25960.1	[illegible]	[illegible]
endless_summer2_g24853	endless_summer2_g24853	Endless Summer genom	Alternative assembl	XP_028059754.1	78.6	XP_028059754.1 anthranilate phosphoribosyltransferase, chloroplastic-like isoform X1 [Camellia sinensis]	camellia sinensis	29760.VIT_17s0000g08660.t01	[illegible]	[illegible]
endless_summer2_g25013	endless_summer2_g25013	Endless Summer genom	Alternative assembl	XP_019265189.1	31.6	XP_019265189.1 PREDICTED: uncharacterized protein LOC109242783 [Nicotiana attenuata]	nicotiana attenuata	29760.VIT_05s0049g01920.t01	[illegible]	[illegible]
endless_summer2_g25052	endless_summer2_g25052	Endless Summer genom	Alternative assembl	XP_028110386.1	39.4	XP_028110386.1 uncharacterized protein LOC114308894 [Camellia sinensis]	camellia sinensis	29760.VIT_12s0035g01470.t01	[illegible]	[illegible]
endless_summer2_g25104	endless_summer2_g25104	Endless Summer genom	Alternative assembl	XP_021804117.1	43.2	XP_021804117.1 uncharacterized protein LOC110748445, partial [Prunus avium]	prunus avium	39947.LOC_Os07g16750.1	[illegible]	[illegible]
endless_summer2_g25108	endless_summer2_g25108	Endless Summer genom	Alternative assembl	XP_028105301.1	51.4	XP_028105301.1 putative late blight resistance protein homolog R1A-3, partial [Camellia sinensis]	camellia sinensis	4113.PGSC0003DMT400088449	[illegible]	[illegible]
endless_summer2_g25183	endless_summer2_g25183	Endless Summer genom	Alternative assembl	XP_028076660.1	68	XP_028076660.1 putative disease resistance protein RGA3 isoform X1 [Camellia sinensis]	camellia sinensis	4081.Solyc12g099040.1.1	[illegible]	[illegible]
endless_summer2_g25219	endless_summer2_g25219	Endless Summer genom	Alternative assembl	tr|A0A2N9IJM5|A0A2N9IJM5_FAGSY	46	tr|A0A2N9IJM5|A0A2N9IJM5_FAGSY RNase H type-1 domain-containing protein OS=Fagus sylvatica OX=28930 GN=FSB	fagus sylvatica	39947.LOC_Os03g36220.1	[illegible]	[illegible]
endless_summer2_g2522	endless_summer2_g2522	Endless Summer genom	Alternative assembl	XP_028063286.1	85.8	XP_028063286.1 hydroxymethylglutaryl-CoA synthase-like [Camellia sinensis]	camellia sinensis	29760.VIT_02s0025g04580.t01	[illegible]	[illegible]
endless_summer2_g2538	endless_summer2_g2538	Endless Summer genom	Alternative assembl	tr|A0A834ZU59|A0A834ZU59_9MAGN	26.9	tr|A0A834ZU59|A0A834ZU59_9MAGN PMD domain-containing protein OS=Tetracentron sinense OX=13715 GN=HHK36_00	tetracentron sinense	4113.PGSC0003DMT400035683	[illegible]	[illegible]
endless_summer2_g25443	endless_summer2_g25443	Endless Summer genom	Alternative assembl	XP_040991977.1	72.7	XP_040991977.1 plastid-lipid-associated protein 6, chloroplastic isoform X1 [Juglans microcarpa x Juglans regia]	juglans microcarpa x juglar	29760.VIT_05s0049g01360.t01	[illegible]	[illegible]
endless_summer2_g25451	endless_summer2_g25451	Endless Summer genom	Alternative assembl	XP_010650126.1	42.5	XP_010650126.1 PREDICTED: splicing factor U2af large subunit B [Vitis vinifera]	vitis vinifera	29760.VIT_05s0049g01490.t01	[illegible]	[illegible]
endless_summer2_g25470	endless_summer2_g25470	Endless Summer genom	Alternative assembl	tr|A0A5J5C1Z7|A0A5J5C1Z7_9ASTE	75.7	tr|A0A5J5C1Z7|A0A5J5C1Z7_9ASTE Amino_oxidase domain-containing protein OS=Nyssa sinensis OX=561372 GN=F0562	nyssa sinensis	29760.VIT_05s0049g01650.t01	[illegible]	[illegible]
endless_summer2_g25501	endless_summer2_g25501	Endless Summer genom	Alternative assembl	tr|R6R8D7|R6R8D7_9FIRM	29.4	tr|R6R8D7|R6R8D7_9FIRM Uncharacterized protein OS=Firmicutes bacterium CAG:424 OX=1263022 GN=BN652_01017 PE	firmicutes bacterium cag:42	4113.PGSC0003DMT400044378	[illegible]	[illegible]
endless_summer2_g25539	endless_summer2_g25539	Endless Summer genom	Alternative assembl	tr|A0A438HZJ5|A0A438HZJ5_VITVI	57.9	tr|A0A438HZJ5|A0A438HZJ5_VITVI Retrovirus-related Pol polyprotein from transposon 17.6 OS=Vitis vinifera OX=29760 GN	vitis vinifera	39947.LOC_Os10g21350.1	[illegible]	[illegible]
endless_summer2_g25562	endless_summer2_g25562	Endless Summer genom	Alternative assembl	XP_028122209.1	69	XP_028122209.1 1-aminocyclopropane-1-carboxylate oxidase homolog 1-like isoform X1 [Camellia sinensis]	camellia sinensis	29760.VIT_05s0049g00310.t01	[illegible]	[illegible]
endless_summer2_g25705	endless_summer2_g25705	Endless Summer genom	Alternative assembl	XP_028072658.1	35.7	XP_028072658.1 protein ACCELERATED CELL DEATH 6-like [Camellia sinensis]	camellia sinensis	29760.VIT_05s0029g01420.t01	[illegible]	[illegible]
endless_summer2_g25836	endless_summer2_g25836	Endless Summer genom	Alternative assembl	XP_018853006.2	35.8	XP_018853006.2 uncharacterized protein LOC109014969 [Juglans regia]	juglans regia	4558.Sb0010s007680.1	[illegible]	[illegible]
endless_summer2_g25906	endless_summer2_g25906	Endless Summer genom	Alternative assembl	tr|A0A6A4KLT0|A0A6A4KLT0_9ERIC	29	tr|A0A6A4KLT0|A0A6A4KLT0_9ERIC PMD domain-containing protein (Fragment) OS=Rhododendron williamsianum OX=2	rhododendron williamsiani	4081.Solyc00g020540.1.1	[illegible]	[illegible]
endless_summer2_g26049	endless_summer2_g26049	Endless Summer genom	Alternative assembl	tr|A0A834L9T4|A0A834L9T4_RHOSS	37	tr|A0A834L9T4|A0A834L9T4_RHOSS GRF-type domain-containing protein OS=Rhododendron simsii OX=118357 GN=RHS	rhododendron simsii	4081.Solyc03g122220.1.1	[illegible]	[illegible]
endless_summer2_g26143	endless_summer2_g26143	Endless Summer genom	Alternative assembl	XP_028064203.1	55.2	XP_028064203.1 uncharacterized protein LOC114267363 [Camellia sinensis]	camellia sinensis	29760.VIT_05s0020g00430.t01	[illegible]	[illegible]
endless_summer2_g26326	endless_summer2_g26326	Endless Summer genom	Alternative assembl	XP_030944966.1	41	XP_030944966.1 uncharacterized protein LOC115969446 [Quercus lobata]	quercus lobata	29760.VIT_19s0027g01660.t01	[illegible]	[illegible]
endless_summer2_g26726	endless_summer2_g26726	Endless Summer genom	Alternative assembl	tr|A0A5C6X119|A0A5C6X119_9DELT	34.9	tr|A0A5C6X119|A0A5C6X119_9DELT Ribonuclease HI family protein (Fragment) OS=Bradymonadales bacterium TMQ4 OX=	bradymonadales bacterium	4113.PGSC0003DMT400038548	[illegible]	[illegible]
endless_summer2_g26828	endless_summer2_g26828	Endless Summer genom	Alternative assembl	XP_047337186.1	26.2	XP_047337186.1 uncharacterized protein LOC124940699 [Impatiens glandulifera]	impatiens glandulifera		[illegible]	[illegible]
endless_summer2_g26880	endless_summer2_g26880	Endless Summer genom	Alternative assembl	tr|A0A438EKS2|A0A438EKS2_VITVI	71.2	tr|A0A438EKS2|A0A438EKS2_VITVI Transposon Ty3-I Gag-Pol polyprotein OS=Vitis vinifera OX=29760 GN=TY3B-I_781	vitis vinifera	3694.POPTR_0011s01230.1	[illegible]	[illegible]
endless_summer2_g26951	endless_summer2_g26951	Endless Summer genom	Alternative assembl	tr|A0A5N6NMY9|A0A5N6NMY9_9ASTR	52.2	tr|A0A5N6NMY9|A0A5N6NMY9_9ASTR Uncharacterized protein OS=Mikania micrantha OX=192012 GN=E3N88_21540 PE	mikania micrantha	4081.Solyc03g096270.1.1	[illegible]	[illegible]
endless_summer2_g26982	endless_summer2_g26982	Endless Summer genom	Alternative assembl	tr|A0A2K3MC08|A0A2K3MC08_TRIPR	29.5	tr|A0A2K3MC08|A0A2K3MC08_TRIPR 60S ribosomal protein l23 OS=Trifolium pratense OX=57577 GN=L195_g044412 PE	trifolium pratense		[illegible]	[illegible]
endless_summer2_g27079	endless_summer2_g27079	Endless Summer genom	Alternative assembl	XP_028067080.1	48	XP_028067080.1 uncharacterized protein LOC114269907 [Camellia sinensis]	camellia sinensis	29760.VIT_18s0001g04430.t01	[illegible]	[illegible]
endless_summer2_g27201	endless_summer2_g27201	Endless Summer genom	Alternative assembl	tr|A0A6V7QX45|A0A6V7QX45_ANACO	35.1	tr|A0A6V7QX45|A0A6V7QX45_ANACO SWIM-type domain-containing protein OS=Ananas comosus var. bracteatus OX=296	ananas comosus var. bracte	39947.LOC_Os08g25960.1	[illegible]	[illegible]
endless_summer2_g27212	endless_summer2_g27212	Endless Summer genom	Alternative assembl	tr|A0A5B7CDP5|A0A5B7CDP5_DAVIN	36.7	tr|A0A5B7CDP5|A0A5B7CDP5_DAVIN Putative retrovirus-related Pol polyprotein OS=Davidia involucrata OX=16924 GN=D	davidia involucrata	39947.LOC_Os09g26970.1	[illegible]	[illegible]
endless_summer2_g27357	endless_summer2_g27357	Endless Summer genom	Alternative assembl	XP_028052963.1	47.4	XP_028052963.1 uncharacterized protein LOC114257408 [Camellia sinensis]	camellia sinensis	39947.LOC_Os09g08590.1	[illegible]	[illegible]
endless_summer2_g2743	endless_summer2_g2743	Endless Summer genom	Alternative assembl	XP_028077335.1	85.1	XP_028077335.1 meiotic recombination protein SPO11-2 isoform X1 [Camellia sinensis]	camellia sinensis	29760.VIT_02s0025g00410.t01	[illegible]	[illegible]
endless_summer2_g27507	endless_summer2_g27507	Endless Summer genom	Alternative assembl	tr|A0A4V6AA29|A0A4V6AA29_POPAL	50.5	tr|A0A4V6AA29|A0A4V6AA29_POPAL CCHC-type domain-containing protein OS=Populus alba OX=43335 GN=D5086_000	populus alba	4081.Solyc04g025620.1.1	[illegible]	[illegible]
endless_summer2_g27560	endless_summer2_g27560	Endless Summer genom	Alternative assembl	tr|A0A7J7FXG4|A0A7J7FXG4_CAMSI	41.4	tr|A0A7J7FXG4|A0A7J7FXG4_CAMSI CCHC-type domain-containing protein OS=Camellia sinensis OX=4442 GN=HYC85	camellia sinensis	29760.VIT_18s0001g09780.t01	[illegible]	[illegible]
endless_summer2_g2759	endless_summer2_g2759	Endless Summer genom	Alternative assembl	XP_012831102.1	44.1	XP_012831102.1 PREDICTED: uncharacterized protein LOC105952133 [Erythranthe guttata]	erythranthe guttata	39947.LOC_Os09g08590.1	[illegible]	[illegible]
endless_summer2_g27654	endless_summer2_g27654	Endless Summer genom	Alternative assembl	tr|A0A438BTN9|A0A438BTN9_VITVI	50.8	tr|A0A438BTN9|A0A438BTN9_VITVI CCHC-type domain-containing protein OS=Vitis vinifera OX=29760 GN=CK203_099(	vitis vinifera	29760.VIT_07s0095g00580.t01	[illegible]	[illegible]
endless_summer2_g27687	endless_summer2_g27687	Endless Summer genom	Alternative assembl	tr|U5U4X7|U5U4X7_SCUBA	47.2	tr|U5U4X7|U5U4X7_SCUBA MYB10 (Fragment) OS=Scutellaria baicalensis OX=65409 GN=myb PE=2 SV=1	scutellaria baicalensis	3847.GLYMA13G19910.1	[illegible]	[illegible]
endless_summer2_g27696	endless_summer2_g27696	Endless Summer genom	Alternative assembl	tr|A0A2P5ATL3|A0A2P5ATL3_TREOI	52.9	tr|A0A2P5ATL3|A0A2P5ATL3_TREOI Zinc finger, GRF-type OS=Trema orientale OX=63057 GN=TorRG33x02_341670 PE	trema orientale	4081.Solyc03g122220.1.1	[illegible]	[illegible]
endless_summer2_g27711	endless_summer2_g27711	Endless Summer genom	Alternative assembl	XP_030959788.1	58.3	XP_030959788.1 sugar transporter ERD6-like 5 isoform X1 [Quercus lobata]	quercus lobata	29760.VIT_14s0030g00230.t01	[illegible]	[illegible]
endless_summer2_g27842	endless_summer2_g27842	Endless Summer genom	Alternative assembl	tr|A0A5B7B4D6|A0A5B7B4D6_DAVIN	42.1	tr|A0A5B7B4D6|A0A5B7B4D6_DAVIN BED-type domain-containing protein OS=Davidia involucrata OX=16924 GN=Din_03	davidia involucrata	3694.POPTR_0007s00640.1	[illegible]	[illegible]
endless_summer2_g28014	endless_summer2_g28014	Endless Summer genom	Alternative assembl	tr|A0A7J7HIJ8|A0A7J7HIJ8_CAMSI	37.1	tr|A0A7J7HIJ8|A0A7J7HIJ8_CAMSI F-box domain-containing protein OS=Camellia sinensis OX=4442 GN=HYC85_010151 F	camellia sinensis	4081.Solyc11g007620.1.1	[illegible]	[illegible]
endless_summer2_g28034	endless_summer2_g28034	Endless Summer genom	Alternative assembl	tr|A0A5J5B2A0|A0A5J5B2A0_9ASTE	55.5	tr|A0A5J5B2A0|A0A5J5B2A0_9ASTE Uncharacterized protein OS=Nyssa sinensis OX=561372 GN=F0562_029168 PE=4 SV=	nyssa sinensis	4113.PGSC0003DMT400094555	[illegible]	[illegible]
endless_summer2_g28157	endless_summer2_g28157	Endless Summer genom	Alternative assembl	tr|A0A5B7BTH8|A0A5B7BTH8_DAVIN	71.3	tr|A0A5B7BTH8|A0A5B7BTH8_DAVIN Uncharacterized protein OS=Davidia involucrata OX=16924 GN=Din_040742 PE=4 S	davidia involucrata	4081.Solyc01g103070.2.1	[illegible]	[illegible]
endless_summer2_g28187	endless_summer2_g28187	Endless Summer genom	Alternative assembl	XP_030941883.1	39.2	XP_030941883.1 replication protein A 70 kDa DNA-binding subunit A-like [Quercus lobata]	quercus lobata	4081.Solyc10g074430.1.1	[illegible]	[illegible]
endless_summer2_g28215	endless_summer2_g28215	Endless Summer genom	Alternative assembl	XP_025877706.1	27	XP_025877706.1 disheveled-associated activator of morphogenesis 1 isoform X2 [Oryza sativa Japonica Group]	oryza sativa japonica grou	39947.LOC_Os12g12690.1	[illegible]	[illegible]
endless_summer2_g28251	endless_summer2_g28251	Endless Summer genom	Alternative assembl	tr|A0A6P6TWU2|A0A6P6TWU2_COFAR	27.2	tr|A0A6P6TWU2|A0A6P6TWU2_COFAR uncharacterized protein LOC113704695 OS=Coffea arabica OX=13443 GN=LOC11	coffea arabica	29760.VIT_05s0049g01920.t01	[illegible]	[illegible]
endless_summer2_g28502	endless_summer2_g28502	Endless Summer genom	Alternative assembl	XP_021990619.1	83	XP_021990619.1 oligopeptide transporter 4 [Helianthus annuus]	helianthus annuus	29760.VIT_03s0097g00510.t01	[illegible]	[illegible]
endless_summer2_g28622	endless_summer2_g28622	Endless Summer genom	Alternative assembl	XP_012837725.1	33.3	XP_012837725.1 PREDICTED: replication protein A 70 kDa DNA-binding subunit B-like [Erythranthe guttata]	erythranthe guttata	4113.PGSC0003DMT400092159	[illegible]	[illegible]
endless_summer2_g28696	endless_summer2_g28696	Endless Summer genom	Alternative assembl	XP_028057130.1	82.1	XP_028057130.1 alpha-L-fucosidase 2-like [Camellia sinensis]	camellia sinensis	3694.POPTR_0009s09690.1	[illegible]	[illegible]
endless_summer2_g29251	endless_summer2_g29251	Endless Summer genom	Alternative assembl	XP_008239864.1	46.9	XP_008239864.1 PREDICTED: uncharacterized protein LOC103338441 [Prunus mume]	prunus mume	3847.GLYMA10G10095.1	[illegible]	[illegible]
endless_summer2_g29309	endless_summer2_g29309	Endless Summer genom	Alternative assembl	XP_034708948.1	34.5	XP_034708948.1 uncharacterized protein LOC117932000 [Vitis riparia]	vitis riparia	4113.PGSC0003DMT400085322	[illegible]	[illegible]
endless_summer2_g29325	endless_summer2_g29325	Endless Summer genom	Alternative assembl	tr|A0A5A7V681|A0A5A7V681_CUCME	32.5	tr|A0A5A7V681|A0A5A7V681_CUCME Retrotrans_gag domain-containing protein OS=Cucumis melo var. makuwa OX=1194	cucumis melo var. makuwa	29760.VIT_05s0049g01920.t01	[illegible]	[illegible]
endless_summer2_g29367	endless_summer2_g29367	Endless Summer genom	Alternative assembl	XP_043633649.1	47	XP_043633649.1 protein FAR1-RELATED SEQUENCE 1-like [Erigeron canadensis]	erigeron canadensis	39947.LOC_Os11g45530.1	[illegible]	[illegible]
endless_summer2_g2938	endless_summer2_g2938	Endless Summer genom	Alternative assembl	tr|A0A5J5AEB1|A0A5J5AEB1_9ASTE	59.1	tr|A0A5J5AEB1|A0A5J5AEB1_9ASTE PCI domain-containing protein OS=Nyssa sinensis OX=561372 GN=F0562_035537 P	nyssa sinensis	29760.VIT_16s0098g01720.t01	Involved in regulation of actin and microtubule organization. Part of a WA	[illegible]
endless_summer2_g29498	endless_summer2_g29498	Endless Summer genom	Alternative assembl	XP_028115849.1	73.8	XP_028115849.1 CRS2-associated factor 2, chloroplastic [Camellia sinensis]	camellia sinensis	29760.VIT_01s0127g00410.t01	[illegible]	[illegible]
endless_summer2_g29537	endless_summer2_g29537	Endless Summer genom	Alternative assembl	tr|A0A5J5AHZ0|A0A5J5AHZ0_9ASTE	38.1	tr|A0A5J5AHZ0|A0A5J5AHZ0_9ASTE Uncharacterized protein OS=Nyssa sinensis OX=561372 GN=F0562_034367 PE=4 SV	nyssa sinensis	4113.PGSC0003DMT400036070	[illegible]	[illegible]
endless_summer2_g29599	endless_summer2_g29599	Endless Summer genom	Alternative assembl	XP_028051140.1	51.4	XP_028051140.1 protein ALP1-like [Camellia sinensis]	camellia sinensis	4081.Solyc09g064290.1.1	[illegible]	[illegible]
endless_summer2_g29628	endless_summer2_g29628	Endless Summer genom	Alternative assembl	XP_020249142.1	53.8	XP_020249142.1 uncharacterized protein LOC109826522 [Asparagus officinalis]	asparagus officinalis	4577.GRMZM2G000628_P01	[illegible]	[illegible]
endless_summer2_g29629	endless_summer2_g29629	Endless Summer genom	Alternative assembl	tr|A0A3S3QHU8|A0A3S3QHU8_9MAGN	44.1	tr|A0A3S3QHU8|A0A3S3QHU8_9MAGN Putative nuclease HARBI1 OS=Cinnamomum micranthum f. kanehirae OX=337451	cinnamomum micranthum f.	3694.POPTR_0008s21790.1	[illegible]	[illegible]
endless_summer2_g29720	endless_summer2_g29720	Endless Summer genom	Alternative assembl	tr|Q153Y5|Q153Y5_SOLME	51.3	tr|Q153Y5|Q153Y5_SOLME Polyprotein (Fragment) OS=Solanum melongena OX=4111 PE=4 SV=1	solanum melongena	39947.LOC_Os11g03830.1	[illegible]	[illegible]
endless_summer2_g2984	endless_summer2_g2984	Endless Summer genom	Alternative assembl	XP_038707252.1	58.1	XP_038707252.1 uncharacterized protein LOC120002560 [Tripterygium wilfordii]	tripterygium wilfordii	4577.GRMZM2G033054_P01	[illegible]	[illegible]
endless_summer2_g29889	endless_summer2_g29889	Endless Summer genom	Alternative assembl	tr|A0A2N9GYT1|A0A2N9GYT1_FAGSY	34.6	tr|A0A2N9GYT1|A0A2N9GYT1_FAGSY SWIM-type domain-containing protein OS=Fagus sylvatica OX=28930 GN=FSB_L	fagus sylvatica	39947.LOC_Os04g33010.1	[illegible]	[illegible]
endless_summer2_g2998	endless_summer2_g2998	Endless Summer genom	Alternative assembl	XP_027081743.1	78.8	XP_027081743.1 endoglucanase 1-like [Coffea arabica]	coffea arabica	29760.VIT_02s0025g02650.t01	[illegible]	[illegible]
endless_summer2_g30041	endless_summer2_g30041	Endless Summer genom	Alternative assembl	XP_028097185.1	43.2	XP_028097185.1 uncharacterized protein LOC114297038 [Camellia sinensis]	camellia sinensis	29760.VIT_05s0020g00850.t01	[illegible]	[illegible]
endless_summer2_g3008	endless_summer2_g3008	Endless Summer genom	Alternative assembl	tr|A0A8C5LN91|A0A8C5LN91_9ANUR	34.6	tr|A0A8C5LN91|A0A8C5LN91_9ANUR Uncharacterized protein OS=Leptobrachium leishanense OX=445787 PE=4 SV=1	leptobrachium leishanense	39946.BGIOSGA028197-PA	[illegible]	[illegible]
endless_summer2_g30137	endless_summer2_g30137	Endless Summer genom	Alternative assembl	tr|A0A7J7FYF3|A0A7J7FYF3_CAMSI	28.9	tr|A0A7J7FYF3|A0A7J7FYF3_CAMSI PMD domain-containing protein OS=Camellia sinensis OX=4442 GN=HYC85_029624	camellia sinensis	4555.Si039121m	[illegible]	[illegible]
endless_summer2_g30156	endless_summer2_g30156	Endless Summer genom	Alternative assembl	XP_028080526.1	79.1	XP_028080526.1 protein DETOXIFICATION 12-like isoform X1 [Camellia sinensis]	camellia sinensis	29760.VIT_01s0011g04450.t01	[illegible]	[illegible]
endless_summer2_g30272	endless_summer2_g30272	Endless Summer genom	Alternative assembl	tr|A0A5B6ZH96|A0A5B6ZH96_DAVIN	53.8	tr|A0A5B6ZH96|A0A5B6ZH96_DAVIN Uncharacterized protein (Fragment) OS=Davidia involucrata OX=16924 GN=Din_0131	davidia involucrata	4081.Solyc10g008220.2.1	[illegible]	[illegible]
endless_summer2_g30295	endless_summer2_g30295	Endless Summer genom	Alternative assembl	tr|A0A2R6PAR1|A0A2R6PAR1_ACTCC	65.9	tr|A0A2R6PAR1|A0A2R6PAR1_ACTCC Late blight resistance protein like OS=Actinidia chinensis var. chinensis OX=159084	actinidia chinensis var. chi	4113.PGSC0003DMT400056264	[illegible]	[illegible]
endless_summer2_g30357	endless_summer2_g30357	Endless Summer genom	Alternative assembl	XP_028078345.1	25.7	XP_028078345.1 uncharacterized protein LOC114280204 [Camellia sinensis]	camellia sinensis	29760.VIT_08s0058g01250.t01	[illegible]	[illegible]
endless_summer2_g30362	endless_summer2_g30362	Endless Summer genom	Alternative assembl	tr|A0A2P5D4I0|A0A2P5D4I0_PARAD	76.7	tr|A0A2P5D4I0|A0A2P5D4I0_PARAD Tetratricopeptide-like helical domain containing protein OS=Parasponia andersonii OX=	parasponia andersonii	3694.POPTR_0007s15300.1	[illegible]	[illegible]
endless_summer2_g30397	endless_summer2_g30397	Endless Summer genom	Alternative assembl	tr|A0A2R6S0F1|A0A2R6S0F1_ACTCC	47.1	tr|A0A2R6S0F1|A0A2R6S0F1_ACTCC RNA-binding protein OS=Actinidia chinensis var. chinensis OX=1590841 GN=CEY0	actinidia chinensis var. chi	4113.PGSC0003DMT400075260	[illegible]	[illegible]
endless_summer2_g30437	endless_summer2_g30437	Endless Summer genom	Alternative assembl	tr|A0A5C7J3L1|A0A5C7J3L1_GOSTO	33.9	tr|A0A5C7J3L1|A0A5C7J3L1_GOSTO Uncharacterized protein OS=Gossypium tomentosum OX=34277 GN=ES332_1Z00090	gossypium tomentosum	4113.PGSC0003DMT400064323	[illegible]	[illegible]
endless_summer2_g30492	endless_summer2_g30492	Endless Summer genom	Alternative assembl	tr|A0A6A1V7J4|A0A6A1V7J4_9ROSI	28.9	tr|A0A6A1V7J4|A0A6A1V7J4_9ROSI Reverse transcriptase domain-containing protein OS=Morella rubra OX=262757 GN=CJ(	morella rubra	4577.GRMZM2G329093_P01	[illegible]	[illegible]
endless_summer2_g30564	endless_summer2_g30564	Endless Summer genom	Alternative assembl	XP_028088374.1	64.6	XP_028088374.1 glutamate receptor 2.8-like isoform X1 [Camellia sinensis]	camellia sinensis	4081.Solyc06g063200.1.1	[illegible]	[illegible]
endless_summer2_g30619	endless_summer2_g30619	Endless Summer genom	Alternative assembl	tr|A0A7J0EX35|A0A7J0EX35_9ERIC	37.4	tr|A0A7J0EX35|A0A7J0EX35_9ERIC Uncharacterized protein OS=Actinidia rufa OX=165716 GN=Acr_07g0011490 PE=4 SV=	actinidia rufa	3694.POPTR_0006s26460.1	[illegible]	[illegible]
endless_summer2_g30622	endless_summer2_g30622	Endless Summer genom	Alternative assembl	XP_034218999.1	36.8	XP_034218999.1 uncharacterized protein LOC117630373 [Prunus dulcis]	prunus dulcis		[illegible]	[illegible]
endless_summer2_g30637	endless_summer2_g30637	Endless Summer genom	Alternative assembl	tr|A0A2N9G373|A0A2N9G373_FAGSY	48.2	tr|A0A2N9G373|A0A2N9G373_FAGSY CCHC-type domain-containing protein OS=Fagus sylvatica OX=28930 GN=FSB_LO(	fagus sylvatica	29760.VIT_07s0095g00580.t01	[illegible]	[illegible]
endless_summer2_g30695	endless_summer2_g30695	Endless Summer genom	Alternative assembl	XP_028066665.1	26.1	XP_028066665.1 uncharacterized protein LOC114269533 [Camellia sinensis]	camellia sinensis	4113.PGSC0003DMT400093611	[illegible]	[illegible]
endless_summer2_g3070	endless_summer2_g3070	Endless Summer genom	Alternative assembl	tr|A0A438DKZ5|A0A438DKZ5_VITVI	46.6	tr|A0A438DKZ5|A0A438DKZ5_VITVI Retrovirus-related Pol polyprotein from transposon opus OS=Vitis vinifera OX=29760 (	vitis vinifera	39947.LOC_Os04g20720.1	[illegible]	[illegible]
endless_summer2_g30836	endless_summer2_g30836	Endless Summer genom	Alternative assembl	XP_030931186.1	49.8	XP_030931186.1 protein FAR1-RELATED SEQUENCE 5-like [Quercus lobata]	quercus lobata	3694.POPTR_0004s21880.1	[illegible]	[illegible]
endless_summer2_g30845	endless_summer2_g30845	Endless Summer genom	Alternative assembl	tr|A0A5B7BU20|A0A5B7BU20_DAVIN	58.9	tr|A0A5B7BU20|A0A5B7BU20_DAVIN Putative Eukaryotic LigT isoform 2 (Fragment) OS=Davidia involucrata OX=16924 G	davidia involucrata	29760.VIT_09s0002g04060.t01	[illegible]	[illegible]
endless_summer2_g30935	endless_summer2_g30935	Endless Summer genom	Alternative assembl	XP_031745312.1	24.9	XP_031745312.1 uncharacterized protein LOC116405439 [Cucumis sativus]	cucumis sativus	4113.PGSC0003DMT400048835	[illegible]	[illegible]
endless_summer2_g3094	endless_summer2_g3094	Endless Summer genom	Alternative assembl	XP_030924558.1	32.7	XP_030924558.1 uncharacterized protein LOC115951515 [Quercus lobata]	quercus lobata		[illegible]	[illegible]
endless_summer2_g30973	endless_summer2_g30973	Endless Summer genom	Alternative assembl	XP_028079638.1	80.8	XP_028079638.1 caffeoylshikimate esterase-like [Camellia sinensis]	camellia sinensis	29760.VIT_09s0002g05730.t01	[illegible]	[illegible]
endless_summer2_g30976	endless_summer2_g30976	Endless Summer genom	Alternative assembl	tr|A0A834GPT9|A0A834GPT9_RHOSS	43.9	tr|A0A834GPT9|A0A834GPT9_RHOSS Retrotran_gag_3 domain-containing protein OS=Rhododendron simsii OX=118357 GN	rhododendron simsii	4113.PGSC0003DMT400096239	[illegible]	[illegible]
endless_summer2_g31034	endless_summer2_g31034	Endless Summer genom	Alternative assembl	tr|A0A5B6ZA07|A0A5B6ZA07_DAVIN	64.2	tr|A0A5B6ZA07|A0A5B6ZA07_DAVIN Uncharacterized protein OS=Davidia involucrata OX=16924 GN=Din_009697 PE=4 S	davidia involucrata	4577.GRMZM2G000628_P01	[illegible]	[illegible]
endless_summer2_g31052	endless_summer2_g31052	Endless Summer genom	Alternative assembl	XP_028099009.1	36	XP_028099009.1 uncharacterized protein LOC114298597 [Camellia sinensis]	camellia sinensis	3694.POPTR_0011s01230.1	[illegible]	[illegible]
endless_summer2_g31166	endless_summer2_g31166	Endless Summer genom	Alternative assembl	tr|A0A5A7TFN2|A0A5A7TFN2_CUCME	27.1	tr|A0A5A7TFN2|A0A5A7TFN2_CUCME Ty3-gypsy retrotransposon protein OS=Cucumis melo var. makuwa OX=1194695 G	cucumis melo var. makuwa	39947.LOC_Os06g25550.1	[illegible]	[illegible]
endless_summer2_g31195	endless_summer2_g31195	Endless Summer genom	Alternative assembl	XP_028099909.1	29	XP_028099909.1 uncharacterized protein LOC114299384 [Camellia sinensis]	camellia sinensis	4538.ORGLA03G0218000.1	[illegible]	[illegible]
endless_summer2_g31316	endless_summer2_g31316	Endless Summer genom	Alternative assembl	XP_009631223.1	84	XP_009631223.1 fumarylacetoacetase [Nicotiana tomentosiformis]	nicotiana tomentosiformis	29760.VIT_00s0187g00340.t01	[illegible]	[illegible]
endless_summer2_g31338	endless_summer2_g31338	Endless Summer genom	Alternative assembl	XP_035836996.1	44.5	XP_035836996.1 uncharacterized protein LOC110877269 [Helianthus annuus]	helianthus annuus	4081.Solyc02g062480.1.1	[illegible]	[illegible]
endless_summer2_g31378	endless_summer2_g31378	Endless Summer genom	Alternative assembl	XP_028796299.1	74.4	XP_028796299.1 LOW QUALITY PROTEIN: stromal cell-derived factor 2-like protein [Prosopis alba]	prosopis alba	4081.Solyc03g117020.2.1	[illegible]	[illegible]
endless_summer2_g31395	endless_summer2_g31395	Endless Summer genom	Alternative assembl	tr|A0A8B8ZND3|A0A8B8ZND3_PHODC	52.4	tr|A0A8B8ZND3|A0A8B8ZND3_PHODC uncharacterized protein LOC120106749 OS=Phoenix dactylifera OX=42345 GN=LO(	phoenix dactylifera	39947.LOC_Os04g16630.1	[illegible]	[illegible]
endless_summer2_g31501	endless_summer2_g31501	Endless Summer genom	Alternative assembl	tr|A0A2N9G2L4|A0A2N9G2L4_FAGSY	53.6	tr|A0A2N9G2L4|A0A2N9G2L4_FAGSY Uncharacterized protein OS=Fagus sylvatica OX=28930 GN=FSB_LOCUS21451 PE=	fagus sylvatica	3694.POPTR_0011s01230.1	[illegible]	[illegible]
endless_summer2_g31543	endless_summer2_g31543	Endless Summer genom	Alternative assembl	XP_022975048.1	47.2	XP_022975048.1 uncharacterized protein LOC111474005 [Cucurbita maxima]	cucurbita maxima	39947.LOC_Os01g35290.1	[illegible]	[illegible]
endless_summer2_g31571	endless_summer2_g31571	Endless Summer genom	Alternative assembl	XP_024020806.1	39.4	XP_024020806.1 uncharacterized protein LOC112091411 [Morus notabilis]	morus notabilis	3694.POPTR_0011s01230.1	[illegible]	[illegible]
endless_summer2_g31577	endless_summer2_g31577	Endless Summer genom	Alternative assembl	tr|A0A834LPB9|A0A834LPB9_RHOSS	61.4	tr|A0A834LPB9|A0A834LPB9_RHOSS Uncharacterized protein OS=Rhododendron simsii OX=118357 GN=RHSIM_Rhsim03	rhododendron simsii	3847.GLYMA12G11564.1	[illegible]	[illegible]
endless_summer2_g31765	endless_summer2_g31765	Endless Summer genom	Alternative assembl	tr|A0A7J0E4H4|A0A7J0E4H4_9ERIC	40	tr|A0A7J0E4H4|A0A7J0E4H4_9ERIC BED-type domain-containing protein OS=Actinidia rufa OX=165716 GN=Acr_01g00115	actinidia rufa	4641.GSMUA_AchrUn_randomP14380_001	[illegible]	[illegible]
endless_summer2_g31859	endless_summer2_g31859	Endless Summer genom	Alternative assembl	tr|A0A6P6BAI5|A0A6P6BAI5_DURZI	36.5	tr|A0A6P6BAI5|A0A6P6BAI5_DURZI uncharacterized protein LOC111316412 OS=Durio zibethinus OX=66656 GN=LOC111	durio zibethinus	3694.POPTR_0016s11650.1	[illegible]	[illegible]
endless_summer2_g32009	endless_summer2_g32009	Endless Summer genom	Alternative assembl	tr|A0A834LLR8|A0A834LLR8_RHOSS	41.6	tr|A0A834LLR8|A0A834LLR8_RHOSS PMD domain-containing protein OS=Rhododendron simsii OX=118357 GN=RHSIM	rhododendron simsii	4113.PGSC0003DMT400039052	[illegible]	[illegible]
endless_summer2_g32010	endless_summer2_g32010	Endless Summer genom	Alternative assembl	tr|A0A5D3D7V5|A0A5D3D7V5_CUCME	51.1	tr|A0A5D3D7V5|A0A5D3D7V5_CUCME Uncharacterized protein OS=Cucumis melo var. makuwa OX=1194695 GN=E5676_	cucumis melo var. makuwa	3694.POPTR_0011s01230.1	[illegible]	[illegible]
endless_summer2_g3202	endless_summer2_g3202	Endless Summer genom	Alternative assembl	XP_028055552.1	59.6	XP_028055552.1 uncharacterized protein LOC114259712 [Camellia sinensis]	camellia sinensis	4081.Solyc03g044840.1.1	[illegible]	[illegible]
endless_summer2_g32241	endless_summer2_g32241	Endless Summer genom	Alternative assembl	tr|A0A6J5VK59|A0A6J5VK59_PRUAR	64.2	tr|A0A6J5VK59|A0A6J5VK59_PRUAR RNase H type-1 domain-containing protein OS=Prunus armeniaca OX=36596 GN=CUI	prunus armeniaca	3694.POPTR_0011s01230.1	[illegible]	[illegible]
endless_summer2_g32272	endless_summer2_g32272	Endless Summer genom	Alternative assembl	tr|A0A5J4ZVQ0|A0A5J4ZVQ0_9ASTE	78.1	tr|A0A5J4ZVQ0|A0A5J4ZVQ0_9ASTE Calcium-transporting ATPase OS=Nyssa sinensis OX=561372 GN=F0562_009101 PE	nyssa sinensis	3694.POPTR_0006s07240.1	[illegible]	[illegible]
endless_summer2_g32293	endless_summer2_g32293	Endless Summer genom	Alternative assembl	XP_022891645.1	44.2	XP_022891645.1 NAC transcription factor 25-like [Olea europaea var. sylvestris]	olea europaea var. sylvestri	4513.MLOC_65286.1	[illegible]	[illegible]
endless_summer2_g3232	endless_summer2_g3232	Endless Summer genom	Alternative assembl	XP_028099224.1	39.1	XP_028099224.1 uncharacterized protein LOC114298799 [Camellia sinensis]	camellia sinensis	3694.POPTR_0001s00740.1	[illegible]	[illegible]
endless_summer2_g32380	endless_summer2_g32380	Endless Summer genom	Alternative assembl	tr|A0A7D9HWX2|A0A7D9HWX2_PARCT	30.5	tr|A0A7D9HWX2|A0A7D9HWX2_PARCT Effector candidate, partial OS=Paramuricea clavata OX=317549 GN=PACLA_6A056(	paramuricea clavata	39947.LOC_Os07g05280.1	[illegible]	[illegible]
endless_summer2_g32428	endless_summer2_g32428	Endless Summer genom	Alternative assembl	tr|A0A5B6Z245|A0A5B6Z245_DAVIN	59.5	tr|A0A5B6Z245|A0A5B6Z245_DAVIN Protein kinase domain-containing protein (Fragment) OS=Davidia involucrata OX=1692	davidia involucrata	29760.VIT_11s0052g01420.t01	[illegible]	[illegible]
endless_summer2_g32528	endless_summer2_g32528	Endless Summer genom	Alternative assembl	tr|A0A558HCW9|A0A558HCW9_9GAMM	33.3	tr|A0A558HCW9|A0A558HCW9_9GAMM Reverse transcriptase-like protein (Fragment) OS=Cobetia crustatorum OX=553385	cobetia crustatorum	4533.OB06G16940.1	[illegible]	[illegible]
endless_summer2_g32573	endless_summer2_g32573	Endless Summer genom	Alternative assembl	XP_028108292.1	74.2	XP_028108292.1 UDP-glucuronate 4-epimerase 1 [Camellia sinensis]	camellia sinensis	3847.GLYMA17G07740.1	[illegible]	[illegible]
endless_summer2_g32580	endless_summer2_g32580	Endless Summer genom	Alternative assembl	tr|A0A5N5J6G7|A0A5N5J6G7_9ROSI	51.4	tr|A0A5N5J6G7|A0A5N5J6G7_9ROSI Uncharacterized protein OS=Salix brachista OX=2182728 GN=DKX38_028687 PE=4 S	salix brachista	39947.LOC_Os02g01840.1	[illegible]	[illegible]
endless_summer2_g32647	endless_summer2_g32647	Endless Summer genom	Alternative assembl	XP_028123309.1	53.6	XP_028123309.1 uncharacterized protein LOC114320353 [Camellia sinensis]	camellia sinensis	39946.BGIOSGA030975-PA	[illegible]	[illegible]
endless_summer2_g32656	endless_summer2_g32656	Endless Summer genom	Alternative assembl	XP_019238934.1	41.7	XP_019238934.1 PREDICTED: uncharacterized protein LOC109218987 [Nicotiana attenuata]	nicotiana attenuata	4113.PGSC0003DMT400091433	[illegible]	[illegible]
endless_summer2_g32668	endless_summer2_g32668	Endless Summer genom	Alternative assembl	tr|W9RWP1|W9RWP1_9ROSA	53	tr|W9RWP1|W9RWP1_9ROSA RT_RNaseH domain-containing protein OS=Morus notabilis OX=981085 GN=L484_009453	morus notabilis	3694.POPTR_0011s01230.1	[illegible]	[illegible]
endless_summer2_g32762	endless_summer2_g32762	Endless Summer genom	Alternative assembl	tr|A0A835DEK8|A0A835DEK8_9MAGN	52.2	tr|A0A835DEK8|A0A835DEK8_9MAGN Uncharacterized protein OS=Tetracentron sinense OX=13715 GN=HHK36_016312 PE	tetracentron sinense	4558.Sb03g000205.1	[illegible]	[illegible]
endless_summer2_g32795	endless_summer2_g32795	Endless Summer genom	Alternative assembl	tr|A0A7J0F7V1|A0A7J0F7V1_9ERIC	41.1	tr|A0A7J0F7V1|A0A7J0F7V1_9ERIC Integral membrane Yip1 family protein OS=Actinidia rufa OX=165716 GN=Acr_10g0001	actinidia rufa	29760.VIT_01s0026g02570.t01	[illegible]	[illegible]
endless_summer2_g32864	endless_summer2_g32864	Endless Summer genom	Alternative assembl	tr|A0A834H198|A0A834H198_RHOSS	52.3	tr|A0A834H198|A0A834H198_RHOSS Retrotran_gag_3 domain-containing protein OS=Rhododendron simsii OX=118357 GN	rhododendron simsii	39946.BGIOSGA002386-PA	[illegible]	[illegible]
endless_summer2_g32875	endless_summer2_g32875	Endless Summer genom	Alternative assembl	tr|A0A2N9G9M4|A0A2N9G9M4_FAGSY	31.8	tr|A0A2N9G9M4|A0A2N9G9M4_FAGSY RNase H type-1 domain-containing protein OS=Fagus sylvatica OX=28930 GN=FSE	fagus sylvatica	39947.LOC_Os01g06700.1	[illegible]	[illegible]
endless_summer2_g32883	endless_summer2_g32883	Endless Summer genom	Alternative assembl	tr|A0A6A4L046|A0A6A4L046_9ERIC	35.7	tr|A0A6A4L046|A0A6A4L046_9ERIC CCHC-type domain-containing protein (Fragment) OS=Rhododendron williamsianum O	rhododendron williamsiani	3694.POPTR_0005s01170.1	[illegible]	[illegible]
endless_summer2_g32895	endless_summer2_g32895	Endless Summer genom	Alternative assembl	tr|A0A5A7SSB3|A0A5A7SSB3_CUCME	61.1	tr|A0A5A7SSB3|A0A5A7SSB3_CUCME Pol protein OS=Cucumis melo var. makuwa OX=1194695 GN=E6C27_scaffold2830	cucumis melo var. makuwa	39947.LOC_Os04g07660.1	[illegible]	[illegible]
endless_summer2_g33009	endless_summer2_g33009	Endless Summer genom	Alternative assembl	tr|A0A5E4GL26|A0A5E4GL26_PRUDU	47.9	tr|A0A5E4GL26|A0A5E4GL26_PRUDU PREDICTED: transposon (Fragment) OS=Prunus dulcis OX=3755 GN=ALMOND_2I	prunus dulcis	39947.LOC_Os04g27730.1	[illegible]	[illegible]
endless_summer2_g331	endless_summer2_g331	Endless Summer genom	Alternative assembl	XP_028100424.1	37.5	XP_028100424.1 uncharacterized protein LOC114299795 [Camellia sinensis]	camellia sinensis	39947.LOC_Os02g24860.1	[illegible]	[illegible]
endless_summer2_g3326	endless_summer2_g3326	Endless Summer genom	Alternative assembl	XP_028106070.1	66.4	XP_028106070.1 uncharacterized protein LOC114305158 [Camellia sinensis]	camellia sinensis	4577.GRMZM2G004107_P01	[illegible]	[illegible]
endless_summer2_g33459	endless_summer2_g33459	Endless Summer genom	Alternative assembl	tr|A0A834ZQU4|A0A834ZQU4_9MAGN	83.6	tr|A0A834ZQU4|A0A834ZQU4_9MAGN Uncharacterized protein OS=Tetracentron sinense OX=13715 GN=HHK36_004558 PE	tetracentron sinense	29760.VIT_07s0130g00270.t01	[illegible]	[illegible]
endless_summer2_g3349	endless_summer2_g3349	Endless Summer genom	Alternative assembl	tr|A0A2R6RH71|A0A2R6RH71_ACTCC	41.4	tr|A0A2R6RH71|A0A2R6RH71_ACTCC NAC domain-containing protein OS=Actinidia chinensis var. chinensis OX=1590841	actinidia chinensis var. chi	3694.POPTR_0010s23650.1	[illegible]	[illegible]
endless_summer2_g33547	endless_summer2_g33547	Endless Summer genom	Alternative assembl	tr|A0A4S4EJF9|A0A4S4EJF9_CAMSI	77.2	tr|A0A4S4EJF9|A0A4S4EJF9_CAMSI Germin-like protein OS=Camellia sinensis var. sinensis OX=542762 GN=TEA_011417	camellia sinensis var. sinen	4081.Solyc01g102910.2.1	[illegible]	[illegible]
endless_summer2_g33554	endless_summer2_g33554	Endless Summer genom	Alternative assembl	XP_016753574.1	78.2	XP_016753574.1 germin-like protein subfamily 1 member 14 isoform X2 [Gossypium hirsutum]	gossypium hirsutum	29760.VIT_14s0060g02690.t01	[illegible]	[illegible]
endless_summer2_g33646	endless_summer2_g33646	Endless Summer genom	Alternative assembl	tr|A0A7J0D7F1|A0A7J0D7F1_9ERIC	55.8	tr|A0A7J0D7F1|A0A7J0D7F1_9ERIC Uncharacterized protein OS=Actinidia rufa OX=165716 GN=Acr_00g0000840 PE=4 SV=	actinidia rufa	39947.LOC_Os04g01400.1	[illegible]	[illegible]
endless_summer2_g33712	endless_summer2_g33712	Endless Summer genom	Alternative assembl	XP_011001746.1	26.4	XP_011001746.1 PREDICTED: uncharacterized protein LOC105108935 [Populus euphratica]	populus euphratica		[illegible]	[illegible]
endless_summer2_g33767	endless_summer2_g33767	Endless Summer genom	Alternative assembl	tr|A0A5J5BRZ8|A0A5J5BRZ8_9ASTE	48	tr|A0A5J5BRZ8|A0A5J5BRZ8_9ASTE BED-type domain-containing protein OS=Nyssa sinensis OX=561372 GN=F0562_019	nyssa sinensis	4641.GSMUA_AchrUn_randomP14380_001	[illegible]	[illegible]
endless_summer2_g33826	endless_summer2_g33826	Endless Summer genom	Alternative assembl	XP_035550278.1	73.8	XP_035550278.1 fatty acid amide hydrolase-like isoform X1 [Juglans regia]	juglans regia	29760.VIT_07s0031g02700.t01	[illegible]	[illegible]
endless_summer2_g33880	endless_summer2_g33880	Endless Summer genom	Alternative assembl	tr|A0A7J0GPP8|A0A7J0GPP8_9ERIC	32	tr|A0A7J0GPP8|A0A7J0GPP8_9ERIC Uncharacterized protein OS=Actinidia rufa OX=165716 GN=Acr_23g0011480 PE=4 SV=	actinidia rufa		[illegible]	[illegible]
endless_summer2_g33983	endless_summer2_g33983	Endless Summer genom	Alternative assembl	tr|A0A835LTC8|A0A835LTC8_9MAGN	25.4	tr|A0A835LTC8|A0A835LTC8_9MAGN Uncharacterized protein OS=Coptis chinensis OX=261450 GN=IFM89_031449 PE=4	coptis chinensis		[illegible]	[illegible]
endless_summer2_g34018	endless_summer2_g34018	Endless Summer genom	Alternative assembl	XP_016694589.1	64.3	XP_016694589.1 probable indole-3-pyruvate monooxygenase YUCCA10 [Gossypium hirsutum]	gossypium hirsutum	3847.GLYMA06G15810.1	[illegible]	[illegible]
endless_summer2_g34032	endless_summer2_g34032	Endless Summer genom	Alternative assembl	XP_041003572.1	62.8	XP_041003572.1 probable pectinesterase/pectinesterase inhibitor 51 [Juglans microcarpa x Juglans regia]	juglans microcarpa x juglar	29760.VIT_04s0044g01030.t01	[illegible]	[illegible]
endless_summer2_g3416	endless_summer2_g3416	Endless Summer genom	Alternative assembl	tr|A0A7J9J191|A0A7J9J191_9ROSI	28.4	tr|A0A7J9J191|A0A7J9J191_9ROSI zf-RVT domain-containing protein OS=Gossypium armourianum OX=34283 GN=Goarm_(	gossypium armourianum	3694.POPTR_0027s00270.1	[illegible]	[illegible]
endless_summer2_g34316	endless_summer2_g34316	Endless Summer genom	Alternative assembl	XP_008374315.2	71.4	XP_008374315.2 regulation of nuclear pre-mRNA domain-containing protein 1A isoform X1 [Malus domestica]	malus domestica	29760.VIT_07s0031g01240.t01	[illegible]	[illegible]
endless_summer2_g3436	endless_summer2_g3436	Endless Summer genom	Alternative assembl	tr|Q6GX04|Q6GX04_9METZ	78.7	tr|Q6GX04|Q6GX04_9METZ Cytochrome c oxidase subunit 1 (Fragment) OS=Mycale flagellifera OX=237144 GN=cox1 PE=3	mycale flagellifera	39947.LOC_Os12g33946.1	Cytochrome c oxidase is the component of the respiratory chain that cataly	[illegible]
endless_summer2_g34378	endless_summer2_g34378	Endless Summer genom	Alternative assembl	XP_021802437.1	46	XP_021802437.1 uncharacterized protein LOC110746516 [Prunus avium]	prunus avium	4113.PGSC0003DMT400001036	[illegible]	[illegible]
endless_summer2_g34513	endless_summer2_g34513	Endless Summer genom	Alternative assembl	XP_023910579.1	34.1	XP_023910579.1 uncharacterized protein LOC112022237 [Quercus suber]	quercus suber	39947.LOC_Os09g01370.1	[illegible]	[illegible]
endless_summer2_g3455	endless_summer2_g3455	Endless Summer genom	Alternative assembl	tr|A0A453C8N1|A0A453C8N1_AEGTS	50.6	tr|A0A453C8N1|A0A453C8N1_AEGTS DUF223 domain-containing protein OS=Aegilops tauschii subsp. strangulata OX=200	aegilops tauschii subsp. str	4513.MLOC_52915.3	[illegible]	[illegible]
endless_summer2_g346	endless_summer2_g346	Endless Summer genom	Alternative assembl	XP_028084578.1	42.7	XP_028084578.1 uncharacterized protein LOC114285698 [Camellia sinensis]	camellia sinensis	4081.Solyc06g065250.1.1	[illegible]	[illegible]
endless_summer2_g34844	endless_summer2_g34844	Endless Summer genom	Alternative assembl	tr|A0A8B9AXT3|A0A8B9AXT3_PHODC	34.1	tr|A0A8B9AXT3|A0A8B9AXT3_PHODC uncharacterized protein LOC120112826 OS=Phoenix dactylifera OX=42345 GN=LO	phoenix dactylifera	39947.LOC_Os10g11250.1	[illegible]	[illegible]
endless_summer2_g34847	endless_summer2_g34847	Endless Summer genom	Alternative assembl	tr|A0A7J7GLE6|A0A7J7GLE6_CAMSI	31	tr|A0A7J7GLE6|A0A7J7GLE6_CAMSI GRF-type domain-containing protein OS=Camellia sinensis OX=4442 GN=HYC85_01	camellia sinensis		[illegible]	[illegible]
endless_summer2_g34884	endless_summer2_g34884	Endless Summer genom	Alternative assembl	XP_028101604.1	32.4	XP_028101604.1 uncharacterized protein LOC114300922 [Camellia sinensis]	camellia sinensis	3711.Bra023240.1-P	[illegible]	[illegible]
endless_summer2_g34926	endless_summer2_g34926	Endless Summer genom	Alternative assembl	tr|A0A2N9EJL9|A0A2N9EJL9_FAGSY	42.7	tr|A0A2N9EJL9|A0A2N9EJL9_FAGSY Retrotrans_gag domain-containing protein OS=Fagus sylvatica OX=28930 GN=FSB_L	fagus sylvatica	29760.VIT_00s0823g00010.t01	[illegible]	[illegible]
endless_summer2_g34956	endless_summer2_g34956	Endless Summer genom	Alternative assembl	XP_004233473.1	51.4	XP_004233473.1 uncharacterized protein LOC101251647 [Solanum lycopersicum]	solanum lycopersicum	29760.VIT_00s0211g00130.t01	[illegible]	[illegible]
endless_summer2_g3496	endless_summer2_g3496	Endless Summer genom	Alternative assembl	XP_028123307.1	37.6	XP_028123307.1 uncharacterized protein LOC114320351 [Camellia sinensis]	camellia sinensis		[illegible]	[illegible]
endless_summer2_g35013	endless_summer2_g35013	Endless Summer genom	Alternative assembl	XP_021833443.1	23.4	XP_021833443.1 uncharacterized protein LOC110773249 [Prunus avium]	prunus avium	3694.POPTR_0006s06200.1	[illegible]	[illegible]
endless_summer2_g35128	endless_summer2_g35128	Endless Summer genom	Alternative assembl	XP_028101613.1	44.3	XP_028101613.1 uncharacterized protein LOC114300935 [Camellia sinensis]	camellia sinensis	4113.PGSC0003DMT400054570	[illegible]	[illegible]
endless_summer2_g3514	endless_summer2_g3514	Endless Summer genom	Alternative assembl	XP_028072349.1	33.8	XP_028072349.1 uncharacterized protein LOC114274585 [Camellia sinensis]	camellia sinensis	29760.VIT_14s0060g00370.t01	[illegible]	[illegible]
endless_summer2_g3515	endless_summer2_g3515	Endless Summer genom	Alternative assembl	tr|A0A7J7FYG0|A0A7J7FYG0_CAMSI	24	tr|A0A7J7FYG0|A0A7J7FYG0_CAMSI ULP_PROTEASE domain-containing protein OS=Camellia sinensis OX=4442 GN=H	camellia sinensis		[illegible]	[illegible]
endless_summer2_g3517	endless_summer2_g3517	Endless Summer genom	Alternative assembl	XP_028072773.1	50.9	XP_028072773.1 uncharacterized protein LOC114274997 [Camellia sinensis]	camellia sinensis	4577.GRMZM2G167360_P01	[illegible]	[illegible]
endless_summer2_g35231	endless_summer2_g35231	Endless Summer genom	Alternative assembl	tr|A0A5A7VDP3|A0A5A7VDP3_CUCME	61.9	tr|A0A5A7VDP3|A0A5A7VDP3_CUCME Pol protein OS=Cucumis melo var. makuwa OX=1194695 GN=E6C27_scaffold1536	cucumis melo var. makuwa	4081.Solyc11g050910.1.1	[illegible]	[illegible]
endless_summer2_g35336	endless_summer2_g35336	Endless Summer genom	Alternative assembl	tr|A0A438IJG7|A0A438IJG7_VITVI	49.5	tr|A0A438IJG7|A0A438IJG7_VITVI Uncharacterized protein OS=Vitis vinifera OX=29760 GN=CK203_026128 PE=4 SV=1	vitis vinifera	4113.PGSC0003DMT400094379	[illegible]	[illegible]
endless_summer2_g35373	endless_summer2_g35373	Endless Summer genom	Alternative assembl	XP_028103685.1	75.4	XP_028103685.1 metal transporter Nramp3-like [Camellia sinensis]	camellia sinensis	29760.VIT_07s0129g00620.t01	[illegible]	[illegible]
endless_summer2_g35447	endless_summer2_g35447	Endless Summer genom	Alternative assembl	XP_028055136.1	37.9	XP_028055136.1 uncharacterized protein LOC114259321 [Camellia sinensis]	camellia sinensis		[illegible]	[illegible]
endless_summer2_g35524	endless_summer2_g35524	Endless Summer genom	Alternative assembl	tr|A0A834FVT0|A0A834FVT0_RHOSS	37.3	tr|A0A834FVT0|A0A834FVT0_RHOSS Uncharacterized protein OS=Rhododendron simsii OX=118357 GN=RHSIM_RhsimU	rhododendron simsii	3847.GLYMA09G20150.1	[illegible]	[illegible]
endless_summer2_g35558	endless_summer2_g35558	Endless Summer genom	Alternative assembl	XP_028098700.1	74.8	XP_028098700.1 thioredoxin-like protein HCF164, chloroplastic isoform X1 [Camellia sinensis]	camellia sinensis	29760.VIT_00s0207g00270.t01	[illegible]	[illegible]
endless_summer2_g35595	endless_summer2_g35595	Endless Summer genom	Alternative assembl	tr|A0A5R9QKF8|A0A5R9QKF8_9BACT	32.9	tr|A0A5R9QKF8|A0A5R9QKF8_9BACT Transposase family protein (Fragment) OS=Labilibacter sediminis OX=2570926 GN=	labilibacter sediminis	39947.LOC_Os03g26290.1	[illegible]	[illegible]
endless_summer2_g3561	endless_summer2_g3561	Endless Summer genom	Alternative assembl	tr|A0A6A1VP82|A0A6A1VP82_9ROSI	24.5	tr|A0A6A1VP82|A0A6A1VP82_9ROSI Uncharacterized protein OS=Morella rubra OX=262757 GN=CJ030_MR5G003412 PE=	morella rubra		[illegible]	[illegible]
endless_summer2_g35624	endless_summer2_g35624	Endless Summer genom	Alternative assembl	tr|A0A2S8NQ69|A0A2S8NQ69_9MOLU	21.1	tr|A0A2S8NQ69|A0A2S8NQ69_9MOLU Reverse transcriptase domain-containing protein OS=Spiroplasma sp. ChiS OX=2099(	spiroplasma sp. chis	3694.POPTR_0001s00740.1	[illegible]	[illegible]
endless_summer2_g35643	endless_summer2_g35643	Endless Summer genom	Alternative assembl	tr|A0A7J6VL52|A0A7J6VL52_THATH	37.3	tr|A0A7J6VL52|A0A7J6VL52_THATH Uncharacterized protein OS=Thalictrum thalictroides OX=46969 GN=FRX31_024770 I	thalictrum thalictroides		[illegible]	[illegible]
endless_summer2_g35725	endless_summer2_g35725	Endless Summer genom	Alternative assembl	tr|A0A834L3I7|A0A834L3I7_RHOSS	55.3	tr|A0A834L3I7|A0A834L3I7_RHOSS Uncharacterized protein OS=Rhododendron simsii OX=118357 GN=RHSIM_RhsimUnG	rhododendron simsii	39947.LOC_Os08g19870.1	[illegible]	[illegible]
endless_summer2_g35791	endless_summer2_g35791	Endless Summer genom	Alternative assembl	tr|A0A834HBD1|A0A834HBD1_RHOSS	45.3	tr|A0A834HBD1|A0A834HBD1_RHOSS SWIM-type domain-containing protein OS=Rhododendron simsii OX=118357 GN=RI	rhododendron simsii	29760.VIT_07s0141g00810.t01	[illegible]	[illegible]
endless_summer2_g35899	endless_summer2_g35899	Endless Summer genom	Alternative assembl	tr|A0A3S3R1F6|A0A3S3R1F6_9MAGN	25.7	tr|A0A3S3R1F6|A0A3S3R1F6_9MAGN Uncharacterized protein OS=Cinnamomum micranthum f. kanehirae OX=337451 GN=	cinnamomum micranthum f.	3694.POPTR_0001s00740.1	[illegible]	[illegible]
endless_summer2_g36147	endless_summer2_g36147	Endless Summer genom	Alternative assembl	XP_021601172.1	75.5	XP_021601172.1 2-methylpropanoate--CoA ligase CCL4 [Manihot esculenta]	manihot esculenta	3694.POPTR_0019s09570.1	[illegible]	[illegible]
endless_summer2_g36165	endless_summer2_g36165	Endless Summer genom	Alternative assembl	tr|A0A2N9GYS2|A0A2N9GYS2_FAGSY	28.7	tr|A0A2N9GYS2|A0A2N9GYS2_FAGSY Uncharacterized protein OS=Fagus sylvatica OX=28930 GN=FSB_LOCUS32336 PE	fagus sylvatica	3711.Bra033839.1-P	[illegible]	[illegible]
endless_summer2_g3634	endless_summer2_g3634	Endless Summer genom	Alternative assembl	tr|A0A498JT33|A0A498JT33_MALDO	62.7	tr|A0A498JT33|A0A498JT33_MALDO Peroxidase OS=Malus domestica OX=3750 GN=DVH24_035741 PE=3 SV=1	malus domestica	29760.VIT_02s0012g00450.t01	[illegible]	[illegible]
endless_summer2_g36370	endless_summer2_g36370	Endless Summer genom	Alternative assembl	tr|A0A834G5X3|A0A834G5X3_RHOSS	47.3	tr|A0A834G5X3|A0A834G5X3_RHOSS Retrotran_gag_3 domain-containing protein OS=Rhododendron simsii OX=118357 GN	rhododendron simsii	4113.PGSC0003DMT400096239	[illegible]	[illegible]
endless_summer2_g36487	endless_summer2_g36487	Endless Summer genom	Alternative assembl	XP_028109274.1	81.2	XP_028109274.1 probable endo-1,3(4)-beta-glucanase ARB_01444 [Camellia sinensis]	camellia sinensis	3694.POPTR_0004s10500.1	[illegible]	[illegible]
endless_summer2_g36542	endless_summer2_g36542	Endless Summer genom	Alternative assembl	XP_028108992.1	49	XP_028108992.1 E3 ubiquitin-protein ligase Praja-2-like [Camellia sinensis]	camellia sinensis	4113.PGSC0003DMT400068309	[illegible]	[illegible]
endless_summer2_g36562	endless_summer2_g36562	Endless Summer genom	Alternative assembl	XP_038678541.1	32.4	XP_038678541.1 F-box/kelch-repeat protein At3g06240-like isoform X2 [Tripterygium wilfordii]	tripterygium wilfordii	4081.Solyc07g044920.1.1	[illegible]	[illegible]
endless_summer2_g3663	endless_summer2_g3663	Endless Summer genom	Alternative assembl	tr|A0A6P5FAG3|A0A6P5FAG3_ANACO	45.3	tr|A0A6P5FAG3|A0A6P5FAG3_ANACO protease Do-like 9 OS=Ananas comosus OX=4615 GN=LOC109711695 PE=3 SV=1	ananas comosus	3694.POPTR_0012s07930.1	[illegible]	[illegible]
endless_summer2_g36662	endless_summer2_g36662	Endless Summer genom	Alternative assembl	XP_028059543.1	63.5	XP_028059543.1 protein RIK isoform X1 [Camellia sinensis]	camellia sinensis	29760.VIT_14s0068g00240.t01	[illegible]	[illegible]
endless_summer2_g36728	endless_summer2_g36728	Endless Summer genom	Alternative assembl	XP_034706273.1	67.4	XP_034706273.1 pentatricopeptide repeat-containing protein At5g15340, mitochondrial [Vitis riparia]	vitis riparia	4081.Solyc06g060050.1.1	[illegible]	[illegible]
endless_summer2_g36778	endless_summer2_g36778	Endless Summer genom	Alternative assembl	XP_028102319.1	60.9	XP_028102319.1 uncharacterized protein LOC114301573 [Camellia sinensis]	camellia sinensis	39947.LOC_Os01g06700.1	[illegible]	[illegible]
endless_summer2_g36815	endless_summer2_g36815	Endless Summer genom	Alternative assembl	tr|A0A6A4L9C5|A0A6A4L9C5_9ERIC	37.1	tr|A0A6A4L9C5|A0A6A4L9C5_9ERIC Uncharacterized protein (Fragment) OS=Rhododendron williamsianum OX=262921 GN	rhododendron williamsianum		[illegible]	[illegible]
endless_summer2_g36828	endless_summer2_g36828	Endless Summer genom	Alternative assembl	tr|A0A5H2Y4J7|A0A5H2Y4J7_PRUDU	42.9	tr|A0A5H2Y4J7|A0A5H2Y4J7_PRUDU Reverse transcriptase domain-containing protein OS=Prunus dulcis OX=3755 GN=Prui	prunus dulcis	3694.POPTR_0014s01050.1	[illegible]	[illegible]
endless_summer2_g36829	endless_summer2_g36829	Endless Summer genom	Alternative assembl	tr|A0A5H2XGR0|A0A5H2XGR0_PRUDU	75	tr|A0A5H2XGR0|A0A5H2XGR0_PRUDU Uncharacterized protein OS=Prunus dulcis OX=3755 GN=Prudu_130S000100 PE=4	prunus dulcis	3694.POPTR_0107s00200.1	[illegible]	[illegible]
endless_summer2_g36845	endless_summer2_g36845	Endless Summer genom	Alternative assembl	tr|A0A7J0H9V4|A0A7J0H9V4_9ERIC	33.3	tr|A0A7J0H9V4|A0A7J0H9V4_9ERIC CCHC-type domain-containing protein OS=Actinidia rufa OX=165716 GN=Acr_28g000	actinidia rufa	29760.VIT_01s0026g02570.t01	[illegible]	[illegible]
endless_summer2_g36867	endless_summer2_g36867	Endless Summer genom	Alternative assembl	tr|A0A2P2MM38|A0A2P2MM38_RHIMU	73.4	tr|A0A2P2MM38|A0A2P2MM38_RHIMU Mediator of RNA polymerase II transcription subunit 13 OS=Rhizophora mucronata	rhizophora mucronata	29760.VIT_06s0004g00450.t01	[illegible]	[illegible]
endless_summer2_g36882	endless_summer2_g36882	Endless Summer genom	Alternative assembl	tr|A0A834L2K2|A0A834L2K2_RHOSS	36.7	tr|A0A834L2K2|A0A834L2K2_RHOSS Retrotrans_gag domain-containing protein OS=Rhododendron simsii OX=118357 GN=	rhododendron simsii	4577.GRMZM2G384943_P01	[illegible]	[illegible]
endless_summer2_g37259	endless_summer2_g37259	Endless Summer genom	Alternative assembl	XP_028086402.1	28.4	XP_028086402.1 zinc finger BED domain-containing protein RICESLEEPER 2-like [Camellia sinensis]	camellia sinensis	3694.POPTR_0001s22360.1	[illegible]	[illegible]
endless_summer2_g37396	endless_summer2_g37396	Endless Summer genom	Alternative assembl	tr|A0A5J4ZZ96|A0A5J4ZZ96_9ASTE	84.6	tr|A0A5J4ZZ96|A0A5J4ZZ96_9ASTE PUA domain-containing protein (Fragment) OS=Nyssa sinensis OX=561372 GN=F0562	nyssa sinensis	29760.VIT_19s0085g00840.t01	[illegible]	[illegible]
endless_summer2_g37454	endless_summer2_g37454	Endless Summer genom	Alternative assembl	tr|A0A834HIX8|A0A834HIX8_RHOSS	30.7	tr|A0A834HIX8|A0A834HIX8_RHOSS Uncharacterized protein OS=Rhododendron simsii OX=118357 GN=RHSIM_Rhsim01(	rhododendron simsii		[illegible]	[illegible]
endless_summer2_g37635	endless_summer2_g37635	Endless Summer genom	Alternative assembl	tr|A0A834LWQ4|A0A834LWQ4_RHOSS	32	tr|A0A834LWQ4|A0A834LWQ4_RHOSS SWIM-type domain-containing protein OS=Rhododendron simsii OX=118357 GN=	rhododendron simsii	39947.LOC_Os03g41570.1	[illegible]	[illegible]
endless_summer2_g37660	endless_summer2_g37660	Endless Summer genom	Alternative assembl	tr|Q5JJS4|Q5JJS4_ORYSJ	67	tr|Q5JJS4|Q5JJS4_ORYSJ Ribosomal protein-like OS=Oryza sativa subsp. japonica OX=39947 GN=OSJNBa0026J14.36 PE=4	oryza sativa subsp. japonic	4577.GRMZM2G000628_P01	[illegible]	[illegible]
endless_summer2_g37661	endless_summer2_g37661	Endless Summer genom	Alternative assembl	tr|H6QQU6|H6QQU6_PUCGT	56.2	tr|H6QQU6|H6QQU6_PUCGT Uncharacterized protein OS=Puccinia graminis f. sp. tritici (strain CRL 75-36-700-3 / race SCCL	puccinia graminis f. sp. tri	3694.POPTR_0008s21790.1	[illegible]	[illegible]
endless_summer2_g37707	endless_summer2_g37707	Endless Summer genom	Alternative assembl	XP_021826606.1	66.6	XP_021826606.1 cytochrome P450 71D9-like [Prunus avium]	prunus avium	29760.VIT_19s0015g00110.t01	[illegible]	[illegible]
endless_summer2_g37771	endless_summer2_g37771	Endless Summer genom	Alternative assembl	tr|A0A5B7AVZ6|A0A5B7AVZ6_DAVIN	58.4	tr|A0A5B7AVZ6|A0A5B7AVZ6_DAVIN Uncharacterized protein OS=Davidia involucrata OX=16924 GN=Din_028714 PE=4 S	davidia involucrata	4081.Solyc12g014540.1.1	[illegible]	[illegible]
endless_summer2_g38152	endless_summer2_g38152	Endless Summer genom	Alternative assembl	tr|A0A2U1N5J9|A0A2U1N5J9_ARTAN	36.3	tr|A0A2U1N5J9|A0A2U1N5J9_ARTAN RNase H type-1 domain-containing protein OS=Artemisia annua OX=35608 GN=CTI	artemisia annua	3694.POPTR_0016s11650.1	[illegible]	[illegible]
endless_summer2_g38485	endless_summer2_g38485	Endless Summer genom	Alternative assembl	XP_028069010.1	44.1	XP_028069010.1 uncharacterized protein LOC114271596 [Camellia sinensis]	camellia sinensis	3694.POPTR_0025s00580.1	[illegible]	[illegible]
endless_summer2_g38591	endless_summer2_g38591	Endless Summer genom	Alternative assembl	XP_034701579.1	58.5	XP_034701579.1 putative E3 ubiquitin-protein ligase RING1a [Vitis riparia]	vitis riparia	29760.VIT_12s0028g03340.t01	[illegible]	[illegible]
endless_summer2_g38814	endless_summer2_g38814	Endless Summer genom	Alternative assembl	tr|A0A7J7FV21|A0A7J7FV21_CAMSI	30.9	tr|A0A7J7FV21|A0A7J7FV21_CAMSI Asp_protease domain-containing protein OS=Camellia sinensis OX=4442 GN=HYC85	camellia sinensis	39947.LOC_Os09g27300.1	[illegible]	[illegible]
endless_summer2_g39145	endless_summer2_g39145	Endless Summer genom	Alternative assembl	XP_028063962.1	44.1	XP_028063962.1 disease resistance protein RPP13-like [Camellia sinensis]	camellia sinensis	4113.PGSC0003DMT400056264	[illegible]	[illegible]
endless_summer2_g39162	endless_summer2_g39162	Endless Summer genom	Alternative assembl	XP_022894259.1	68.3	XP_022894259.1 scarecrow-like protein 1 [Olea europaea var. sylvestris]	olea europaea var. sylvestri	29760.VIT_18s0001g15270.t01	[illegible]	[illegible]
endless_summer2_g39163	endless_summer2_g39163	Endless Summer genom	Alternative assembl	XP_042492711.1	84	XP_042492711.1 ribosome maturation protein SBDS [Macadamia integrifolia]	macadamia integrifolia	29760.VIT_18s0001g15290.t01	[illegible]	[illegible]
endless_summer2_g39461	endless_summer2_g39461	Endless Summer genom	Alternative assembl	XP_034681005.1	30	XP_034681005.1 uncharacterized protein LOC117910933 [Vitis riparia]	vitis riparia	29760.VIT_18s0041g00480.t01	[illegible]	[illegible]
endless_summer2_g3964	endless_summer2_g3964	Endless Summer genom	Alternative assembl	XP_028065066.1	69.5	XP_028065066.1 probable receptor-like protein kinase At1g11050 [Camellia sinensis]	camellia sinensis	29760.VIT_12s0034g02220.t01	[illegible]	[illegible]
endless_summer2_g397	endless_summer2_g397	Endless Summer genom	Alternative assembl	XP_024041750.1	27.7	XP_024041750.1 uncharacterized protein LOC112098966 [Citrus clementina]	citrus clementina	39947.LOC_Os10g04640.1	[illegible]	[illegible]
endless_summer2_g3973	endless_summer2_g3973	Endless Summer genom	Alternative assembl	XP_020243329.1	40.2	XP_020243329.1 uncharacterized protein LOC109821560 [Asparagus officinalis]	asparagus officinalis		[illegible]	[illegible]
endless_summer2_g39781	endless_summer2_g39781	Endless Summer genom	Alternative assembl	tr|A0A2N9FGT8|A0A2N9FGT8_FAGSY	46.4	tr|A0A2N9FGT8|A0A2N9FGT8_FAGSY RNA-directed DNA polymerase OS=Fagus sylvatica OX=28930 GN=FSB_LOCUS1	fagus sylvatica	39947.LOC_Os01g23330.1	[illegible]	[illegible]
endless_summer2_g40036	endless_summer2_g40036	Endless Summer genom	Alternative assembl	tr|A0A4Q1ZSZ5|A0A4Q1ZSZ5_9LACO	57.8	tr|A0A4Q1ZSZ5|A0A4Q1ZSZ5_9LACO Reverse transcriptase domain-containing protein (Fragment) OS=Ligilactobacillus muri	ligilactobacillus murinus	4081.Solyc08g060910.1.1	[illegible]	[illegible]
endless_summer2_g40111	endless_summer2_g40111	Endless Summer genom	Alternative assembl	tr|A0A2T7G1X9|A0A2T7G1X9_9RHOB	40.5	tr|A0A2T7G1X9|A0A2T7G1X9_9RHOB Reverse transcriptase Ty1/copia-type domain-containing protein (Fragment) OS=Pelag	pelagivirga sediminicola	39947.LOC_Os05g45550.1	[illegible]	[illegible]
endless_summer2_g4015	endless_summer2_g4015	Endless Summer genom	Alternative assembl	tr|A0A5B7ATY8|A0A5B7ATY8_DAVIN	51.8	tr|A0A5B7ATY8|A0A5B7ATY8_DAVIN F-box domain-containing protein OS=Davidia involucrata OX=16924 GN=Din_0295	davidia involucrata	4113.PGSC0003DMT400070878	[illegible]	[illegible]
endless_summer2_g4016	endless_summer2_g4016	Endless Summer genom	Alternative assembl	tr|B7ES14|B7ES14_ORYSJ	36.1	tr|B7ES14|B7ES14_ORYSJ Os09g0480300 protein OS=Oryza sativa subsp. japonica OX=39947 GN=Os09g0480300 PE=2 SV	oryza sativa subsp. japonic	39947.LOC_Os04g33010.1	[illegible]	[illegible]
endless_summer2_g40230	endless_summer2_g40230	Endless Summer genom	Alternative assembl	tr|A0A7J7FZ96|A0A7J7FZ96_CAMSI	26.7	tr|A0A7J7FZ96|A0A7J7FZ96_CAMSI RRM domain-containing protein OS=Camellia sinensis OX=4442 GN=HYC85_029676	camellia sinensis	3847.GLYMA02G25024.1	[illegible]	[illegible]
endless_summer2_g4024	endless_summer2_g4024	Endless Summer genom	Alternative assembl	XP_034700039.1	48.7	XP_034700039.1 nuclear pore complex protein NUP1 isoform X1 [Vitis riparia]	vitis riparia	29760.VIT_11s0016g05470.t01	[illegible]	[illegible]
endless_summer2_g40244	endless_summer2_g40244	Endless Summer genom	Alternative assembl	XP_009608201.1	97.1	XP_009608201.1 tubulin beta-1 chain [Nicotiana tomentosiformis]	nicotiana tomentosiformis	29760.VIT_18s0001g14360.t01	Tubulin is the major constituent of microtubules. It binds two moles of G	[illegible]
endless_summer2_g40308	endless_summer2_g40308	Endless Summer genom	Alternative assembl	tr|A0A2U1Q365|A0A2U1Q365_ARTAN	29.4	tr|A0A2U1Q365|A0A2U1Q365_ARTAN RNase H type-1 domain-containing protein OS=Artemisia annua OX=35608 GN=CTI	artemisia annua	3694.POPTR_0019s01390.1	[illegible]	[illegible]
endless_summer2_g40332	endless_summer2_g40332	Endless Summer genom	Alternative assembl	tr|A0A834LPU3|A0A834LPU3_RHOSS	58.4	tr|A0A834LPU3|A0A834LPU3_RHOSS Retrotran_gag_3 domain-containing protein OS=Rhododendron simsii OX=118357 GN	rhododendron simsii	29760.VIT_09s0054g01090.t01	[illegible]	[illegible]
endless_summer2_g40523	endless_summer2_g40523	Endless Summer genom	Alternative assembl	tr|A0A834G5Z1|A0A834G5Z1_RHOSS	36.3	tr|A0A834G5Z1|A0A834G5Z1_RHOSS CCHC-type domain-containing protein OS=Rhododendron simsii OX=118357 GN=RI	rhododendron simsii	39947.LOC_Os11g14330.1	[illegible]	[illegible]
endless_summer2_g40570	endless_summer2_g40570	Endless Summer genom	Alternative assembl	tr|A0A7N2M120|A0A7N2M120_QUELO	33.7	tr|A0A7N2M120|A0A7N2M120_QUELO Uncharacterized protein OS=Quercus lobata OX=97700 PE=4 SV=1	quercus lobata	4081.Solyc10g074430.1.1	[illegible]	[illegible]
endless_summer2_g4065	endless_summer2_g4065	Endless Summer genom	Alternative assembl	XP_042967066.1	72.4	XP_042967066.1 uncharacterized protein LOC122300460 [Carya illinoinensis]	carya illinoinensis	3694.POPTR_0008s20920.1	[illegible]	[illegible]
endless_summer2_g40673	endless_summer2_g40673	Endless Summer genom	Alternative assembl	XP_007220613.2	68.6	XP_007220613.2 cyclic nucleotide-gated ion channel 1 isoform X1 [Prunus persica]	prunus persica	29760.VIT_16s0050g02560.t01	[illegible]	[illegible]
endless_summer2_g40679	endless_summer2_g40679	Endless Summer genom	Alternative assembl	XP_034709393.1	64.1	XP_034709393.1 DEAD-box ATP-dependent RNA helicase 31-like [Vitis riparia]	vitis riparia	29760.VIT_15s0046g01010.t01	[illegible]	[illegible]
endless_summer2_g40780	endless_summer2_g40780	Endless Summer genom	Alternative assembl	tr|A0A443Q7W0|A0A443Q7W0_9ACAR	29.6	tr|A0A443Q7W0|A0A443Q7W0_9ACAR DUF659 domain-containing protein OS=Dinothrombium tinctorium OX=1965070 GI	dinothrombium tinctorium	39946.BGIOSGA038560-PA	[illegible]	[illegible]
endless_summer2_g40806	endless_summer2_g40806	Endless Summer genom	Alternative assembl	tr|A0A5D3CRP8|A0A5D3CRP8_CUCME	36.4	tr|A0A5D3CRP8|A0A5D3CRP8_CUCME RNA-directed DNA polymerase (Reverse transcriptase), Ribonuclease H-like protein (	cucumis melo var. makuwa	4113.PGSC0003DMT400083953	[illegible]	[illegible]
endless_summer2_g40835	endless_summer2_g40835	Endless Summer genom	Alternative assembl	XP_022034108.1	73.9	XP_022034108.1 perakine reductase isoform X1 [Helianthus annuus]	helianthus annuus	4081.Solyc01g097390.2.1	[illegible]	[illegible]
endless_summer2_g4091	endless_summer2_g4091	Endless Summer genom	Alternative assembl	XP_028118238.1	45.3	XP_028118238.1 nucleolar protein dao-5-like [Camellia sinensis]	camellia sinensis	29760.VIT_08s0040g02430.t01	[illegible]	[illegible]
endless_summer2_g41004	endless_summer2_g41004	Endless Summer genom	Alternative assembl	XP_022894027.1	40.5	XP_022894027.1 uncharacterized protein LOC111408512 [Olea europaea var. sylvestris]	olea europaea var. sylvestri	29760.VIT_07s0031g02450.t01	[illegible]	[illegible]
endless_summer2_g41019	endless_summer2_g41019	Endless Summer genom	Alternative assembl	tr|A0A7J7FVW1|A0A7J7FVW1_CAMSI	35.5	tr|A0A7J7FVW1|A0A7J7FVW1_CAMSI SWIM-type domain-containing protein OS=Camellia sinensis OX=4442 GN=HYC85	camellia sinensis	29760.VIT_14s0060g00370.t01	[illegible]	[illegible]
endless_summer2_g41041	endless_summer2_g41041	Endless Summer genom	Alternative assembl	tr|A0A5B6ZLW5|A0A5B6ZLW5_DAVIN	30.4	tr|A0A5B6ZLW5|A0A5B6ZLW5_DAVIN Uncharacterized protein (Fragment) OS=Davidia involucrata OX=16924 GN=Din_01(	davidia involucrata		[illegible]	[illegible]
endless_summer2_g41072	endless_summer2_g41072	Endless Summer genom	Alternative assembl	tr|A0A7J0HGA2|A0A7J0HGA2_9ERIC	93.5	tr|A0A7J0HGA2|A0A7J0HGA2_9ERIC Uncharacterized protein OS=Actinidia rufa OX=165716 GN=Acr_00g0000030 PE=4 SV	actinidia rufa		[illegible]	[illegible]
endless_summer2_g41101	endless_summer2_g41101	Endless Summer genom	Alternative assembl	tr|A0A7W1HLN3|A0A7W1HLN3_9BACT	61	tr|A0A7W1HLN3|A0A7W1HLN3_9BACT 30S ribosomal protein S12 OS=Chthoniobacterales bacterium OX=2201154 GN=rp	chthoniobacterales bacteriu	4555.Si020787m	[illegible]	[illegible]
endless_summer2_g41117	endless_summer2_g41117	Endless Summer genom	Alternative assembl	XP_028089617.1	70.7	XP_028089617.1 protein ELC-like [Camellia sinensis]	camellia sinensis	29760.VIT_05s0029g01060.t01	[illegible]	[illegible]
endless_summer2_g41286	endless_summer2_g41286	Endless Summer genom	Alternative assembl	tr|A0A2N9GMV5|A0A2N9GMV5_FAGSY	31.1	tr|A0A2N9GMV5|A0A2N9GMV5_FAGSY Uncharacterized protein OS=Fagus sylvatica OX=28930 GN=FSB_LOCUS28602 P	fagus sylvatica	39947.LOC_Os02g14520.1	[illegible]	[illegible]
endless_summer2_g41332	endless_summer2_g41332	Endless Summer genom	Alternative assembl	XP_028102989.1	72.6	XP_028102989.1 LOW QUALITY PROTEIN: polyamine oxidase 1 [Camellia sinensis]	camellia sinensis	3694.POPTR_0001s27060.1	[illegible]	[illegible]
endless_summer2_g41383	endless_summer2_g41383	Endless Summer genom	Alternative assembl	tr|A0A5J4ZQR7|A0A5J4ZQR7_9ASTE	39.9	tr|A0A5J4ZQR7|A0A5J4ZQR7_9ASTE FBA_3 domain-containing protein OS=Nyssa sinensis OX=561372 GN=F0562_01448	nyssa sinensis	29760.VIT_11s0065g00730.t01	[illegible]	[illegible]
endless_summer2_g41426	endless_summer2_g41426	Endless Summer genom	Alternative assembl	tr|A0A5B7CC48|A0A5B7CC48_DAVIN	58.5	tr|A0A5B7CC48|A0A5B7CC48_DAVIN Toprim domain-containing protein (Fragment) OS=Davidia involucrata OX=16924 GN	davidia involucrata	4081.Solyc02g081910.1.1	[illegible]	[illegible]
endless_summer2_g41532	endless_summer2_g41532	Endless Summer genom	Alternative assembl	tr|A0A3Q7GWB6|A0A3Q7GWB6_SOLLC	48.5	tr|A0A3Q7GWB6|A0A3Q7GWB6_SOLLC Reverse transcriptase Ty1/copia-type domain-containing protein OS=Solanum lyco	solanum lycopersicum	4081.Solyc11g062200.1.1	[illegible]	[illegible]
endless_summer2_g41595	endless_summer2_g41595	Endless Summer genom	Alternative assembl	XP_007034811.1	45.6	XP_007034811.1 PREDICTED: uncharacterized protein LOC18603023 [Theobroma cacao]	theobroma cacao	3847.GLYMA20G28472.1	[illegible]	[illegible]
endless_summer2_g4160	endless_summer2_g4160	Endless Summer genom	Alternative assembl	tr|A0A5B7ABB8|A0A5B7ABB8_DAVIN	53	tr|A0A5B7ABB8|A0A5B7ABB8_DAVIN Putative alpha-latrocrustotoxin-Lt1a-like isoform X1 (Fragment) OS=Davidia involuc	davidia involucrata	3694.POPTR_0015s15000.1	[illegible]	[illegible]
endless_summer2_g4167	endless_summer2_g4167	Endless Summer genom	Alternative assembl	XP_028065156.1	76	XP_028065156.1 probable dolichyl pyrophosphate Man9GlcNAc2 alpha-1,3-glucosyltransferase [Camellia sinensis]	camellia sinensis	29760.VIT_14s0006g03000.t01	[illegible]	[illegible]
endless_summer2_g41703	endless_summer2_g41703	Endless Summer genom	Alternative assembl	XP_028074006.1	74.2	XP_028074006.1 AP2-associated protein kinase 1-like [Camellia sinensis]	camellia sinensis	4113.PGSC0003DMT400016206	[illegible]	[illegible]

endless_summer2_g41740	endless_summer2_g41740	Endless Summer genom	Alternative assembl	tr|A0A443P6A9|A0A443P6A9_9MAGN	31.7	tr|A0A443P6A9|A0A443P6A9_9MAGN Transposase, Ptta/En/Spm, plant OS=Cinnamomum micranthum f. kanehirae OX=337	cinnamomum micranthum f.	4113.PGSC0003DMT400017273	[illegible]	[illegible]
endless_summer2_g41745	endless_summer2_g41745	Endless Summer genom	Alternative assembl	tr|A0A2R6RAC2|A0A2R6RAC2_ACTCC	51.5	tr|A0A2R6RAC2|A0A2R6RAC2_ACTCC Nucleoredoxin OS=Actinidia chinensis var. chinensis OX=1590841 GN=CEY00_Ac	actinidia chinensis var. chi	3694.POPTR_0001s23700.1	[illegible]	[illegible]
endless_summer2_g41914	endless_summer2_g41914	Endless Summer genom	Alternative assembl	tr|A0A7N2L026|A0A7N2L026_QUELO	37.9	tr|A0A7N2L026|A0A7N2L026_QUELO Uncharacterized protein OS=Quercus lobata OX=97700 PE=4 SV=1	quercus lobata	3694.POPTR_0003s19090.1	[illegible]	[illegible]
endless_summer2_g41927	endless_summer2_g41927	Endless Summer genom	Alternative assembl	XP_024926953.1	43	XP_024926953.1 uncharacterized protein LOC112490735 isoform X2 [Ziziphus jujuba]	ziziphus jujuba	15368.BRADI2G29227.1	[illegible]	[illegible]
endless_summer2_g42003	endless_summer2_g42003	Endless Summer genom	Alternative assembl	tr|A0A7J9AQT3|A0A7J9AQT3_9ROSI	25	tr|A0A7J9AQT3|A0A7J9AQT3_9ROSI RRM domain-containing protein OS=Gossypium laxum OX=34288 GN=Golax_02234	gossypium laxum	3847.GLYMA02G15561.1	[illegible]	[illegible]
endless_summer2_g4216	endless_summer2_g4216	Endless Summer genom	Alternative assembl	tr|A0A5B7BPQ8|A0A5B7BPQ8_DAVIN	28.9	tr|A0A5B7BPQ8|A0A5B7BPQ8_DAVIN Phorbol-ester/DAG-type domain-containing protein (Fragment) OS=Davidia involucra	davidia involucrata	3694.POPTR_0019s03690.1	[illegible]	[illegible]
endless_summer2_g42346	endless_summer2_g42346	Endless Summer genom	Alternative assembl	tr|A0A438GTG5|A0A438GTG5_VITVI	63.5	tr|A0A438GTG5|A0A438GTG5_VITVI Transposon Ty3-I Gag-Pol polyprotein OS=Vitis vinifera OX=29760 GN=TY3B-I_865	vitis vinifera	3694.POPTR_0011s01230.1	[illegible]	[illegible]
endless_summer2_g42404	endless_summer2_g42404	Endless Summer genom	Alternative assembl	tr|A0A2J6MDC6|A0A2J6MDC6_LACSA	65.8	tr|A0A2J6MDC6|A0A2J6MDC6_LACSA Dimer_Tnp_hAT domain-containing protein OS=Lactuca sativa OX=4236 GN=LSAT	lactuca sativa	4577.GRMZM2G036637_P01	[illegible]	[illegible]
endless_summer2_g42564	endless_summer2_g42564	Endless Summer genom	Alternative assembl	XP_020080518.1	26.9	XP_020080518.1 uncharacterized protein LOC109704178 [Ananas comosus]	ananas comosus	4533.OB06G16940.1	[illegible]	[illegible]
endless_summer2_g42565	endless_summer2_g42565	Endless Summer genom	Alternative assembl	XP_028059693.1	29.7	XP_028059693.1 uncharacterized protein LOC114263376 [Camellia sinensis]	camellia sinensis		[illegible]	[illegible]
endless_summer2_g42608	endless_summer2_g42608	Endless Summer genom	Alternative assembl	XP_022848674.1	69.2	XP_022848674.1 uncharacterized protein LOC111370980 [Olea europaea var. sylvestris]	olea europaea var. sylvestri	4577.GRMZM2G053744_P01	[illegible]	[illegible]
endless_summer2_g42647	endless_summer2_g42647	Endless Summer genom	Alternative assembl	XP_028076868.1	48.9	XP_028076868.1 uncharacterized protein LOC114278908 [Camellia sinensis]	camellia sinensis	4558.Sb03g023051.1	[illegible]	[illegible]
endless_summer2_g42666	endless_summer2_g42666	Endless Summer genom	Alternative assembl	XP_028126744.1	46.4	XP_028126744.1 PKS-NRPS hybrid synthetase CHGG_01239-like [Camellia sinensis]	camellia sinensis	29760.VIT_03s0038g03060.t01	[illegible]	[illegible]
endless_summer2_g42802	endless_summer2_g42802	Endless Summer genom	Alternative assembl	XP_034708660.1	58.6	XP_034708660.1 O-glucosyltransferase rumi-like [Vitis riparia]	vitis riparia	29760.VIT_15s0046g03070.t01	[illegible]	[illegible]
endless_summer2_g4293	endless_summer2_g4293	Endless Summer genom	Alternative assembl	XP_004295618.1	44.6	XP_004295618.1 PREDICTED: uncharacterized protein LOC101300039 [Fragaria vesca subsp. vesca]	fragaria vesca subsp. vesca	3847.GLYMA03G24720.1	[illegible]	[illegible]
endless_summer2_g42997	endless_summer2_g42997	Endless Summer genom	Alternative assembl	tr|A0A7J0EPS6|A0A7J0EPS6_9ERIC	32.2	tr|A0A7J0EPS6|A0A7J0EPS6_9ERIC Retrotrans_gag domain-containing protein OS=Actinidia rufa OX=165716 GN=Acr_05g0	actinidia rufa	39947.LOC_Os05g38260.1	[illegible]	[illegible]
endless_summer2_g43030	endless_summer2_g43030	Endless Summer genom	Alternative assembl	XP_047182601.1	58.5	XP_047182601.1 cyanogenic beta-glucosidase-like [Vigna umbellata]	vigna umbellata	3847.GLYMA11G13820.1	[illegible]	[illegible]
endless_summer2_g431	endless_summer2_g431	Endless Summer genom	Alternative assembl	tr|A0A5B7BUC8|A0A5B7BUC8_DAVIN	36.4	tr|A0A5B7BUC8|A0A5B7BUC8_DAVIN HMG box domain-containing protein (Fragment) OS=Davidia involucrata OX=16924	davidia involucrata	4081.Solyc03g043600.1.1	[illegible]	[illegible]
endless_summer2_g4321	endless_summer2_g4321	Endless Summer genom	Alternative assembl	tr|A0A4U2KJ40|A0A4U2KJ40_9BACT	37.5	tr|A0A4U2KJ40|A0A4U2KJ40_9BACT Ribonuclease HI family protein (Fragment) OS=Puteibacter caeruleilacunae OX=252863	puteibacter caeruleilacunae	4641.GSMUA_AchrUn_randomP11040_001	[illegible]	[illegible]
endless_summer2_g4324	endless_summer2_g4324	Endless Summer genom	Alternative assembl	XP_020417382.1	50.8	XP_020417382.1 protein SUPPRESSOR OF GENE SILENCING 3 [Prunus persica]	prunus persica	29760.VIT_07s0130g00190.t01	[illegible]	[illegible]
endless_summer2_g4341	endless_summer2_g4341	Endless Summer genom	Alternative assembl	tr|A0A2P5C456|A0A2P5C456_TREOI	87.6	tr|A0A2P5C456|A0A2P5C456_TREOI Squalene cyclase OS=Trema orientale OX=63057 GN=TorRG33x02_298300 PE=3 SV=	trema orientale	29760.VIT_09s0054g01220.t01	[illegible]	[illegible]
endless_summer2_g4343	endless_summer2_g4343	Endless Summer genom	Alternative assembl	tr|A0A6P5TQA3|A0A6P5TQA3_PRUAV	60.3	tr|A0A6P5TQA3|A0A6P5TQA3_PRUAV zinc finger MYM-type protein 1-like OS=Prunus avium OX=42229 GN=LOC110769	prunus avium	3694.POPTR_0004s03580.1	[illegible]	[illegible]
endless_summer2_g43558	endless_summer2_g43558	Endless Summer genom	Alternative assembl	XP_028085857.1	44.6	XP_028085857.1 uncharacterized protein LOC114286832 [Camellia sinensis]	camellia sinensis	148304.MAPG_11083T0	[illegible]	[illegible]
endless_summer2_g43625	endless_summer2_g43625	Endless Summer genom	Alternative assembl	tr|A0A438D317|A0A438D317_VITVI	68.3	tr|A0A438D317|A0A438D317_VITVI Retrovirus-related Pol polyprotein from transposon TNT 1-94 OS=Vitis vinifera OX=297	vitis vinifera	39947.LOC_Os06g22090.1	[illegible]	[illegible]
endless_summer2_g43676	endless_summer2_g43676	Endless Summer genom	Alternative assembl	tr|A0A368TLS3|A0A368TLS3_9EURY	31.8	tr|A0A368TLS3|A0A368TLS3_9EURY Sulfate permease, MFS superfamily (Fragment) OS=Methanophagales archaeon OX=20	methanophagales archaeon	3694.POPTR_0008s12940.1	[illegible]	[illegible]
endless_summer2_g43946	endless_summer2_g43946	Endless Summer genom	Alternative assembl	tr|A0A835HLR9|A0A835HLR9_9MAGN	28.3	tr|A0A835HLR9|A0A835HLR9_9MAGN PMD domain-containing protein OS=Coptis chinensis OX=261450 GN=IFM89_0345	coptis chinensis	3694.POPTR_0001s20780.1	[illegible]	[illegible]
endless_summer2_g4396	endless_summer2_g4396	Endless Summer genom	Alternative assembl	tr|A0A6P9DWN3|A0A6P9DWN3_JUGRE	55	tr|A0A6P9DWN3|A0A6P9DWN3_JUGRE uncharacterized protein LOC118344033 OS=Juglans regia OX=51240 GN=LOC118	juglans regia	4081.Solyc00g042640.1.1	[illegible]	[illegible]
endless_summer2_g44010	endless_summer2_g44010	Endless Summer genom	Alternative assembl	XP_044482817.1	78.9	XP_044482817.1 pentatricopeptide repeat-containing protein At5g27270 isoform X2 [Mangifera indica]	mangifera indica	3694.POPTR_0019s07590.1	[illegible]	[illegible]
endless_summer2_g44076	endless_summer2_g44076	Endless Summer genom	Alternative assembl	tr|F6H8F5|F6H8F5_VITVI	81.2	tr|F6H8F5|F6H8F5_VITVI Cellulase (Fragment) OS=Vitis vinifera OX=29760 GN=VIT_00s2620g00010 PE=3 SV=1	vitis vinifera	39946.BGIOSGA014799-PA	[illegible]	[illegible]
endless_summer2_g44156	endless_summer2_g44156	Endless Summer genom	Alternative assembl	tr|A0A6J5XPR1|A0A6J5XPR1_PRUAR	54.2	tr|A0A6J5XPR1|A0A6J5XPR1_PRUAR SWIM-type domain-containing protein OS=Prunus armeniaca OX=36596 GN=CURH	prunus armeniaca	39947.LOC_Os02g48930.1	[illegible]	[illegible]
endless_summer2_g44286	endless_summer2_g44286	Endless Summer genom	Alternative assembl	XP_028108386.1	49.6	XP_028108386.1 dammarenediol 12-hydroxylase-like [Camellia sinensis]	camellia sinensis	29760.VIT_04s0008g02960.t01	[illegible]	[illegible]
endless_summer2_g44357	endless_summer2_g44357	Endless Summer genom	Alternative assembl	tr|A0A7J7FXC8|A0A7J7FXC8_CAMSI	32.2	tr|A0A7J7FXC8|A0A7J7FXC8_CAMSI Uncharacterized protein OS=Camellia sinensis OX=4442 GN=HYC85_029214 PE=4 S	camellia sinensis		[illegible]	[illegible]
endless_summer2_g44412	endless_summer2_g44412	Endless Summer genom	Alternative assembl	tr|A5C0B1|A5C0B1_VITVI	33.9	tr|A5C0B1|A5C0B1_VITVI PMD domain-containing protein OS=Vitis vinifera OX=29760 GN=VITISV_000588 PE=4 SV=1	vitis vinifera	3694.POPTR_0001s20780.1	[illegible]	[illegible]
endless_summer2_g44425	endless_summer2_g44425	Endless Summer genom	Alternative assembl	tr|A0A2N9GY06|A0A2N9GY06_FAGSY	38.1	tr|A0A2N9GY06|A0A2N9GY06_FAGSY Uncharacterized protein OS=Fagus sylvatica OX=28930 GN=FSB_LOCUS32122 PE	fagus sylvatica	39947.LOC_Os01g06700.1	[illegible]	[illegible]
endless_summer2_g4448	endless_summer2_g4448	Endless Summer genom	Alternative assembl	XP_028101941.1	84.8	XP_028101941.1 probable pectate lyase 8 [Camellia sinensis]	camellia sinensis	29760.VIT_07s0005g05520.t01	[illegible]	[illegible]
endless_summer2_g44490	endless_summer2_g44490	Endless Summer genom	Alternative assembl	XP_045817623.1	72.6	XP_045817623.1 peroxisome biogenesis protein 12-like [Trifolium pratense]	trifolium pratense	3847.GLYMA19G43700.2	[illegible]	[illegible]
endless_summer2_g44547	endless_summer2_g44547	Endless Summer genom	Alternative assembl	tr|A0A7J0ERT3|A0A7J0ERT3_9ERIC	65.8	tr|A0A7J0ERT3|A0A7J0ERT3_9ERIC Retrotran_gag_3 domain-containing protein OS=Actinidia rufa OX=165716 GN=Acr_06	actinidia rufa	3694.POPTR_0018s07320.1	[illegible]	[illegible]
endless_summer2_g44548	endless_summer2_g44548	Endless Summer genom	Alternative assembl	tr|A0A5B7BAK4|A0A5B7BAK4_DAVIN	56.6	tr|A0A5B7BAK4|A0A5B7BAK4_DAVIN Uncharacterized protein OS=Davidia involucrata OX=16924 GN=Din_035380 PE=4 S	davidia involucrata	4081.Solyc06g060030.1.1	[illegible]	[illegible]
endless_summer2_g44560	endless_summer2_g44560	Endless Summer genom	Alternative assembl	tr|A0A5B7BJH8|A0A5B7BJH8_DAVIN	66	tr|A0A5B7BJH8|A0A5B7BJH8_DAVIN RNA helicase OS=Davidia involucrata OX=16924 GN=Din_038400 PE=4 SV=1	davidia involucrata	29760.VIT_07s0005g00860.t01	[illegible]	[illegible]
endless_summer2_g4466	endless_summer2_g4466	Endless Summer genom	Alternative assembl	XP_028060069.1	78.5	XP_028060069.1 zinc finger HIT domain-containing protein 2 [Camellia sinensis]	camellia sinensis	29760.VIT_02s0025g02330.t01	[illegible]	[illegible]
endless_summer2_g4467	endless_summer2_g4467	Endless Summer genom	Alternative assembl	XP_010106141.1	79.2	XP_010106141.1 plant UBX domain-containing protein 10 [Morus notabilis]	morus notabilis	4081.Solyc08g008510.2.1	[illegible]	[illegible]
endless_summer2_g44687	endless_summer2_g44687	Endless Summer genom	Alternative assembl	tr|A0A5B7BZM5|A0A5B7BZM5_DAVIN	40	tr|A0A5B7BZM5|A0A5B7BZM5_DAVIN Retrotrans_gag domain-containing protein (Fragment) OS=Davidia involucrata OX=1	davidia involucrata	29760.VIT_00s0823g00010.t01	[illegible]	[illegible]
endless_summer2_g44716	endless_summer2_g44716	Endless Summer genom	Alternative assembl	XP_028081905.1	53.8	XP_028081905.1 uncharacterized protein LOC114283284 [Camellia sinensis]	camellia sinensis	3847.GLYMA20G36922.1	[illegible]	[illegible]
endless_summer2_g44781	endless_summer2_g44781	Endless Summer genom	Alternative assembl	tr|A0A3S3N564|A0A3S3N564_9MAGN	69.9	tr|A0A3S3N564|A0A3S3N564_9MAGN LRR.XII-like protein OS=Cinnamomum micranthum f. kanehirae OX=337451 GN=CK	cinnamomum micranthum f.	29760.VIT_09s0018g01080.t01	[illegible]	[illegible]
endless_summer2_g44855	endless_summer2_g44855	Endless Summer genom	Alternative assembl	tr|A0A6J1BCB5|A0A6J1BCB5_9ROSI	63.8	tr|A0A6J1BCB5|A0A6J1BCB5_9ROSI LOW QUALITY PROTEIN: probable LRR receptor-like serine/threonine-protein kinase	herrania umbratica	29760.VIT_09s0018g01080.t01	[illegible]	[illegible]
endless_summer2_g45043	endless_summer2_g45043	Endless Summer genom	Alternative assembl	tr|A0A314Y598|A0A314Y598_PRUYE	81.6	tr|A0A314Y598|A0A314Y598_PRUYE Diphosphomevalonate decarboxylase MVD2-like OS=Prunus yedoensis var. nudiflora O	prunus yedoensis var. nudi	4113.PGSC0003DMT400031130	[illegible]	[illegible]
endless_summer2_g45046	endless_summer2_g45046	Endless Summer genom	Alternative assembl	tr|A0A5B6YSH7|A0A5B6YSH7_DAVIN	40.2	tr|A0A5B6YSH7|A0A5B6YSH7_DAVIN Uncharacterized protein OS=Davidia involucrata OX=16924 GN=Din_004046 PE=4 S	davidia involucrata	4558.Sb02g028950.1	[illegible]	[illegible]
endless_summer2_g45248	endless_summer2_g45248	Endless Summer genom	Alternative assembl	XP_040365497.1	24.5	XP_040365497.1 uncharacterized protein LOC121050192 isoform X2 [Rosa chinensis]	rosa chinensis	29760.VIT_13s0019g01850.t01	[illegible]	[illegible]
endless_summer2_g45368	endless_summer2_g45368	Endless Summer genom	Alternative assembl	tr|A0A5B7BHI6|A0A5B7BHI6_DAVIN	77.3	tr|A0A5B7BHI6|A0A5B7BHI6_DAVIN Uncharacterized protein OS=Davidia involucrata OX=16924 GN=Din_037831 PE=4 SV	davidia involucrata	29760.VIT_13s0019g03480.t01	[illegible]	[illegible]
endless_summer2_g45462	endless_summer2_g45462	Endless Summer genom	Alternative assembl	XP_035836993.1	44.8	XP_035836993.1 uncharacterized protein LOC110877269 [Helianthus annuus]	helianthus annuus	4081.Solyc02g062480.1.1	[illegible]	[illegible]
endless_summer2_g45478	endless_summer2_g45478	Endless Summer genom	Alternative assembl	XP_028084032.1	43.6	XP_028084032.1 uncharacterized protein LOC114285206 [Camellia sinensis]	camellia sinensis	39947.LOC_Os01g06700.1	[illegible]	[illegible]
endless_summer2_g45504	endless_summer2_g45504	Endless Summer genom	Alternative assembl	XP_042942647.1	38.8	XP_042942647.1 ATP-dependent DNA helicase PIF1-like [Carya illinoinensis]	carya illinoinensis	4558.Sb06g016400.1	[illegible]	[illegible]
endless_summer2_g45527	endless_summer2_g45527	Endless Summer genom	Alternative assembl	tr|A0A438H1R5|A0A438H1R5_VITVI	57	tr|A0A438H1R5|A0A438H1R5_VITVI Retrovirus-related Pol polyprotein from transposon TNT 1-94 OS=Vitis vinifera OX=29	vitis vinifera	29760.VIT_07s0005g06030.t01	[illegible]	[illegible]
endless_summer2_g45539	endless_summer2_g45539	Endless Summer genom	Alternative assembl	XP_023882197.1	32.7	XP_023882197.1 uncharacterized protein LOC111994540 [Quercus suber]	quercus suber	39946.BGIOSGA016647-PA	[illegible]	[illegible]
endless_summer2_g45567	endless_summer2_g45567	Endless Summer genom	Alternative assembl	tr|A0A4R5JFT5|A0A4R5JFT5_9FIRM	47.4	tr|A0A4R5JFT5|A0A4R5JFT5_9FIRM Reverse transcriptase domain-containing protein (Fragment) OS=Zhaonella formicivoran	zhaonella formicivorans	39947.LOC_Os07g23480.1	[illegible]	[illegible]
endless_summer2_g45639	endless_summer2_g45639	Endless Summer genom	Alternative assembl	tr|A0A815VJJ8|A0A815VJJ8_9BILA	44.9	tr|A0A815VJJ8|A0A815VJJ8_9BILA Hypothetical protein (Fragment) OS=Adineta steineri OX=433720 GN=JYZ213_LOCUS4	adineta steineri	39947.LOC_Os08g02990.1	[illegible]	[illegible]
endless_summer2_g45761	endless_summer2_g45761	Endless Summer genom	Alternative assembl	tr|A0A7N2R7F0|A0A7N2R7F0_QUELO	31.4	tr|A0A7N2R7F0|A0A7N2R7F0_QUELO ATP-dependent DNA helicase OS=Quercus lobata OX=97700 PE=3 SV=1	quercus lobata	39947.LOC_Os07g09300.1	[illegible]	[illegible]
endless_summer2_g45782	endless_summer2_g45782	Endless Summer genom	Alternative assembl	XP_028051953.1	84.7	XP_028051953.1 transcription termination factor MTERF4, chloroplastic-like [Camellia sinensis]	camellia sinensis	4081.Solyc11g017050.1.1	[illegible]	[illegible]
endless_summer2_g45874	endless_summer2_g45874	Endless Summer genom	Alternative assembl	tr|A0A7N2KQI9|A0A7N2KQI9_QUELO	46.8	tr|A0A7N2KQI9|A0A7N2KQI9_QUELO Helitron_like_N domain-containing protein OS=Quercus lobata OX=97700 PE=4 SV=	quercus lobata	39947.LOC_Os11g41190.1	[illegible]	[illegible]
endless_summer2_g45895	endless_summer2_g45895	Endless Summer genom	Alternative assembl	tr|A0A3N9PIQ9|A0A3N9PIQ9_9RHOB	42.4	tr|A0A3N9PIQ9|A0A3N9PIQ9_9RHOB Ribonuclease HI family protein (Fragment) OS=Rhodobacteraceae bacterium CH30 OX=	rhodobacteraceae bacterium	4113.PGSC0003DMT400038548	[illegible]	[illegible]
endless_summer2_g46013	endless_summer2_g46013	Endless Summer genom	Alternative assembl	XP_014523828.1	44.9	XP_014523828.1 uncharacterized protein LOC106780099 [Vigna radiata var. radiata]	vigna radiata var. radiata	29760.VIT_07s0095g00580.t01	[illegible]	[illegible]
endless_summer2_g46026	endless_summer2_g46026	Endless Summer genom	Alternative assembl	XP_021833930.1	76.2	XP_021833930.1 uncharacterized protein LOC110773712 [Prunus avium]	prunus avium	4081.Solyc03g096270.1.1	[illegible]	[illegible]
endless_summer2_g46052	endless_summer2_g46052	Endless Summer genom	Alternative assembl	tr|A0A5B6Z8U3|A0A5B6Z8U3_DAVIN	36.3	tr|A0A5B6Z8U3|A0A5B6Z8U3_DAVIN Retrotrans_gag domain-containing protein (Fragment) OS=Davidia involucrata OX=16	davidia involucrata	29760.VIT_05s0049g01920.t01	[illegible]	[illegible]
endless_summer2_g46063	endless_summer2_g46063	Endless Summer genom	Alternative assembl	XP_030923133.1	39	XP_030923133.1 uncharacterized protein LOC115950020 [Quercus lobata]	quercus lobata	29760.VIT_04s0008g06010.t01	[illegible]	[illegible]
endless_summer2_g46081	endless_summer2_g46081	Endless Summer genom	Alternative assembl	tr|A0A5B7BX00|A0A5B7BX00_DAVIN	50.8	tr|A0A5B7BX00|A0A5B7BX00_DAVIN PK_Tyr_Ser-Thr domain-containing protein OS=Davidia involucrata OX=16924 GN=	davidia involucrata	3694.POPTR_1358s00200.1	[illegible]	[illegible]
endless_summer2_g46133	endless_summer2_g46133	Endless Summer genom	Alternative assembl	XP_022850805.1	51.3	XP_022850805.1 uncharacterized protein LOC111372655 [Olea europaea var. sylvestris]	olea europaea var. sylvestri	4113.PGSC0003DMT400089863	[illegible]	[illegible]
endless_summer2_g46139	endless_summer2_g46139	Endless Summer genom	Alternative assembl	tr|A0A5B7BCS4|A0A5B7BCS4_DAVIN	87.7	tr|A0A5B7BCS4|A0A5B7BCS4_DAVIN ABC1 domain-containing protein (Fragment) OS=Davidia involucrata OX=16924 GN	davidia involucrata	4081.Solyc06g051730.2.1	[illegible]	[illegible]
endless_summer2_g46162	endless_summer2_g46162	Endless Summer genom	Alternative assembl	tr|A0A834GZD8|A0A834GZD8_RHOSS	67.5	tr|A0A834GZD8|A0A834GZD8_RHOSS Integrase catalytic domain-containing protein OS=Rhododendron simsii OX=118357 C	rhododendron simsii	39947.LOC_Os01g06700.1	[illegible]	[illegible]
endless_summer2_g46165	endless_summer2_g46165	Endless Summer genom	Alternative assembl	XP_023879450.1	35.4	XP_023879450.1 uncharacterized protein LOC111991867 [Quercus suber]	quercus suber	4577.GRMZM2G462450_P01	[illegible]	[illegible]
endless_summer2_g46209	endless_summer2_g46209	Endless Summer genom	Alternative assembl	tr|A0A5A7UGW6|A0A5A7UGW6_CUCMI	38.3	tr|A0A5A7UGW6|A0A5A7UGW6_CUCME PMD domain-containing protein OS=Cucumis melo var. makuwa OX=1194695 G	cucumis melo var. makuwa	4113.PGSC0003DMT400035683	[illegible]	[illegible]
endless_summer2_g4641	endless_summer2_g4641	Endless Summer genom	Alternative assembl	tr|A0A2N9IIP1|A0A2N9IIP1_FAGSY	65.9	tr|A0A2N9IIP1|A0A2N9IIP1_FAGSY Integrase catalytic domain-containing protein OS=Fagus sylvatica OX=28930 GN=FSB_I	fagus sylvatica	39947.LOC_Os08g22660.1	[illegible]	[illegible]
endless_summer2_g4645	endless_summer2_g4645	Endless Summer genom	Alternative assembl	tr|A0A5B6ZDP9|A0A5B6ZDP9_DAVIN	51.1	tr|A0A5B6ZDP9|A0A5B6ZDP9_DAVIN Uncharacterized protein OS=Davidia involucrata OX=16924 GN=Din_011339 PE=4 S	davidia involucrata	3694.POPTR_0003s18000.1	[illegible]	[illegible]
endless_summer2_g46635	endless_summer2_g46635	Endless Summer genom	Alternative assembl	tr|A0A7J6VNA4|A0A7J6VNA4_THATH	28.8	tr|A0A7J6VNA4|A0A7J6VNA4_THATH Uncharacterized protein OS=Thalictrum thalictroides OX=46969 GN=FRX31_024767	thalictrum thalictroides	39947.LOC_Os05g18980.1	[illegible]	[illegible]
endless_summer2_g4681	endless_summer2_g4681	Endless Summer genom	Alternative assembl	XP_028064709.1	66.3	XP_028064709.1 receptor-like protein EIX2 [Camellia sinensis]	camellia sinensis	29760.VIT_00s0397g00010.t01	[illegible]	[illegible]
endless_summer2_g46855	endless_summer2_g46855	Endless Summer genom	Alternative assembl	tr|A0A6J1E2J7|A0A6J1E2J7_MOMCH	25.1	tr|A0A6J1E2J7|A0A6J1E2J7_MOMCH LOW QUALITY PROTEIN: uncharacterized protein LOC111025431 OS=Momordica c	momordica charantia	29760.VIT_05s0049g01920.t01	[illegible]	[illegible]
endless_summer2_g46884	endless_summer2_g46884	Endless Summer genom	Alternative assembl	XP_028057937.1	68	XP_028057937.1 uncharacterized protein LOC114261823 [Camellia sinensis]	camellia sinensis	59689.fgenesh2_kg.7__1701__AT4G25590.1	[illegible]	[illegible]
endless_summer2_g4717	endless_summer2_g4717	Endless Summer genom	Alternative assembl	tr|A0A803L6Q2|A0A803L6Q2_CHEQI	31.5	tr|A0A803L6Q2|A0A803L6Q2_CHEQI CCHC-type domain-containing protein OS=Chenopodium quinoa OX=63459 PE=4 SV	chenopodium quinoa	4081.Solyc03g096270.1.1	[illegible]	[illegible]
endless_summer2_g47233	endless_summer2_g47233	Endless Summer genom	Alternative assembl	XP_028119178.1	76.4	XP_028119178.1 TPR repeat-containing protein ZIP4 [Camellia sinensis]	camellia sinensis	4113.PGSC0003DMT400056391	[illegible]	[illegible]
endless_summer2_g47262	endless_summer2_g47262	Endless Summer genom	Alternative assembl	XP_042513723.1	91.3	XP_042513723.1 calmodulin-like protein 3 [Macadamia integrifolia]	macadamia integrifolia	29760.VIT_07s0289g00040.t01	[illegible]	[illegible]
endless_summer2_g4729	endless_summer2_g4729	Endless Summer genom	Alternative assembl	tr|A0A438EQZ5|A0A438EQZ5_VITVI	63.5	tr|A0A438EQZ5|A0A438EQZ5_VITVI Premnaspirodiene oxygenase OS=Vitis vinifera OX=29760 GN=CYP71D55_5 PE=3 SV	vitis vinifera	29760.VIT_19s0015g00110.t01	[illegible]	[illegible]
endless_summer2_g47394	endless_summer2_g47394	Endless Summer genom	Alternative assembl	XP_030947448.1	51.9	XP_030947448.1 ATP-dependent DNA helicase PIF1-like [Quercus lobata]	quercus lobata	4577.GRMZM2G143961_P01	[illegible]	[illegible]
endless_summer2_g47413	endless_summer2_g47413	Endless Summer genom	Alternative assembl	XP_028100961.1	66.5	XP_028100961.1 UDP-glycosyltransferase 87A1-like isoform X1 [Camellia sinensis]	camellia sinensis	29760.VIT_06s0004g07310.t01	[illegible]	[illegible]
endless_summer2_g47433	endless_summer2_g47433	Endless Summer genom	Alternative assembl	tr|A0A2N9GYI1|A0A2N9GYI1_FAGSY	36.1	tr|A0A2N9GYI1|A0A2N9GYI1_FAGSY Uncharacterized protein OS=Fagus sylvatica OX=28930 GN=FSB_LOCUS35228 PE=	fagus sylvatica	39947.LOC_Os04g34810.1	[illegible]	[illegible]
endless_summer2_g47434	endless_summer2_g47434	Endless Summer genom	Alternative assembl	tr|A0A2R6P6W0|A0A2R6P6W0_ACTCC	76.4	tr|A0A2R6P6W0|A0A2R6P6W0_ACTCC Serine/threonine-protein kinase OS=Actinidia chinensis var. chinensis OX=1590841	actinidia chinensis var. chi	3847.GLYMA18G37650.2	[illegible]	[illegible]
endless_summer2_g47465	endless_summer2_g47465	Endless Summer genom	Alternative assembl	XP_030933951.1	63.2	XP_030933951.1 uncharacterized protein LOC115959627 [Quercus lobata]	quercus lobata	29760.VIT_07s0005g05640.t01	[illegible]	[illegible]
endless_summer2_g47698	endless_summer2_g47698	Endless Summer genom	Alternative assembl	tr|A0A7J7FX28|A0A7J7FX28_CAMSI	31.9	tr|A0A7J7FX28|A0A7J7FX28_CAMSI Uncharacterized protein OS=Camellia sinensis OX=4442 GN=HYC85_028868 PE=4 SV	camellia sinensis		[illegible]	[illegible]
endless_summer2_g47738	endless_summer2_g47738	Endless Summer genom	Alternative assembl	XP_003635108.2	36.8	XP_003635108.2 PREDICTED: HIV Tat-specific factor 1 [Vitis vinifera]	vitis vinifera	3847.GLYMA10G31450.1	[illegible]	[illegible]
endless_summer2_g47767	endless_summer2_g47767	Endless Summer genom	Alternative assembl	tr|A0A5B7C2Z5|A0A5B7C2Z5_DAVIN	46	tr|A0A5B7C2Z5|A0A5B7C2Z5_DAVIN CCHC-type domain-containing protein (Fragment) OS=Davidia involucrata OX=16924	davidia involucrata	3694.POPTR_0005s01170.1	[illegible]	[illegible]
endless_summer2_g47885	endless_summer2_g47885	Endless Summer genom	Alternative assembl	tr|A0A5J5BFG9|A0A5J5BFG9_9ASTE	56.7	tr|A0A5J5BFG9|A0A5J5BFG9_9ASTE Uncharacterized protein OS=Nyssa sinensis OX=561372 GN=F0562_023027 PE=4 SV	nyssa sinensis	4641.GSMUA_AchrUn_randomP14380_001	[illegible]	[illegible]
endless_summer2_g4789	endless_summer2_g4789	Endless Summer genom	Alternative assembl	tr|F6HF47|F6HF47_VITVI	76.4	tr|F6HF47|F6HF47_VITVI RING-type domain-containing protein OS=Vitis vinifera OX=29760 GN=VIT_01s0011g02160 PE=	vitis vinifera	29760.VIT_01s0011g02160.t01	[illegible]	[illegible]
endless_summer2_g47903	endless_summer2_g47903	Endless Summer genom	Alternative assembl	tr|A0A5J5CB55|A0A5J5CB55_9ASTE	54.2	tr|A0A5J5CB55|A0A5J5CB55_9ASTE CUE domain-containing protein OS=Nyssa sinensis OX=561372 GN=F0562_002575 P	nyssa sinensis	29760.VIT_07s0104g00310.t01	[illegible]	[illegible]
endless_summer2_g48151	endless_summer2_g48151	Endless Summer genom	Alternative assembl	XP_021656058.1	80.6	XP_021656058.1 ethylene response sensor 1-like isoform X1 [Hevea brasiliensis]	hevea brasiliensis	29760.VIT_07s0005g00850.t01	[illegible]	[illegible]
endless_summer2_g48226	endless_summer2_g48226	Endless Summer genom	Alternative assembl	tr|A0A822XHC6|A0A822XHC6_NELNU	53	tr|A0A822XHC6|A0A822XHC6_NELNU Uncharacterized protein OS=Nelumbo nucifera OX=4432 GN=HUJ06_019902 PE=4 S	nelumbo nucifera	3694.POPTR_0014s05430.1	[illegible]	[illegible]
endless_summer2_g48779	endless_summer2_g48779	Endless Summer genom	Alternative assembl	tr|A0A4V1T0C4|A0A4V1T0C4_9SPHI	41.3	tr|A0A4V1T0C4|A0A4V1T0C4_9SPHI gag_pre-integrs domain-containing protein OS=Sphingobacteriaceae bacterium OX=202	sphingobacteriaceae bacteri	29760.VIT_04s0008g04840.t01	[illegible]	[illegible]
endless_summer2_g48850	endless_summer2_g48850	Endless Summer genom	Alternative assembl	tr|A0A540M8S6|A0A540M8S6_MALBA	46.5	tr|A0A540M8S6|A0A540M8S6_MALBA SWIM-type domain-containing protein OS=Malus baccata OX=106549 GN=C1H46	malus baccata	39947.LOC_Os03g41570.1	[illegible]	[illegible]
endless_summer2_g48961	endless_summer2_g48961	Endless Summer genom	Alternative assembl	tr|A0A5J4ZLP6|A0A5J4ZLP6_9ASTE	86.6	tr|A0A5J4ZLP6|A0A5J4ZLP6_9ASTE Uncharacterized protein OS=Nyssa sinensis OX=561372 GN=F0562_014029 PE=4 SV=	nyssa sinensis	4081.Solyc02g078010.2.1	[illegible]	[illegible]
endless_summer2_g49137	endless_summer2_g49137	Endless Summer genom	Alternative assembl	tr|A0A5B7ASI2|A0A5B7ASI2_DAVIN	61.1	tr|A0A5B7ASI2|A0A5B7ASI2_DAVIN Uncharacterized protein OS=Davidia involucrata OX=16924 GN=Din_028416 PE=4 SV	davidia involucrata	29760.VIT_10s0116g00750.t01	[illegible]	[illegible]
endless_summer2_g492	endless_summer2_g492	Endless Summer genom	Alternative assembl	XP_017974545.1	44.5	XP_017974545.1 PREDICTED: uncharacterized protein LOC108661602 [Theobroma cacao]	theobroma cacao	39947.LOC_Os01g06700.1	[illegible]	[illegible]
endless_summer2_g49200	endless_summer2_g49200	Endless Summer genom	Alternative assembl	tr|A0A7J6W3S1|A0A7J6W3S1_THATH	56.2	tr|A0A7J6W3S1|A0A7J6W3S1_THATH Nuclease OS=Thalictrum thalictroides OX=46969 GN=FRX31_018385 PE=3 SV=1	thalictrum thalictroides	4577.GRMZM2G053744_P01	[illegible]	[illegible]
endless_summer2_g49232	endless_summer2_g49232	Endless Summer genom	Alternative assembl	tr|A0A5J5B2D9|A0A5J5B2D9_9ASTE	26.2	tr|A0A5J5B2D9|A0A5J5B2D9_9ASTE Uncharacterized protein OS=Nyssa sinensis OX=561372 GN=F0562_029276 PE=4 SV=	nyssa sinensis		[illegible]	[illegible]
endless_summer2_g49266	endless_summer2_g49266	Endless Summer genom	Alternative assembl	XP_028116970.1	84.9	XP_028116970.1 sugar carrier protein C-like [Camellia sinensis]	camellia sinensis	4113.PGSC0003DMT400057709	[illegible]	[illegible]
endless_summer2_g493	endless_summer2_g493	Endless Summer genom	Alternative assembl	XP_023916823.1	33	XP_023916823.1 uncharacterized protein LOC112028365 [Quercus suber]	quercus suber	3694.POPTR_0011s01230.1	[illegible]	[illegible]
endless_summer2_g49333	endless_summer2_g49333	Endless Summer genom	Alternative assembl	tr|A0A6A3BK95|A0A6A3BK95_HIBSY	49.8	tr|A0A6A3BK95|A0A6A3BK95_HIBSY Uncharacterized protein OS=Hibiscus syriacus OX=106335 GN=F3Y22_tig00110186	hibiscus syriacus	4081.Solyc03g096270.1.1	[illegible]	[illegible]
endless_summer2_g49461	endless_summer2_g49461	Endless Summer genom	Alternative assembl	XP_023871789.1	42.4	XP_023871789.1 uncharacterized protein LOC111984391 [Quercus suber]	quercus suber	39947.LOC_Os11g38300.1	[illegible]	[illegible]
endless_summer2_g49633	endless_summer2_g49633	Endless Summer genom	Alternative assembl	XP_028082352.1	38.7	XP_028082352.1 uncharacterized protein LOC114283672 isoform X3 [Camellia sinensis]	camellia sinensis	4577.GRMZM2G018788_P01	[illegible]	[illegible]
endless_summer2_g49651	endless_summer2_g49651	Endless Summer genom	Alternative assembl	tr|A0A498K730|A0A498K730_MALDO	59.9	tr|A0A498K730|A0A498K730_MALDO Ribulose bisphosphate carboxylase small subunit OS=Malus domestica OX=3750 GN=	malus domestica	29760.VIT_00s0391g00010.t01	[illegible]	[illegible]
endless_summer2_g49707	endless_summer2_g49707	Endless Summer genom	Alternative assembl	tr|A0A5J5B2U1|A0A5J5B2U1_9ASTE	29.6	tr|A0A5J5B2U1|A0A5J5B2U1_9ASTE Uncharacterized protein OS=Nyssa sinensis OX=561372 GN=F0562_029467 PE=4 SV=	nyssa sinensis		[illegible]	[illegible]
endless_summer2_g49820	endless_summer2_g49820	Endless Summer genom	Alternative assembl	XP_026378259.1	51.2	XP_026378259.1 zinc finger BED domain-containing protein RICESLEEPER 2-like [Papaver somniferum]	papaver somniferum	39947.LOC_Os10g08970.1	[illegible]	[illegible]
endless_summer2_g49937	endless_summer2_g49937	Endless Summer genom	Alternative assembl	XP_028096812.1	54	XP_028096812.1 protein TPX2-like [Camellia sinensis]	camellia sinensis	29760.VIT_12s0028g03330.t01	[illegible]	[illegible]
endless_summer2_g49968	endless_summer2_g49968	Endless Summer genom	Alternative assembl	tr|A0A834SVN3|A0A834SVN3_9FABA	59.2	tr|A0A834SVN3|A0A834SVN3_9FABA Uncharacterized protein OS=Senna tora OX=362788 GN=G2W53_037369 PE=4 SV=	senna tora	39947.LOC_Os04g16630.1	[illegible]	[illegible]
endless_summer2_g50041	endless_summer2_g50041	Endless Summer genom	Alternative assembl	tr|A0A7J0F995|A0A7J0F995_9ERIC	31.5	tr|A0A7J0F995|A0A7J0F995_9ERIC Retrotrans_gag domain-containing protein OS=Actinidia rufa OX=165716 GN=Acr_10g00	actinidia rufa		[illegible]	[illegible]
endless_summer2_g50129	endless_summer2_g50129	Endless Summer genom	Alternative assembl	XP_034700723.1	81.9	XP_034700723.1 NADPH:adrenodoxin oxidoreductase, mitochondrial isoform X1 [Vitis riparia]	vitis riparia	29760.VIT_11s0016g03550.t01	[illegible]	[illegible]
endless_summer2_g50361	endless_summer2_g50361	Endless Summer genom	Alternative assembl	XP_028067526.1	33.1	XP_028067526.1 uncharacterized protein LOC114270278 [Camellia sinensis]	camellia sinensis		[illegible]	[illegible]
endless_summer2_g50570	endless_summer2_g50570	Endless Summer genom	Alternative assembl	XP_028058048.1	84.1	XP_028058048.1 brefeldin A-inhibited guanine nucleotide-exchange protein 5 [Camellia sinensis]	camellia sinensis	29760.VIT_11s0016g00170.t01	[illegible]	[illegible]
endless_summer2_g50574	endless_summer2_g50574	Endless Summer genom	Alternative assembl	tr|A0A5B6YYC0|A0A5B6YYC0_DAVIN	54.7	tr|A0A5B6YYC0|A0A5B6YYC0_DAVIN BED-type domain-containing protein (Fragment) OS=Davidia involucrata OX=16924	davidia involucrata		[illegible]	[illegible]
endless_summer2_g50687	endless_summer2_g50687	Endless Summer genom	Alternative assembl	XP_034711579.1	54	XP_034711579.1 DNA-binding protein BIN4 isoform X1 [Vitis riparia]	vitis riparia	29760.VIT_16s0098g00630.t01	[illegible]	[illegible]
endless_summer2_g50710	endless_summer2_g50710	Endless Summer genom	Alternative assembl	tr|A0A2N9HZQ0|A0A2N9HZQ0_FAGSY	41.8	tr|A0A2N9HZQ0|A0A2N9HZQ0_FAGSY Retrotran_gag_3 domain-containing protein OS=Fagus sylvatica OX=28930 GN=FSI	fagus sylvatica	4577.GRMZM2G100647_P01	[illegible]	[illegible]
endless_summer2_g50771	endless_summer2_g50771	Endless Summer genom	Alternative assembl	XP_006441148.1	69.3	XP_006441148.1 acyl-CoA-binding domain-containing protein 1 isoform X1 [Citrus clementina]	citrus clementina	3847.GLYMA06G13630.1	[illegible]	[illegible]
endless_summer2_g50829	endless_summer2_g50829	Endless Summer genom	Alternative assembl	tr|A0A5J5BBJ2|A0A5J5BBJ2_9ASTE	75.3	tr|A0A5J5BBJ2|A0A5J5BBJ2_9ASTE Mur_ligase_M domain-containing protein OS=Nyssa sinensis OX=561372 GN=F0562	nyssa sinensis	29760.VIT_16s0050g02490.t01	[illegible]	[illegible]
endless_summer2_g50842	endless_summer2_g50842	Endless Summer genom	Alternative assembl	tr|A0A2N9I6M6|A0A2N9I6M6_FAGSY	57.7	tr|A0A2N9I6M6|A0A2N9I6M6_FAGSY RNA-directed DNA polymerase OS=Fagus sylvatica OX=28930 GN=FSB_LOCUS4	fagus sylvatica	39947.LOC_Os04g07710.1	[illegible]	[illegible]
endless_summer2_g50978	endless_summer2_g50978	Endless Summer genom	Alternative assembl	XP_028092800.1	34.7	XP_028092800.1 uncharacterized protein LOC114292993 [Camellia sinensis]	camellia sinensis	39947.LOC_Os02g32300.1	[illegible]	[illegible]
endless_summer2_g50997	endless_summer2_g50997	Endless Summer genom	Alternative assembl	tr|A0A5D2PGC5|A0A5D2PGC5_GOSTO	49.5	tr|A0A5D2PGC5|A0A5D2PGC5_GOSTO Uncharacterized protein OS=Gossypium tomentosum OX=34277 GN=ES332_A08G1	gossypium tomentosum	4555.Si015244m	[illegible]	[illegible]
endless_summer2_g5104	endless_summer2_g5104	Endless Summer genom	Alternative assembl	tr|A0A834FZI9|A0A834FZI9_RHOSS	49.8	tr|A0A834FZI9|A0A834FZI9_RHOSS Retrotrans_gag domain-containing protein OS=Rhododendron simsii OX=118357 GN=R	rhododendron simsii	29760.VIT_01s0026g02570.t01	[illegible]	[illegible]
endless_summer2_g51063	endless_summer2_g51063	Endless Summer genom	Alternative assembl	tr|A0A834ZWY8|A0A834ZWY8_9MAGN	52.6	tr|A0A834ZWY8|A0A834ZWY8_9MAGN Uncharacterized protein OS=Tetracentron sinense OX=13715 GN=HHK36_001056 P	tetracentron sinense	39947.LOC_Os05g32740.1	[illegible]	[illegible]
endless_summer2_g51188	endless_summer2_g51188	Endless Summer genom	Alternative assembl	tr|A0A4U1CWZ0|A0A4U1CWZ0_9BACI	30.1	tr|A0A4U1CWZ0|A0A4U1CWZ0_9BACI Ribonuclease HI family protein OS=Robertmurraya kyonggiensis OX=1037680 GN=	robertmurraya kyonggiensis	39947.LOC_Os07g13340.1	[illegible]	[illegible]
endless_summer2_g51287	endless_summer2_g51287	Endless Summer genom	Alternative assembl	XP_021684896.1	77.6	XP_021684896.1 adenine/guanine permease AZG1-like [Hevea brasiliensis]	hevea brasiliensis	4081.Solyc06g050250.1.1	[illegible]	[illegible]
endless_summer2_g51425	endless_summer2_g51425	Endless Summer genom	Alternative assembl	XP_028083832.1	69.5	XP_028083832.1 probable LRR receptor-like serine/threonine-protein kinase At3g47570 [Camellia sinensis]	camellia sinensis	3694.POPTR_0011s10430.1	[illegible]	[illegible]
endless_summer2_g51441	endless_summer2_g51441	Endless Summer genom	Alternative assembl	XP_028083844.1	54.2	XP_028083844.1 probable LRR receptor-like serine/threonine-protein kinase At3g47570 [Camellia sinensis]	camellia sinensis	3694.POPTR_1358s00200.1	[illegible]	[illegible]
endless_summer2_g51450	endless_summer2_g51450	Endless Summer genom	Alternative assembl	tr|A0A5B7BF39|A0A5B7BF39_DAVIN	76.3	tr|A0A5B7BF39|A0A5B7BF39_DAVIN Protein kinase domain-containing protein (Fragment) OS=Davidia involucrata OX=169	davidia involucrata	3694.POPTR_0018s02450.1	[illegible]	[illegible]
endless_summer2_g51456	endless_summer2_g51456	Endless Summer genom	Alternative assembl	XP_021296044.1	70.1	XP_021296044.1 LOW QUALITY PROTEIN: probable LRR receptor-like serine/threonine-protein kinase At3g47570 [Herrania	herrania umbratica	3694.POPTR_0018s02450.1	[illegible]	[illegible]
endless_summer2_g51517	endless_summer2_g51517	Endless Summer genom	Alternative assembl	tr|F4NCK7|F4NCK7_BETVV	30	tr|F4NCK7|F4NCK7_BETVV Reverse transcriptase domain-containing protein OS=Beta vulgaris subsp. vulgaris OX=3555 PE=	beta vulgaris subsp. vulgar	3694.POPTR_0019s01390.1	[illegible]	[illegible]
endless_summer2_g51653	endless_summer2_g51653	Endless Summer genom	Alternative assembl	XP_011465812.1	71	XP_011465812.1 PREDICTED: uncharacterized protein LOC101310615 [Fragaria vesca subsp. vesca]	fragaria vesca subsp. vesca	4577.GRMZM2G039911_P01	[illegible]	[illegible]
endless_summer2_g51661	endless_summer2_g51661	Endless Summer genom	Alternative assembl	XP_021804720.1	44.3	XP_021804720.1 uncharacterized protein LOC110749029 [Prunus avium]	prunus avium	4558.Sb03g023051.1	[illegible]	[illegible]
endless_summer2_g51662	endless_summer2_g51662	Endless Summer genom	Alternative assembl	tr|A0A834LDS2|A0A834LDS2_RHOSS	33.6	tr|A0A834LDS2|A0A834LDS2_RHOSS Uncharacterized protein OS=Rhododendron simsii OX=118357 GN=RHSIM_Rhsim10	rhododendron simsii		[illegible]	[illegible]
endless_summer2_g51718	endless_summer2_g51718	Endless Summer genom	Alternative assembl	XP_028078469.1	37.1	XP_028078469.1 uncharacterized protein LOC114280312 isoform X3 [Camellia sinensis]	camellia sinensis	3847.GLYMA05G29901.1	[illegible]	[illegible]
endless_summer2_g51808	endless_summer2_g51808	Endless Summer genom	Alternative assembl	tr|A0A803LXY4|A0A803LXY4_CHEQI	56.4	tr|A0A803LXY4|A0A803LXY4_CHEQI Reverse transcriptase Ty1/copia-type domain-containing protein OS=Chenopodium qui	chenopodium quinoa	39947.LOC_Os05g45550.1	[illegible]	[illegible]
endless_summer2_g5185	endless_summer2_g5185	Endless Summer genom	Alternative assembl	tr|A0A7J6WF95|A0A7J6WF95_THATH	36.6	tr|A0A7J6WF95|A0A7J6WF95_THATH ULP_PROTEASE domain-containing protein OS=Thalictrum thalictroides OX=4696	thalictrum thalictroides	29760.VIT_13s0019g01850.t01	[illegible]	[illegible]
endless_summer2_g51927	endless_summer2_g51927	Endless Summer genom	Alternative assembl	tr|A0A2N9HMQ6|A0A2N9HMQ6_FAGSY	35.8	tr|A0A2N9HMQ6|A0A2N9HMQ6_FAGSY Integrase catalytic domain-containing protein OS=Fagus sylvatica OX=28930 GN=F	fagus sylvatica	4577.GRMZM2G131990_P01	[illegible]	[illegible]
endless_summer2_g51991	endless_summer2_g51991	Endless Summer genom	Alternative assembl	XP_028123119.1	67.6	XP_028123119.1 pentatricopeptide repeat-containing protein At1g25360 [Camellia sinensis]	camellia sinensis	29760.VIT_18s0001g13940.t01	[illegible]	[illegible]
endless_summer2_g52006	endless_summer2_g52006	Endless Summer genom	Alternative assembl	tr|A0A834GSF8|A0A834GSF8_RHOSS	31.9	tr|A0A834GSF8|A0A834GSF8_RHOSS PMD domain-containing protein OS=Rhododendron simsii OX=118357 GN=RHSIM	rhododendron simsii	4555.Si039373m	[illegible]	[illegible]
endless_summer2_g52037	endless_summer2_g52037	Endless Summer genom	Alternative assembl	tr|A0A4R5JH87|A0A4R5JH87_9FIRM	38.2	tr|A0A4R5JH87|A0A4R5JH87_9FIRM Retroviral-like aspartic protease (Fragment) OS=Zhaonella formicivorans OX=2528593 C	zhaonella formicivorans	29760.VIT_17s0053g00270.t01	[illegible]	[illegible]
endless_summer2_g52163	endless_summer2_g52163	Endless Summer genom	Alternative assembl	XP_028100951.1	34.5	XP_028100951.1 uncharacterized protein LOC114300283, partial [Camellia sinensis]	camellia sinensis	4113.PGSC0003DMT400025159	[illegible]	[illegible]
endless_summer2_g52227	endless_summer2_g52227	Endless Summer genom	Alternative assembl	tr|A0A5B7BCG9|A0A5B7BCG9_DAVIN	66.7	tr|A0A5B7BCG9|A0A5B7BCG9_DAVIN Enhancer of polycomb-like protein OS=Davidia involucrata OX=16924 GN=Din_035	davidia involucrata	29760.VIT_13s0074g00710.t01	[illegible]	[illegible]
endless_summer2_g52236	endless_summer2_g52236	Endless Summer genom	Alternative assembl	XP_030442099.1	47	XP_030442099.1 uncharacterized protein LOC115664291 [Syzygium oleosum]	syzygium oleosum	29760.VIT_18s0117g00190.t01	[illegible]	[illegible]
endless_summer2_g52267	endless_summer2_g52267	Endless Summer genom	Alternative assembl	tr|A0A5B7ADP8|A0A5B7ADP8_DAVIN	45.8	tr|A0A5B7ADP8|A0A5B7ADP8_DAVIN CCHC-type domain-containing protein (Fragment) OS=Davidia involucrata OX=1692	davidia involucrata	3694.POPTR_0005s01170.1	[illegible]	[illegible]
endless_summer2_g52458	endless_summer2_g52458	Endless Summer genom	Alternative assembl	tr|A0A7J7G1E3|A0A7J7G1E3_CAMSI	35.3	tr|A0A7J7G1E3|A0A7J7G1E3_CAMSI Uncharacterized protein OS=Camellia sinensis OX=4442 GN=HYC85_029217 PE=4 SV	camellia sinensis		[illegible]	[illegible]
endless_summer2_g52499	endless_summer2_g52499	Endless Summer genom	Alternative assembl	XP_017970485.1	48.1	XP_017970485.1 PREDICTED: uncharacterized protein LOC108660722 [Theobroma cacao]	theobroma cacao	29760.VIT_07s0095g00580.t01	[illegible]	[illegible]
endless_summer2_g5257	endless_summer2_g5257	Endless Summer genom	Alternative assembl	tr|A0A699LGD6|A0A699LGD6_TANCI	55.4	tr|A0A699LGD6|A0A699LGD6_TANCI Reverse transcriptase domain-containing protein OS=Tanacetum cinerariifolium OX=11	tanacetum cinerariifolium	3694.POPTR_0025s00240.1	[illegible]	[illegible]
endless_summer2_g52703	endless_summer2_g52703	Endless Summer genom	Alternative assembl	tr|A0A5B7BIV0|A0A5B7BIV0_DAVIN	47.1	tr|A0A5B7BIV0|A0A5B7BIV0_DAVIN Uncharacterized protein (Fragment) OS=Davidia involucrata OX=16924 GN=Din_0378	davidia involucrata	29760.VIT_10s0116g00400.t01	[illegible]	[illegible]
endless_summer2_g52710	endless_summer2_g52710	Endless Summer genom	Alternative assembl	tr|A0A7J7G1X7|A0A7J7G1X7_CAMSI	48.6	tr|A0A7J7G1X7|A0A7J7G1X7_CAMSI Uncharacterized protein OS=Camellia sinensis OX=4442 GN=HYC85_030850 PE=4 S	camellia sinensis	39947.LOC_Os01g06700.1	[illegible]	[illegible]
endless_summer2_g52737	endless_summer2_g52737	Endless Summer genom	Alternative assembl	tr|A0A803M3U1|A0A803M3U1_CHEQI	47.9	tr|A0A803M3U1|A0A803M3U1_CHEQI CCHC-type domain-containing protein OS=Chenopodium quinoa OX=63459 PE=4 S	chenopodium quinoa	4081.Solyc04g025620.1.1	[illegible]	[illegible]
endless_summer2_g52790	endless_summer2_g52790	Endless Summer genom	Alternative assembl	XP_024191644.2	61.1	XP_024191644.2 uncharacterized protein LOC112195438 [Rosa chinensis]	rosa chinensis	4577.GRMZM2G137153_P01	[illegible]	[illegible]
endless_summer2_g52793	endless_summer2_g52793	Endless Summer genom	Alternative assembl	tr|A0A2N9J0D8|A0A2N9J0D8_FAGSY	36.8	tr|A0A2N9J0D8|A0A2N9J0D8_FAGSY zf-RVT domain-containing protein OS=Fagus sylvatica OX=28930 GN=FSB_LOCUS	fagus sylvatica	3694.POPTR_0011s01220.1	[illegible]	[illegible]
endless_summer2_g52817	endless_summer2_g52817	Endless Summer genom	Alternative assembl	tr|A0A4Q3ENA8|A0A4Q3ENA8_9SPHI	25.9	tr|A0A4Q3ENA8|A0A4Q3ENA8_9SPHI Reverse transcriptase-like protein (Fragment) OS=Sphingobacteriaceae bacterium OX=2	sphingobacteriaceae bacteri	4641.GSMUA_AchrUn_randomP11040_001	[illegible]	[illegible]
endless_summer2_g52826	endless_summer2_g52826	Endless Summer genom	Alternative assembl	XP_016490910.1	45.4	XP_016490910.1 PREDICTED: uncharacterized protein LOC107810631 [Nicotiana tabacum]	nicotiana tabacum	3847.GLYMA15G21480.1	[illegible]	[illegible]
endless_summer2_g52968	endless_summer2_g52968	Endless Summer genom	Alternative assembl	XP_028103119.1	66.3	XP_028103119.1 pentatricopeptide repeat-containing protein At2g20710, mitochondrial-like [Camellia sinensis]	camellia sinensis	3694.POPTR_0016s06320.1	[illegible]	[illegible]
endless_summer2_g53052	endless_summer2_g53052	Endless Summer genom	Alternative assembl	tr|A0A5B6YU87|A0A5B6YU87_DAVIN	41.7	tr|A0A5B6YU87|A0A5B6YU87_DAVIN Phorbol-ester/DAG-type domain-containing protein OS=Davidia involucrata OX=1692	davidia involucrata	3694.POPTR_0016s05820.1	[illegible]	[illegible]
endless_summer2_g53059	endless_summer2_g53059	Endless Summer genom	Alternative assembl	tr|A0A5B7C4P1|A0A5B7C4P1_DAVIN	41.8	tr|A0A5B7C4P1|A0A5B7C4P1_DAVIN Retrotrans_gag domain-containing protein OS=Davidia involucrata OX=16924 GN=Di	davidia involucrata	29760.VIT_00s0823g00010.t01	[illegible]	[illegible]
endless_summer2_g53060	endless_summer2_g53060	Endless Summer genom	Alternative assembl	tr|A0A5A7SMZ7|A0A5A7SMZ7_CUCME	34.1	tr|A0A5A7SMZ7|A0A5A7SMZ7_CUCME Gag-pro-like protein OS=Cucumis melo var. makuwa OX=1194695 GN=E5676_sca	cucumis melo var. makuwa	4113.PGSC0003DMT400091433	[illegible]	[illegible]
endless_summer2_g53139	endless_summer2_g53139	Endless Summer genom	Alternative assembl	XP_027072900.1	68.4	XP_027072900.1 pistil-specific extensin-like protein [Coffea arabica]	coffea arabica	3694.POPTR_0006s12070.1	[illegible]	[illegible]
endless_summer2_g53168	endless_summer2_g53168	Endless Summer genom	Alternative assembl	XP_028085646.1	27.1	XP_028085646.1 uncharacterized protein LOC114286664 [Camellia sinensis]	camellia sinensis		[illegible]	[illegible]
endless_summer2_g5317	endless_summer2_g5317	Endless Summer genom	Alternative assembl	tr|A0A834GYJ2|A0A834GYJ2_RHOSS	34.5	tr|A0A834GYJ2|A0A834GYJ2_RHOSS SWIM-type domain-containing protein OS=Rhododendron simsii OX=118357 GN=RI	rhododendron simsii	29760.VIT_14s0060g00370.t01	[illegible]	[illegible]
endless_summer2_g5318	endless_summer2_g5318	Endless Summer genom	Alternative assembl	tr|A0A5B7CCK8|A0A5B7CCK8_DAVIN	36.3	tr|A0A5B7CCK8|A0A5B7CCK8_DAVIN ULP_PROTEASE domain-containing protein (Fragment) OS=Davidia involucrata O	davidia involucrata		[illegible]	[illegible]
endless_summer2_g53182	endless_summer2_g53182	Endless Summer genom	Alternative assembl	tr|A0A7J0DK60|A0A7J0DK60_9ERIC	62.1	tr|A0A7J0DK60|A0A7J0DK60_9ERIC Retrotran_gag_3 domain-containing protein OS=Actinidia rufa OX=165716 GN=Acr_00	actinidia rufa	39946.BGIOSGA002386-PA	[illegible]	[illegible]
endless_summer2_g53304	endless_summer2_g53304	Endless Summer genom	Alternative assembl	tr|A0A6P9EA76|A0A6P9EA76_JUGRE	42.1	tr|A0A6P9EA76|A0A6P9EA76_JUGRE ATP-dependent DNA helicase OS=Juglans regia OX=51240 GN=LOC118344042 PE=	juglans regia	4558.Sb04g037770.1	[illegible]	[illegible]
endless_summer2_g53313	endless_summer2_g53313	Endless Summer genom	Alternative assembl	tr|A0A7J0DSQ5|A0A7J0DSQ5_9ERIC	36.8	tr|A0A7J0DSQ5|A0A7J0DSQ5_9ERIC CCHC-type domain-containing protein OS=Actinidia rufa OX=165716 GN=Acr_00s007	actinidia rufa	29760.VIT_18s0001g09780.t01	[illegible]	[illegible]
endless_summer2_g53351	endless_summer2_g53351	Endless Summer genom	Alternative assembl	tr|A0A834YN52|A0A834YN52_9MAGN	75.9	tr|A0A834YN52|A0A834YN52_9MAGN Uncharacterized protein OS=Tetracentron sinense OX=13715 GN=HHK36_024444 PE	tetracentron sinense	39947.LOC_Os09g08510.1	[illegible]	[illegible]
endless_summer2_g53364	endless_summer2_g53364	Endless Summer genom	Alternative assembl	tr|A0A7J7G0C5|A0A7J7G0C5_CAMSI	21.8	tr|A0A7J7G0C5|A0A7J7G0C5_CAMSI Uncharacterized protein OS=Camellia sinensis OX=4442 GN=HYC85_030304 PE=4 S	camellia sinensis		[illegible]	[illegible]
endless_summer2_g53396	endless_summer2_g53396	Endless Summer genom	Alternative assembl	tr|A0A4Y8W7B9|A0A4Y8W7B9_9VIBR	37.1	tr|A0A4Y8W7B9|A0A4Y8W7B9_9VIBR CCHC-type domain-containing protein (Fragment) OS=Vibrio ouci OX=2499078 GN	vibrio ouci	4081.Solyc04g025620.1.1	[illegible]	[illegible]
endless_summer2_g53531	endless_summer2_g53531	Endless Summer genom	Alternative assembl	XP_037496372.1	67.5	XP_037496372.1 uncharacterized protein LOC119371045 [Jatropha curcas]	jatropha curcas	4081.Solyc04g025620.1.1	[illegible]	[illegible]
endless_summer2_g53545	endless_summer2_g53545	Endless Summer genom	Alternative assembl	tr|A0A2N9ES54|A0A2N9ES54_FAGSY	48.8	tr|A0A2N9ES54|A0A2N9ES54_FAGSY Protein kinase domain-containing protein OS=Fagus sylvatica OX=28930 GN=FSB_L	fagus sylvatica	29760.VIT_13s0067g00490.t01	[illegible]	[illegible]
endless_summer2_g53572	endless_summer2_g53572	Endless Summer genom	Alternative assembl	XP_019079633.1	41.8	XP_019079633.1 PREDICTED: uncharacterized protein LOC109123680 [Vitis vinifera]	vitis vinifera	3847.GLYMA15G21480.1	[illegible]	[illegible]
endless_summer2_g53580	endless_summer2_g53580	Endless Summer genom	Alternative assembl	tr|A0A834G7V7|A0A834G7V7_RHOSS	67.1	tr|A0A834G7V7|A0A834G7V7_RHOSS RNase H type-1 domain-containing protein OS=Rhododendron simsii OX=118357 GN	rhododendron simsii	3694.POPTR_0011s01230.1	[illegible]	[illegible]
endless_summer2_g53784	endless_summer2_g53784	Endless Summer genom	Alternative assembl	XP_028077313.1	30.4	XP_028077313.1 uncharacterized protein LOC114279294 [Camellia sinensis]	camellia sinensis	29760.VIT_12s0055g00470.t01	[illegible]	[illegible]
endless_summer2_g53861	endless_summer2_g53861	Endless Summer genom	Alternative assembl	tr|A0A4Q3EUQ8|A0A4Q3EUQ8_9SPHI	37.2	tr|A0A4Q3EUQ8|A0A4Q3EUQ8_9SPHI Uncharacterized protein OS=Sphingobacteriaceae bacterium OX=2021370 GN=EOP45	sphingobacteriaceae bacteri	39947.LOC_Os04g33010.1	[illegible]	[illegible]
endless_summer2_g53870	endless_summer2_g53870	Endless Summer genom	Alternative assembl	tr|A0A5H2XRU3|A0A5H2XRU3_PRUDU	41	tr|A0A5H2XRU3|A0A5H2XRU3_PRUDU PMD domain-containing protein OS=Prunus dulcis OX=3755 GN=Prudu_945S0001	prunus dulcis	4113.PGSC0003DMT400035683	[illegible]	[illegible]
endless_summer2_g53946	endless_summer2_g53946	Endless Summer genom	Alternative assembl	tr|A0A6P6WYL5|A0A6P6WYL5_COFAR	44.3	tr|A0A6P6WYL5|A0A6P6WYL5_COFAR uncharacterized protein LOC113737393 OS=Coffea arabica OX=13443 GN=LOC113	coffea arabica	39947.LOC_Os09g08590.1	[illegible]	[illegible]
endless_summer2_g54101	endless_summer2_g54101	Endless Summer genom	Alternative assembl	tr|E6NUA4|E6NUA4_JATCU	43.7	tr|E6NUA4|E6NUA4_JATCU JHL23C09.13 protein OS=Jatropha curcas OX=180498 GN=JHL23C09.13 PE=4 SV=1	jatropha curcas	29760.VIT_00s0823g00010.t01	[illegible]	[illegible]
endless_summer2_g54252	endless_summer2_g54252	Endless Summer genom	Alternative assembl	XP_028085327.1	62.5	XP_028085327.1 E3 ubiquitin-protein ligase RMA3-like [Camellia sinensis]	camellia sinensis	29760.VIT_19s0014g02900.t01	[illegible]	[illegible]
endless_summer2_g54277	endless_summer2_g54277	Endless Summer genom	Alternative assembl	XP_023913708.1	43.7	XP_023913708.1 uncharacterized protein LOC112025290 [Quercus suber]	quercus suber	29760.VIT_04s0008g06010.t01	[illegible]	[illegible]
endless_summer2_g54310	endless_summer2_g54310	Endless Summer genom	Alternative assembl	tr|A0A4S4EG61|A0A4S4EG61_CAMSI	68.9	tr|A0A4S4EG61|A0A4S4EG61_CAMSI TCP domain-containing protein OS=Camellia sinensis var. sinensis OX=542762 GN=	camellia sinensis var. sinen	3694.POPTR_0001s38480.1	[illegible]	[illegible]
endless_summer2_g54651	endless_summer2_g54651	Endless Summer genom	Alternative assembl	XP_028073220.1	83.3	XP_028073220.1 serine/threonine protein phosphatase 2A 59 kDa regulatory subunit B' gamma isoform-like [Camellia sinensis]	camellia sinensis	29760.VIT_12s0059g01170.t01	[illegible]	[illegible]
endless_summer2_g54686	endless_summer2_g54686	Endless Summer genom	Alternative assembl	XP_022158689.1	32.3	XP_022158689.1 uncharacterized protein LOC111025150 [Momordica charantia]	momordica charantia	29760.VIT_15s0046g02360.t01	[illegible]	[illegible]
endless_summer2_g54977	endless_summer2_g54977	Endless Summer genom	Alternative assembl	tr|A0A2I4G585|A0A2I4G585_JUGRE	34.2	tr|A0A2I4G585|A0A2I4G585_JUGRE uncharacterized protein LOC109004818 OS=Juglans regia OX=51240 GN=LOC1090048	juglans regia	4558.Sb07g024435.1	[illegible]	[illegible]
endless_summer2_g54979	endless_summer2_g54979	Endless Summer genom	Alternative assembl	tr|A0A7J7FZ44|A0A7J7FZ44_CAMSI	23.3	tr|A0A7J7FZ44|A0A7J7FZ44_CAMSI ULP_PROTEASE domain-containing protein OS=Camellia sinensis OX=4442 GN=HY	camellia sinensis		[illegible]	[illegible]
endless_summer2_g55034	endless_summer2_g55034	Endless Summer genom	Alternative assembl	tr|A0A834CRN0|A0A834CRN0_JUGRE	39.3	tr|A0A834CRN0|A0A834CRN0_JUGRE Uncharacterized protein OS=Juglans regia OX=51240 GN=F2P56_013047 PE=4 SV=	juglans regia	4577.GRMZM2G089755_P01	[illegible]	[illegible]
endless_summer2_g55116	endless_summer2_g55116	Endless Summer genom	Alternative assembl	tr|A0A5J5A800|A0A5J5A800_9ASTE	51.1	tr|A0A5J5A800|A0A5J5A800_9ASTE Uncharacterized protein OS=Nyssa sinensis OX=561372 GN=F0562_008388 PE=3 SV=	nyssa sinensis	3694.POPTR_0013s13930.1	[illegible]	[illegible]
endless_summer2_g5517	endless_summer2_g5517	Endless Summer genom	Alternative assembl	tr|A0A834LE88|A0A834LE88_RHOSS	39.5	tr|A0A834LE88|A0A834LE88_RHOSS Integrase_H2C2 domain-containing protein OS=Rhododendron simsii OX=118357 GN=	rhododendron simsii	4577.GRMZM2G131990_P01	[illegible]	[illegible]
endless_summer2_g5518	endless_summer2_g5518	Endless Summer genom	Alternative assembl	tr|A0A834GF70|A0A834GF70_RHOSS	59.6	tr|A0A834GF70|A0A834GF70_RHOSS Uncharacterized protein OS=Rhododendron simsii OX=118357 GN=RHSIM_Rhsim10	rhododendron simsii	4577.GRMZM2G006169_P01	[illegible]	[illegible]
endless_summer2_g55245	endless_summer2_g55245	Endless Summer genom	Alternative assembl	tr|A0A834GVC8|A0A834GVC8_RHOSS	78.2	tr|A0A834GVC8|A0A834GVC8_RHOSS Prohibitin OS=Rhododendron simsii OX=118357 GN=RHSIM_Rhsim06G0136000	rhododendron simsii	3847.GLYMA09G30410.1	[illegible]	[illegible]
endless_summer2_g55261	endless_summer2_g55261	Endless Summer genom	Alternative assembl	XP_030941202.1	44.8	XP_030941202.1 uncharacterized protein LOC115966010 [Quercus lobata]	quercus lobata	3847.GLYMA04G17450.1	[illegible]	[illegible]
endless_summer2_g55273	endless_summer2_g55273	Endless Summer genom	Alternative assembl	XP_028087356.1	58.1	XP_028087356.1 uncharacterized protein LOC114288041 [Camellia sinensis]	camellia sinensis	4533.OB06G16940.1	[illegible]	[illegible]
endless_summer2_g55312	endless_summer2_g55312	Endless Summer genom	Alternative assembl	XP_028092203.1	39.5	XP_028092203.1 uncharacterized protein LOC114292429 [Camellia sinensis]	camellia sinensis	4081.Solyc07g053320.2.1	NA	[illegible]
endless_summer2_g55326	endless_summer2_g55326	Endless Summer genom	Alternative assembl	XP_022773772.1	77.1	XP_022773772.1 senescence-specific cysteine protease SAG39-like [Durio zibethinus]	durio zibethinus	29760.VIT_12s0028g02290.t01	[illegible]	[illegible]
endless_summer2_g55584	endless_summer2_g55584	Endless Summer genom	Alternative assembl	XP_028127050.1	66.4	XP_028127050.1 kinesin-like protein KIN-12F isoform X1 [Camellia sinensis]	camellia sinensis	29760.VIT_19s0090g01600.t01	[illegible]	[illegible]
endless_summer2_g55612	endless_summer2_g55612	Endless Summer genom	Alternative assembl	XP_035836999.1	41.6	XP_035836999.1 uncharacterized protein LOC110877269 [Helianthus annuus]	helianthus annuus	4081.Solyc03g096270.1.1	[illegible]	[illegible]
endless_summer2_g55651	endless_summer2_g55651	Endless Summer genom	Alternative assembl	XP_011097757.1	77.2	XP_011097757.1 probable L-type lectin-domain containing receptor kinase S.7 [Sesamum indicum]	sesamum indicum	29760.VIT_19s0015g00620.t01	[illegible]	[illegible]
endless_summer2_g55681	endless_summer2_g55681	Endless Summer genom	Alternative assembl	XP_034206412.1	45.3	XP_034206412.1 PKS-NRPS hybrid synthetase CHGG_01239-like [Prunus dulcis]	prunus dulcis	653948.CCA20024	[illegible]	[illegible]
endless_summer2_g55702	endless_summer2_g55702	Endless Summer genom	Alternative assembl	XP_024925570.1	90.3	XP_024925570.1 uridine kinase-like protein 3 [Ziziphus jujuba]	ziziphus jujuba	29760.VIT_19s0015g01730.t01	[illegible]	[illegible]
endless_summer2_g5582	endless_summer2_g5582	Endless Summer genom	Alternative assembl	XP_028067249.1	46.6	XP_028067249.1 uncharacterized protein LOC114270057 [Camellia sinensis]	camellia sinensis	3694.POPTR_0011s01230.1	[illegible]	[illegible]
endless_summer2_g55822	endless_summer2_g55822	Endless Summer genom	Alternative assembl	tr|A0A8C0UC45|A0A8C0UC45_CYACU	27.3	tr|A0A8C0UC45|A0A8C0UC45_CYACU Uncharacterized protein OS=Cyanistes caeruleus OX=156563 PE=4 SV=1	cyanistes caeruleus	3694.POPTR_0014s03680.1	[illegible]	[illegible]
endless_summer2_g55933	endless_summer2_g55933	Endless Summer genom	Alternative assembl	XP_009627394.1	57.9	XP_009627394.1 pectinesterase-like [Nicotiana tomentosiformis]	nicotiana tomentosiformis	4113.PGSC0003DMT400061821	[illegible]	[illegible]
endless_summer2_g55985	endless_summer2_g55985	Endless Summer genom	Alternative assembl	tr|A0A7J7FSX7|A0A7J7FSX7_CAMSI	66.7	tr|A0A7J7FSX7|A0A7J7FSX7_CAMSI Uncharacterized protein OS=Camellia sinensis OX=4442 GN=HYC85_032318 PE=4 S	camellia sinensis	29760.VIT_07s0005g01510.t01	NA	[illegible]
endless_summer2_g56031	endless_summer2_g56031	Endless Summer genom	Alternative assembl	tr|A0A453HZ91|A0A453HZ91_AEGTS	63.5	tr|A0A453HZ91|A0A453HZ91_AEGTS ATP-dependent DNA helicase OS=Aegilops tauschii subsp. strangulata OX=200361 PE	aegilops tauschii subsp. str	4555.Si019133m	[illegible]	[illegible]
endless_summer2_g56295	endless_summer2_g56295	Endless Summer genom	Alternative assembl	tr|A0A5B6YQ96|A0A5B6YQ96_DAVIN	48.9	tr|A0A5B6YQ96|A0A5B6YQ96_DAVIN PHD-type domain-containing protein OS=Davidia involucrata OX=16924 GN=Din_00	davidia involucrata	29760.VIT_07s0151g00870.t01	[illegible]	[illegible]
endless_summer2_g56301	endless_summer2_g56301	Endless Summer genom	Alternative assembl	tr|A0A834GEZ4|A0A834GEZ4_RHOSS	50	tr|A0A834GEZ4|A0A834GEZ4_RHOSS Uncharacterized protein OS=Rhododendron simsii OX=118357 GN=RHSIM_Rhsim10	rhododendron simsii	4533.OB12G18400.1	[illegible]	[illegible]
endless_summer2_g56387	endless_summer2_g56387	Endless Summer genom	Alternative assembl	tr|A0A2N9HHG6|A0A2N9HHG6_FAGSY	37.4	tr|A0A2N9HHG6|A0A2N9HHG6_FAGSY Uncharacterized protein OS=Fagus sylvatica OX=28930 GN=FSB_LOCUS41608 PE	fagus sylvatica		[illegible]	[illegible]
endless_summer2_g56411	endless_summer2_g56411	Endless Summer genom	Alternative assembl	tr|A0A438BYV4|A0A438BYV4_VITVI	61.6	tr|A0A438BYV4|A0A438BYV4_VITVI Retrotran_gag_3 domain-containing protein OS=Vitis vinifera OX=29760 GN=CK203	vitis vinifera	3847.GLYMA10G18381.1	[illegible]	[illegible]
endless_summer2_g56497	endless_summer2_g56497	Endless Summer genom	Alternative assembl	tr|A0A7J7HF94|A0A7J7HF94_CAMSI	42	tr|A0A7J7HF94|A0A7J7HF94_CAMSI Myb_DNA-bind_3 domain-containing protein OS=Camellia sinensis OX=4442 GN=HY	camellia sinensis	4113.PGSC0003DMT400077913	NA	[illegible]
endless_summer2_g5654	endless_summer2_g5654	Endless Summer genom	Alternative assembl	XP_028065895.1	58.8	XP_028065895.1 uncharacterized protein LOC114268864 [Camellia sinensis]	camellia sinensis	39947.LOC_Os08g36130.1	[illegible]	[illegible]
endless_summer2_g56778	endless_summer2_g56778	Endless Summer genom	Alternative assembl	tr|A0A6P6X856|A0A6P6X856_COFAR	51.4	tr|A0A6P6X856|A0A6P6X856_COFAR ATP-dependent DNA helicase OS=Coffea arabica OX=13443 GN=LOC113739044 PE=	coffea arabica	4081.Solyc03g083220.1.1	DNA helicase	[illegible]
endless_summer2_g56797	endless_summer2_g56797	Endless Summer genom	Alternative assembl	tr|A0A7J0FQP2|A0A7J0FQP2_9ERIC	58.5	tr|A0A7J0FQP2|A0A7J0FQP2_9ERIC CCHC-type domain-containing protein OS=Actinidia rufa OX=165716 GN=Acr_14g000	actinidia rufa	4081.Solyc03g096270.1.1	[illegible]	[illegible]
endless_summer2_g57096	endless_summer2_g57096	Endless Summer genom	Alternative assembl	tr|A0A3S3Q7E7|A0A3S3Q7E7_9MAGN	52.6	tr|A0A3S3Q7E7|A0A3S3Q7E7_9MAGN BED-type domain-containing protein OS=Cinnamomum micranthum f. kanehirae OX=	cinnamomum micranthum f.	3847.GLYMA13G26211.1	[illegible]	[illegible]
endless_summer2_g57197	endless_summer2_g57197	Endless Summer genom	Alternative assembl	XP_028073407.1	33.3	XP_028073407.1 uncharacterized protein LOC114275554 [Camellia sinensis]	camellia sinensis	3694.POPTR_0011s01230.1	[illegible]	[illegible]
endless_summer2_g57325	endless_summer2_g57325	Endless Summer genom	Alternative assembl	XP_024965021.1	87.3	XP_024965021.1 protein TIC 62, chloroplastic-like isoform X2 [Cynara cardunculus var. scolymus]	cynara cardunculus var. sco	59689.fgenesh2_kg.7__1017__AT4G31530.1	[illegible]	[illegible]
endless_summer2_g57346	endless_summer2_g57346	Endless Summer genom	Alternative assembl	tr|A0A2R6P8I4|A0A2R6P8I4_ACTCC	61.3	tr|A0A2R6P8I4|A0A2R6P8I4_ACTCC LRR receptor-like serine/threonine-protein kinase OS=Actinidia chinensis var. chinensis	actinidia chinensis var. chi	59689.fgenesh1_pm.C_scaffold_3000235	[illegible]	[illegible]
endless_summer2_g57425	endless_summer2_g57425	Endless Summer genom	Alternative assembl	XP_028054597.1	51.3	XP_028054597.1 uncharacterized protein LOC114258805 [Camellia sinensis]	camellia sinensis	15368.BRADI1G12267.1	[illegible]	[illegible]
endless_summer2_g5763	endless_summer2_g5763	Endless Summer genom	Alternative assembl	tr|A0A7J6VK22|A0A7J6VK22_THATH	36.5	tr|A0A7J6VK22|A0A7J6VK22_THATH Uncharacterized protein OS=Thalictrum thalictroides OX=46969 GN=FRX31_025295	thalictrum thalictroides		[illegible]	[illegible]
endless_summer2_g57673	endless_summer2_g57673	Endless Summer genom	Alternative assembl	XP_028064702.1	59.3	XP_028064702.1 uncharacterized protein LOC114267828 [Camellia sinensis]	camellia sinensis	39947.LOC_Os01g06700.1	[illegible]	[illegible]
endless_summer2_g57674	endless_summer2_g57674	Endless Summer genom	Alternative assembl	tr|A0A6C0FCH9|A0A6C0FCH9_9ZZZZ	41.3	tr|A0A6C0FCH9|A0A6C0FCH9_9ZZZZ RNase H type-1 domain-containing protein OS=viral metagenome OX=1070528 PE=4	viral metagenome	39947.LOC_Os07g13340.1	[illegible]	[illegible]
endless_summer2_g57787	endless_summer2_g57787	Endless Summer genom	Alternative assembl	tr|M1BTL6|M1BTL6_SOLTU	53.3	tr|M1BTL6|M1BTL6_SOLTU Ankyrin repeat-containing protein OS=Solanum tuberosum OX=4113 GN=102583755 PE=4 SV	solanum tuberosum	3694.POPTR_0007s00400.1	[illegible]	[illegible]
endless_summer2_g57847	endless_summer2_g57847	Endless Summer genom	Alternative assembl	XP_028122859.1	46.9	XP_028122859.1 vinorine synthase-like [Camellia sinensis]	camellia sinensis	3694.POPTR_0002s01180.1	[illegible]	[illegible]
endless_summer2_g57983	endless_summer2_g57983	Endless Summer genom	Alternative assembl	tr|A0A834H4L8|A0A834H4L8_RHOSS	28.5	tr|A0A834H4L8|A0A834H4L8_RHOSS PMD domain-containing protein OS=Rhododendron simsii OX=118357 GN=RHSIM	rhododendron simsii	3711.Bra023240.1-P	[illegible]	[illegible]
endless_summer2_g58032	endless_summer2_g58032	Endless Summer genom	Alternative assembl	tr|A0A3N7FBD9|A0A3N7FBD9_POPTR	34.2	tr|A0A3N7FBD9|A0A3N7FBD9_POPTR zf-RVT domain-containing protein OS=Populus trichocarpa OX=3694 GN=POPTR	populus trichocarpa	3694.POPTR_0064s00260.1	[illegible]	[illegible]
endless_summer2_g58104	endless_summer2_g58104	Endless Summer genom	Alternative assembl	tr|A5BU47|A5BU47_VITVI	39.6	tr|A5BU47|A5BU47_VITVI ULP_PROTEASE domain-containing protein OS=Vitis vinifera OX=29760 GN=VITISV_003642	vitis vinifera	3847.GLYMA15G21480.1	[illegible]	[illegible]
endless_summer2_g58239	endless_summer2_g58239	Endless Summer genom	Alternative assembl	tr|A0A2N9HMN3|A0A2N9HMN3_FAGSY	51.2	tr|A0A2N9HMN3|A0A2N9HMN3_FAGSY Uncharacterized protein OS=Fagus sylvatica OX=28930 GN=FSB_LOCUS41100 P	fagus sylvatica	3694.POPTR_0008s21790.1	[illegible]	[illegible]
endless_summer2_g58296	endless_summer2_g58296	Endless Summer genom	Alternative assembl	XP_028119483.1	62.8	XP_028119483.1 uncharacterized protein LOC114316972 [Camellia sinensis]	camellia sinensis	59689.Al_scaffold_0007_3409	NA	[illegible]
endless_summer2_g58419	endless_summer2_g58419	Endless Summer genom	Alternative assembl	XP_021816366.1	33.3	XP_021816366.1 uncharacterized protein LOC110758765 [Prunus avium]	prunus avium		[illegible]	[illegible]
endless_summer2_g58447	endless_summer2_g58447	Endless Summer genom	Alternative assembl	XP_028062998.1	68.3	XP_028062998.1 probable LRR receptor-like serine/threonine-protein kinase At3g47570 [Camellia sinensis]	camellia sinensis	29760.VIT_08s0007g02110.t01	[illegible]	[illegible]
endless_summer2_g58596	endless_summer2_g58596	Endless Summer genom	Alternative assembl	tr|A0A4Y1RAZ6|A0A4Y1RAZ6_PRUDU	61.5	tr|A0A4Y1RAZ6|A0A4Y1RAZ6_PRUDU Toll-Interleukin-Resistance domain family protein OS=Prunus dulcis OX=3755 GN=	prunus dulcis	3694.POPTR_0228s00200.1	[illegible]	[illegible]
endless_summer2_g58709	endless_summer2_g58709	Endless Summer genom	Alternative assembl	tr|A0A2N9ETJ6|A0A2N9ETJ6_FAGSY	57.3	tr|A0A2N9ETJ6|A0A2N9ETJ6_FAGSY RNA-directed DNA polymerase OS=Fagus sylvatica OX=28930 GN=FSB_LOCUS60	fagus sylvatica	39947.LOC_Os10g06550.1	[illegible]	[illegible]
endless_summer2_g58745	endless_summer2_g58745	Endless Summer genom	Alternative assembl	tr|A0A4R5JFK7|A0A4R5JFK7_9FIRM	41.9	tr|A0A4R5JFK7|A0A4R5JFK7_9FIRM Uncharacterized protein (Fragment) OS=Zhaonella formicivorans OX=2528593 GN=EY	zhaonella formicivorans	29760.VIT_18s0001g09780.t01	[illegible]	[illegible]
endless_summer2_g59007	endless_summer2_g59007	Endless Summer genom	Alternative assembl	XP_028125329.1	29.8	XP_028125329.1 uncharacterized protein LOC114322248 [Camellia sinensis]	camellia sinensis	3711.Bra013389.1-P	[illegible]	[illegible]
endless_summer2_g5901	endless_summer2_g5901	Endless Summer genom	Alternative assembl	XP_030950986.1	72.4	XP_030950986.1 uncharacterized protein LOC115974663 [Quercus lobata]	quercus lobata	4113.PGSC0003DMT400078376	NA	[illegible]
endless_summer2_g5902	endless_summer2_g5902	Endless Summer genom	Alternative assembl	XP_028060182.1	78.1	XP_028060182.1 sec-independent protein translocase protein TATC, chloroplastic [Camellia sinensis]	camellia sinensis	4113.PGSC0003DMT400078514	Part of the twin-arginine translocation (Tat) system that transports large fo	[illegible]
endless_summer2_g59033	endless_summer2_g59033	Endless Summer genom	Alternative assembl	tr|A0A5D3E3A0|A0A5D3E3A0_CUCME	46.4	tr|A0A5D3E3A0|A0A5D3E3A0_CUCME RNase H type-1 domain-containing protein OS=Cucumis melo var. makuwa OX=119	cucumis melo var. makuwa	3694.POPTR_0011s01230.1	[illegible]	[illegible]
endless_summer2_g5936	endless_summer2_g5936	Endless Summer genom	Alternative assembl	tr|A0A5B6YXH7|A0A5B6YXH7_DAVIN	59.4	tr|A0A5B6YXH7|A0A5B6YXH7_DAVIN Putative UDP-glycosyltransferase 83A1 (Fragment) OS=Davidia involucrata OX=169	davidia involucrata	29760.VIT_14s0068g00450.t01	[illegible]	[illegible]
endless_summer2_g6040	endless_summer2_g6040	Endless Summer genom	Alternative assembl	XP_028106919.1	75.7	XP_028106919.1 acyl-acyl carrier protein thioesterase ATL3, chloroplastic-like [Camellia sinensis]	camellia sinensis	29760.VIT_01s0011g03700.t01	[illegible]	[illegible]
endless_summer2_g6094	endless_summer2_g6094	Endless Summer genom	Alternative assembl	XP_028071339.1	58.8	XP_028071339.1 protein FLX-like 3 [Camellia sinensis]	camellia sinensis	29760.VIT_01s0011g03510.t01	[illegible]	[illegible]
endless_summer2_g6128	endless_summer2_g6128	Endless Summer genom	Alternative assembl	tr|A0A2N9EE40|A0A2N9EE40_FAGSY	40.3	tr|A0A2N9EE40|A0A2N9EE40_FAGSY Uncharacterized protein OS=Fagus sylvatica OX=28930 GN=FSB_LOCUS5139 PE=4	fagus sylvatica	4081.Solyc11g056600.1.1	[illegible]	[illegible]
endless_summer2_g6379	endless_summer2_g6379	Endless Summer genom	Alternative assembl	XP_047315935.1	34.9	XP_047315935.1 uncharacterized mitochondrial protein AtMg00810-like [Impatiens glandulifera]	impatiens glandulifera	39947.LOC_Os05g45550.1	[illegible]	[illegible]
endless_summer2_g6382	endless_summer2_g6382	Endless Summer genom	Alternative assembl	XP_023893323.1	57.7	XP_023893323.1 cytochrome P450 CYP749A22-like [Quercus suber]	quercus suber	3694.POPTR_0010s14900.1	[illegible]	[illegible]
endless_summer2_g6688	endless_summer2_g6688	Endless Summer genom	Alternative assembl	XP_028085680.1	79.1	XP_028085680.1 SNF1-related protein kinase regulatory subunit gamma-1-like isoform X1 [Camellia sinensis]	camellia sinensis	29760.VIT_01s0026g01740.t01	[illegible]	[illegible]
endless_summer2_g6781	endless_summer2_g6781	Endless Summer genom	Alternative assembl	XP_030957026.1	62.3	XP_030957026.1 leucine zipper protein 1 isoform X1 [Quercus lobata]	quercus lobata	3694.POPTR_0008s19490.1	NA	[illegible]
endless_summer2_g7305	endless_summer2_g7305	Endless Summer genom	Alternative assembl	tr|A0A5B7BM92|A0A5B7BM92_DAVIN	54.3	tr|A0A5B7BM92|A0A5B7BM92_DAVIN Uncharacterized protein OS=Davidia involucrata OX=16924 GN=Din_037674 PE=3 S	davidia involucrata	3694.POPTR_0005s25820.1	[illegible]	[illegible]
endless_summer2_g740	endless_summer2_g740	Endless Summer genom	Alternative assembl	XP_022889192.1	33.5	XP_022889192.1 uncharacterized protein LOC111404645 [Olea europaea var. sylvestris]	olea europaea var. sylvestri	4081.Solyc03g096270.1.1	[illegible]	[illegible]
endless_summer2_g7485	endless_summer2_g7485	Endless Summer genom	Alternative assembl	XP_019079556.1	32.6	XP_019079556.1 PREDICTED: uncharacterized protein LOC100241310 isoform X4 [Vitis vinifera]	vitis vinifera	29760.VIT_13s0019g01850.t01	[illegible]	[illegible]
endless_summer2_g7604	endless_summer2_g7604	Endless Summer genom	Alternative assembl	tr|A0A5B7APE0|A0A5B7APE0_DAVIN	39.3	tr|A0A5B7APE0|A0A5B7APE0_DAVIN Myb_DNA-bind_3 domain-containing protein (Fragment) OS=Davidia involucrata OX	davidia involucrata	3847.GLYMA03G06712.1	NA	[illegible]
endless_summer2_g7814	endless_summer2_g7814	Endless Summer genom	Alternative assembl	XP_020421265.1	49.7	XP_020421265.1 uncharacterized protein LOC109949602 [Prunus persica]	prunus persica	39947.LOC_Os06g47180.1	[illegible]	[illegible]
endless_summer2_g810	endless_summer2_g810	Endless Summer genom	Alternative assembl	tr|A0A6A4LMR4|A0A6A4LMR4_9ERIC	65	tr|A0A6A4LMR4|A0A6A4LMR4_9ERIC Clp_R domain-containing protein (Fragment) OS=Rhododendron williamsianum OX=	rhododendron williamsiana	3847.GLYMA04G06610.1	[illegible]	[illegible]
endless_summer2_g8136	endless_summer2_g8136	Endless Summer genom	Alternative assembl	tr|A0A6A6LJJ4|A0A6A6LJJ4_HEVBR	27.8	tr|A0A6A6LJJ4|A0A6A6LJJ4_HEVBR DDE_Tnp4 domain-containing protein OS=Hevea brasiliensis OX=3981 GN=GH714_00	hevea brasiliensis	4081.Solyc09g015810.1.1	[illegible]	[illegible]
endless_summer2_g8209	endless_summer2_g8209	Endless Summer genom	Alternative assembl	tr|A0A2N9HBD5|A0A2N9HBD5_FAGSY	37.1	tr|A0A2N9HBD5|A0A2N9HBD5_FAGSY ATP-dependent DNA helicase OS=Fagus sylvatica OX=28930 GN=FSB_LOCUS36	fagus sylvatica	4555.Si012162m	[illegible]	[illegible]
endless_summer2_g8259	endless_summer2_g8259	Endless Summer genom	Alternative assembl	tr|A0A3S3Q7R5|A0A3S3Q7R5_9MAGN	57.8	tr|A0A3S3Q7R5|A0A3S3Q7R5_9MAGN BED-type domain-containing protein OS=Cinnamomum micranthum f. kanehirae OX=	cinnamomum micranthum f.	3847.GLYMA03G13213.1	[illegible]	[illegible]
endless_summer2_g8280	endless_summer2_g8280	Endless Summer genom	Alternative assembl	tr|A0A5E4FGS5|A0A5E4FGS5_PRUDU	41.3	tr|A0A5E4FGS5|A0A5E4FGS5_PRUDU PREDICTED: rve domain-containing (Fragment) OS=Prunus dulcis OX=3755 GN=A	prunus dulcis	39947.LOC_Os04g07670.1	[illegible]	[illegible]
endless_summer2_g8287	endless_summer2_g8287	Endless Summer genom	Alternative assembl	tr|A0A6A6KLC6|A0A6A6KLC6_HEVBR	51	tr|A0A6A6KLC6|A0A6A6KLC6_HEVBR DUF4216 domain-containing protein OS=Hevea brasiliensis OX=3981 GN=GH714	hevea brasiliensis	39947.LOC_Os08g40360.1	[illegible]	[illegible]
endless_summer2_g8343	endless_summer2_g8343	Endless Summer genom	Alternative assembl	XP_018505659.1	45.1	XP_018505659.1 PREDICTED: uncharacterized protein LOC108868022 [Pyrus x bretschneideri]	pyrus x bretschneideri	39947.LOC_Os07g39230.1	[illegible]	[illegible]
endless_summer2_g840	endless_summer2_g840	Endless Summer genom	Alternative assembl	XP_039166850.1	35.7	XP_039166850.1 uncharacterized protein LOC108958275 [Eucalyptus grandis]	eucalyptus grandis	39947.LOC_Os10g37170.1	[illegible]	[illegible]
endless_summer2_g865	endless_summer2_g865	Endless Summer genom	Alternative assembl	tr|A0A7J0E1Z2|A0A7J0E1Z2_9ERIC	32.7	tr|A0A7J0E1Z2|A0A7J0E1Z2_9ERIC CCHC-type domain-containing protein OS=Actinidia rufa OX=165716 GN=Acr_00s0099	actinidia rufa	29760.VIT_18s0001g09780.t01	[illegible]	[illegible]
endless_summer2_g8674	endless_summer2_g8674	Endless Summer genom	Alternative assembl	tr|A0A438IZS4|A0A438IZS4_VITVI	40.2	tr|A0A438IZS4|A0A438IZS4_VITVI Transpos_assoc domain-containing protein OS=Vitis vinifera OX=29760 GN=CK203_025	vitis vinifera	4558.Sb05g021440.1	[illegible]	[illegible]
endless_summer2_g8785	endless_summer2_g8785	Endless Summer genom	Alternative assembl	tr|A0A438GCV2|A0A438GCV2_VITVI	59.6	tr|A0A438GCV2|A0A438GCV2_VITVI Retrotrans_gag domain-containing protein OS=Vitis vinifera OX=29760 GN=CK203_0	vitis vinifera	29760.VIT_18s0117g00190.t01	[illegible]	[illegible]
endless_summer2_g8840	endless_summer2_g8840	Endless Summer genom	Alternative assembl	XP_028074940.1	81.5	XP_028074940.1 proteasome assembly chaperone 3 isoform X2 [Camellia sinensis]	camellia sinensis	3694.POPTR_0001s34890.1	[illegible]	[illegible]
endless_summer2_g8863	endless_summer2_g8863	Endless Summer genom	Alternative assembl	XP_012851118.1	58.8	XP_012851118.1 PREDICTED: uncharacterized protein LOC105970840 [Erythranthe guttata]	erythranthe guttata	4577.GRMZM2G004107_P01	[illegible]	[illegible]

endless_summer2_g8929	endless_summer2_g8929	Endless Summer genom	Alternative assembl	tr|A0A2N9EYS5|A0A2N9EYS5_FAGSY	33.4	tr|A0A2N9EYS5|A0A2N9EYS5_FAGSY RNA-directed DNA polymerase OS=Fagus sylvatica OX=28930 GN=FSB_LOCUS7	*fagus sylvatica*	39947.LOC_Os08g26650.1				
endless_summer2_g8955	endless_summer2_g8955	Endless Summer genom	Alternative assembl	tr|A0A5J5BRN8|A0A5J5BRN8_9ASTE	60	tr|A0A5J5BRN8|A0A5J5BRN8_9ASTE Uncharacterized protein OS=Nyssa sinensis OX=561372 GN=F0562_019566 PE=4 SV	*nyssa sinensis*	3847.GLYMA08G03760.1	Ceramide glucosyltransferase	GO:0008150-biological_process(L=0),GO:0008152-metabolic proce		GO:0003674-molecular_function(L=0),GO:0003824-catalytic activity(L=1),GO:0016740-transferase activity(L=2),GO:0016757-transferase activity, transferring glycosyl groups(L=3),
endless_summer2_g9060	endless_summer2_g9060	Endless Summer genom	Alternative assembl	XP_019226297.1	30.5	XP_019226297.1 PREDICTED: uncharacterized protein LOC109207766 [Nicotiana attenuata]	*nicotiana attenuata*					
endless_summer2_g9066	endless_summer2_g9066	Endless Summer genom	Alternative assembl	tr|A0A5N5FYB2|A0A5N5FYB2_9ROSA	30	tr|A0A5N5FYB2|A0A5N5FYB2_9ROSA PMD domain-containing protein OS=Pyrus ussuriensis x Pyrus communis OX=2448	*pyrus ussuriensis x pyrus co*	4081.Solyc00g020540.1.1	Plant mobile domain			
endless_summer2_g9081	endless_summer2_g9081	Endless Summer genom	Alternative assembl	tr|A0A7J7FUI4|A0A7J7FUI4_CAMSI	34.7	tr|A0A7J7FUI4|A0A7J7FUI4_CAMSI Uncharacterized protein OS=Camellia sinensis OX=4442 GN=HYC85_028214 PE=4 SV	*camellia sinensis*					
endless_summer2_g9092	endless_summer2_g9092	Endless Summer genom	Alternative assembl	XP_042415332.1	63	XP_042415332.1 zinc finger MYM-type protein 1-like [Zingiber officinale]	*zingiber officinale*	3694.POPTR_0004s03580.1	protein kinase, interferon-inducible double stranded RNA dependent inhibitor, repressor of (P58 repressor)			GO:0003674-molecular_function(L=0),GO:0003676-nucleic acid binding(L=3),GO:0005488-binding(L=1),GO:0005549-odorant binding(L=2),GO:0097159-organic cyclic compound binding(L=2),GO:1901363-heterocyclic compound binding(L=2),
endless_summer2_g9100	endless_summer2_g9100	Endless Summer genom	Alternative assembl	XP_028074367.1	66.7	XP_028074367.1 uncharacterized protein LOC114276756 [Camellia sinensis]	*camellia sinensis*	39947.LOC_Os07g13340.1	Inherit from KOG: Retrotransposon protein			
endless_summer2_g912	endless_summer2_g912	Endless Summer genom	Alternative assembl	tr|A0A2N9EFA1|A0A2N9EFA1_FAGSY	58.6	tr|A0A2N9EFA1|A0A2N9EFA1_FAGSY Integrase catalytic domain-containing protein OS=Fagus sylvatica OX=28930 GN=FS	*fagus sylvatica*	39947.LOC_Os08g22660.1				
endless_summer2_g9128	endless_summer2_g9128	Endless Summer genom	Alternative assembl	tr|A0A834HPQ4|A0A834HPQ4_RHOSS	54	tr|A0A834HPQ4|A0A834HPQ4_RHOSS RNase H type-1 domain-containing protein OS=Rhododendron simsii OX=118357 GN	*rhododendron simsii*	39947.LOC_Os01g06700.1	Retrotransposon protein	GO:0006139-nucleobase-containing compound metabolic process(L-		GO:0003674-molecular_function(L=0),GO:0003676-nucleic acid binding(L=3),GO:0003723-RNA binding(L=4),GO:0003824-catalytic activity(L=1),GO:0003964-RNA-directed DNA polymerase activity(L=6),GO:0004518-nuclease activity(L=4),GO:0004519-endonuclease activity(L=5),GO:0004521-endoribonuclease activity(L=6),GO:0004523-RNA-DNA hybrid ribonuclease activity(L=8),GO:0004540-ribonuclease activity(L=5),GO:0005488-binding(L=1),GO:0016740-transferase activity(L=2),GO:0016772-transferase activity, transferring phosphorus-containing groups(L=3),GO:0016779-nucleotidyltransferase activity(L=4),GO:0016787-hydrolase activity(L=2),GO:0016788-hydrolase activity, acting on ester bonds(L=3),GO:0016891-endoribonuclease activity, producing 5'-phosphomonoesters(L=7),GO:0016893-endonuclease activity, active with either ri
endless_summer2_g9140	endless_summer2_g9140	Endless Summer genom	Alternative assembl	tr|A0A4U1CWG0|A0A4U1CWG0_9BACI	27.8	tr|A0A4U1CWG0|A0A4U1CWG0_9BACI Reverse transcriptase domain-containing protein OS=Robertmurraya kyonggiensis O	*robertmurraya kyonggiensis*	4577.GRMZM2G568664_P01				
endless_summer2_g9197	endless_summer2_g9197	Endless Summer genom	Alternative assembl	tr|A0A6A6N1Y2|A0A6A6N1Y2_HEVBR	70.7	tr|A0A6A6N1Y2|A0A6A6N1Y2_HEVBR GST N-terminal domain-containing protein OS=Hevea brasiliensis OX=3981 GN=GE	*hevea brasiliensis*	29760.VIT_15s0024g01540.t01	glutathione	GO:0008150-biological_process(L=0),GO:0008152-metabolic proce		GO:0003674-molecular_function(L=0),GO:0003824-catalytic activity(L=1),GO:0016740-transferase activity(L=2),
endless_summer2_g9657	endless_summer2_g9657	Endless Summer genom	Alternative assembl	XP_018835366.1	54	XP_018835366.1 subtilisin-like protease SBT3.18 isoform X2 [Juglans regia]	*juglans regia*	29760.VIT_06s0004g00210.t01	Subtilase family	GO:0000003-reproduction(L=1	GO:0005575-cellular_component(l	GO:0003674-molecular_function(L=0),GO:0003824-catalytic activity(L=1),GO:0004175-endopeptidase activity(L=5),GO:0004252-serine-type endopeptidase activity(L=5),GO:0008233-peptidase activity(L=3),GO:0008236-serine-type peptidase activity(L=4),GO:0016787-hydrolase activity(L=2),GO:0017171-serine hydrolase activity(L=3),GO:0070011-peptidase activity, acting on L-amino acid peptides(L=4),
endless_summer2_g9696	endless_summer2_g9696	Endless Summer genom	Alternative assembl	tr|A0A7J8TIE3|A0A7J8TIE3_GOSDV	77.1	tr|A0A7J8TIE3|A0A7J8TIE3_GOSDV Uncharacterized protein (Fragment) OS=Gossypium davidsonii OX=34287 GN=Godav_0	*gossypium davidsonii*	3847.GLYMA08G18760.1	RuBisCO large subunit-binding protein subunit beta	GO:0002376-immune system p	GO:0005575-cellular_component(l	GO:0000166-nucleotide binding(L=3),GO:0001882-nucleoside binding(L=3),GO:0001883-purine nucleoside binding(L=4),GO:0003674-molecular_function(L=0),GO:0005488-binding(L=1),GO:0005524-ATP binding(L=7),GO:0017076-purine nucleotide binding(L=4),GO:0030554-adenyl nucleotide binding(L=5),GO:0032549-ribonucleoside binding(L=4),GO:0032550-purine ribonucleoside binding(L=5),GO:0032553-ribonucleotide binding(L=3),GO:0032555-purine ribonucleotide binding(L=4),GO:0032559-adenyl ribonucleotide binding(L=6),GO:0035639-purine ribonucleoside triphosphate binding(L=4),GO:0036094-small molecule binding(L=2),GO:0043167-ion binding(L=2),GO:0043168-anion binding(L=3),GO:0097159-organic cyclic compound binding(L=2),GO:1901265-nucleoside phosphate binding(L=3),GO:1901363-heterocyclic compound
endless_summer2_g9702	endless_summer2_g9702	Endless Summer genom	Alternative assembl	XP_028107371.1	74.8	XP_028107371.1 fatty acyl-CoA reductase 2 [Camellia sinensis]	*camellia sinensis*	29760.VIT_08s0007g07100.t01	fatty acyl-CoA reductase	GO:0006629-lipid metabolic process(L=3),GO:0007275-multicellul		GO:0000166-nucleotide binding(L=3),GO:0003674-molecular_function(L=0),GO:0003824-catalytic activity(L=1),GO:0005488-binding(L=1),GO:0016491-oxidoreductase activity(L=2),GO:0016620-oxidoreductase activity, acting on the aldehyde or oxo group of donors, NAD or NADP as acceptor(L=4),GO:0016627-oxidoreductase activity, acting on the CH-CH group of donors(L=3),GO:0016628-oxidoreductase activity, acting on the CH-CH group of donors, NAD or NADP as acceptor(L=4),GO:0016903-oxidoreductase activity, acting on the aldehyde or oxo group of donors(L=3),GO:0036094-small molecule binding(L=2),GO:0080019-fatty-acyl-CoA reductase (alcohol-forming) activity(L=5),GO:0097159-organic cyclic compound binding(L=2),GO:1901265-nucleoside phosphate binding(L=3),GO:1901363-heterocyclic compound binding(L=2),
endless_summer2_g9713	endless_summer2_g9713	Endless Summer genom	Alternative assembl	XP_021902527.1	90.5	XP_021902527.1 tubulin beta-1 chain-like, partial [Carica papaya]	*carica papaya*	29760.VIT_18s0001g14360.t01	Tubulin is the major constituent of microtubules. It binds two moles of GTP, one at an exchangeable site on the	GO:0000902-cell morphogenesi	GO:0005575-cellular_component(l	GO:0000166-nucleotide binding(L=3),GO:0001882-nucleoside binding(L=3),GO:0001883-purine nucleoside binding(L=4),GO:0003674-molecular_function(L=0),GO:0003824-catalytic activity(L=1),GO:0003924-GTPase activity(L=7),GO:0005198-structural molecule activity(L=1),GO:0005200-structural constituent of cytoskeleton(L=2),GO:0005488-binding(L=1),GO:0005525-GTP binding(L=7),GO:0016462-pyrophosphatase activity(L=5),GO:0016787-hydrolase activity(L=2),GO:0016817-hydrolase activity, acting on acid anhydrides(L=3),GO:0016818-hydrolase activity, acting on acid anhydrides, in phosphorus-containing anhydrides(L=4),GO:0017076-purine nucleotide binding(L=4),GO:0017111-nucleoside-triphosphatase activity(L=6),GO:0019001-guanyl nucleotide binding(L=5),GO:0032549-ribonucleoside binding(L=4),GO:0032550-purine rib
endless_summer2_g9715	endless_summer2_g9715	Endless Summer genom	Alternative assembl	XP_021665686.1	35.5	XP_021665686.1 uncharacterized protein LOC110654108 [Hevea brasiliensis]	*hevea brasiliensis*	29760.VIT_00s0823g00010.t01	Retrotransposon gag protein	GO:0006139-nucleobase-contai	GO:0005575-cellular_component(l	GO:0003674-molecular_function(L=0),GO:0003676-nucleic acid binding(L=3),GO:0003677-DNA binding(L=4),GO:0003723-RNA binding(L=4),GO:0003824-catalytic activity(L=1),GO:0003964-RNA-directed DNA polymerase activity(L=6),GO:0004518-nuclease activity(L=4),GO:0004519-endonuclease activity(L=5),GO:0004521-endoribonuclease activity(L=6),GO:0004523-RNA-DNA hybrid ribonuclease activity(L=8),GO:0004540-ribonuclease activity(L=5),GO:0005488-binding(L=1),GO:0016740-transferase activity(L=2),GO:0016772-transferase activity, transferring phosphorus-containing groups(L=3),GO:0016779-nucleotidyltransferase activity(L=4),GO:0016787-hydrolase activity(L=2),GO:0016788-hydrolase activity, acting on ester bonds(L=3),GO:0016891-endoribonuclease activity, producing 5'-phosphomonoesters(L=7),GO:0016893-endonuc
endless_summer2_g9762	endless_summer2_g9762	Endless Summer genom	Alternative assembl	tr|A0A5J4ZJL6|A0A5J4ZJL6_9ASTE	49.1	tr|A0A5J4ZJL6|A0A5J4ZJL6_9ASTE HSA domain-containing protein OS=Nyssa sinensis OX=561372 GN=F0562_016401 PE	*nyssa sinensis*	15368.BRADI2G42337.1	HSA	GO:0008150-biological_process(L=0),GO:0008152-metabolic proce		GO:0003674-molecular_function(L=0),GO:0003824-catalytic activity(L=1),GO:0016787-hydrolase activity(L=2),GO:0016817-hydrolase activity, acting on acid anhydrides(L=3),
endless_summer2_g9799	endless_summer2_g9799	Endless Summer genom	Alternative assembl	tr|A0A834GWY7|A0A834GWY7_RHOSS	42.1	tr|A0A834GWY7|A0A834GWY7_RHOSS Uncharacterized protein OS=Rhododendron simsii OX=118357 GN=RHSIM_Rhsim	*rhododendron simsii*	4113.PGSC0003DMT400049107	CACTA, En/Spm sub-class			
endless_summer2_g9898	endless_summer2_g9898	Endless Summer genom	Alternative assembl	tr|F4NCM4|F4NCM4_BETVV	37.9	tr|F4NCM4|F4NCM4_BETVV Reverse transcriptase domain-containing protein OS=Beta vulgaris subsp. vulgaris OX=3555 PE	*beta vulgaris subsp. vulgari*	3694.POPTR_0019s01390.1	Retrotransposon protein	GO:0006082-organic acid metal	GO:0000145-exocyst(L=5),GO:00	GO:0000166-nucleotide binding(L=3),GO:0001882-nucleoside binding(L=3),GO:0001883-purine nucleoside binding(L=4),GO:0003674-molecular_function(L=0),GO:0003676-nucleic acid binding(L=3),GO:0003677-DNA binding(L=4),GO:0003723-RNA binding(L=4),GO:0003824-catalytic activity(L=1),GO:0003964-RNA-directed DNA polymerase activity(L=6),GO:0004518-nuclease activity(L=4),GO:0004519-endonuclease activity(L=5),GO:0004521-endoribonuclease activity(L=6),GO:0004523-RNA-DNA hybrid ribonuclease activity(L=8),GO:0004527-exonuclease activity(L=5),GO:0004540-ribonuclease activity(L=5),GO:0004672-protein kinase activity(L=5),GO:0004674-protein serine/threonine kinase activity(L=6),GO:0004812-aminoacyl-tRNA ligase activity(L=5),GO:0004820-glycine-tRNA ligase activity(L=6),GO:0005198-structural molecul
endless_summer2_g9915	endless_summer2_g9915	Endless Summer genom	Alternative assembl	tr|A0A7C8ZEB5|A0A7C8ZEB5_OPUST	53.8	tr|A0A7C8ZEB5|A0A7C8ZEB5_OPUST Retrotran_gag_3 domain-containing protein OS=Opuntia streptacantha OX=393608 PI	*opuntia streptacantha*					
endless_summer2_g9949	endless_summer2_g9949	Endless Summer genom	Alternative assembl	tr|A0A5B6Z5V5|A0A5B6Z5V5_DAVIN	52.2	tr|A0A5B6Z5V5|A0A5B6Z5V5_DAVIN ABC-type xenobiotic transporter OS=Davidia involucrata OX=16924 GN=Din_007834	*davidia involucrata*	3694.POPTR_0010s06540.1	ABC transporter	GO:0006139-nucleobase-contai	GO:0005575-cellular_component(l	GO:0000166-nucleotide binding(L=3),GO:0001882-nucleoside binding(L=3),GO:0001883-purine nucleoside binding(L=4),GO:0003674-molecular_function(L=0),GO:0003824-catalytic activity(L=1),GO:0005215-transporter activity(L=1),GO:0005488-binding(L=1),GO:0005524-ATP binding(L=7),GO:0015399-primary active transmembrane transporter activity(L=4),GO:0015405-P-P-bond-hydrolysis-driven transmembrane transporter activity(L=5),GO:0016462-pyrophosphatase activity(L=5),GO:0016787-hydrolase activity(L=2),GO:0016817-hydrolase activity, acting on acid anhydrides(L=3),GO:0016818-hydrolase activity, acting on acid anhydrides, in phosphorus-containing anhydrides(L=4),GO:0016820-hydrolase activity, acting on acid anhydrides, catalyzing transmembrane movement of substances(L=4),GO:0016887-ATPase activity(L=7),GO:001

nding(L=4),GO:0017111-nucleoside-triphosphatase activity(L=6),GO:0030554-adenyl nucleotide binding(L=5),GO:0032549-ribonucleoside binding(L=4),GO:0032550-purine ribonucleoside binding(L=5),GO:0032553-ribonucleotide binding(L=3),GO:0032

ribonucleotide binding(L=6),GO:0035639-purine ribonucleoside triphosphate binding(L=4),GO:0036094-small molecule binding(L=2),GO:0043167-ion binding(L=2),GO:0043168-anion binding(L=3),GO:0043531-ADP binding(L=4),GO:0097159-organic

e binding(L=4),GO:0032561-guanyl ribonucleotide binding(L=6),GO:0035639-purine ribonucleoside triphosphate binding(L=4),GO:0036094-small molecule binding(L=2),GO:0043167-ion binding(L=2),GO:0043168-anion binding(L=3),GO:0097159-org

488-binding(L=1),GO:0005506-iron ion binding(L=6),GO:0005507-copper ion binding(L=6),GO:0005524-ATP binding(L=7),GO:0008233-peptidase activity(L=3),GO:0008234-cysteine-type peptidase activity(L=5),GO:0008235-metalloexopeptidase activi

ribonucleotide binding(L=6),GO:0035639-purine ribonucleoside triphosphate binding(L=4),GO:0036094-small molecule binding(L=2),GO:0043167-ion binding(L=2),GO:0043168-anion binding(L=3),GO:0097159-organic cyclic compound binding(L=2

L=4),GO:0032559-adenyl ribonucleotide binding(L=6),GO:0035639-purine ribonucleoside triphosphate binding(L=4),GO:0036094-small molecule binding(L=2),GO:0043167-ion binding(L=2),GO:0043168-anion binding(L=3),GO:0043169-cation binding(L

ribonucleotide binding(L=6),GO:0035639-purine ribonucleoside triphosphate binding(L=4),GO:0036094-small molecule binding(L=2),GO:0043167-ion binding(L=2),GO:0043168-anion binding(L=3),GO:0043531-ADP binding(L=4),GO:0097159-organic

,GO:0032559-adenyl ribonucleotide binding(L=6),GO:0035639-purine ribonucleoside triphosphate binding(L=4),GO:0036094-small molecule binding(L=2),GO:0043167-ion binding(L=2),GO:0043168-anion binding(L=3),GO:0097159-organic cyclic compou

a acceptor(L=4),GO:0016829-lyase activity(L=2),GO:0016835-carbon-oxygen lyase activity(L=3),GO:0016836-hydro-lyase activity(L=4),GO:0016853-isomerase activity(L=2),GO:0016854-racemase and epimerase activity(L=3),GO:0016856-racemase and epimera

),GO:0032555-purine ribonucleotide binding(L=4),GO:0032559-adenyl ribonucleotide binding(L=6),GO:0035639-purine ribonucleoside triphosphate binding(L=4),GO:0036094-small molecule binding(L=2),GO:0043167-ion binding(L=2),GO:0043168-anion b

le activity(L=1),GO:0005488-binding(L=1),GO:0005524-ATP binding(L=7),GO:0008270-zinc ion binding(L=6),GO:0016301-kinase activity(L=4),GO:0016740-transferase activity(L=2),GO:0016772-transferase activity, transferring phosphorus-containin

,GO:0032549-ribonucleoside binding(L=4),GO:0032550-purine ribonucleoside binding(L=5),GO:0032553-ribonucleotide binding(L=3),GO:0032555-purine ribonucleotide binding(L=4),GO:0032559-adenyl ribonucleotide binding(L=6),GO:0035639-purine

)034061-DNA polymerase activity(L=5),GO:0036094-small molecule binding(L=2),GO:0043167-ion binding(L=2),GO:0043168-anion binding(L=3),GO:0043531-ADP binding(L=4),GO:0097159-organic cyclic compound binding(L=2),GO:1901265-nucleoside

le activity(L=1),GO:0005488-binding(L=1),GO:0005524-ATP binding(L=7),GO:0008270-zinc ion binding(L=6),GO:0016301-kinase activity(L=4),GO:0016740-transferase activity(L=2),GO:0016772-transferase activity, transferring phosphorus-containin

035639-purine ribonucleoside triphosphate binding(L=4),GO:0036094-small molecule binding(L=2),GO:0043167-ion binding(L=2),GO:0043168-anion binding(L=3),GO:0043169-cation binding(L=3),GO:0046872-metal ion binding(L=4),GO:0046914-transi

le activity(L=1),GO:0005488-binding(L=1),GO:0005524-ATP binding(L=7),GO:0008270-zinc ion binding(L=6),GO:0016301-kinase activity(L=4),GO:0016740-transferase activity(L=2),GO:0016772-transferase activity, transferring phosphorus-containin

le activity(L=1),GO:0005488-binding(L=1),GO:0005524-ATP binding(L=7),GO:0008270-zinc ion binding(L=6),GO:0016301-kinase activity(L=4),GO:0016740-transferase activity(L=2),GO:0016772-transferase activity, transferring phosphorus-containin

034061-DNA polymerase activity(L=5),GO:0036094-small molecule binding(L=2),GO:0043167-ion binding(L=2),GO:0043168-anion binding(L=3),GO:0043531-ADP binding(L=4),GO:0097159-organic cyclic compound binding(L=2),GO:1901265-nucleoside

399-primary active transmembrane transporter activity(L=4),GO:0015405-P-P-bond-hydrolysis-driven transmembrane transporter activity(L=5),GO:0016462-pyrophosphatase activity(L=5),GO:0016740-transferase activity(L=2),GO:0016772-transferase activi

ribonucleotide binding(L=6),GO:0035639-purine ribonucleoside triphosphate binding(L=4),GO:0036094-small molecule binding(L=2),GO:0043167-ion binding(L=2),GO:0043168-anion binding(L=3),GO:0043531-ADP binding(L=4),GO:0097159-organic

inase activity(L=5),GO:0004674-protein serine/threonine kinase activity(L=6),GO:0004721-phosphoprotein phosphatase activity(L=6),GO:0004722-protein serine/threonine phosphatase activity(L=7),GO:0004871-signal transducer activity(L=1),GO:0004872-

cule binding(L=2),GO:0043167-ion binding(L=2),GO:0043168-anion binding(L=3),GO:0043169-cation bi[illegible]-cation binding(L=3),GO:0046872-metal ion binding(L=4),GO:0046914-transition metal ion binding(L=5),GO:0097159-organic cyclic compound binding(L=2),GO:19012

: binding(L=4),GO:0032559-adenyl ribonucleotide binding(L=6),GO:0035639-purine ribonucleoside triphosphate binding(L=4),GO:0036094-small molecule binding(L=2),GO:0043167-ion binding(L=2),GO:0043168-anion binding(L=3),GO:0097159-org

le activity(L=1),GO:0005488-binding(L=1),GO:0005524-ATP binding(L=7),GO:0008270-zinc ion binding(L=6),GO:0016301-kinase activity(L=4),GO:0016740-transferase activity(L=2),GO:0016772-transferase activity, transferring phosphorus-containin

le activity(L=1),GO:0005488-binding(L=1),GO:0005524-ATP binding(L=7),GO:0008270-zinc ion binding(L=6),GO:0016301-kinase activity(L=4),GO:0016740-transferase activity(L=2),GO:0016772-transferase activity, transferring phosphorus-containin

le activity(L=1),GO:0005488-binding(L=1),GO:0005524-ATP binding(L=7),GO:0008270-zinc ion binding(L=6),GO:0016301-kinase activity(L=4),GO:0016740-transferase activity(L=2),GO:0016772-transferase activity, transferring phosphorus-containin

2549-ribonucleoside binding(L=4),GO:0032550-purine ribonucleoside binding(L=5),GO:0032553-ribonucleotide binding(L=3),GO:0032555-purine ribonucleotide binding(L=4),GO:0032559-adenyl ribonucleotide binding(L=6),GO:0035639-purine ribon

399-primary active transmembrane transporter activity(L=4),GO:0015405-P-P-bond-hydrolysis-driven transmembrane transporter activity(L=5),GO:0016462-pyrophosphatase activity(L=5),GO:0016740-transferase activity(L=2),GO:0016772-transferase activi

i5639-purine ribonucleoside triphosphate binding(L=4),GO:0036094-small molecule binding(L=2),GO:0043167-ion binding(L=2),GO:0043168-anion binding(L=3),GO:0097159-organic cyclic compound binding(L=2),GO:1901265-nucleoside phosphate

le activity(L=1),GO:0005488-binding(L=1),GO:0005524-ATP binding(L=7),GO:0008270-zinc ion binding(L=6),GO:0016301-kinase activity(L=4),GO:0016740-transferase activity(L=2),GO:0016772-transferase activity, transferring phosphorus-containin

le activity(L=1),GO:0005488-binding(L=1),GO:0005524-ATP binding(L=7),GO:0008270-zinc ion binding(L=6),GO:0016301-kinase activity(L=4),GO:0016740-transferase activity(L=2),GO:0016772-transferase activity, transferring phosphorus-containin

ribonucleotide binding(L=6),GO:0035639-purine ribonucleoside triphosphate binding(L=4),GO:0036094-small molecule binding(L=2),GO:0043167-ion binding(L=2),GO:0043168-anion binding(L=3),GO:0097159-organic cyclic compound binding(L=2

le activity(L=1),GO:0005488-binding(L=1),GO:0005524-ATP binding(L=7),GO:0008270-zinc ion binding(L=6),GO:0016301-kinase activity(L=4),GO:0016740-transferase activity(L=2),GO:0016772-transferase activity, transferring phosphorus-containin

Table S5. Statistics of orthology genes within eight plant species in a comparative study

	H. macrophylla	*C. acuminata*	*D. carota*	*H. annuus*	*M. guttatus*	*S. lycopersicum*	*V. darrowii*	*V. vinifera*
Number of genes	71,773	51,735	32,113	52,243	28,140	34,074	34,809	31,845
Number of genes in orthogroups	65,958	45,980	28,854	43,247	25,744	28,798	32,717	27,102
Number of unassigned genes	5,815	5,755	3,259	8,996	2,396	5,276	2,092	4,743
Percentage of genes in orthogroups (%)	91.90	88.90	89.90	82.80	91.50	84.50	94.00	85.10
Percentage of unassigned genes (%)	8.10	11.10	10.10	17.20	8.50	15.50	6.00	14.90
Number of orthogroups containing species	17,380	15,694	14,380	16,649	14,419	15,072	15,098	15,629
Percentage of orthogroups containing species (%)	59.10	53.40	48.90	56.60	49.10	51.30	51.40	53.20
Number of species-specific orthogroups	2,967	1,470	1,123	2,557	601	835	1,028	850
Number of genes in species-specific orthogroups	27,126	5,175	5,314	11,214	2,863	4,532	5,937	2,936
Percentage of genes in species-specific orthogroups (%)	37.80	10.00	16.50	21.50	10.20	13.30	17.10	9.20

Table S6. Mapping of known Inflorescence markers in the developed hydrangea genomes

Approach	Marker	Marker name	Chromosome	Location (Endless Summer Primary)	Location (Endless Summer Haplotype)	Location (Veitchii Primary)	Location (Veitchii Haplotype)	Reference	Note
GWAS	SNP	TP279504	4	116,702,580-116,702,782	73,832,199-73,832,401	90,261,590-90,261,792	63,976,900-63,977,102	Wu et al., 2020	GWAS
Genetic mapping								Unpublished	F1 population GBS-UNEAK
Genetic mapping	SSR/Contig	LC225531.1	4	126,623,084-126,621,315	81,422,780-81,424,552	99,489,532-99,491,303	n/a	Waki et al., 2018	F2 population
Association mapping	SNP	A109A110	4	116,278,511-116,278,708	73,401,684-73,401,886	89,895,935-89,896,141	n/a	Trankner et al., 2019	Di- and Tri-ploid
	InDel/haploid	A133A134	4	n/a	75,574,905-75,575,018	n/a	63,631,734-63,631,931		

Table S7. Sequences of BAM3 in select hydrangea cultivars.

Cultivar	BAM3 Amplified region
Double_Delights/DoubleFlower/1-167	TCTGGCAGATTCCTCCTGACATCAGTAATTGTCTGTCCCTGAGTTACTTCGATTTGAGCCAAAACCAACTTT<br>CAGGTCCAATCCCGGTTCAGATTTCTCAAATTCACATATTGAATTATCTGAACATATCTTGGAATCGCATGA<br>ACCAGAGCCTCCCCAAGGAAATA
Forever_and_Ever/DoubleFlower/1-167	CAGGTCCAATCCCGGTTCAGATTTCTCAAATTCACATATTGAATTATCTGAACATATCTTGGAATCGCATGA
Doublicious/DoubleFlower/1-167	CAGGTCCAATCCCGGTTCAGATTTCTCAAATTCACATATTGAATTATCTGAACATATCTTGGAATCGCATGA
Fuji_waterfall/DoubleFlower/1-167	CAGGTCCAATCCCGGTTCAGATTTCTCAAATTCACATATTAAATTATCTGAACATATCTTGGAACCGCATGA
Uzu_azisai/SingleFlower/1-167	CAGGTCCAATCCCGGTTCAGATTTCTCAAATTCACATATTGAATTATCTGAACATATCTTGGAATCGCATGA
Veitchii/SingleFlower/1-167	CAGGTCCAATCCCGGTTCAGATTTCTCAAATTCACATATTGAATTATCTGAACATATCTTGGAATCGCATGA
Endless_Summer/SingleFlower/1-167	CAGGTCCAATCCCGGTTCAGATTTCTCAAATTCACATATTAAATTATCTGAACATATCTTGGAATCGCATGA
