# Supplementary figures and images for: Two haplotype-resolved genomes reveal important flower traits in bigleaf hydrangea (Hydrangea macrophylla) and insights into Asterid evolution
Source: Hortic Res. 2023 Nov 9;10(12):uhad217. doi: 10.1093/hr/uhad217 (PMC10734616; doi:10.1093/hr/uhad217)

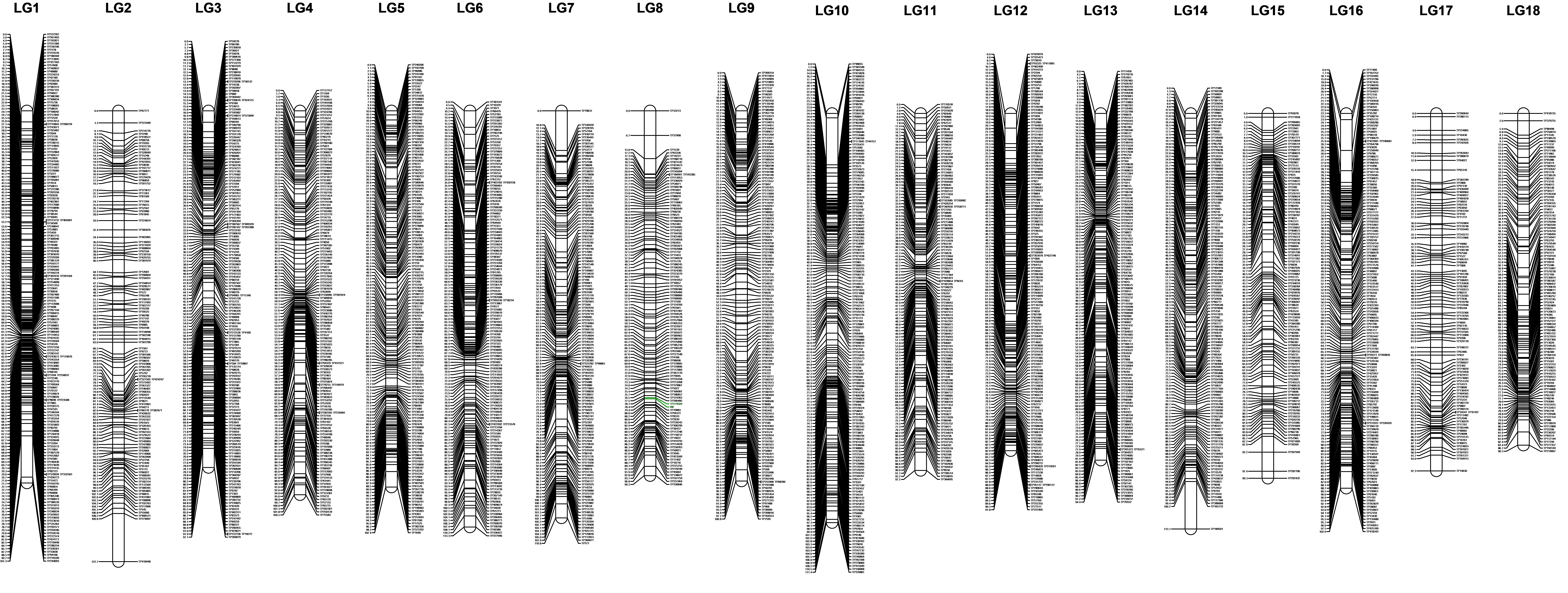

Supplement: Web_Material_uhad217 [file web_material_uhad217.zip › Figure S4.tif]
